# Ru(II)-Catalyzed C–H Sulfonamidation of the Medicinally Relevant Benzodiazepine Scaffolds

**DOI:** 10.1021/acs.orglett.6c02186

**Published:** 2026-07-22

**Authors:** Shourabh Rav, Shiv Shankar Gupta, Sarthi, Storm Hassell-Hart, Goose Markby, Hazel Cox, John Spencer, Upendra Sharma

**Affiliations:** † C−H Activation & Phytochemistry Lab, Chemical Technology Division, CSIR-Institute of Himalayan Bioresource Technology, Palampur 176061, India; ∥ Academy of Scientific and Innovative Research (AcSIR), Ghaziabad 201002, India; ‡ Sussex Drug Discovery Centre, School of Life Sciences, 1948 University of Sussex, Falmer, Brighton BN1 9QJ, U.K.; § Department of Chemistry, University of Sussex, Falmer, Brighton BN1 9QJ, U.K.

## Abstract

A late-stage, efficient C–H functionalization of medicinally relevant 1,4-benzodiazepines for direct C–H sulfonamidation has been developed via Ru-catalyzed C–H activation. Tosyl azide (TsN_3_) serves as a bench-stable sulfonamidating reagent. The sulfonamidated product is further converted to an aminated product through chemoselective hydrolysis of the sulfonamide bond. In mechanistic studies, a ruthenacycle (**BDZ-Ru-Cl**) has been synthesized and characterized by NMR spectroscopy and SC-XRD. Additionally, a novel intermediate, **Int-C** (a six-membered N–SO_2_Ar-inserted species), was synthesized from **BDZ-Ru-Cl**, providing further support for the proposed mechanistic pathway. Density functional theory (DFT) studies were also conducted to gain deeper mechanistic insight and to validate the developed protocol.

## Graphical abstract

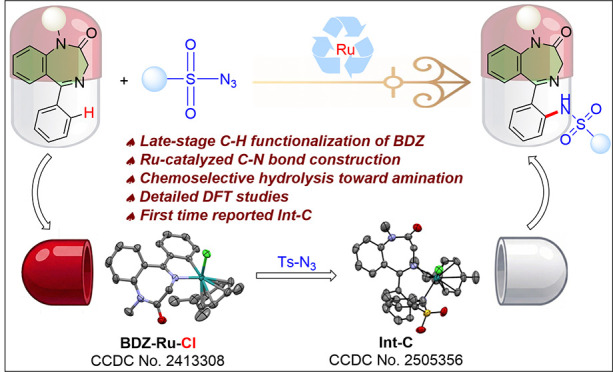

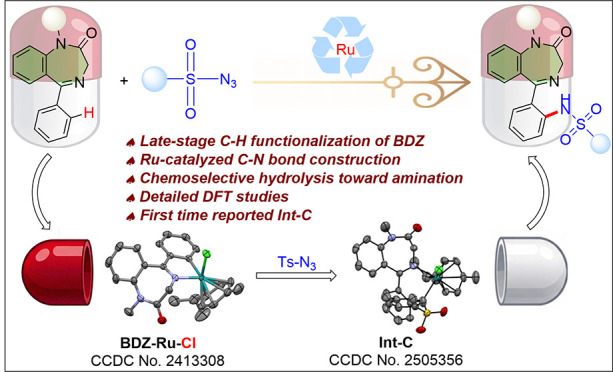

The production of diverse compounds through atom-economical and late-stage C–H functionalization approaches offers a valuable route for developing bioactive chemical products in the medicinal chemistry field.^1^ Late-stage C–H functionalization is an efficient tool for generating biologically active novel molecules, presenting further opportunities for discovering therapeutic candidates.^2^ Producing a library of closely related analogues of natural or complex drug molecules is crucial in drug discovery as it accelerates hit-to-lead optimization by refining bioactive cores to enhance activity, selectivity, pharmacokinetics, and reduce toxicity.^3^ Among various important heterocycles, 1,4-benzodiazepines (BDZs) stand out for their broad pharmacological activities, particularly in treating anxiety, insomnia, seizures, and muscle spasms.^4^ This scaffold includes well-known pharmaceuticals such as diazepam, alprazolam, clonazepam, midazolam, and flurazepam. Due to their substantial medicinal applications, BDZs remain a central focus in drug discovery, driving efforts to functionalize them to enhance their therapeutic potential.

In this context, chelation-assisted C–H functionalization is a valuable tool for introducing various functionalities into important *N*-heterocycles.^5^ The first report on BDZ C–H activation was presented by Stoccoro, followed by the Spencer group, who conducted detailed C–H activation studies leading to the synthesis of a stable BDZ palladacycle.^6^ Further advances were made by the Cintrat group by introducing halogen functionality at the 2’ position of BDZs using Pd-catalysis (Figure 1).^7^ In 2016, the Hong group demonstrated the C–H phosphorylation of BDZs under Rh-catalysis.^8^ The Spencer group later disclosed efficient Pd-catalyzed and photoredox methods for the direct C–H arylation of BDZs (Figure 1).^9^ In 2020, the Xie group disclosed the electrochemical and Ir-catalyzed C–H alkynylation of the BDZ moiety under mild conditions (Figure 1).^10^ Later in 2023 the Ackermann group reported ruthenium-catalyzed C–H amidation,^11^ and in 2026 a cobalt-catalyzed C–H amidation (Figure 1).^12^

**Figure 1:**
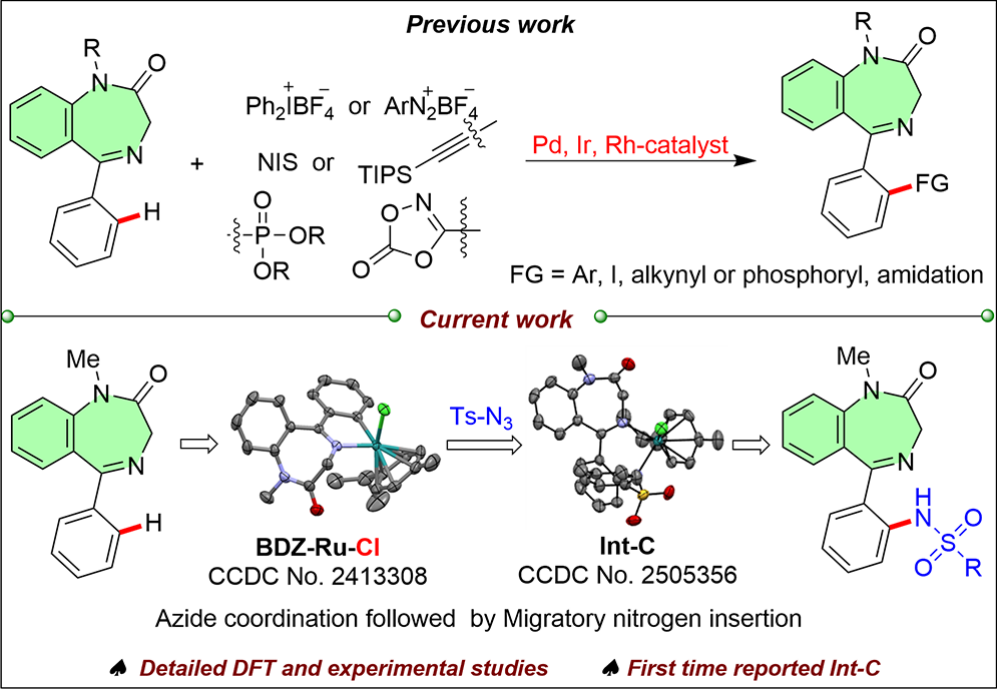
Ru-catalyzed vs other metal-catalyzed C–H functionalization strategies.

Despite their importance as drug scaffolds, further diversification of BDZs has to date been limited to direct C–H activation. Moreover, C–N bond formation via C–H metalation is a fascinating approach for introducing nitrogen into medicinal chemistry libraries. The direct C–H sulfonamidation with TsN_3_ has been explored for other scaffolds under different metal-catalysis, but there are only a few reports with a ruthenium catalyst.^13^ Our group has recently disclosed the C–H amidation with Ir and Co-based catalysis.^14^ However, the C–H sulfonamidation of BDZs with TsN_3_ under Ru-catalysis has not been reported and adds to an increasing portfolio of manipulations of key pharmaceuticals via cyclometalated ruthenium complexes.^15^

An efficient tool has been established to install the sulfonamide functionality at the C2′ of BDZs under Ru-catalysis (Figure 1).

Optimization studies commenced by reacting BDZ (**1a**) and tosyl azide (**2a**, 1.5 equiv) in the presence of [Ru­(*p*-cymene)­Cl_2_]_2_ (5 mol%), AgSbF_6_ (20 mol%), and AcOH (1 equiv) in HFIP at 100 °C for 24 h to obtain the amidated product (**3a**) in 40% yield (Table E2, See the ESI, entry 5). The product was confirmed by NMR spectroscopy and SC-XRD. In the absence of the ruthenium catalyst, no product was observed, and omitting MesCOOH reduced the yield to 30% (Table E2, entries 2–3). Using 30 mol% of MesCOOH produced only 40% of the desired product (Table E2, entry 4). Replacing Ru with a Co-based catalyst led to a drastic decrease in yield (Table E2, entry 6). Solvent screening revealed HFIP as the solvent of choice for the current reaction (Table E2, entries 7–10). Reducing the reaction time and temperature further decreased the yield (Table E2, entries 11–12). Ultimately, the optimal conditions afforded the desired sulfonamidated product **3a** in 82% yield (Table E2, entry 1).

Under optimal conditions, substrate scope studies were performed with various BDZ derivatives to produce the desired sulfonamidated derivatives (Table 1). Benzenesulfonyl azide (**2b**) served as the reactant and afforded the anticipated product (**3b**) in 55% yield. A sulphonyl azide bearing an electron-withdrawing group at the C-4 position (**2c**) resulted in a 60% yield of **3c**, while the electron-donating variant provided an excellent yield 90% of **3d.** The amide-substituted sulfonyl azide (**2e**) at the *para* position gave the desired product (**3e**) in 57% yield, and the fluoro-substituted azide produced **3f** in 54% yield. Naphthyl sulfonylazide also yielded the desired sulfonamidated product, albeit in lower yield (**3g**). Thiophene-based sulfonyl azide also furnished the corresponding product **3h** in a 36% yield. Further aliphatic sulfonyl azides were evaluated, providing the desired products in 21–42% yields (**3i–3j**).

**Table 1:**
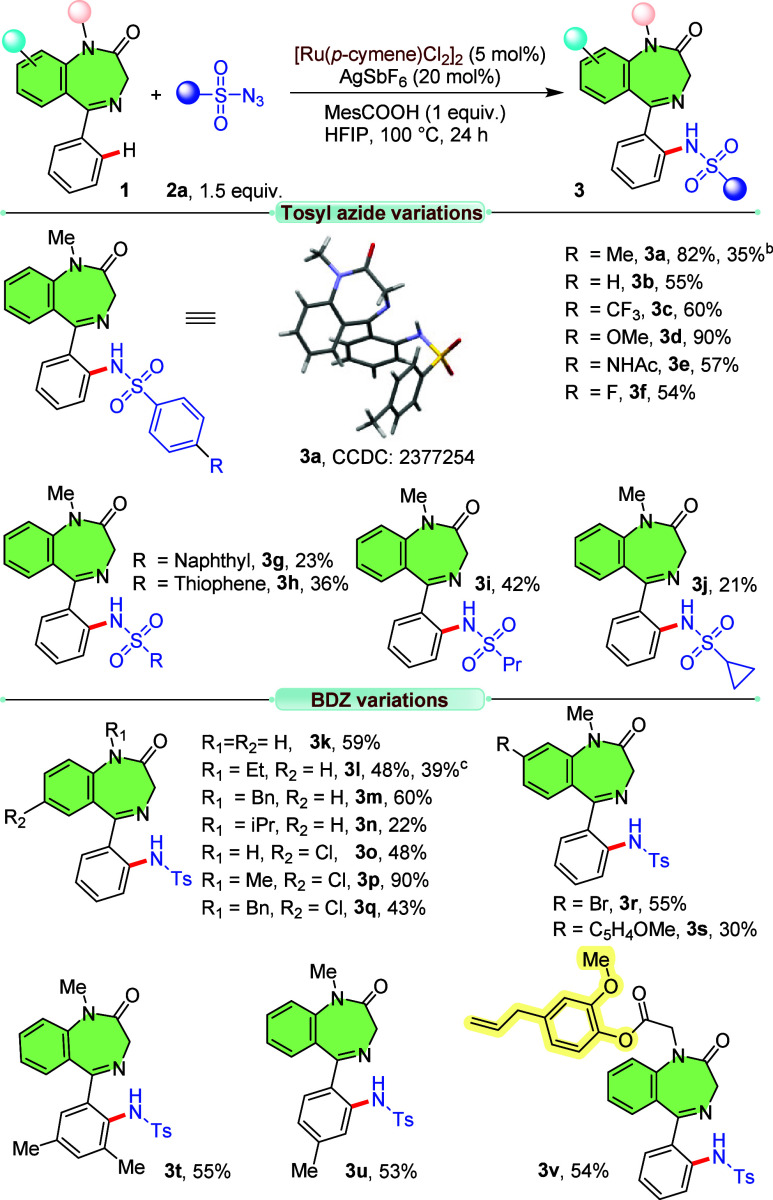
Substrate Scope of BDZ and Azides^a^

a Reaction conditions: **1a** (0.2 mmol), **2a** (1.5 equiv), [Ru­(*p*-cymene)­Cl_2_]_2_ (5 mol%), AgSbF_6_ (20 mol%), MesCOOH (1 equiv) in HFIP (0.2 M) at 100 °C for 24 h.

b 6 mmol scale.

c 4 mmol scale.

Various BDZ derivatives reacted smoothly, yielding the desired products in moderate to good yields (Table 1). BDZs with different *N*-substituents yielded the corresponding sulfonamidated products (**3k**-**n**) in 22–60% yields. The introduction of a chloro group at the C-7 position of BDZ (**3o**), along with *N*-substituent variations, afforded sulfonamidation products in 43–90% yields (**3o**–**3q**). Additionally, a bromo substituent was tolerated, and 8-bromo and 8-(*p*-OMe-C_6_H_4_) substituted BDZs afforded the desired products in 30–55% yields (**3r**–**s**). The presence of substituents on the phenyl ring undergoing functionalization was not deleterious to the sulfonamidation process and provided the desired products in 53–55% yields (**3t**–**u**). A Eugenol (4-allyl-2-methoxyphenol)-derived BDZ also reacted smoothly to produce the corresponding sulfonamidated product (**3v)** in 54% yield. Scale-up reaction was also performed by reacting **1a** and **1l** with **2a** under developed reaction conditions. (See the ESI).

To demonstrate the synthetic utility of the sulfonamidated product, it was chemoselectively converted into the corresponding primary amine containing BDZs **4,** which have potential uses as building blocks, by treatment with sulfuric acid (Scheme 1).

**Scheme 1:**
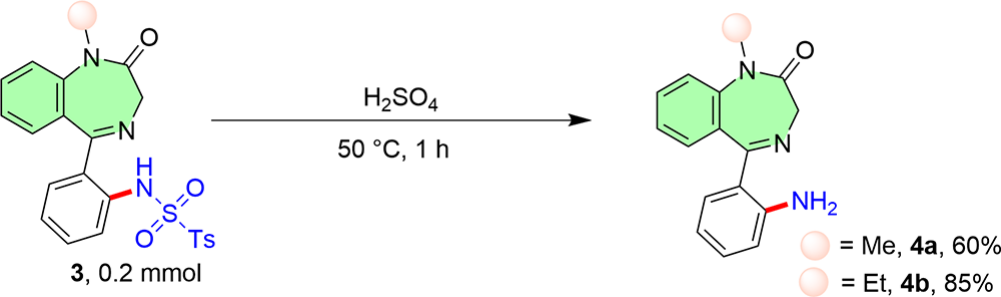
Chemoselective Hydrolysis of the Sulfonamide Bond

Control experiments were conducted to gain mechanistic insights into this interesting sulfonamidation process. First, radical quenching experiments were performed using 2 equiv of TEMPO ((2,2,6,6-tetramethylpiperidin-1-yl)­oxidanyl) and BHT (butylated hydroxytoluene), yielding 48% and 80% yields of the desired product **3a**, respectively (See the SI). These experiments suggest a nonradical pathway under current reaction conditions. Deuterium-labeling studies were conducted using deuterated acetic acid under standard conditions, yielding 26% deuteration at the C-2′ position of the phenyl ring. This experiment indicated that the reaction might involve a reversible C–H activation step. Additionally, 10% and 9% deuterium incorporation were also observed at the methylene positions of the 7-membered ring, likely due to previously reported tautomerism^9a,16^ (See the ESI) .

Further, the ruthenacycle (**BDZ-Ru-Cl**) was synthesized in 60% yield and characterized by NMR, SC-XRD, and HR-ESI-MS (Scheme 2a).^17^ To investigate the intermediacy of a ruthenacycle (see cationic species in Scheme 3), a standard reaction was conducted using **BDZ-Ru-Cl** as precatalyst in place of the [Ru­(*p*-cymene)­Cl_2_]_2_, to afford the desired product **3a** (Scheme 2b), indicating the involvement of the ruthenacycle. **BDZ-Ru-Cl** was employed as a stoichiometric reagent in the reaction at both room temperature and 100 °C (Scheme 2c). The results showed an 80% yield at 100 °C, whereas the yield was significantly lower at room temperature, highlighting the need for elevated temperatures for the reaction to proceed effectively. Next, we continued investigating the reaction mechanism by synthesizing additional ruthenacycle intermediates. Gratifyingly, upon reaction of BDZ-Ru-Cl with Ts-N_3_, we were able to synthesize **Int-C** in 65% isolated yield, thereby confirming the role of **Int-B** in the catalytic reaction mechanism. (Note that in the catalytic cycle, Scheme 3, this involves a cationic species rather than a Ru–Cl bond). The structure of **Int-C** was unambiguously confirmed by NMR and SC-XRD (Scheme 2d).

**Scheme 2:**
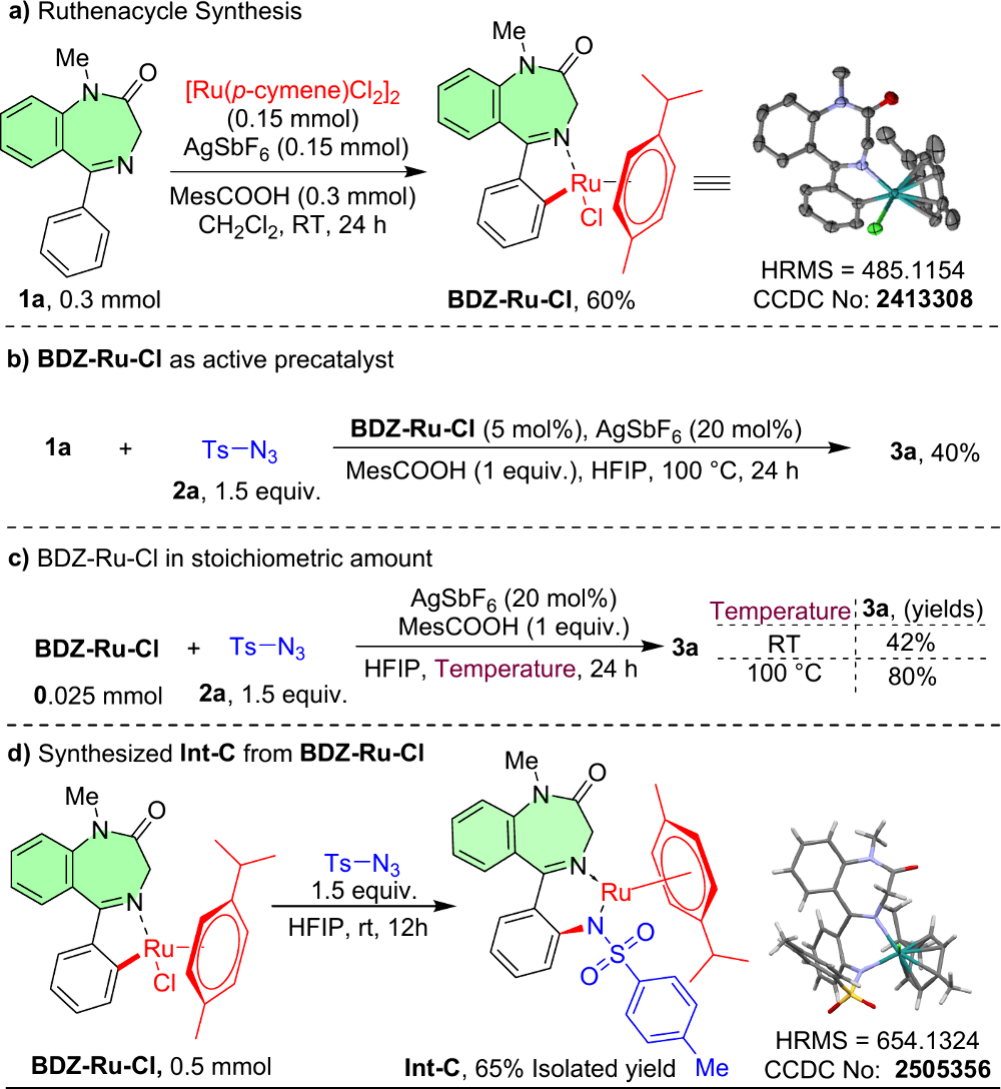
Synthesis of Intermediates and Its Utility in the Mechanistic Studies

**Scheme 3:**
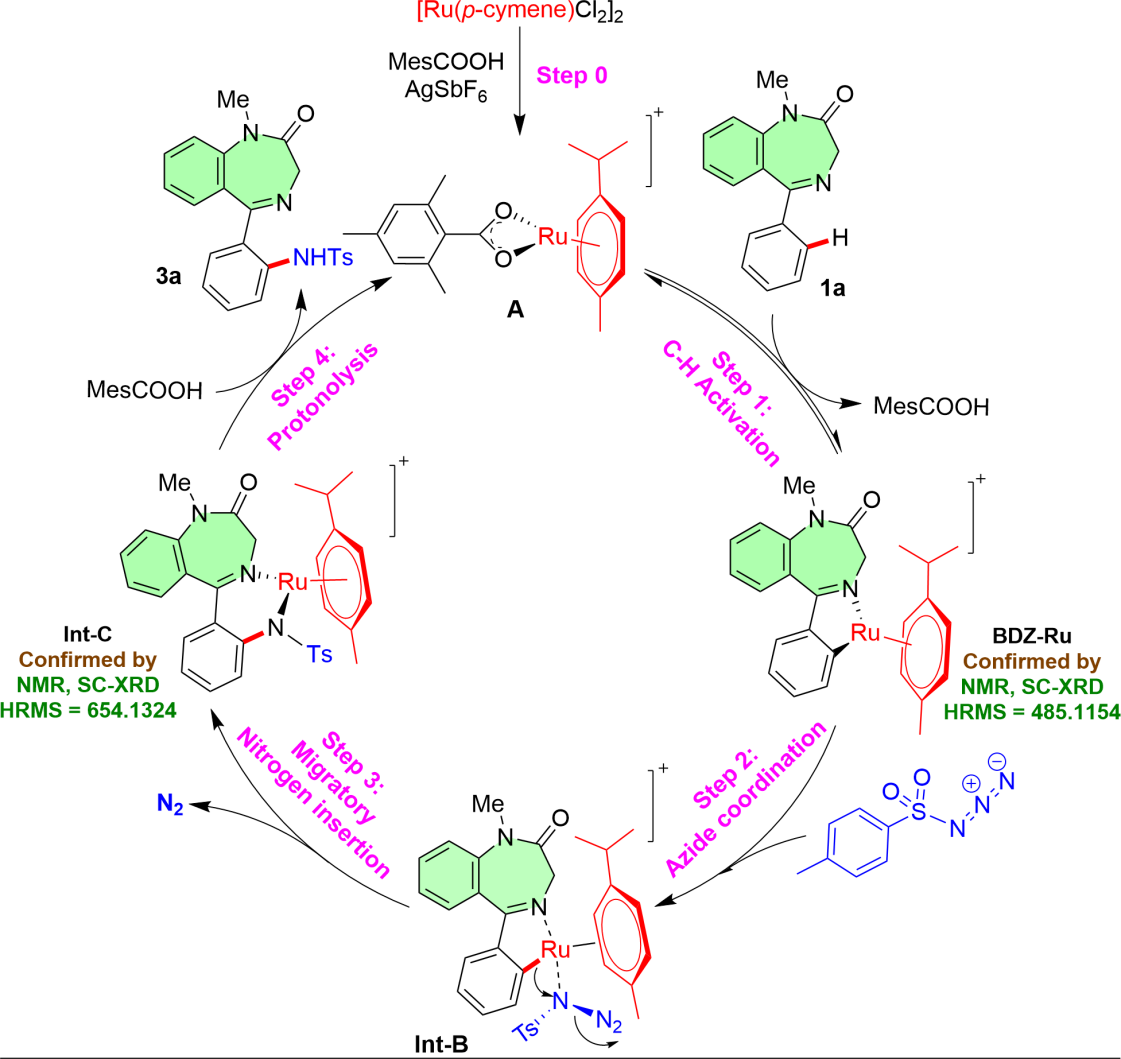
Catalytic Cycle for BDZ C–H Sulfonamidation

To further investigate the role that **BDZ-Ru-Cl** plays in the catalytic cycle, DFT calculations were performed to determine the relative stability of the intermediates (minima) at each step in the cycle at the PBE0/def2-TZVP-LanL2TZ­(f) level of theory.^18–20^ Initially, calculations were performed to elucidate the role of the Cl in the formation of the active catalyst (step 0 of Scheme 3). It was found that it is considerably more favorable, to lose both chlorine atoms on each Ru center of the precursor [Ru­(*p*-cymene)­Cl_2_]_2_ rather than just one. This is feasible based on the stoichiometry, i.e. 4 equivalents of AgSbF_6_ to the Ru catalysis, and agrees with the assertion by Chang et al.^23^ who reported that the neutral dimeric ruthenium precursor [Ru­(*p*-cymene)­Cl_2_]_2_ was converted to the cationic species by the silver salt.

The relative Gibbs free energy profile and structures for the minima involved in the proposed mechanism (Scheme 3) are provided in Figure 2. The active catalyst **A** is set as the free energy reference (0 kJ mol^–1^). The species **A** reacts with the proximal C–H bond of **1a**, forming a five-membered ruthenacycle (**BDZ-Ru**). This is an exergonic process in the gas phase and slightly endergonic with solvent correction. The next step involves the azide coordination. The tosyl azide can coordinate to BDZ-Ru via the terminal nitrogen or via the nitrogen coordinated to the sulfur, to form **Int-B**. Three geometric conformers for **Int-B** were confirmed as minima. A natural population analysis and Quantum Theory of Atoms in Molecules (QTAIM)^21,22^ analysis of TsN_3_ confirmed that the N bound to the sulfur atom has a much greater negative charge than the terminal N (See the SI, Figure S3). However, coordination of TsN_3_ to BDZ-Ru via the terminal N is slightly more favorable due to reduced steric hindrance. Nevertheless, the energy difference between conformers is small (<20 kJ mol^–1^) and coordination through the N coordinated to S facilitates the migratory nitrogen insertion step, hence this was used to study the subsequent steps. The orbitals involved in the N–Ru bond and the electron density contour map are provided in the ESI (Figures S4–S7).

**Figure 2:**
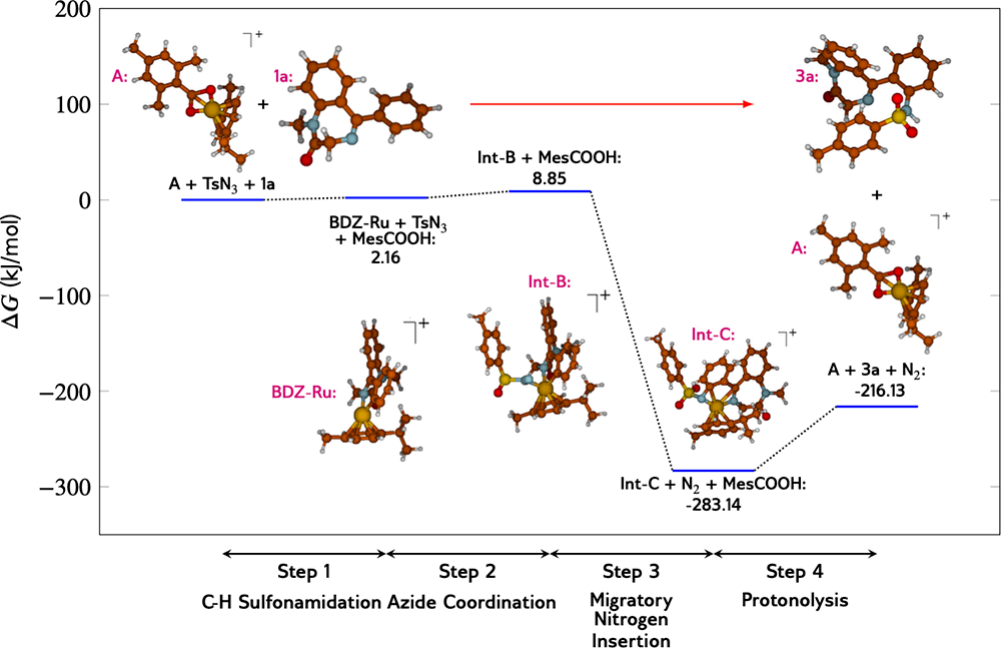
Gibbs free energy profile for the intermediates in the proposed mechanism for the catalytic cycle (Scheme 3) calculated at the PBE0/def2-TZVP-LanL2TZ­(f) level of theory, with a HFIP solvent correction.

In QTAIM analysis, chemical bonding can be characterized by locating bond critical points (BCPs): the point where the electron density becomes a minimum value along the bond path between interacting atoms, ESI Table S4 provides the optimized Ru–N bond lengths and QTAIM parameters in the key intermediates.

This analysis reveals that the Ru–N­(BDZ) parameters in **Int-B** are similar to those in **BDZ-Ru** and that the Ru–N­(TsN_3_) is weaker and less covalent in character than the Ru–N­(BDZ) bond in **Int-B**; this is further supported by the optimized bond lengths. The formation of **Int-B** is slightly endergonic by 6.69 kJ/mol (and exergonic by 22.71 kJ/mol without solvent correction). Species **Int-B** subsequently undergoes migratory insertion of amido nitrogen across the C–Ru bond, yielding the 6-membered species **C** and releasing N_2_.

As shown in Figure 2, the cationic species **Int-C**, is the most stable intermediate in the catalytic cycle. A QTAIM analysis revealed a stabilizing interaction (BCP) between an oxygen atom from the TsN_3_ and the Ru atom. The topology of **Int-C** is shown in Figure 3. The bond lengths have contracted due to the formation of the 6-membered ring, which is more stable than the 5-membered ring due to reduced bond angle strain. The magnitude of ρ­(r) at the BCP of the Ru–N bond arising for the tosyl group (ESI Table S4) indicates that the bond is slightly stronger, but the nature is similar to the interaction between Ru and TsN_3_ in **Int-B**. The unexpected Ru–O interaction is weaker than the Ru–N interactions, as indicated by the magnitude of the electron density ρ­(r) at the BCP, but it too has a similar nature. The bond ellipticity value, ε, reflects a greater π character contribution than σ character which is largest for the Ru–O and Ru–N­(BDZ) bonds. The relatively low delocalization index δ­(Ru–N) value indicate minimal covalent character. The formation of a carbon–nitrogen bond during the migratory nitrogen insertion to form a sulfonamidated benzodiazepine, is the crucial step in the catalytic cycle. From the QTAIM investigation on the newly formed C–N bond in **Int-C** and **3a**, it is shown that the bond is covalent due to the negative Laplacian of electron density, and the ellipticity indicates that the bond is mostly σ-character (ESI Table S4).

**Figure 3:**
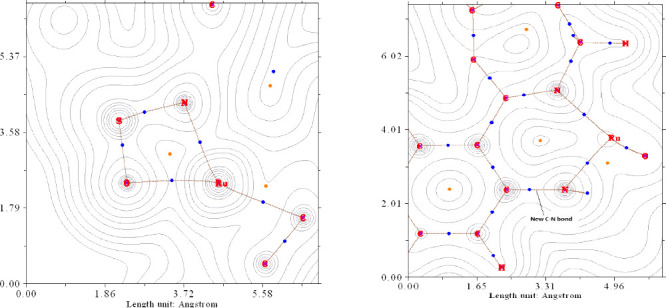
Contour graph of C highlighting a) the stabilizing Ru–O bond, and b) the new C–N bond and the 6 membered ring. BCPs are blue dots, yellow dots are RCPs and the brown lines are bond paths.

The final step is protonolysis, in the presence of MesCOOH, which leads to the formation of the desired sulfonamidated product **3a** along with the regeneration of the active catalytic species **A** to continue the catalytic cycle (Scheme 3). This protonolysis step is an endergonic process, with an overall reaction energy of +67.27 kJmol^–1^. As shown in the ESI Figure S2, the effect of applying the HFIP solvent correction to the gas phase energies is, primarily, to reduce the stability of **Int-C** relative to the starting material and thus reduce the relative energy difference between **Int-C** and the final products.

In conclusion, an efficient methodology has been developed for the late-stage C–H sulfonamidation of BDZ-based molecules using Ru-catalysis. This protocol enables C–N bond formation of pharmaceutically relevant motifs, utilizing a cost-effective metal catalyst under simple conditions. Additionally, chemoselective deprotection of the sulfonamidated product was achieved *via* acid hydrolysis. Comprehensive mechanistic studies were conducted to elucidate the most efficient reaction pathway for C–N bond formation. Furthermore, to gain mechanistic insight, ruthenacycle (BDZ-Ru-Cl) and **Int-C** were synthesized and characterized, providing strong support for the proposed reaction mechanism (Scheme 3). This methodology represents a significant advancement for medicinal chemists, facilitating the investigation of medicinally important candidates in an atom-economical manner.

## Supplementary Material





## Data Availability

The data underlying this study are available in the published article and its Supporting Information.

## Notes

General comments, procedures, optimization details, analytic data, NMR spectra of products, crystallographic data, and computational analysis (PDF)
FAIR data, including the primary NMR FID files, for compounds **3a**–**3v**, **4a**, **4b**, **Int-C** and **BDZ-Ru** (ZIP)
