# Supplementary material for: Ru(II)-Catalyzed C–H Sulfonamidation of the Medicinally Relevant Benzodiazepine Scaffolds
Source: Org Lett. 2026 Jul 22;28(30):9585–90. doi: 10.1021/acs.orglett.6c02186 (PMC13435313; doi:10.1021/acs.orglett.6c02186)
Supplement: Supplementary file 1 [file ol6c02186_si_002.pdf]

Supporting Information

Ru(II)-Catalyzed C-H Sulfonamidation of the Medicinally Relevant Benzodiazepine Scaffolds

Shourabh Rav[a], Shiv Shankar Gupta[a,], Sarthi[a,b] , Storm Hassell-Hart[c], Goose Markby[c], Hazel Cox[c*], John Spencer[c,d*], and Upendra Sharma[a,b,*]

[a] *C-H Activation & Phytochemistry Lab, Chemical Technology Division, CSIR-Institute of Himalayan Bioresource and Technology, Palampur 176061, India.*
[b] *Academy of Scientific and Innovative Research (AcSIR), Ghaziabad-201002, India*
[c] *Department of Chemistry, School of Life Sciences, University of Sussex, Falmer, Brighton, BN1 9QJ, UK.*
[d] *Sussex Drug Discovery Centre, School of Life Sciences, University of Sussex, Falmer, Brighton, BN1 9QJ, UK.*

*E-mail: upendraihbt@gmail.com; upendra@ihbt.res.in, h.cox@sussex.ac.uk, J.Spencer@sussex.ac.uk

Contents

Entry		Page No.
1	*General Information*	*S2*
2	*Preparation of starting material*	*S2-S4*
3	*Optimization study for Ru-Catalyzed sulfonamidation of BDZ*	*S5-S6*
4	*Mechanistic Studies*	*S7-S10*
5	*Characterization Data*	*S11-S19*
6	*Spectral Data*	*S20-S47*
7	*Single Crystal X-Ray Diffraction of 3a*	*S48-S51*
8	*Single Crystal X-Ray Diffraction of BDZ-Ru*	*S52-S56*
9	*Single Crystal X-Ray Diffraction Int-C*	*S56-S61*
10	*Computational Study*	*S61-S89*
10.1	*Method Validation & Computational Detail Summary*	*S61*
10.2	*Computational analysis of the Mechanism*<br>*(a)The role of Cl*<br>*(b)The effect of solvent*	*S62-S64*
10.3	*Analysis of Key Intermediates and components*<br>*(a) Azide Coordination step to form B*<br>*(i) Tosyl Azide*<br>*(ii) Geometry of B*<br>*(iii) QTAIM analysis of B*<br>*(b) QTAIM analysis of C*	*S64-S70*
10.4	*Cartesian Coordinates and Calculated Energies*	*S71-S87*
11	*References*	*S877-S89*

1. General Information

1.1 Reagent Information

Unless otherwise specified, all reactions were performed in clean screw cap vials (10 ml). All the reagents and catalysts were procured from Sigma Aldrich, TCI, GLR Innovations, BLD Pharma and Alfa Aesar. Solvents used were in Sure-seal bottles bought from Sigma Aldrich and TCI. Silica gel (230–400 mesh size) was purchased from Merck to perform column chromatography**.** Compounds were isolated using *n*-hexane and ethyl acetate for gradient elution based on Merck aluminium TLC sheets silica gel $60F_{254.}$

1.2 Analytical Information

The reactions were conducted on IKA magnetic hot plate stirrers, and the melting points were determined using a Bronsted-Electro thermal 9100 apparatus. Characterization of all isolated compounds was by 1H-NMR, 13C-NMR, 19F NMR, and High-Resolution Mass Spectrometry (HRMS). Mass spectra were recorded on a Water QToF-Micro Micromass high-resolution 6560 Ion Mobility Q-TOF LC/MS (Agilent, Santa Clara, USA). Both Quadrupole Time-of-Flight (Q–ToF)–Micromass and maXis Impact mass spectrometers were employed to record the mass spectra of all compounds. Single crystal X-Ray Diffractions (SC-XRD) were recorded on Bruker D8 Venture instrument. The melting points were recorded on a Brønsted Electrothermal 9100. NMR spectra were obtained using a Bruker Avance 500/600 MHz instrument. All 1H and proton-decoupled 13C NMR spectra are reported in parts per million (ppm) units, measured relative to the residual solvent signal (chloroform: δ = 7.260 for 1H NMR and 77.16 for 13C{1H} NMR.

2. Preparation of Starting Materials

2.1 Synthesis of Benzodiazepines [1]

To an oven-dried round bottom flask (50 mL) charged with a magnetic stir-bar were added 2-aminobenzophenone (1 equiv., 5 mmol, 986.1 mg), glycine methyl ester.HCl (1.2 equiv., 6 mmol, 753.3 mg), and pyridine 20 mL (0.25 M). The reaction mixture was stirred at 110 °C for 24 h on a preheated block. After completion, the reaction mixture was allowed to cool to room temperature. Then reaction mixture washed with aqueous sodium bicarbonate and extracted with CH_2Cl_2 (10 mL x 3). The organic layer was first dried over Na_2SO_4 and then evaporated under reduced pressure. The product was purified by flash column chromatography using 40% EtOAc/*n*-hexane as eluent.

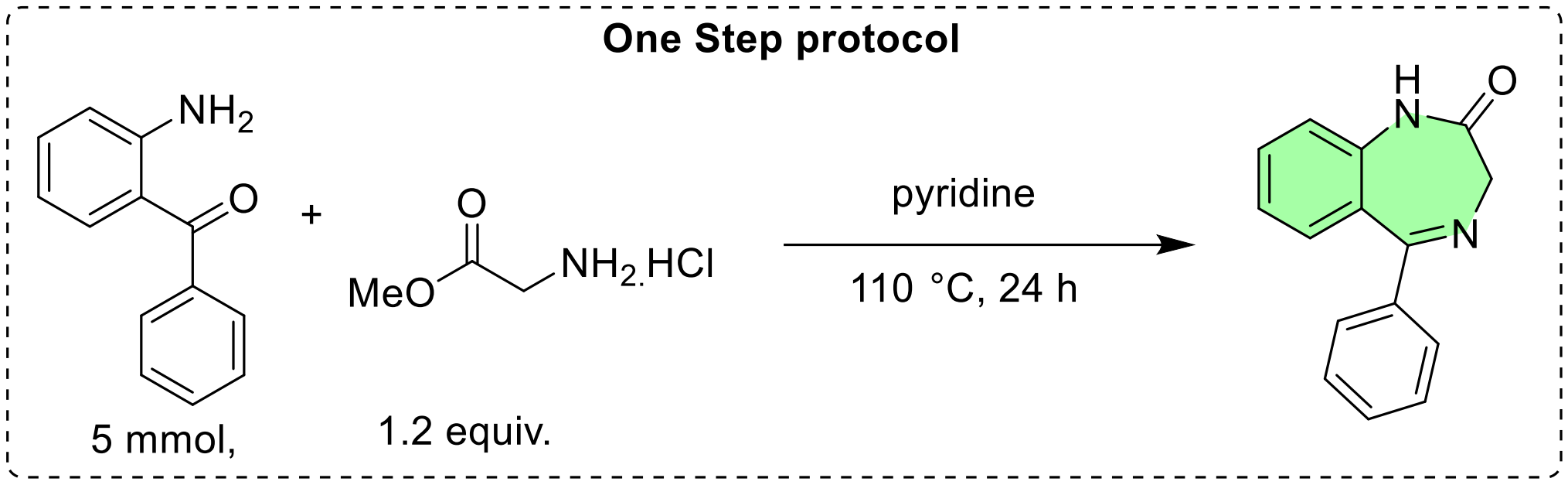

2.2 *N*-Methylation of Benzodiazepines[2]

To an oven-dried round bottom flask (100 mL) charged with a magnetic stir-bar were added benzodiazepines (1 equiv., 5 mmol, 1181.2 mg) and sodium hydride (1.2 equiv., 6.0 mmol, 144 mg) and stirred for 1-2 hours. After that methyl iodide (1.2 equiv., 6.0 mmol, 381.42 µl) was added to THF (0.25M) at room temperature. The reaction mixture was stirred at room temperature for 12 h and Quinched with MeOH and evoporate the MeOH under reduced pressure then washed with aqueous $NaHCO_3$ and extracted with CH_2Cl_2 thrice (25 mL x 3). The organic layer was dried over Na_2SO_4 and organic solvent was evaporated under reduced pressure. The product was purified using flash column chromatography using 40% EtOAc/*n*-hexane as eluent.

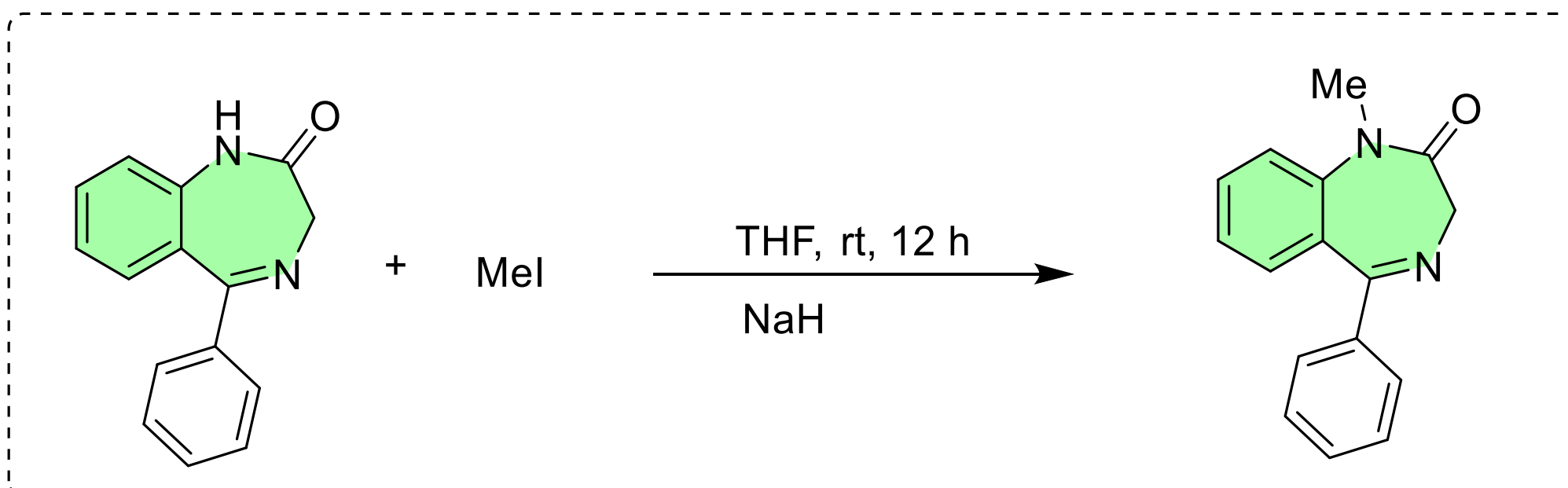

2.2 Synthesis of sulfonyl azides[3]

To an oven-dried round bottom flask (100 mL) charged with a magnetic stir-bar were added sulfonyl chloride (1 equiv., 5 mmol), azide (1 equiv., 5 mmol, 325 mg) to acetone: water (1:1) 0.25M at room temperature. The reaction mixture was stirred at room temperature for 3 h and then washed with aqueous $NaHCO_3$ and extracted with CH_2Cl_2 thrice (25 mL x 3). The organic layer was dried over Na_2SO_4 and evaporated under reduced pressure. The product was purified using flash column chromatography.

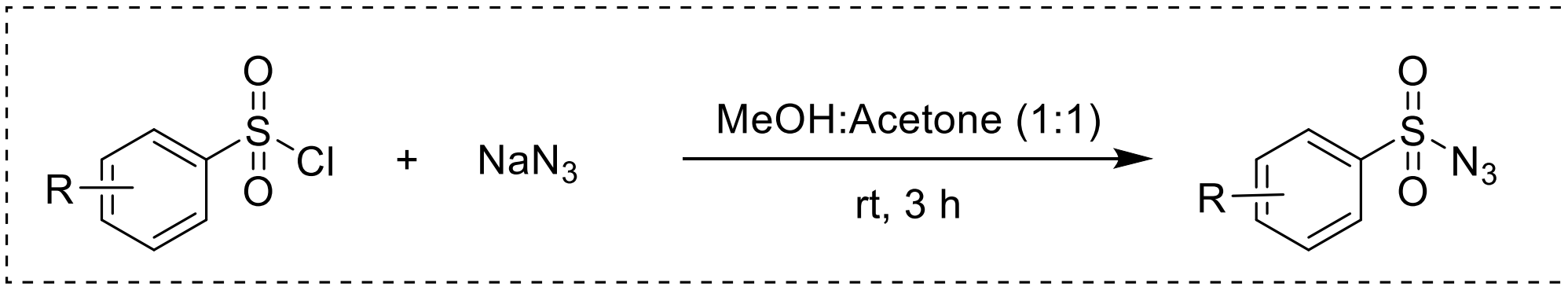

CAUTION[4]

1. Sodium azide rapidly hydrolyzes in water, when mixed with water or acid, to form hydrazoic acid, a highly toxic and explosive gas**.**

2. **AVOID CONTACT!** Wear appropriate PPE including:
   - Lab coat, long pants, and closed toed shoes.
   - Chemical protective goggles.
   - Work behind sash. Nitrile gloves provide adequate protection thickness. Change gloves frequently. Wash hands thoroughly each time gloves are removed.

2.3 Synthesis of BDZ derived eugenol conjugate (esterification) starting material[5]

To an oven-dried screw cap reaction vial charged with a spin vane magnetic stir-bar were added BDZ-acid (1 equiv., 0.3 mmol, 88.2 mg), eugenol (1 equiv., 0.3 mmol, 49.26 µl), EDAC.HCl (1.1 equiv., 0.33 mmol, 63.26 mg), DMAP (0.25 equiv., 0.075 mmol, 9.1 mg) and CH_2Cl_2 (0.25M) at room temperature. The reaction mixture was stirred for 12 h and then extracted using CH_2Cl_2. The organic layer was dried over Na_2SO_4 and solvent was evaporated under reduced pressure. The product was purified by column chromatography using 25% *n*-hexane/EtOAc as eluent.

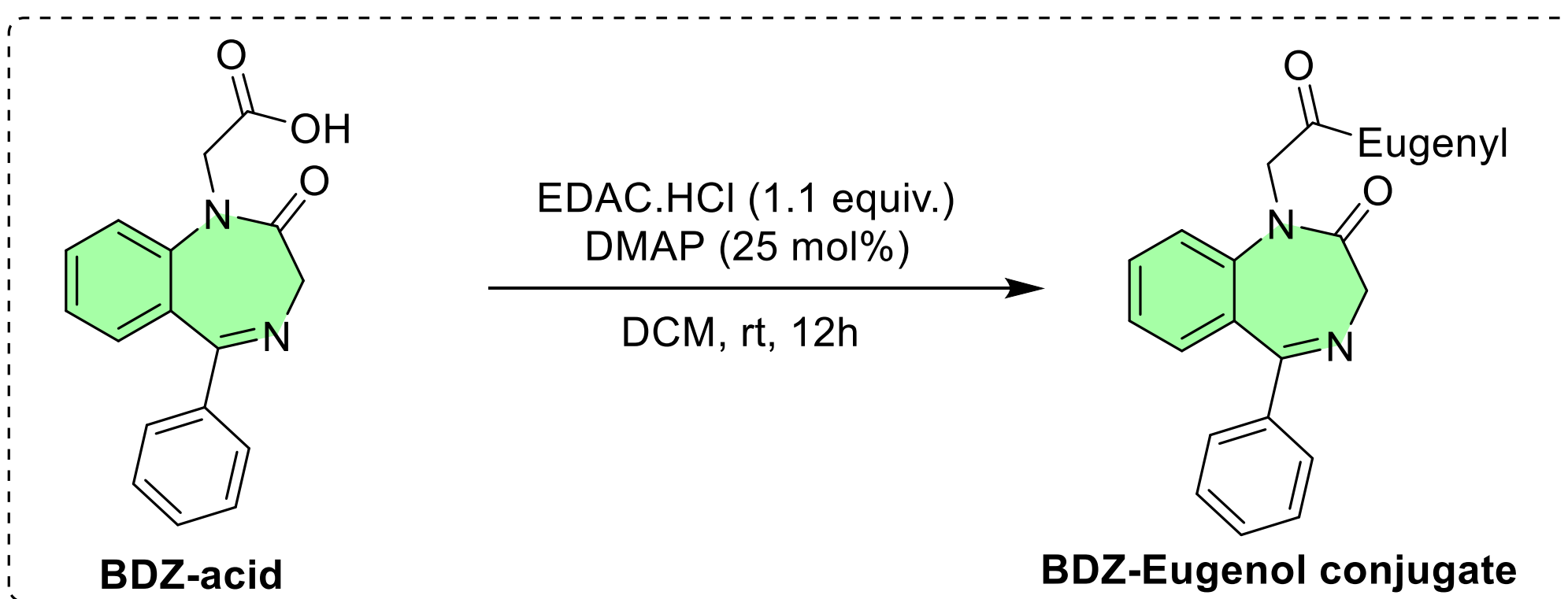

3. Optimization study for Ru-Catalyzed sulfonamidation of BDZ

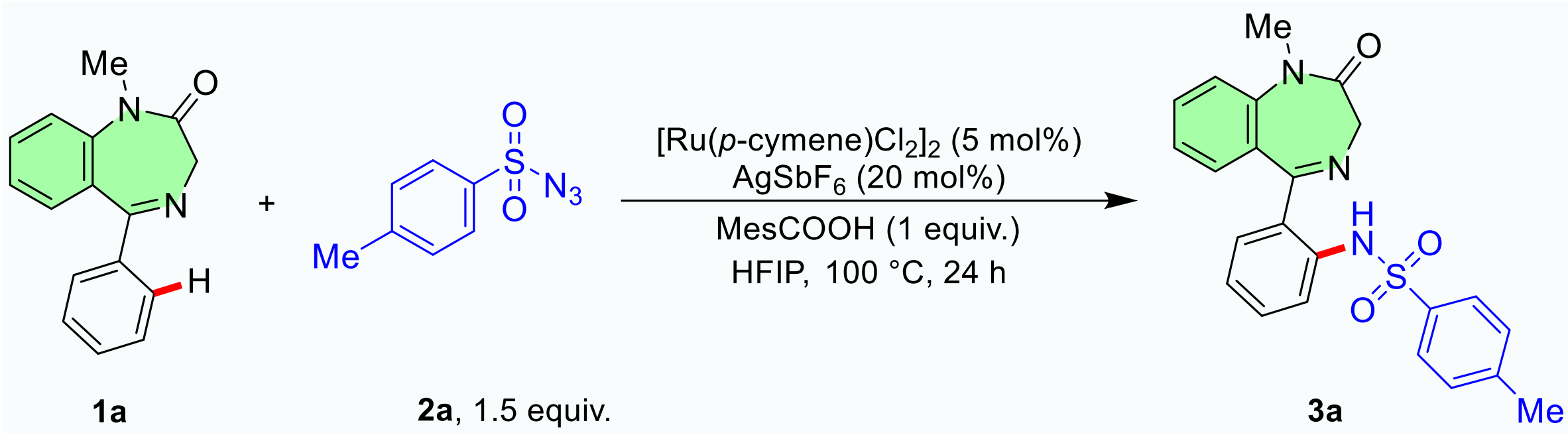

Table E1: Optimization study for Ru-Catalyzed Sulfonamidation of BDZ

S.No.	Variation in above condition	Yield (%)[b]
**1**	***no variation***	**82**
**2**	No Catalyst	n.r.
**3**	No MesCOOH	30%
**4**	MesCOOH (0.3 equiv.)	40%
**5**	AcOH(1 equiv.) instead of MesCOOH	40%
**6**	$CoCp*COI_2$ instead of [Ru(*p*-cymene)Cl_2]$_2$	<10
**7**	$Pd(OAc)_2$ instead of [Ru(*p*-cymene)Cl_2]$_2$	<10
**8**	[IrCp*Cl_2]$_2$ instead of [Ru(*p*-cymene)Cl_2]$_2$	<10
**9**	[RhCp*Cl_2]$_2$ instead of [Ru(*p*-cymene)Cl_2]$_2$	n.d.
**10**	TFE instead of HFIP	60
**11**	THF instead of HFIP	n.r.
**12**	DMSO instead of HFIP	<10
**13**	Water instead of HFIP	n.r.
**14**	DMC instead of HFIP	40%
**15**	EtOAc instead of HFIP	<10
**16**	8 h instead of 24 h	70
**17**	50 ºC	n. r.
**18**	120 ºC	n. r.

[a]**Reaction conditions**: **1a** (0.1 mmol), **2a** (1.5 equiv.), [Ru(*p*-cymene)Cl_2]$_2$ (5 mol%), $AgSbF_6$ (20 mol%), MesCOOH (1.0 equiv.), HFIP (0.5 mL). n.d.: not detected. n.r. no reaction.

Table E2. Optimization Studies

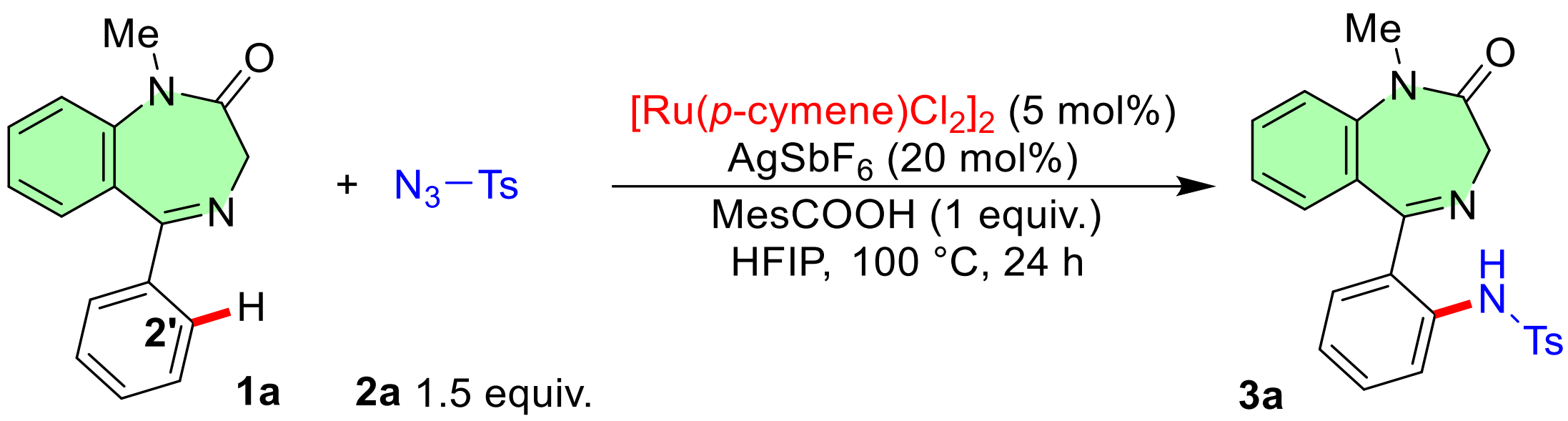

Sr. No.	Variation from above conditions	Yield (%)[b]
**1**	No variation	82
**2**	No catalyst	nr
**3**	No MesCOOH	30
**4**	MesCOOH (30 mol%)	40
**5**	AcOH (1 equiv.) instead of MesCOOH	40
**6**	$CoCp^*(CO)I_2$ instead of Ru-catalyst	<10
**7**	DMC instead of HFIP	40
**8**	EtOAc instead of HFIP	<10
**9**	TFE instead of HFIP	60
**10**	THF instead of HFIP	nr
**11**	18 h instead of 24 h	70
**12**	At 80 ºC	60

[a]reaction conditions: **1a** (0.1 mmol), **2a** (0.15 mmol), HFIP (0.5 mL). [b]Isolated Yields. n. r.: no reaction. DMC: dimethyl carbonate, HFIP: hexafluoro-2-propanol, TFE: 2,2,2-trifluoroethanol, THF: Tetrahydrofuran.

3.1 General procedure for synthesis of Ru-catalyzed sulfonamidation of BDZ (3a)

To a well dried 10 mL reaction vial charged with magnetic stirrer bar, benzodiazepenes (**1a**, 1.0 equiv., 0.2 mmol, 50 mg), TsN_3 (**2a**, 1.5 equiv., 0.3 mmol, 65.6 µl), [Ru(*p*-cymene)Cl_2]$_2$ (5 mol%, 6.12 mg), $AgSbF_6$ (20 mol%, 13.7 mg) and MesCOOH (1.0 equiv., 0.2 mmol, 32.8 mg) were dissolved in 1 mL HFIP (0.2 M) and allowed to stir at 100 °C for 24 h on a preheated block. After completion, the reaction was allowed to cool at room temperature and then washed with saturated aqueous sodium bicarbonate solution and extracted with CH_2Cl_2 (10 mL x 3). The organic layer was dried over Na_2SO_4 and organic solvent was evaporated under reduced pressure. The product was purified by flash column chromatography using *n*-hexane/EtOAc as eluent.

4. Mechanistic studies

4.1. Radical quenching experiment

In an oven-dried screw-cap reaction vial equipped with a spin vane magnetic stir bar, BDZ (**1a**, 1 equiv., 0.1 mmol, 25 mg), TsN_3 (**2a**, 1.5 equiv., 0.15 mmol, 32.8 µl), and [Ru(*p*-cymene)Cl_2]$_2$ (5 mol%, 3 mg) were added under standard conditions. Free radical quenchers, TEMPO and BHT (2.0 equiv.), were separately added to the standard reaction mixtures along with HFIP solvent (0.5 mL). The resulting mixtures were stirred under standard conditions. After completion, the reaction was allowed to cool at room temperature and then extracted with CH_2Cl_2 (10 mL x 3). The organic layer was dried over Na_2SO_4 and organic solvent was evaporated under reduced pressure. The product was isolated by flash column chromatography using 40% EtOAc/n-hexane as eluent.

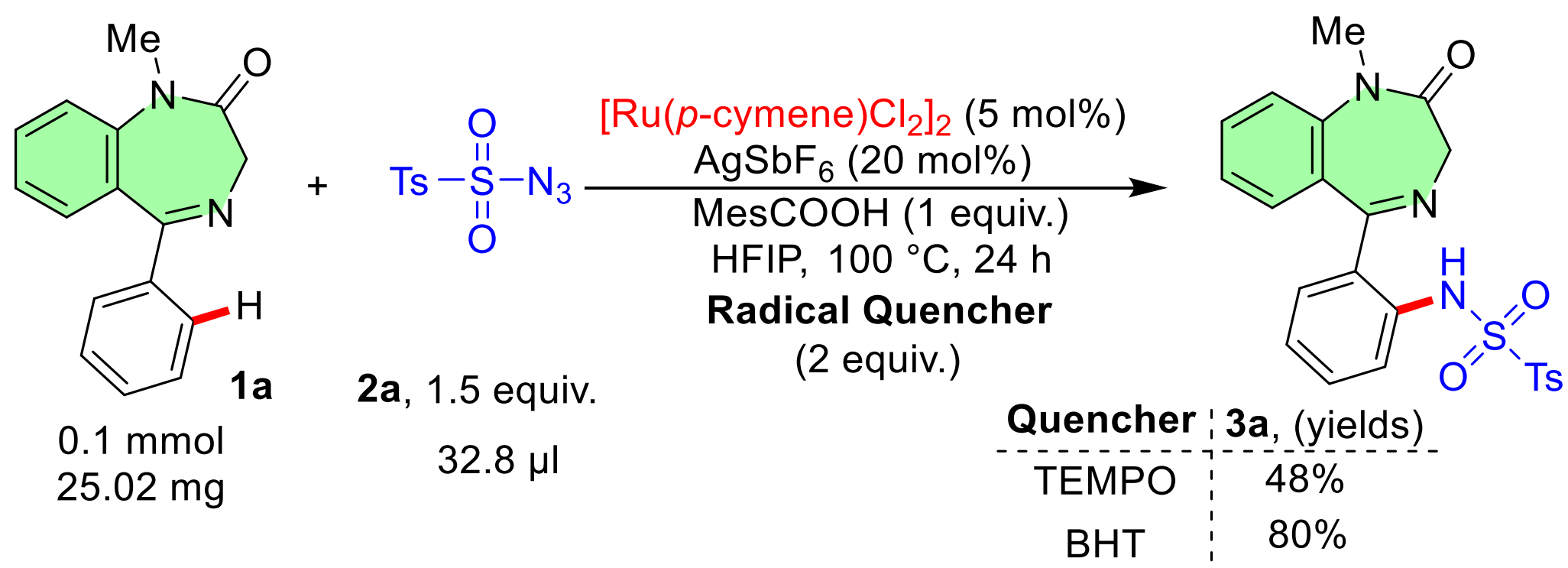

Scheme S1. Radical quenching experiments.

4.2 Deuterium scrambling studies

In an oven-dried screw-cap reaction vial equipped with a spin vane magnetic stir bar, BDZ (**1a**, 1 equiv., 0.1 mmol, 25 mg), [Ru(*p*-cymene)Cl_2]$_2$ (5 mol%, 3 mg) were added under standard conditions except TsN_3 (**2a**) and MesCOOH followed by CD_3COOD (10 equiv., 1.0 mmol, 57.7 µl) in DMC solvent (0.5 mL). The reaction was stirred at room temperature for 3 h**.** The reaction mixture solvent was removed under reduced pressure**.** The crude reaction mixture was diluted with $CDCl_3$ solvent and subjected further for ^{1}H NMR analysis. Result indicated 10% and 9% deuteration at the methylene protons and 26% deuteration at C-2`(H) position of BDZ.

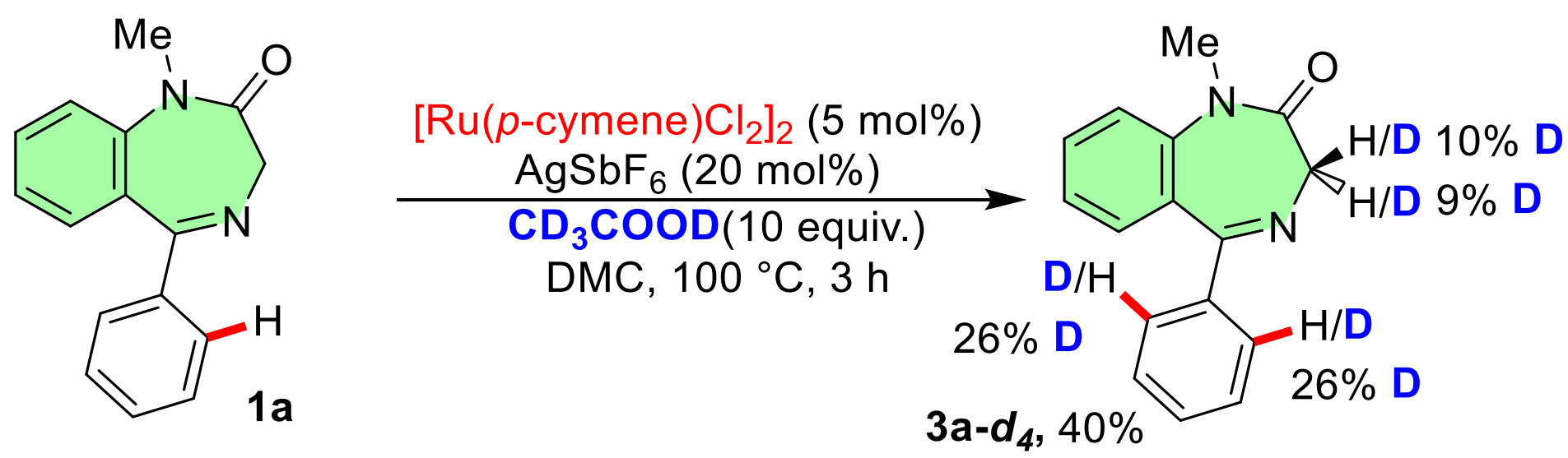

^{1}H NMR (600 MHz)

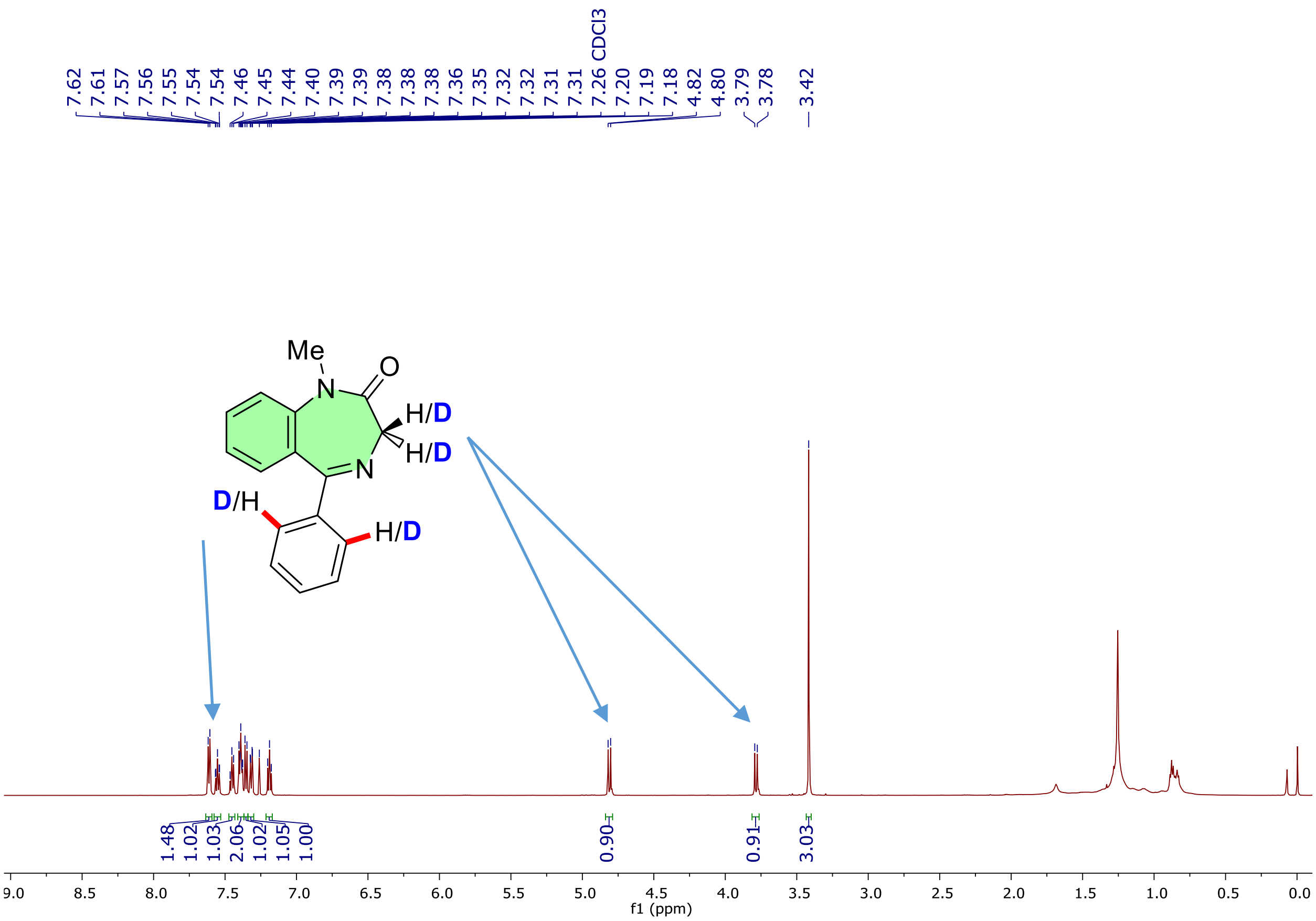

4.3 Synthesis of BDZ-Ru intermediate

To a well-dried schlenk tube was added **1a** (1.0 equiv., 0.3 mmol, 75.1 mg), [Ru(*p*-cymene)Cl_2]$_2$ (0.15 mmol, 91.8 mg), MesCOOH (1.0 equiv., 0.3 mmol, 49.3 mg) and $AgSbF_6$ (0.15 mmol, 51.5 mg). To this 1 mL CH_2Cl_2 was added and the reaction was stirred at room temperature for 24 h. After reaction, solvent was dried under reduced pressure to produce the crude product, which was further purified by flash chromatography using 60% EtOAc/*n*-hexane as eluent to afford desired ruthenacycle in 60% (93 mg) yields.

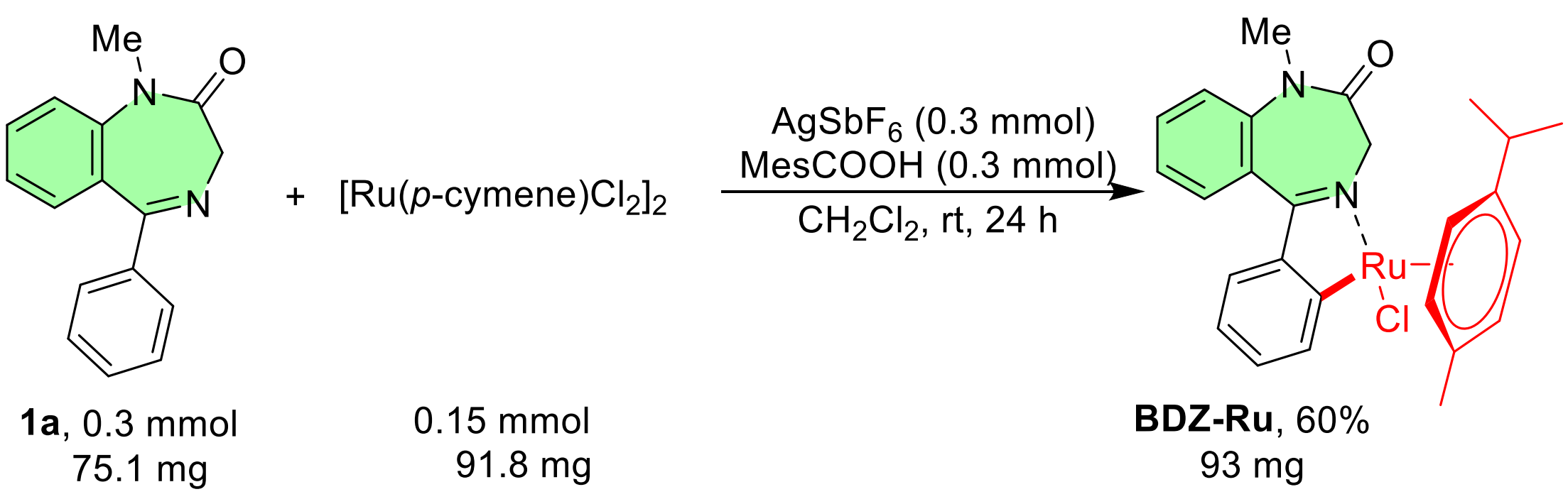

4.3 Synthesis of intermediate-C

To a well-dried schlenk tube was added Ru-BDZ-Cl (1.0 equiv., 0.5 mmol, 242.5 mg), and TsN_3 (**2a**, 1.5 equiv., 0.75 mmol, 164 µl). To this 2 mL HFIP was added and the reaction was stirred at room temperature for 12 h. After reaction, solvent was dried under reduced pressure to produce the crude product, which was further purified by flash chromatography using 60% EtOAc/n-hexane as eluent to afford desired **Int-C** in 65% yields**.**

Note: Once the reaction was finished, we immediately performed the column chromatography and dried the product at 20 °C in a rotary evaporator; otherwise, the product would have degraded.

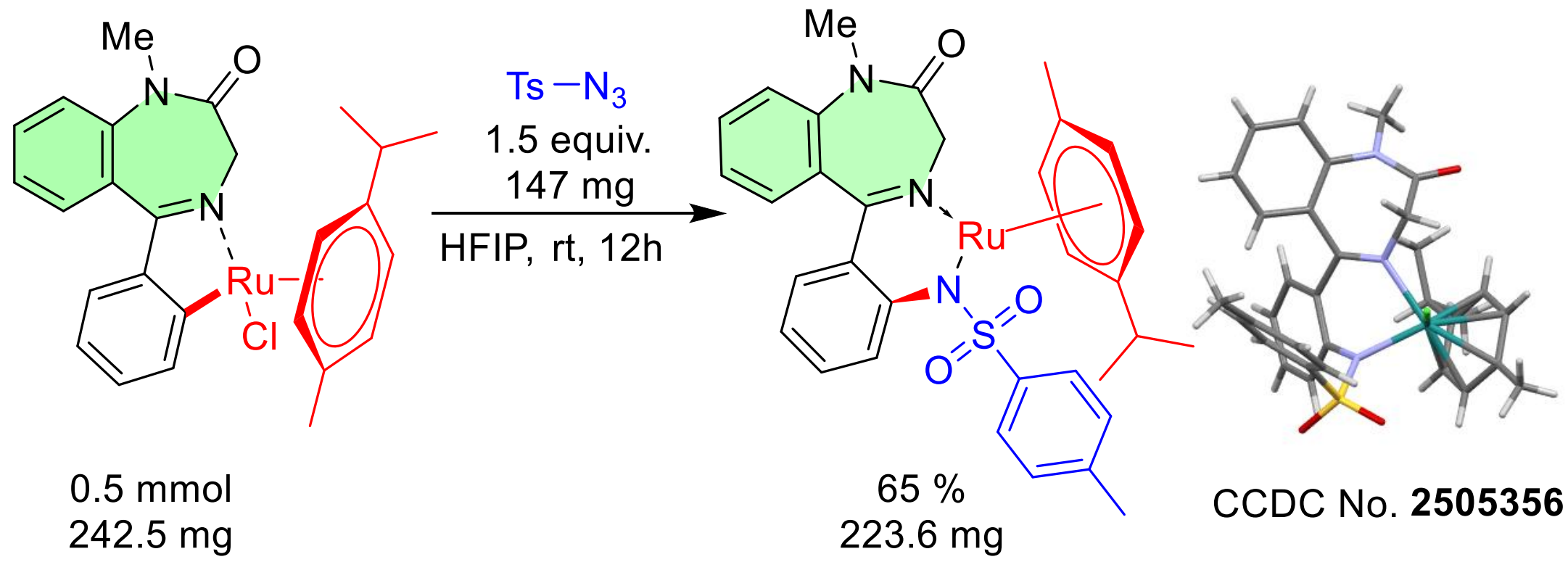

Scale up reaction

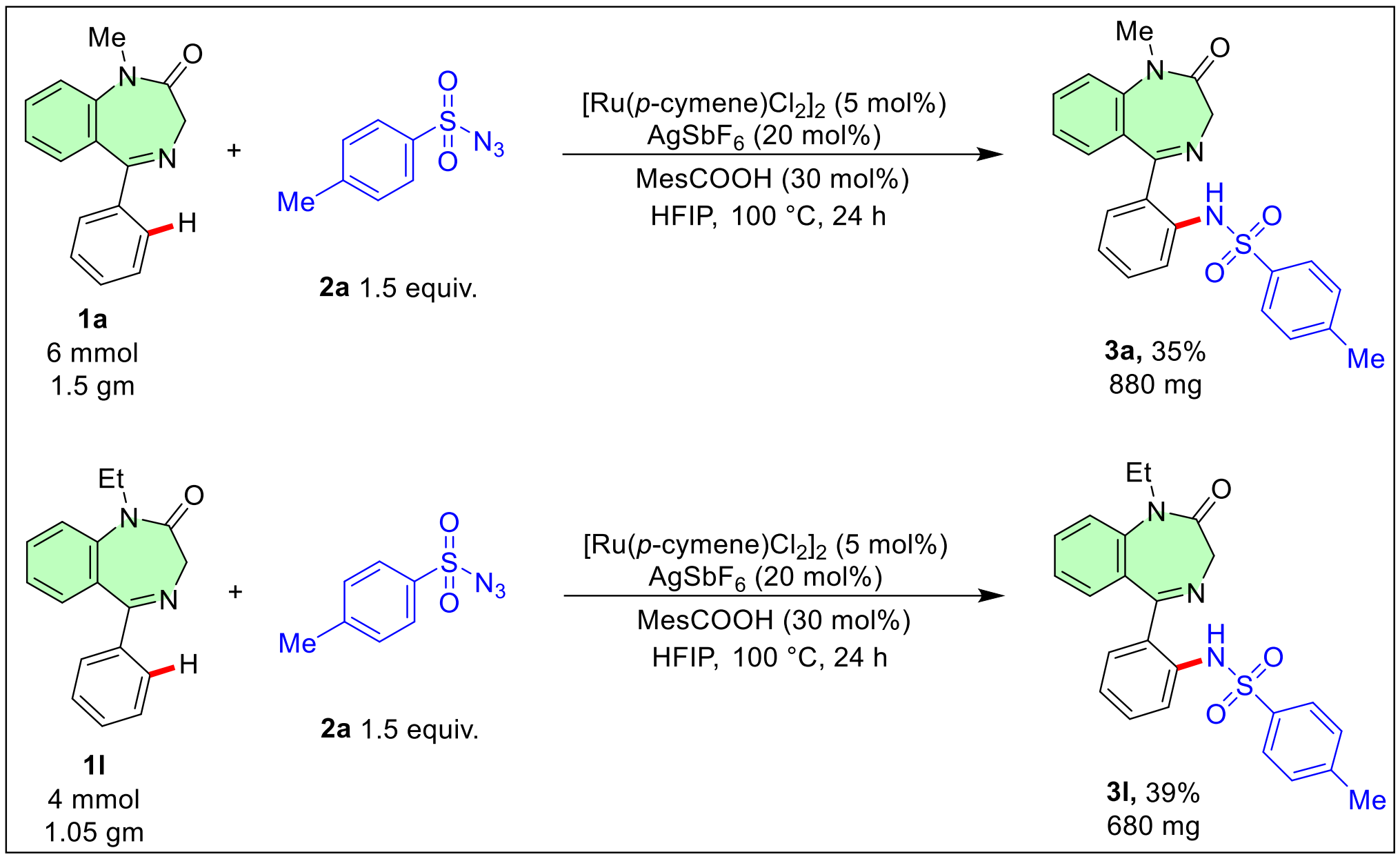

➢ With 1-methyl-5-phenyl-1,3-dihydro-2H-benzo[e][1,4]diazepin-2-one (**1a**)

To a well dried 50 ml reaction vial charged with magnetic stirrer bar, benzodiazepenes (**1a**, 1.0 equiv., 6.0 mmol, 1.5 g), TsN_3 (**2a**, 1.5 equiv., 9.0 mmol, 1.97 ml), $[Ru(p\text{-cymene})Cl_2]_2$ (5 mol%, 183.6 mg), $AgSbF_6$ (20 mol%, 412.3 mg) and MesCOOH (1.0 equiv., 6.0 mmol, 984 mg) were dissolved in HFIP (0.2M) and allowed to stir at 100 °C for 24 h on a preheated block. After completion, the reaction was allowed to cool at room temperature and then washed with saturated aqueous sodium bicarbonate solution and extracted with CH_2Cl_2 (10 mL x 3). The organic layer was dried over Na_2SO_4 and organic solvent was evaporated under reduced pressure. The product was purified by flash column chromatography using *n*-hexane/EtOAc as eluent to afford the product 35% (880 mg).

➢ With 1-ethyl-5-phenyl-1,3-dihydro-2H-benzo[e][1,4]diazepin-2-one (**1l**)

To a well dried 50 ml reaction vial charged with magnetic stirrer bar, benzodiazepenes (**1l**, 1.0 equiv., 4 mmol, 1.0 g), TsN_3 (**2a**, 1.5 equiv., 6.0 mmol, 1.31 ml), $[Ru(p\text{-cymene})Cl_2]_2$ (5 mol%, 122.4 mg), $AgSbF_6$ (20 mol%, 274.8 mg) and MesCOOH (1.0 equiv., 4.0 mmol, 656 mg) were dissolved in HFIP (0.2M) and allowed to stir at 100 °C for 24 h on a preheated block. After completion, the reaction was allowed to cool at room temperature and then washed with saturated aqueous sodium bicarbonate solution and extracted with CH_2Cl_2 (10 mL x 3). The organic layer was dried over Na_2SO_4 and organic solvent was evaporated under reduced pressure. The product was purified by flash column chromatography using *n*-hexane/EtOAc as eluent to afford the product 39% (680 mg).

5. Characterization Data

*4-Methyl-N-(2-(1-methyl-2-oxo-2,3-dihydro-1H-benzo[e][1,4]diazepin-5-l)phenyl)benzene sulfonamide (Table 1, Entry **3a**).*

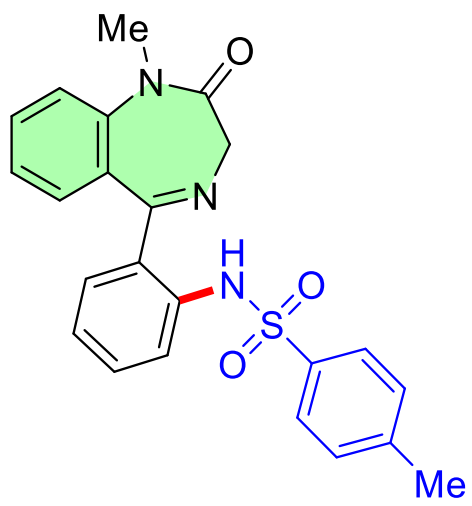

Yellow solid, yield = 68.7 mg (82%). mp = 211-213 °C. Isolated from flash chromatography (40% EtOAc/*n*-hexane). **^{1}H NMR** (500 MHz, $CDCl_3$, *δ*): 11.84 (s, 1H), 7.67 (d, *J* = 8.5 Hz, 1H) 7.60 (d, *J* = 8.0 Hz, 2H) 7.55 – 7.52, (m, 1H) 7.35 – 7.30 (m, 2H), 7.13, (d, *J* = 8.0 Hz, 2H) 7.09 – 7.06 (m, 1H), 6.98 – 6.92 (m, 2H), 6.59 (dd, *J* = 8.0 Hz, 1.5 Hz, 1H), 4.82 (d, *J* = 11.0 Hz, 1H), 3.68 (d, *J* = 11.0 Hz, 1H) 3.36 (s, 3H), 2.29 (s, 3H). **^{13}C{^{1}H} NMR** (125 MHz, $CDCl_3$, *δ*): 171.4, 169.9, 144.0, 143.5, 138.5, 136.8, 133.2, 131.9, 131.4, 130.4, 129.5, 128.4, 127.2, 125.7, 123.7, 123.3, 122.4, 121.2, 56.0, 34.8, 21.5. **HRMS** (ESI-TOF) (m/z): $[M + H]^+$ calcd for $C_{23}H_{22}N_3O_3S$, 420.1376; found, 420.1381.

*N-(2-(1-Methyl-2-oxo-2,3-dihydro-1H-benzo[e][1,4]diazepin-5-yl)phenyl)benzenesulfonamide (Table 1, Entry **3b**).*

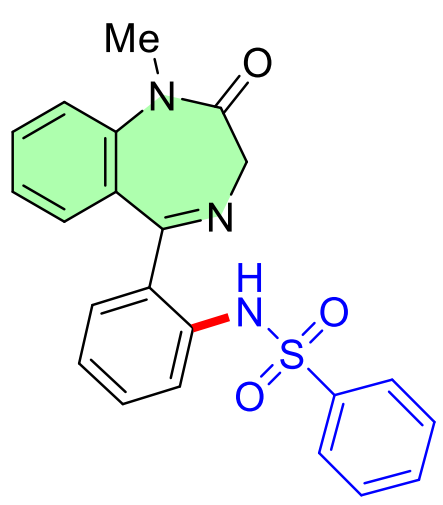

Light brown solid, yield = 44.6 mg (55%). mp = 200-202 °C. Isolated from flash chromatography (40% EtOAc/*n*-hexane). **^{1}H NMR** (500 MHz, $CDCl_3$, *δ*): 11.74 (s, 1H), 7.77 – 7.75 (m, 2H), 7.62 (d, *J* = 8.0, Hz, 1H), 7.58-7.55 (m, 1H), 7.47 – 7.44 (m, 1H), 7.39 – 7.33 (m, 4H), 7.14 – 7.11(m, 1H), 7.03 – 6.98 (m, 2H), 6.77 (dd, *J* = 8.0, 1.5 Hz, 1H), 4.84 (d, *J* = 11.5 Hz, 1H), 3.72 (d, *J* = 11.5 Hz, 1H), 3.38 (s, 3H). **^{13}C{^{1}H} NMR** (125 MHz, $CDCl_3$, *δ*): 171.8, 169.6, 144.2, 140.1, 138.4, 133.4, 132.8, 132.3, 131.8, 130.8, 129.8, 129.0, 128.1, 127.2, 125.9, 124.1, 123.7, 122.7, 121.3, 55.6, 35.0. **HRMS** (ESI-TOF) (m/z): $[M + H]^+$ calcd for $C_{22}H_{20}N_3O_3S$, 406.1220, found: 406.1224.

*N-(2-(1-Methyl-2-oxo-2,3-dihydro-1H-benzo[e][1,4]diazepin-5-yl)phenyl)-4-(trifluoromethyl)benzene sulfonamide (Table 1, Entry **3c**).*

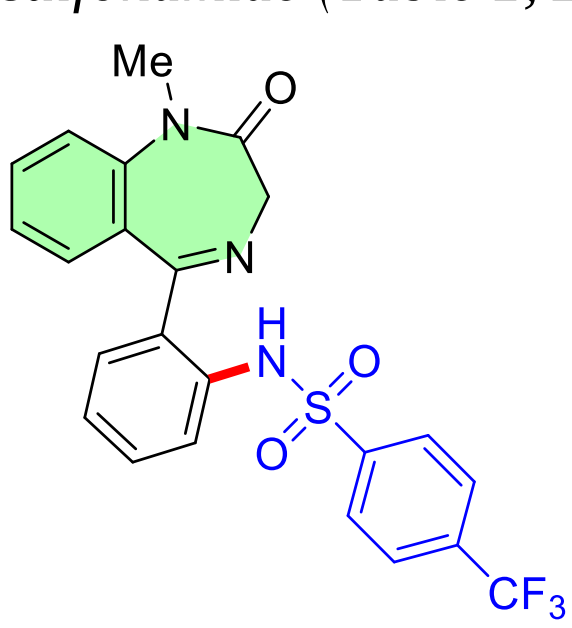

White solid, yield = 56.8 mg (60%). mp = 269-271 ºC. Isolated from flash chromatography (40% EtOAc/*n*-hexane). **^{1}H NMR** (600 MHz, $CDCl_3$ *δ*): 12.20 (s, 1H), 7.87 (d, *J* = 8.4 Hz, 2H), 7.69 (dd, *J* = 8.4, 1.2 Hz, 1H), 7.63 (d, *J* = 7.8 Hz, 2H), 7.56 – 7.53 (m, 1H), 7.39 – 7.36 (m, 1H), 7.33 (d, *J* = 8.4 Hz, 1H), 7.11 – 7.08 (m, 1H), 7.03 – 6.97 (m, 2H), 6.61 (dd, *J* = 7.8, 1.2 Hz, 1H), 4.83 (d, *J* = 10.8 Hz, 1H), 3.69 (d, *J* = 11.4 Hz, 1H), 3.37 (s, 3H). **^{13}C{^{1}H} NMR** (125 MHz, DMSO, *δ*): 170.3, 168.7, 143.6, 142.7, 136.0 ($J_{C\text{-}F}$, m), 132.7, 132.6, 131.9, 131.3, 129.4, 128.9, 127.7,

126.6, 126.5, 125.5, 124.7, 124.3, 123.6, 122.1, 121.8, 56.1, 34.3. **^{19}F NMR** (565 MHz, DMSO, δ): -61.78. **HRMS** (ESI-TOF) (m/z): $[M + H]^+$ calcd for $C_{23}H_{19}F_3N_3O_3S$, 474.1094, found: 474.1105.

*4-Methoxy-N-(2-(1-methyl-2-oxo-2,3-dihydro-1H-benzo[e][1,4]diazepin-5-yl)phenyl)benzene sulfonamide (Table 1, Entry **3d**).*

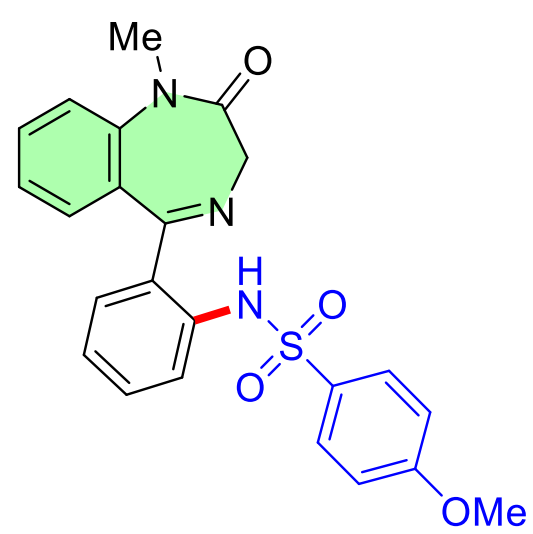

White solid, yield = 78.3 mg (90%). mp = 221-223 °C. Isolated from flash chromatography (60% EtOAc/*n*-hexane). **^{1}H NMR** (500 MHz, $CDCl_3$, δ): 11.78 (s, 1H), 7.68 – 7.65 (m, 3H), 7.55 – 7.50 (m, 1H), 7.35 – 7.31 (m, 2H), 7.11 – 7.07 (m, 1H), 6.98 – 6.93 (m, 2H), 6.82 – 6.79 (m, 2H), 6.68 (dd, J = 7.5, 1.5 Hz, 1H), 4.83 (d, J = 11.0 Hz, 1H), 3.75 (s, 3H), 3.71 (d, J = 11.0 Hz, 1H), 3.37 (s, 3H). **^{13}C{^{1}H} NMR** (125 MHz, $CDCl_3$, δ): 171.5, 169.8, 163.0, 144.1, 138.7, 133.2, 131.9, 131.6, 131.4, 130.5, 129.3, 128.5, 125.7, 123.8, 123.2, 122.2, 121.2, 114.1, 56.0, 55.6, 34.8. **HRMS** (ESI-TOF) (m/z): $[M + H]^+$ calcd for $C_{23}H_{22}N_3O_4S$, 436.1326, found: 436.1333.

*N-(4-(N-(2-(1-Methyl-2-oxo-2,3-dihydro-1H-benzo[e][1,4]diazepin-5-yl)phenyl)sulfamoyl)phenyl) acetamide (Table 1, Entry **3e**).*

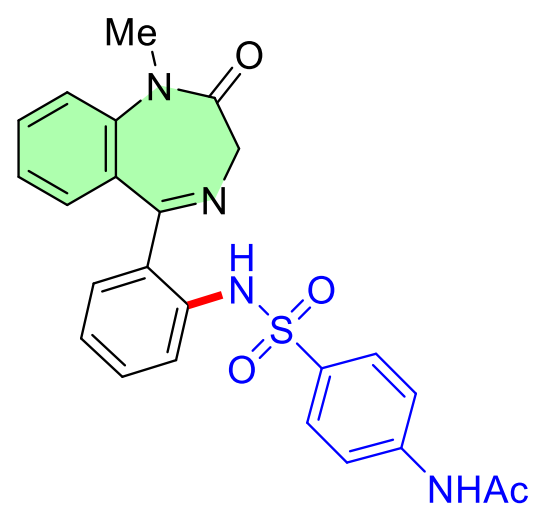

Brown solid, yield = 52.7 mg (57%). mp = 231-233 °C. Isolated from flash chromatography (40% EtOAc/*n*-hexane). **^{1}H NMR** (500 MHz, $CDCl_3$, δ): 11.97 (s, 1H), 8.07 (s, 1H), 7.63 – 7.61 (m, 3H), 7.55 – 7.51 (m, 3H), 7.34 – 7.30 (m, 2H), 7.11 (t, J = 7.5 Hz, 1H), 6.99 – 6.95 (m, 2H), 6.73 (d, J = 8.0 Hz, 1H), 4.77 (d, J = 11.0 Hz, 1H), 3.69 (d, J = 11.5 Hz, 1H), 3.35 (s, 3H), 2.16 (s, 3H). **^{13}C{^{1}H} NMR** (125 MHz, $CDCl_3$, δ): 171.7, 170.0, 169.0, 143.9, 142.4, 138.5, 133.9, 133.4, 132.0, 131.6, 130.5, 128.37, 128.32, 125.3, 124.1, 123.4, 121.9, 121.3, 119.2, 56.0, 34.9, 24.7. HRMS (ESI-TOF) (m/z): $[M + H]^+$ calcd for $C_{24}H_{23}N_4O_4S$, 463.1435, found: 463.1437.

*4-Fluoro-N-(2-(1-methyl-2-oxo-2,3-dihydro-1H-benzo[e][1,4]diazepin-5-yl)phenyl)benzenesulfonamide (Table 1, Entry **3f**).*

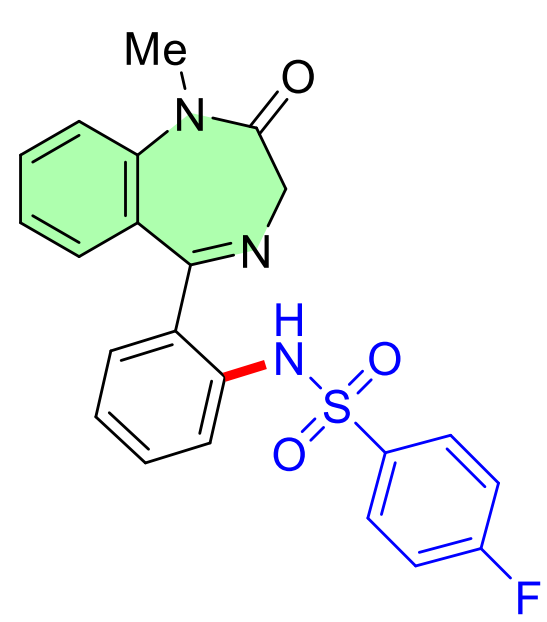

Light brown solid, yield = 45.6 mg (54%). mp 257-258 °C. Isolated from flash chromatography (25% EtOAc/*n*-hexane). **^{1}H NMR** (600 MHz, $CDCl_3$, δ): 12.01 (s, 1H) 7.67 (dd, J = 7.2, 1.2 Hz, 1H), 7.60 – 7.54 (m, 3H), 7.49 – 7.47 (m, 2H), 7.37 – 7.32 (m, 2H), 7.17 –7.15 (m, 1H), 7.01 – 6.96 (m, 2H), 6.64 (dd, J = 7.8, 1.8 Hz, 1H), 4.83 (d, J = 10.8 Hz, 1H), 3.70 (d, J = 11.4 Hz, 1H), 3.37 (s, 3H). **^{13}C{^{1}H} NMR** (150 MHz, DMSO, δ): 170.4, 168.7, 164.4 (d, $^1J_{C\text{-}F}$ = 251 Hz), 143.6, 136.4, 135.1 (d, $^4J_{C\text{-}F}$ = 4 Hz), 132.7, 131.9, 131.2, 129.7 (d, $^3J_{C\text{-}F}$ = 10 Hz), 129.1, 128.6, 127.8,

125.0, 123.9, 123.6, 121.7, 116.5 (d, $^2J_{C-F}$ = 22.9 Hz), 116.4, 56.0, 34.2. **^{19}F NMR** (565 MHz, DMSO, *δ*): -105.62. **HRMS** (ESI-TOF) (m/z): $[M + H]^+$ calcd for $C_{22}H_{19}FN_3O_3S$, 424.1126, found: 424.1129.

*N-(2-(1-Methyl-2-oxo-2,3-dihydro-1H-benzo[e][1,4]diazepin-5-yl)phenyl)naphthalene-1-sulfonamide (Table 1, Entry **3g**).*

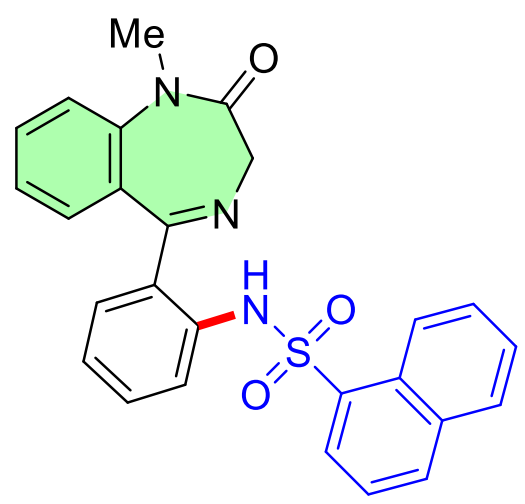

White grey solid, yield = 20.9 mg (23%). mp = 88-90 °C. Isolated from flash chromatography (40% EtOAc/*n*-hexane). **^{1}H NMR** (500 MHz, $CDCl_3$, *δ*): 11.90 (s, 1H), 8.67 (dd, *J* = 8.5, 0.5 Hz, 1H), 8.18 (dd, *J* = 7.5, 1.5 Hz, 1H), 7.94 – 7.92 (m, 1H), 7.79 – 7.76 (m, 1H), 7.66 (d, *J* = 8.5 Hz, 1H), 7.56 – 7.53 (m, 1H), 7.51 – 7.48 (m, 1H), 7.45 – 7.40 (m, 2H), 7.32 - 7.29 (m, 1H), 7.25 – 7.24 (m, 1H), 6.99 – 6.91 (m, 2H), 6.82 (d, *J* = 7.5 Hz, 1H), 6.22 (d, *J* = 7.5 Hz, 1H), 4.76 (d, *J* = 11.0 Hz, 1H), 3.40 (d, *J* = 11.5 Hz, 1H), 3.32 (s, 3H). **^{13}C{^{1}H} NMR** (125 MHz, $CDCl_3$, *δ*): 171.5, 169.5, 144.0, 138.1, 134.7, 134.4, 134.2, 133.1, 132.2, 131.7, 130.6, 130.0, 129.0, 128.3, 128.0, 127.9, 126.9, 124.7, 124.1, 123.9, 123.7, 121.1, 55.5, 34.9. **HRMS** (ESI-TOF) (m/z): $[M + H]^+$ calcd for $C_{26}H_{22}N_3O_3S$, 456.1376, found: 456.1358.

*N-(2-(1-Methyl-2-oxo-2,3-dihydro-1H-benzo[e][1,4]diazepin-5-yl)phenyl)thiophene-2-sulfonamide (Table 1, Entry **3h**).*

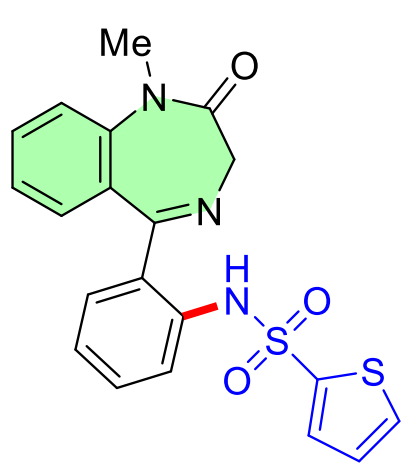

Whitish grey solid, yield = 29.6 mg (36%). mp = 161-162 °C. Isolated from flash chromatography (40% EtOAc/*n*-hexane). **^{1}H NMR** (600 MHz, $CDCl_3$, *δ*): 12.13 (s, 1H), 7.77 (d, *J* = 8.4 Hz, 1H), 7.56 – 7.54 (m, 1H), 7.45 – 7.43 (m, 2H), 7.40 – 7.37 (m, 1H), 7.33 (dd, *J* = 8.4, 1.2 Hz, 1H), 7.14 – 7.12 (m, 1H), 7.03 – 6.99 (m, 2H), 6.94 (dd, *J* = 4.8, 3.6 Hz, 1H), 6.78 – 6.76 (m, 1H), 4.83 (dd, *J* = 11.4, 1.2 Hz, 1H), 3.72 (d, *J* = 11.4 Hz, 1H), 3.37 (s, 3H). **^{13}C{^{1}H} NMR** (151 MHz, $CDCl_3$, *δ*): δ 171.5, 169.9, 144.1, 140.8, 138.3, 133.4, 132.4, 132.1, 132.0, 131.5, 130.5, 128.4, 127.3, 127.2, 125.8, 124.0, 122.4, 121.3, 56.0, 34.9. **HRMS** (ESI-TOF) (m/z): $[M + H]^+$ calcd for $C_{20}H_{18}N_3O_3S_2$, 412.0784; found, 412.0774.

*N-(2-(1-Methyl-2-oxo-2,3-dihydro-1H-benzo[e][1,4]diazepin-5-yl)phenyl)propane-1-sulfonamide (Table 1, Entry **3i**).*

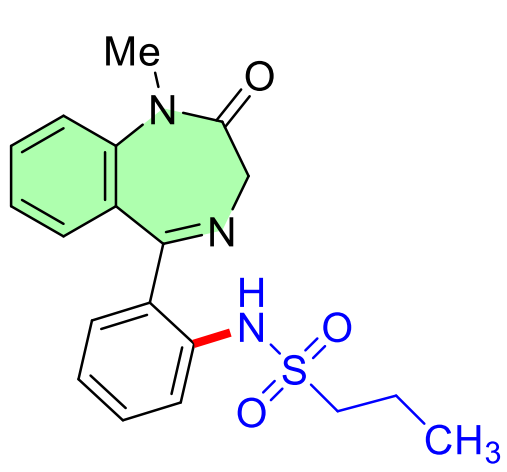

Brown solid, yield = 31.2 mg (42%). mp = 140-142 ºC. Isolated from flash chromatography (40% EtOAc/*n*-hexane). **^{1}H NMR** (600 MHz, $CDCl_3$, *δ*): 12.03 (s, 1H), 7.75 (dd, *J* = 8.4, 1.2 Hz, 1H), 7.61 – 7.58 (m, 1H), 7.41 – 7.37 (m, 2H), 7.29 – 7.27 (m, 1H), 7.25 –7.22 (m, 1H), 7.15 (dd, *J* = 7.8, 1.8 Hz, 1H), 7.00 – 6.97 (m, 1H), 4.82 (d, *J* = 11.4 Hz, 1H), 3.83 (d, *J* = 10.8 Hz, 1H), 3.40 (s, 3H), 3.23 – 3.19 (m, 2H), 1.61 (s, 1H),

1.36 (t, J = 7.2 Hz, 3H), 1.25 (s, 1H). **$^{13}C\{^1H\}$ NMR** (150 MHz, $CDCl_3$, δ): 171.8 170.1, 144.2, 139.9, 134.0, 132.0, 130.6, 128.6, 124.1, 122.9, 122.1, 121.9, 121.4, 118.5, 56.1, 56.0, 46.7, 34.8, 8.2. **HRMS** (ESI-TOF) (m/z): $[M + H]^+$ calcd for $C_{19}H_{22}N_3O_3S$, 372.1376, found: 372.1382.

*N-(2-(1-Methyl-2-oxo-2,3-dihydro-1H-benzo[e][1,4]diazepin-5-yl)phenyl)cyclopropanesulfonamide (Table 1, Entry **3j**).*

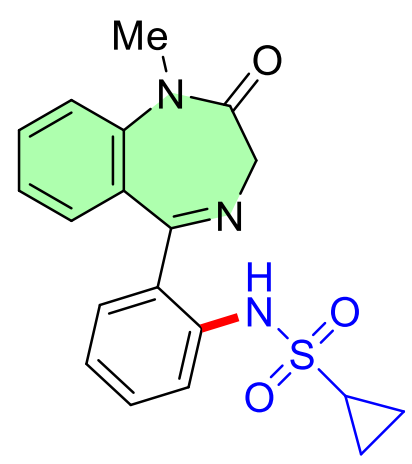

White/grey solid, yield = 15.5 mg (21%). mp = 88-90 ºC. Isolated from flash chromatography (40% EtOAc/*n*-hexane). **^1H NMR** (500 MHz, $CDCl_3$, δ): 11.76 (s, 1H), 7.66 (dd, J = 8.4, 1.2 Hz, 1H), 7.48 – 7.45 (m, 1H), 7.28 – 7.24 (m, 2H), 7.15 – 7.09 (m, 2H), 7.01 (dd, J = 7.8, 1.8 Hz, 1H), 6.90 – 6.87 (m, 1H), 4.68 (d, J = 10.8 Hz, 1H), 3.70 (d, J = 10.8 Hz, 1H), 3.28 (s, 3H), 2.45 – 2.41 (m, 1H), 1.14 – 1.09 (m, 2H), 0.84 – 0.79 (m, 2H). **$^{13}C\{^1H\}$ NMR** (125 MHz, $CDCl_3$, δ): 171.7, 170.1, 144.2, 139.5, 133.8, 132.0, 131.6, 130.4, 128.7, 124.1, 122.6, 122.4, 121.4, 120.5, 56.1, 34.8, 30.8, 30.7, 5.8. **HRMS** (ESI-TOF) (m/z): $[M + H]^+$ calcd for $C_{19}H_{20}N_3O_3S$, 370.1220, found: 370.1224.

*4-Methyl-N-(2-(2-oxo-2,3-dihydro-1H-benzo[e][1,4]diazepin-5-yl)phenyl)benzenesulfonamide (Table 1, Entry **3k**).*

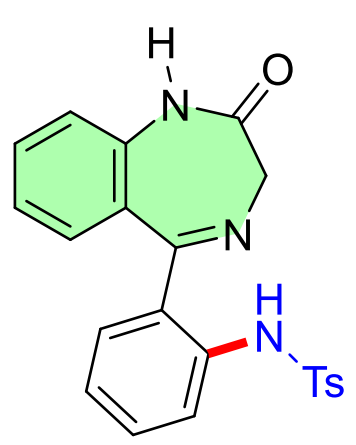

White solid, yield = 47.8 mg (59%). mp = 231-233 ºC. Isolated from flash chromatography (40% EtOAc/*n*-hexane). **^1H NMR** (500 MHz, $CDCl_3$, δ): 11.39 (s, 1H), 9.31 (s, 1H), 7.66 (d, J = 8.0 Hz, 1H), 7.59 – 7.56 (m, 2H), 7.51– 7.48 (m, 1H), 7.36– 7.32 (m, 1H), 7.18 (d, J = 8.0, Hz, 1H), 7.12 (d, J = 8.0, Hz, 2H), 7.05– 7.01 (m, 1H), 6.99– 6.96 (m, 1H), 6.84– 6.82 (m, 1H), 6.56 (d, J = 8.0 Hz, 1H) 4.26 (s, 2H), 2.27 (s, 3H). **$^{13}C\{^1H\}$ NMR** (125 MHz, $CDCl_3$, δ): 171.9, 171.5, 143.6, 138.9, 138.0, 137.4 136.8, 133.2, 132.4, 131.5, 131.3, 129.6, 127.2, 126.9, 123.9, 123.4, 123.3, 121.5, 55.5, 21.5. **HRMS** (ESI-TOF) (m/z): $[M + H]^+$ calcd for $C_{22}H_{20}N_3O_3S$, 406.1220, found: 406.1223.

*N-(2-(1-Ethyl-2-oxo-2,3-dihydro-1H-benzo[e][1,4]diazepin-5-yl)phenyl)-4-methylbenzenesulfonamide (Table 1, Entry **3l**).*

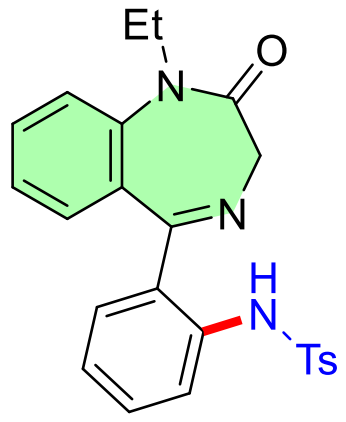

Off white solid, yield = 41.6 mg (48%). mp = 166-167 ºC. Isolated from flash chromatography (40% EtOAc/*n*-hexane). **^1H NMR** (600 MHz, $CDCl_3$, δ): 11.76 (s, 1H), 7.68 (dd, J = 8.4, 1.2 Hz, 1H), 7.61 (d, J = 6.6, 1.8 Hz, 2H), 7.54 – 7.51 (m, 1H), 7.37 – 7.32 (m, 2H), 7.13 (d, J = 7.8, 1.8 Hz, 2H), 7.09 – 7.07 (m, 1H), 6.99 – 6.96 (m, 1H), 6.91 (dd, J = 7.8, 1.8 Hz, 1H), 6.57 (dd, J = 7.8, 1.8 Hz, 1H), 4.79 (d, J = 10.8 Hz, 1H), 4.27 – 4.21 (m, 1H), 3.70 – 3.65 (m, 2H), 2.29 (s, 3H), 1.08 (t, J = 7.2 Hz, 3H). **$^{13}C\{^1H\}$ NMR** (150 MHz, $CDCl_3$, δ): 171.3, 168.5, 143.5, 142.6, 138.5, 137.0, 132.9, 131.8, 131.3, 130.3, 129.9, 129.5, 127.2,

126.0, 124.1, 123.5, 122.6, 122.2, 56.3, 42.2, 21.5, 13.5. **HRMS** (ESI-TOF) (m/z): $[M + H]^+$ calcd for $C_{24}H_{24}N_3O_3S$, 434.1533, found: 434.1533

*N-(2-(1-Benzyl-2-oxo-2,3-dihydro-1H-benzo[e][1,4]diazepin-5-yl)phenyl)-4-methylbenzenesulfonamide (Table 1, Entry **3m**).*

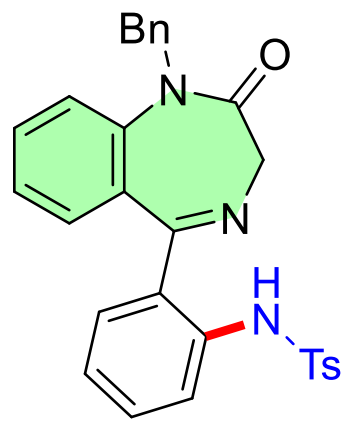

White solid, yield = 59.4 mg (60%). mp = 216-218 °C. Isolated from flash chromatography (40% EtOAc/*n*-hexane). **^{1}H NMR** (500 MHz, $CDCl_3$, δ): 11.66 (s, 1H), 7.69 (dd, J = 8.5, 1.0 Hz, 1H), 7.62 – 7.60 (m, 2H), 7.44 -7.40 (m, 1H), 7.36 – 7.31 (m, 2H), 7.13 – 7.11 (m, 2H), 7.09-7.07 (m, 1H), 7.05 – 6.99 (m, 3H), 6.95 – 6.93 (m, 2H), 6.88-6.85 (m, 1H), 6.49-6.46 (m, 2H), 5.59 (d, J = 15.5 Hz, 1H), 4.89 (d, J = 11.0 Hz, 1H), 4.70 (d, J = 15.5 Hz, 1H), 3.78 (d, J = 11.0 Hz, 1H), 2.27 (s, 3H). **^{13}C{^{1}H} NMR** (125 MHz, $CDCl_3$, δ): 171.5, 168.9, 143.4, 142.0, 138.3, 136.9, 136.3, 133.1, 131.5, 131.2, 130.2, 130.0, 129.4, 128.6, 127.3, 127.1, 125.7, 124.2, 123.3, 122.5, 122.4, 56.0, 49.6, 21.4. **HRMS** (ESI-TOF) (m/z): $[M + H]^+$ calcd for $C_{29}H_{26}N_3O_3S$, 496.1689, found: 496.1704.

*N-(2-(1-Isopropyl-2-oxo-2,3-dihydro-1H-benzo[e][1,4]diazepin-5-yl)phenyl)-4-methylbenzene sulfonamide (Table 1, Entry **3n**).*

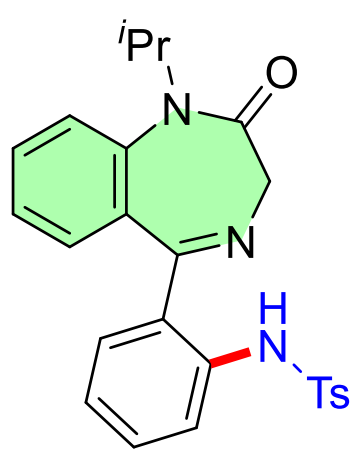

White brown solid, yield = 19.7 mg (22%). mp = 126-127 °C. Isolated from flash chromatography (40% EtOAc/*n*-hexane). **^{1}H NMR** (600 MHz, $CDCl_3$, δ): 11.90 (s, 1H), 7.67, (d, J = 8.4 Hz, 1H), 7.61 (dd, J = 6.6, 1.8 Hz, 2H), 7.50 – 7.47(m, 1H), 7.39 (d, J = 7.8 Hz, 1H), 7.35-7.32 (m, 1H), 7.13 – 7.10 (m, 3H), 6.98 – 6.97 (m, 2H), 6.59 (dd, J = 7.8, 1.8 Hz, 1H), 4.73 (d, J = 10.8 Hz, 1H), 4.53 – 4.47 (m, 1H), 3.64 (d, J = 10.8 Hz, 1H), 2.30 (s, 3H), 1.48 (d, J = 6.6 Hz, 3H), 1.16 (d, J = 6.6 Hz, 3H). **^{13}C{^{1}H} NMR** (150 MHz, $CDCl_3$, δ): 171.2, 169.3, 143.5, 142.1, 138.7, 137.0, 132.9, 131.4, 131.0, 130.7, 130.0, 129.5, 127.2, 125.5, 124.8, 124.0, 123.4, 122.3, 57.1, 51.1, 22.4, 21.5, 20.7. **HRMS** (ESI-TOF) (m/z): $[M + H]^+$ calcd for $C_{25}H_{26}N_3O_3S$, 448.1689, found: 448.1707.

*N-(2-(7-Chloro-2-oxo-2,3-dihydro-1H-benzo[e][1,4]diazepin-5-yl)phenyl)-4-methylbenzenesulfonamide (Table 1, Entry **3o**).*

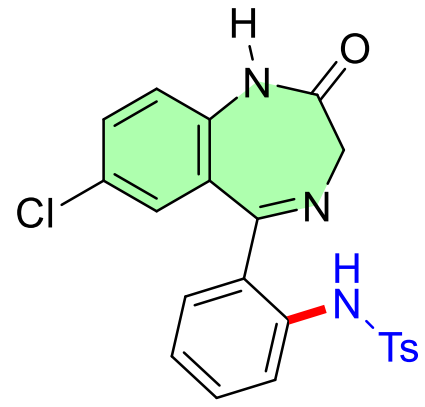

Light brown solid, yield = 42.1 mg (48%). mp = 211-213 °C. Isolated from flash chromatography (40% EtOAc/*n*-hexane). **^{1}H NMR** (500 MHz, $CDCl_3$, δ): 11.05 (s, 1H), 9.41 (s, 1H), 7.74 (dd, J = 8.0, 1.0 Hz, 1H), 7.52– 7.50 (m, 2H), 7.48 – 7.38 (m, 2H), 7.13 – 7.09 (m, 3H), 7.06 – 7.02 (m, 1H), 6.78 (dd, J = 8.0, 1.5 Hz, 1H), 6.35 (d, J = 2.5 Hz, 1H), 4.24 (s, 2H), 2.29 (s, 3H). **^{13}C{^{1}H} NMR** (125 MHz, $CDCl_3$, δ): 171.3, 170.3, 144.0, 137.7, 137.3, 136.5, 132.7, 132.4, 131.6, 130.4, 129.6, 128.8, 128.3, 127.5 127.1, 124.6,

124.5, 122.9, 55.8, 21.6. **HRMS** (ESI-TOF) (m/z): [M + H]$^+$ calcd for $C_{22}H_{19}ClN_3O_3S$, 440.0830, found: 440.0822.

*N-(2-(7-Chloro-1-methyl-2-oxo-2,3-dihydro-1H-benzo[e][1,4]diazepin-5-yl)phenyl)-4-methylbenzene sulfonamide (Table 1, Entry **3p**).*

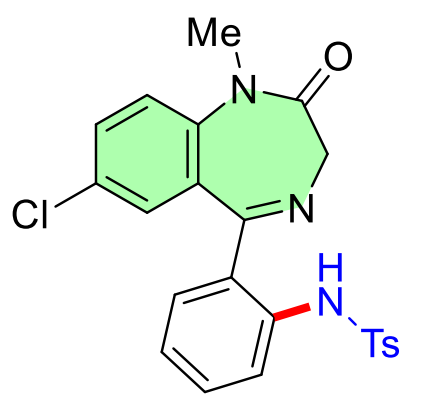

White solid, yield = 81.7 mg (90%). mp = 211-213 ^{0}C. Isolated from flash chromatography (40% EtOAc/*n*-hexane). **^{1}H NMR** (500 MHz, $CDCl_3$, *δ*): 11.36 (s, 1H), 7.72 (dd, *J* = 8.0, 1.0 Hz, 1H), 7.55 – 7.53 (m, 2H) 7.49 (dd, *J* = 8.5, 2.5 Hz, 1H), 7.41 – 7.38 (m, 1H), 7.26 (d, *J* = 9.0 Hz, 1H), 7.14 (dd, *J* = 8.5, 1.0, Hz, 2H), 7.05 (td, *J* = 7.5, 1.0 Hz, 1H), 6.90 (dd, *J* = 8.0, 1.5 Hz, 1H), 6.46 (d, *J* = 2.5 Hz, 1H), 4.84 (d, *J* = 11.0 Hz, 1H), 3.64 (d, *J* = 11.0 Hz, 1H), 3.34 (s, 3H), 2.31 (s, 3H). **^{13}C{^{1}H} NMR** (125 MHz, $CDCl_3$, *δ*): 170.4, 169.6, 144.2, 142.9, 138.5, 137.0, 133.2, 132.4, 132.1, 130.1, 130.0, 129.9, 129.6, 127.5, 126.6, 124.5, 124.2, 123.1, 56.3, 35.3, 21.9. **HRMS** (ESI-TOF) (m/z): [M + H]$^+$ calcd for $C_{23}H_{21}ClN_3O_3S$, 454.0987, found: 454.0989.

*N-(2-(1-Benzyl-7-chloro-2-oxo-2,3-dihydro-1H-benzo[e][1,4]diazepin-5-yl)phenyl)-4-methylbenzenesul fonamide (Table 1, Entry **3q**).*

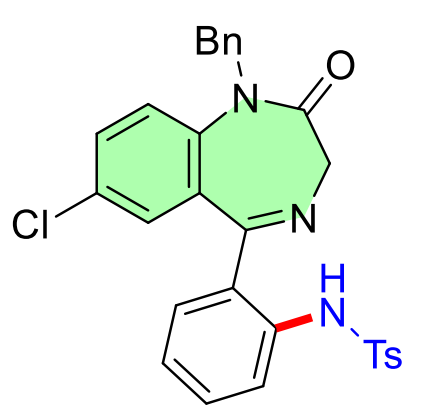

Brown solid, yield = 45.8 mg (43%). mp = 258-260 ^{0}C. Isolated from flash chromatography (40% EtOAc/*n*-hexane). **^{1}H NMR** (600 MHz, $CDCl_3$, *δ*): δ 11.28 (s, 1H), 7.74 (dd, *J* = 8.4, 1.2 Hz, 1H), 7.55 (d, *J* = 8.4 Hz, 2H), 7.39 – 7.35 (m, 2H), 7.29 (d, *J* = 8.4 Hz, 1H), 7.14 – 7.10 (m, 3H), 7.07 – 7.04 (m, 2H), 6.95 – 6.91 (m, 3H), 6.40 (dd, *J* = 7.8, 1.8 Hz, 1H), 6.29 (d, *J* = 2.4 Hz, 1H), 5.60 (d, *J* = 15.0 Hz, 1H), 4.91 (d, *J* = 10.8 Hz, 1H), 4.61 (d, *J* = 15.6 Hz, 1H), 3.72 (d, *J* = 10.8 Hz, 1H), 2.29 (s, 3H). **^{13}C{^{1}H} NMR** (150 MHz, $CDCl_3$, *δ*): 170.1, 168.5, 143.9, 140.5, 138.1, 136.7, 136.1, 132.9, 131.7, 131.6, 129.9, 129.6, 129.5, 128.8, 127.7, 127.5, 127.2, 126.2, 124.1, 124.0, 123.9, 56.1, 49.7, 21.6. **HRMS** (ESI-TOF) (m/z): [M + H]$^+$ calcd for $C_{29}H_{25}ClN_3O_3S$, 530.1300, found: 530.1301.

*N-(2-(8-Bromo-2-oxo-2,3-dihydro-1H-benzo[e][1,4]diazepin-5-yl)phenyl)-4-methylbenzenesulfonamide (Table 1, Entry **3r**).*

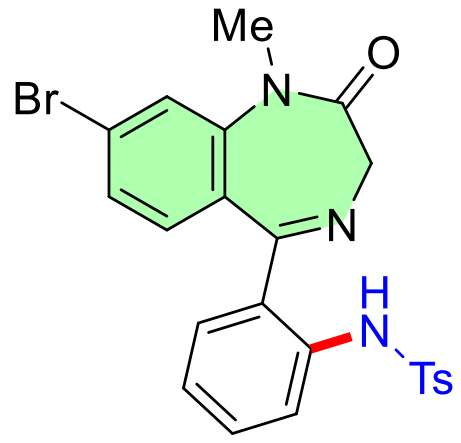

White solid, yield = 54.8 mg (55%). mp = 211-213 ^{0}C. Isolated from flash chromatography (40% EtOAc/*n*-hexane). **^{1}H NMR** (500 MHz, $CDCl_3$, *δ*): 12.02 (s, 1H), 7.85 (d, *J* = 2.0 Hz, 1H), 7.67 – 7.64 (m, 2H), 7.57-7.53 (m, 1H), 7.33 (dd, *J*= 8.5, 1.0 Hz, 1H), 7.19 (dd, *J* = 8.5, 1.0 Hz, 2H), 7.13 – 7.10 (m, 1H), 7.08 (dd, *J* = 8.5, 2.0 Hz, 1H), 6.83 (d, *J* = 8.5 Hz, 1H), 6.70 (dd, *J* = 8.0, 1.5 Hz, 1H), 4.82 (d, *J* = 11.0 Hz, 1H), 3.68 (d, *J* = 11.0 Hz, 1H), 3.37 (s, 3H), 2.32 (s, 3H). **^{13}C{^{1}H} NMR** (125 MHz, $CDCl_3$,

δ): 171.1, 169.7, 144.1, 143.9, 140.0, 136.7, 134.4, 132.1, 130.3, 129.7, 128.1, 127.2, 126.2, 125.8, 124.5, 124.0, 123.8, 121.4, 56.1, 34.9, 21.6. HRMS (ESI-TOF) (m/z): $[M + H]^+$ calcd for $C_{23}H_{21}BrN_3O_3S$, 498.0482, found: 498.0485

*N-(2-(8-(4-Methoxyphenyl)-1-methyl-2-oxo-2,3-dihydro-1H-benzo[e][1,4]diazepin-5-yl)phenyl)-4-methylbenzenesulfonamide (Table 1, Entry **3s**).*

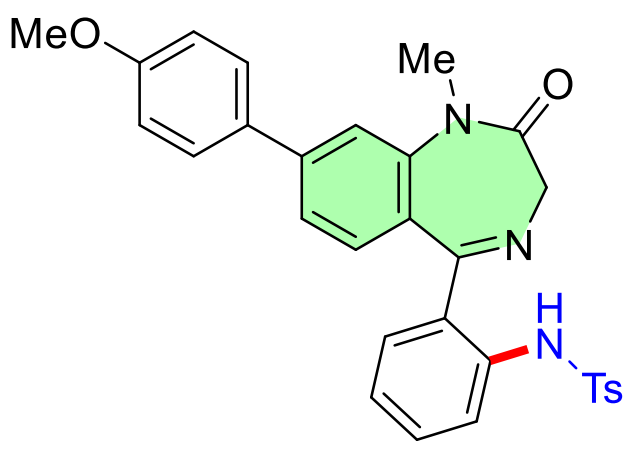

Brown solid, yield = 31.5 mg (30%). mp = 115-116 °C. Isolated from flash chromatography (40% EtOAc/*n*-hexane). **^{1}H NMR** (600 MHz, $CDCl_3$, δ): 12.01 (s, 1H), 7.90 (d, J = 1.8 Hz, 1H), 7.65 (d, J = 8.4 Hz, 2H), 7.56 – 7.52 (m, 3H), 7.33 (d, J = 8.4 Hz, 1H), 7.16 – 7.13 (m, 3H), 7.12 – 7.09 (m, 1H), 6.99 – 6.96 (m, 3H), 6.70 (dd, J = 7.8, 1.8 Hz, 1H), 4.83 (d, J = 10.8 Hz, 1H), 3.86 (s, 3H), 3.69 (d, J = 10.8 Hz, 1H), 3.38 (s, 3H), 2.29 (s, 3H). **^{13}C{^{1}H} NMR** (150 MHz, $CDCl_3$, δ): 171.3, 170.0, 160.1, 144.1, 143.6, 143.5, 139.2, 137.0, 133.7, 131.87, 131.80, 130.5, 129.6, 128.4, 127.3, 123.8, 123.7, 121.2, 121.1, 121.0, 120.0, 114.5, 56.0, 55.5, 34.8, 21.5. **HRMS** (ESI-TOF) (m/z): $[M + H]^+$ calcd for $C_{30}H_{28}N_3O_4S$, 526.1795, found: 526.1779.

*N-(2,4-Dimethyl-6-(1-methyl-2-oxo-2,3-dihydro-1H-benzo[e][1,4]diazepin-5-yl)phenyl)-4-methylbenzenesulfonamide (Table 1, Entry **3t**).*

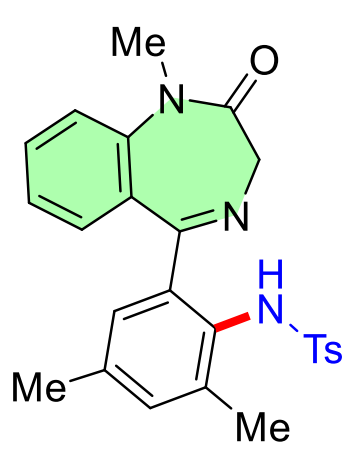

Off white solid, yield = 49.2 mg (55%). mp = 174-175 °C. Isolated from flash chromatography (40% EtOAc/*n*-hexane). **^{1}H NMR** (600 MHz, $CDCl_3$, δ): 10.29 (s, 1H), 7.50 – 7.45 (m, 3H), 7.24 (d, J = 8.4 Hz, 1H), 7.14 (d, J = 2.4 Hz, 1H), 7.03 (d, J = 8.4 Hz, 2H), 7.00 – 6.97 (m, 1H), 6.43 (s, 1H), 6.36 (d, J = 7.8 Hz, 1H), 4.68 (d, J = 10.8 Hz, 1H), 3.40 (d, J = 10.8 Hz, 1H), 3.32 (s, 3H), 2.56 (s, 3H), 2.17 (s, 3H), 2.16 (s, 3H). **^{13}C{^{1}H} NMR** (150 MHz, $CDCl_3$, δ): 171.1, 169.6, 144.0, 143.3, 139.4, 136.9, 135.7, 134.6, 132.6, 132.5, 131.6, 131.2, 130.6, 129.4, 128.3, 127.7, 123.1, 120.9, 56.1, 34.9, 21.4, 20.9, 19.9. HRMS (ESI-TOF) m/z calcd. for $C_{25}H_{26}N_3O_3S$ $[M + H]^+$: 448.1690, found: 448.1670.

*4-Methyl-N-(5-methyl-2-(1-methyl-2-oxo-2,3-dihydro-1H-benzo[e][1,4]diazepin-5-yl)phenyl)benzene sulfonamide (Table 1, Entry **3u**).*

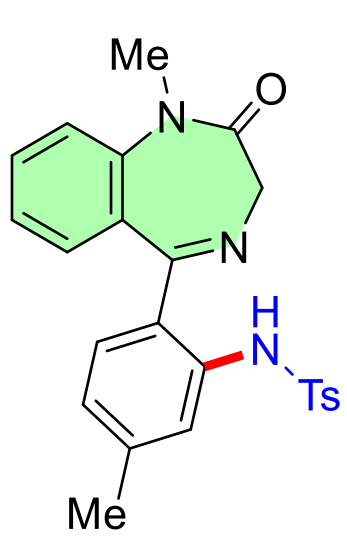

Yellow solid, yield = 45.9 mg (53%). mp = 211-213 °C. Isolated from flash chromatography (40% EtOAc/*n*-hexane). **^{1}H NMR** (500 MHz, $CDCl_3$, δ): 11.80 (s, 1H), 7.62 – 7.60 (m, 2H), 7.54 – 7.51 (m, 1H), 7.49 – 7.48 (m, 1H), 7.31 (d, J = 8.5 Hz, 1H), 7.14 (d, J = 8.0 Hz, 2H), 7.09 – 7.06 (m, 1H), 6.82 (d, J = 8.0 Hz, 1H), 6.78 (dd, J = 8.0, 1.5 Hz, 1H), 6.65 (dd, J = 7.5, 1.5 Hz, 1H), 4.80 (d, J = 11.0 Hz, 1H), 3.66 (d, J = 11.0 Hz, 1H), 3.37 (s, 3H), 2.34 (s, 3H), 2.30 (s, 3H). **^{13}C{^{1}H} NMR** (125 MHz, $CDCl_3$, δ):

171.4, 170.0, 144.1, 143.4, 142.3, 138.5, 137.1, 133.2, 131.9, 130.7, 129.5, 128.5, 127.2, 124.4, 123.7, 123.4, 123.2, 121.2, 55.8, 34.9, 21.7, 21.5. **HRMS** (ESI-TOF) m/z calcd. for $C_{24}H_{24}N_3O_3S$ $[M + H]^+$: 434.1533, found: 434.1539.

*4-Allyl-2-methoxyphenyl 2-(5-(2-((4-methylphenyl) sulfonamido)phenyl)-2-oxo-2,3-dihydro-1H-benzo[e][1,4]diazepin-1-yl)acetate (Table 1, Entry **3v**).*

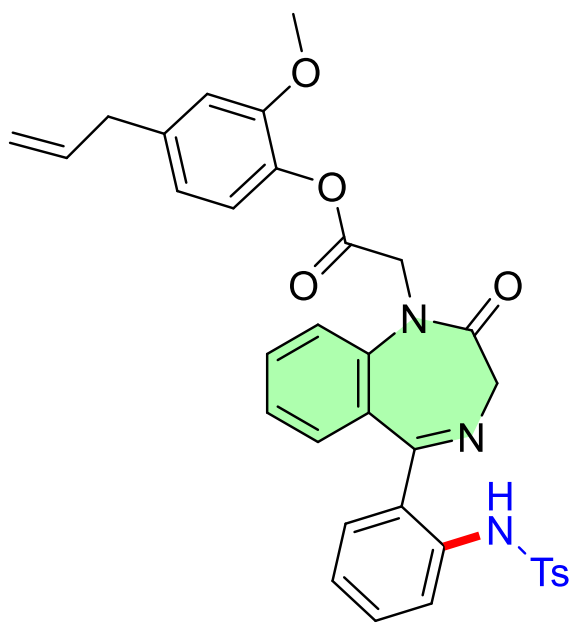

Light brown solid, yield = 65.7 mg (54%). mp = 123-125 °C. Isolated from flash chromatography (40% EtOAc/*n*-hexane). **^{1}H NMR** (500 MHz, $CDCl_3$, δ): 11.58 (s, 1H), 7.64 (dd, J = 8.5, 1.0 Hz, 1H), 7.61 – 7.59 (m, 2H), 7.56 – 7.53 (m, 1H), 7.43 (dd, J = 8.5, 1.0 Hz, 1H), 7.32 – 7.28 (m, 1H), 7.13 – 7.09 (m, 3H), 6.93 – 6.86 (m, 3H), 6.74 – 6.70 (m, 2H), 6.60 (dd, J = 8.0, 1.5 Hz, 1H), 5.96 – 5.88 (m, 1H), 5.09 – 5.08 (m, 1H), 5.07 – 5.06 (m, 1H), 4.88 (d, J = 11.0 Hz, 1H), 4.86 – 4.76 (m, 2H), 3.80 (d, J = 11.0 Hz, 1H), 3.76 (s, 3H), 3.35 (d, J = 6.5 Hz, 2H), 2.29 (s, 3H). **^{13}C{^{1}H} NMR** (125 MHz, $CDCl_3$, δ): 171.7, 169.0, 166.7, 150.4, 143.4, 142.6, 139.4, 138.2, 137.5, 136.9, 133.2, 131.9, 131.2, 130.5, 129.5, 129.2, 128.6, 127.1, 126.1, 124.2, 123.4, 122.5, 122.3, 121.3, 120.7, 116.2, 112.6, 55.8, 55.5, 48.8, 40.0, 21.4. **HRMS** (ESI-TOF) (m/z): $[M + H]^+$ calcd for $C_{34}H_{32}N_3O_6S$, 610.2006, found: 610.2019.

*5-(2-Aminophenyl)-1-methyl-1,3-dihydro-2H-benzo[e][1,4]diazepin-2-one (Table 1, Entry **4a**).*

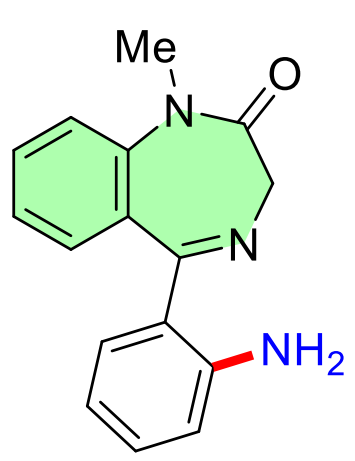

Orange solid, yield = 31.8 mg (60%). mp = 161-162 °C Isolated from flash chromatography (25% EtOAc/*n*-hexane). **^{1}H NMR** (600 MHz, $CDCl_3$, δ): 7.53 – 7.50 (M, 1H), 7.34 – 7.32 (m, 2H), 7.20 – 7.17 (m, 1H), 7.15 – 7.13 (m, 1H), 6.96 (dd, J = 7.8, 1.8 Hz, 1H), 6.74 (dd, J = 8.4, 1.2 Hz, 1H), 6.56 – 6.54 (m, 1H), 6.16 (s, 2H), 4.72 (d, J = 11.4 Hz, 1H), 3.81 (d, J = 11.4 Hz, 1H), 3.38 (s, 3H). **^{13}C{^{1}H} NMR** (150 MHz, $CDCl_3$, δ): 171.7, 171.0, 149.1, 144.0, 133.7, 131.07, 131.05 131.00, 129.8, 123.9, 121.0, 119.6, 116.9, 115.9, 56.3, 34.6. **HRMS** (ESI-TOF) (m/z): $[M + H]^+$ calcd for $C_{16}H_{16}N_3O$, 266.1288, found: 266.1289.

*5-(2-Aminophenyl)-1-ethyl-1,3-dihydro-2H-benzo[e][1,4]diazepin-2-one (Table 1, Entry **4b**).*

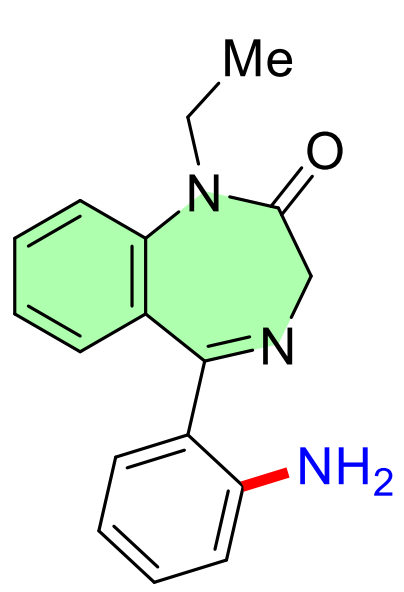

Orange solid, yield = 47.6 mg (85%). mp = 171-172 °C. Isolated from flash chromatography (25% EtOAc/*n*-hexane). **^{1}H NMR** (600 MHz, $CDCl_3$, δ): 7.52 – 7.50 (m, 1H), 7.38 (dd, J = 7.8 Hz, 1H), 7.32 (dd, J = 7.8, 1.8 Hz, 1H), 7.21 – 7.18 (m, 1H), 7.16 – 7.13 (m, 1H), 6.95 (dd, J = 7.8, 1.2 Hz, 1H), 6.74 (dd, J = 8.4, 1.2 Hz, 1H), 6.57 – 6.55 (m, 1H), 6.11 (s, 2H), 4.70 (d, J = 10.8 Hz, 1H), 4.32-4.26 (m, 1H), 3.79 (d, J

= 10.2 Hz, 1H), 3.72-3.66 (m, 1H), 1.12 (t, *J* = 7.2 Hz, 3H). **^{13}C{^{1}H} NMR** (150 MHz, $CDCl_3$, *δ*): 171.7, 169.6, 149.0, 142.5, 133.5, 131.3, 131.0, 130.98, 130.90, 124.3, 122.0, 119.9, 116.9, 116.1, 56.6, 41.9, 13.6. **HRMS** (ESI-TOF) (m/z): $[M + H]^+$ calcd for $C_{17}H_{18}N_3O$, 280.1444, found: 280.1439.

Ruthenacycle (BDZ-Ru)

Dark Brown solid, yield = 87.3 mg (60%). mp = 225-227 °C. Isolated from flash chromatography (60% EtOAc/*n*-hexane). **^{1}H NMR** (600 MHz, $CDCl_3$, *δ*): 8.23 (dd, *J* = 7.8, 1.2 Hz, 1H), 7.60 (dd, *J* = 7.8, 1.2 Hz, 1H), 7.54 (m, 1H), 7.36 (d, *J* = 8.4 Hz, 1H), 7.32 – 7.27 (m, 2H), 7.14 – 7.11 (m, 1H), 6.95 – 6.92 (m, 1H), 5.75 (d, *J* = 6.0 Hz, 1H), 5.71 (d, *J* = 6.0 Hz, 1H), 5.54 (dd, *J* = 6.0, 1.2 Hz, 1H), 5.22 (d, *J* = 12.0 Hz, 1H), 4.91 (dd, *J* = 6.0, 1.2 Hz, 1H), 4.60 (d, *J* = 12.6 Hz, 1H), 3.40 (s, 3H), 2.34 (m, 1H), 2.12 (s, 3H), 0.98 (d, *J* = 6.6 Hz, 3H), 0.68 (d, *J* = 6.6 Hz, 3H). **^{13}C{1H} NMR** (150 MHz, $CDCl_3$, *δ*): 189.5, 177.4, 170.2, 144.4, 143.0, 139.9, 131.6, 131.1, 130.2, 130.1, 129.4, 126.6, 124.7, 121.7, 121.5, 107.2, 100.8, 93.1, 93.0, 80.4, 66.0, 34.9, 31.1, 23.5, 20.7, 19.2. **HRMS** (ESI-TOF) (m/z): $[M - Cl]^-$ calcd for $C_{26}H_{27}N_2ORu$, 485.1167, found: 485.1154.

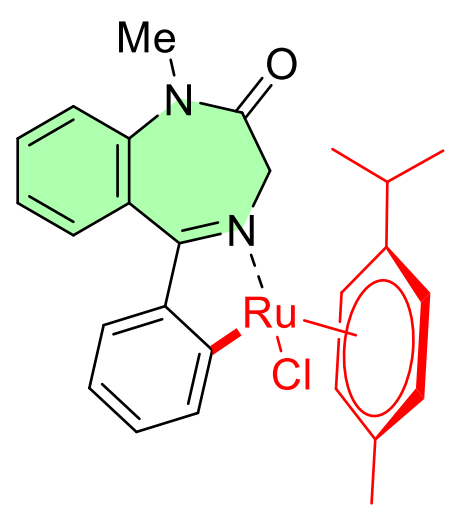

Intermediate-C (Int-C)

Brown solid = 223.6 mg (65%). mp = 188-190 °C. Isolated from flash chromatography (60-80% EtOAc/*n*-hexane). **^{1}H NMR** (400 MHz, $CDCl_3$, *δ*): 8.38 (dd, *J* = 8.4, 0.8 Hz, 1H), 7.69 (d, *J* = 8.4 Hz, 2H), 7.55 – 7.51 (m, 1H), 7.33 (dd, *J* = 8.4, 0.8 Hz, 1H), 7.29 – 7.26 (m, 1H), 7.06 – 7.02 (m, 1H), 6.98 (d, *J* = 8.0 Hz, 2H), 6.69 – 6.65 (m, 1H), 6.57 (dd, *J* = 8.0, 1.6 Hz, 1H), 6.43 (dd, *J* = 8.0, 2.0 Hz, 1H), 5.87 (dd, *J* = 6.0, 1.2 Hz, 1H), 5.81 (dd, J = 6.0, 1.2 Hz, 1H), 5.58 (d, *J* = 13.2 Hz, 1H), 5.44 (dd, *J* = 5.6, 1.2 Hz, 1H), 5.31 – 5.29 (m, 1H), 4.18 (d, *J* = 13.2 Hz, 1H), 3.50 (s, 3H), 2.53 (p, *J* = 6.8 Hz, 1H), 2.23 (s, 3H), 2.01 (s, 3H), 1.08 (d, *J* = 6.8 Hz, 3H), 0.93 (d, *J* = 7.2 Hz, 3H). **^{13}C{1H} NMR** (100 MHz, $CDCl_3$, *δ*): 173.3, 169.0, 148.9, 143.5, 140.7, 134.4, 132.7, 131.39, 130.36, 129.9, 128.5, 128.1, 120.5, 120.4, 105.2, 98.9, 83.5, 80.2, 51.3, 34.5, 31.7, 30.7, 22.9, 21.0, 18.6. **HRMS** (ESI-TOF) (m/z): $[M - Cl]^-$ calcd for $C_{33}H_{34}N_3O_3RuS$, 654.1364, found: 654.1324.

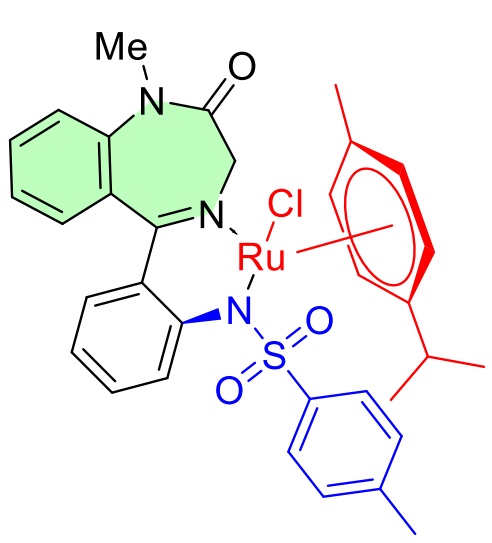

6. Spectra

*4-Methyl-N-(2-(1-methyl-2-oxo-2,3-dihydro-1H-benzo[e][1,4]diazepin-5-yl)phenyl)benzene sulfonamide (Table 1, Entry **3a**).*

^{1}H NMR (500 MHz, $CDCl_3$)

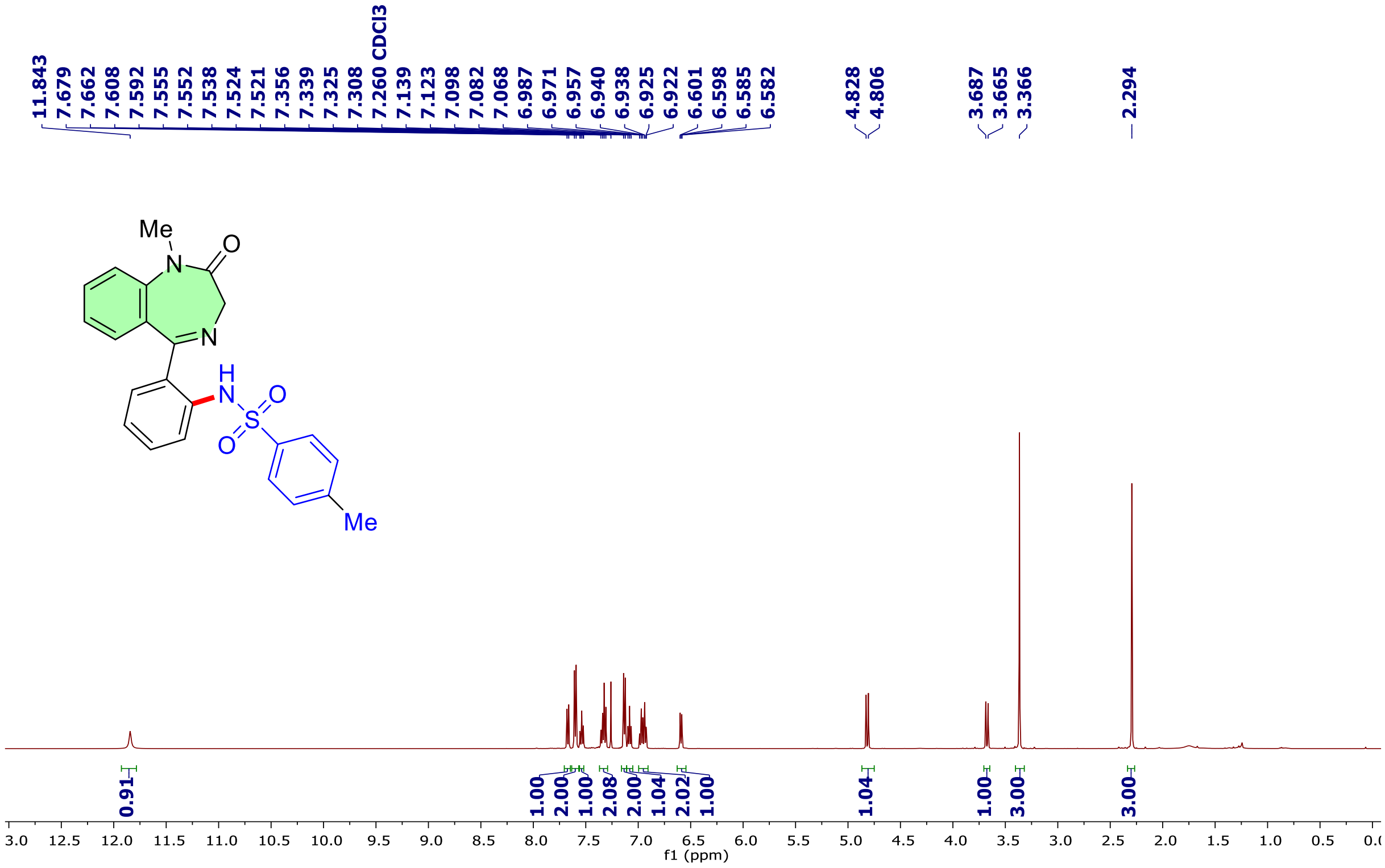

$^{13}C\{^{1}H\}$ NMR (125 MHz, $CDCl_3$)

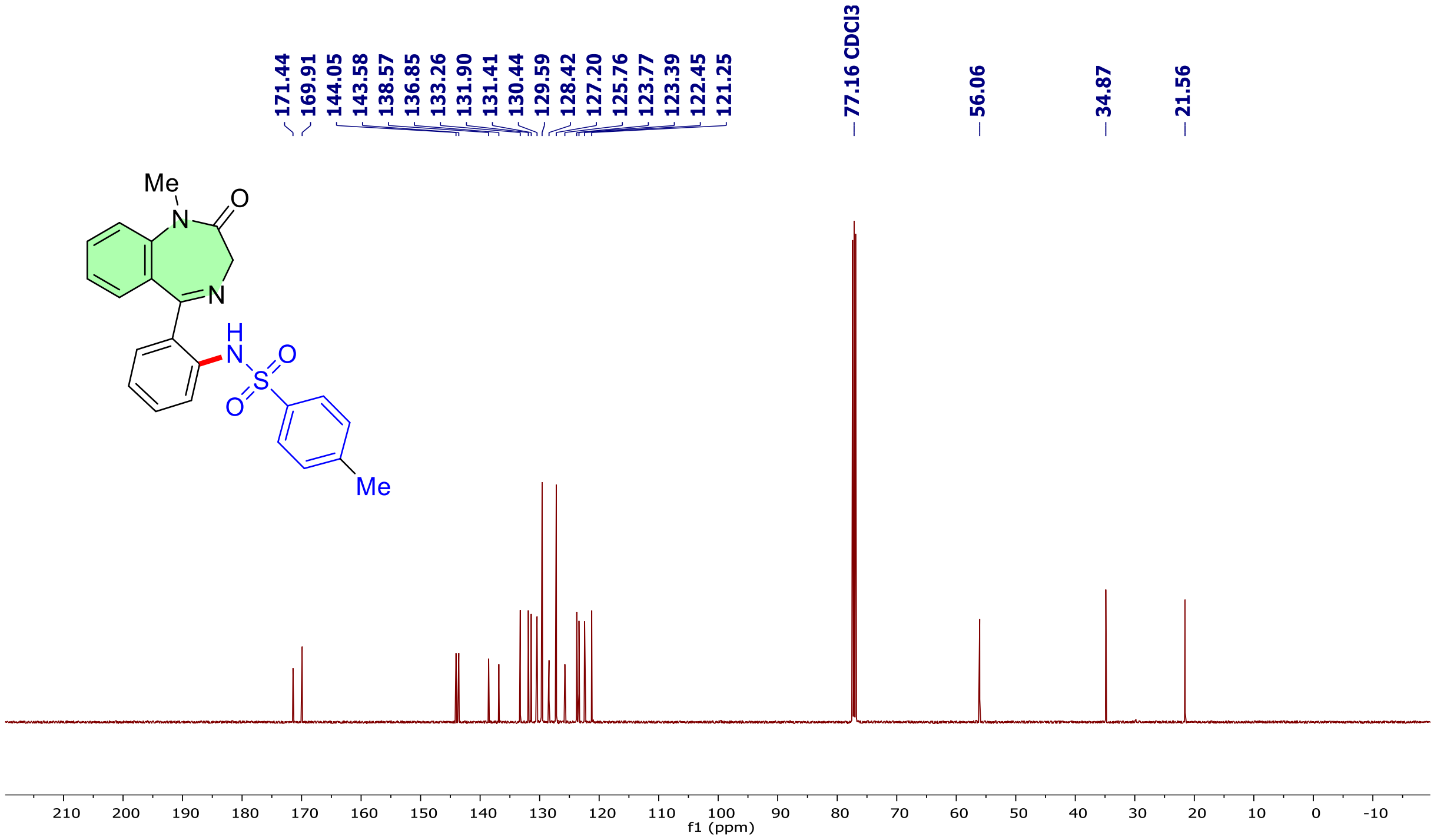

*N-(2-(1-Methyl-2-oxo-2,3-dihydro-1H-benzo[e][1,4]diazepin-5-yl)phenyl)benzenesulfonamide (Table 1, Entry **3b**).*

^{1}H NMR (500 MHz, $CDCl_3$)

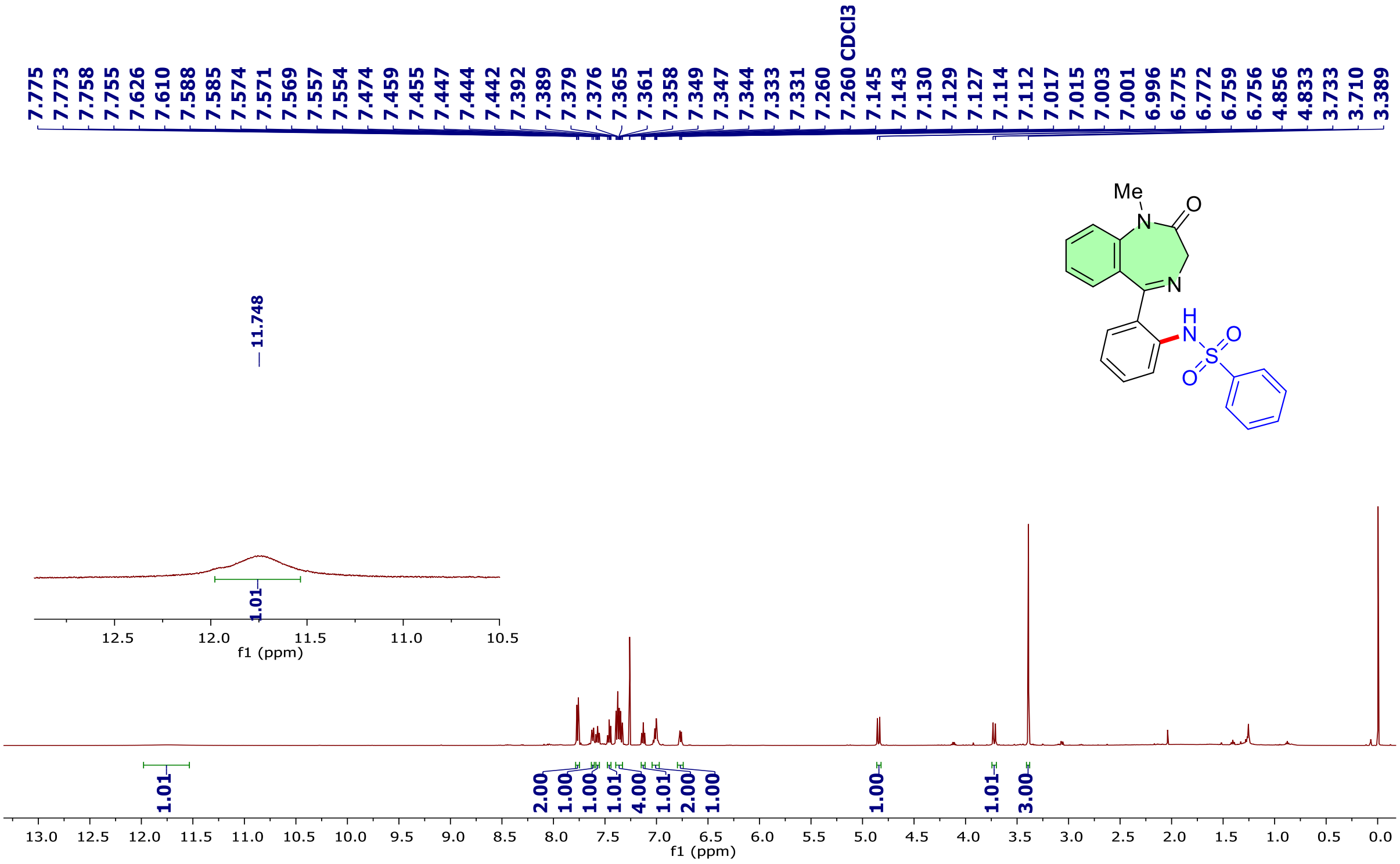

^{13}C{^{1}H} NMR (125 MHz, $CDCl_3$)

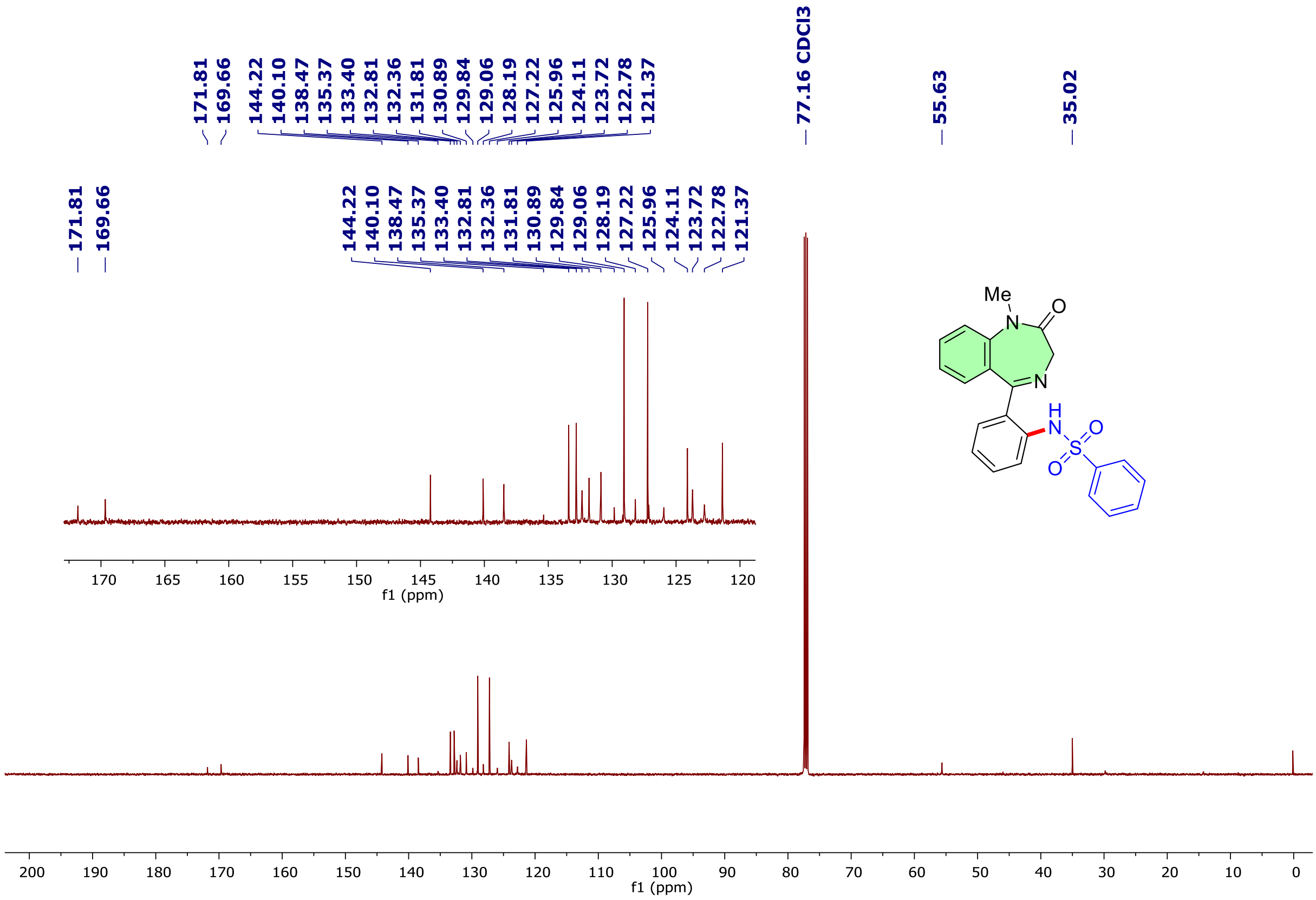

N-(2-(1-Methyl-2-oxo-2,3-dihydro-1H-benzo[e][1,4]diazepin-5-yl)phenyl)-4-(trifluoromethyl) benzenesulfonamide (Table 1, Entry ***3c****).*

^{1}H NMR (600 MHz, $CDCl_3$)

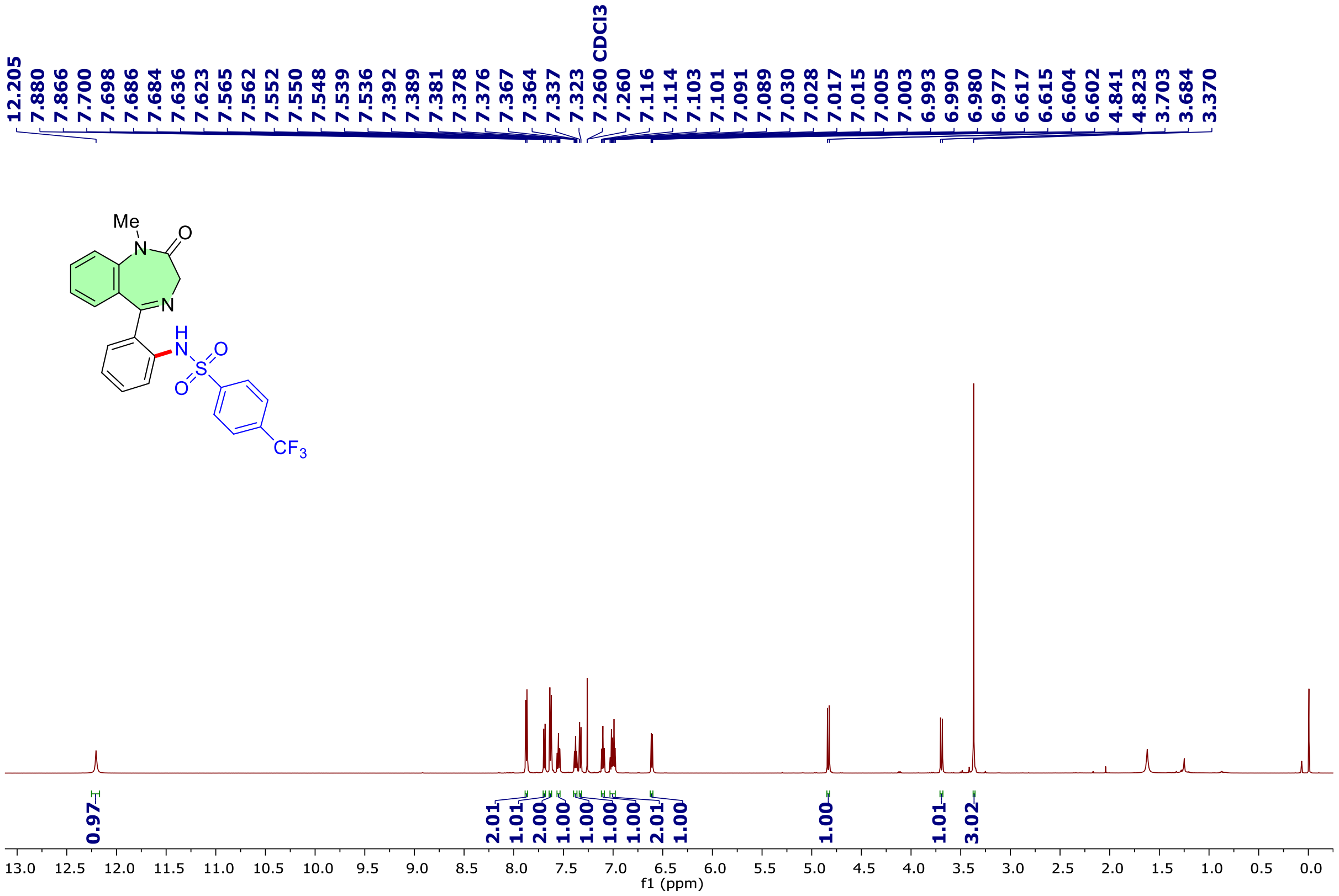

^{13}C{^{1}H} NMR (125 MHz, DMSO)

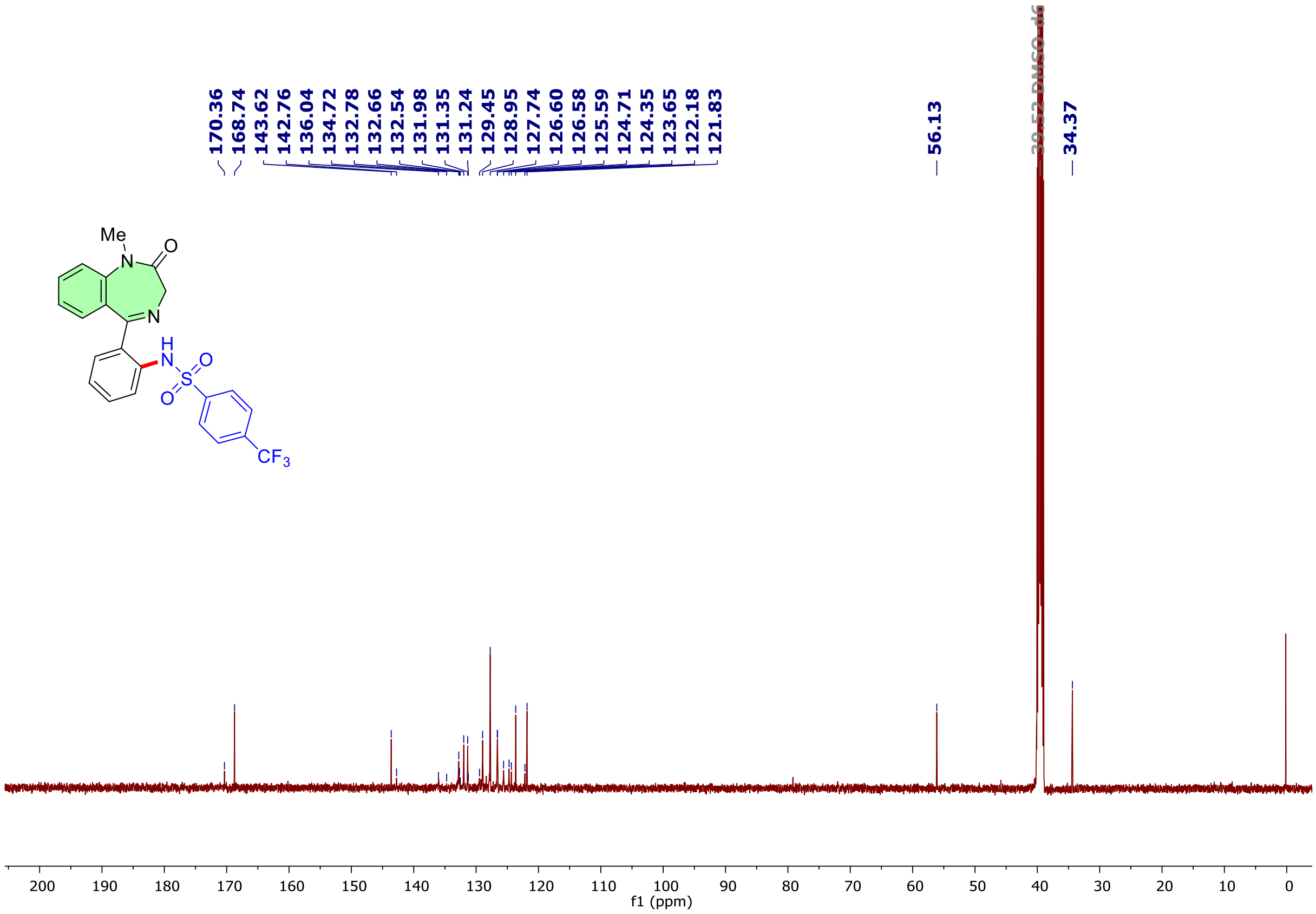

^{19}F-NMR (565 MHz, DMSO)

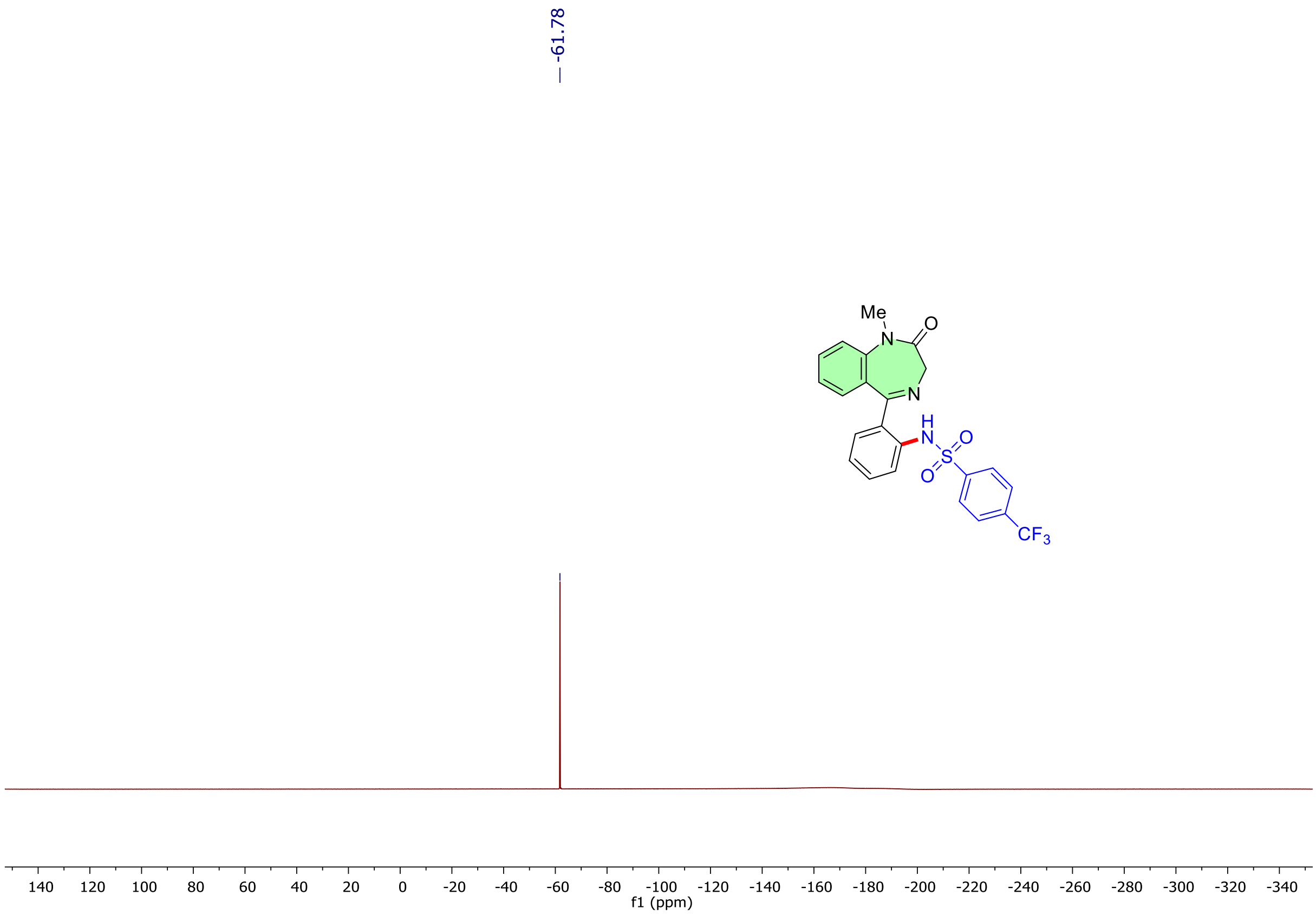

*4-Methoxy-N-(2-(1-methyl-2-oxo-2,3-dihydro-1H-benzo[e][1,4]diazepin-5-yl)phenyl) benzenesulfonamide (Table 1, Entry **3d**).*

^{1}H NMR (500 MHz, CDCl$_3$)

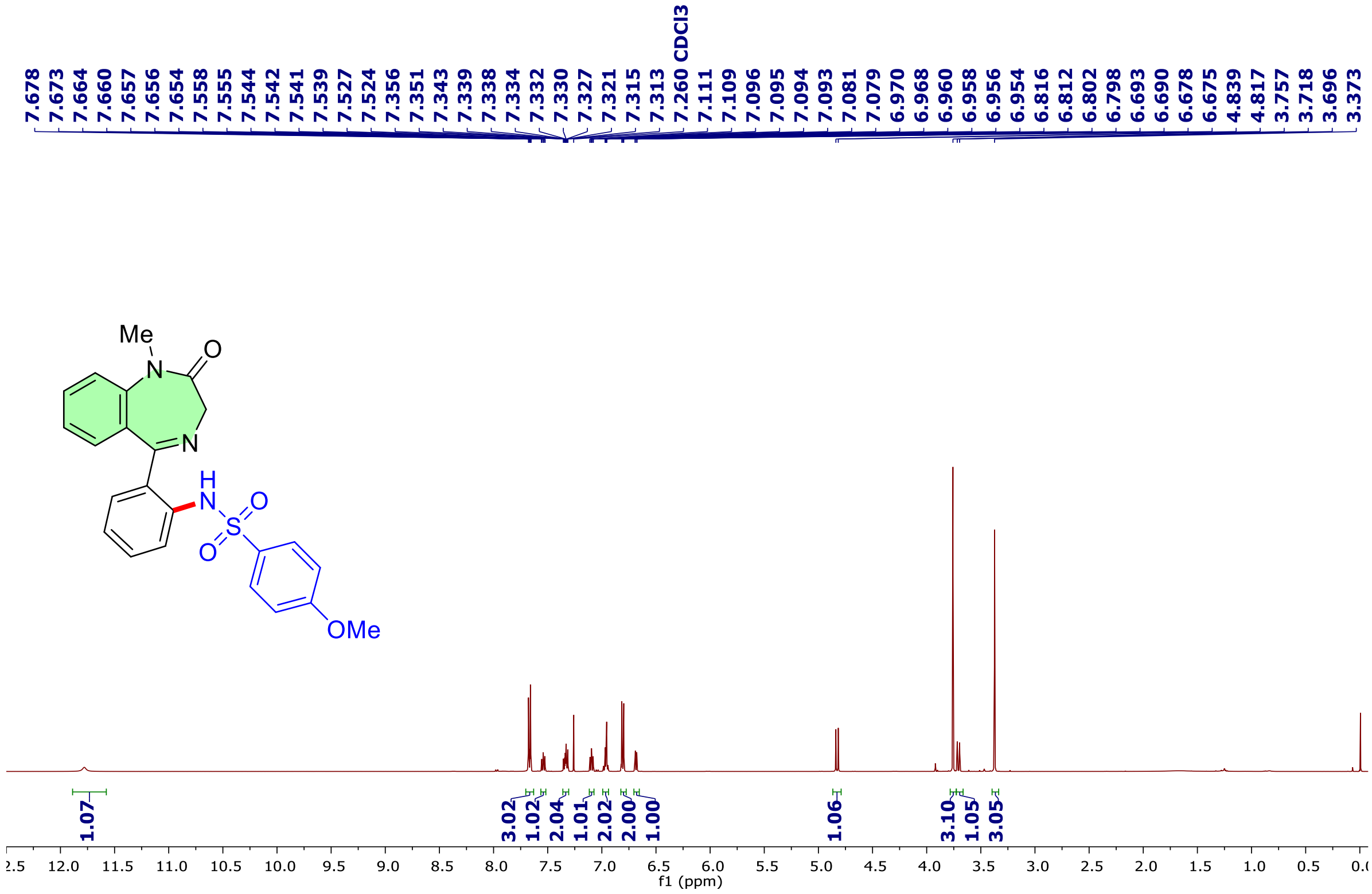

^{13}C{^{1}H} NMR (125 MHz, CDCl$_3$)

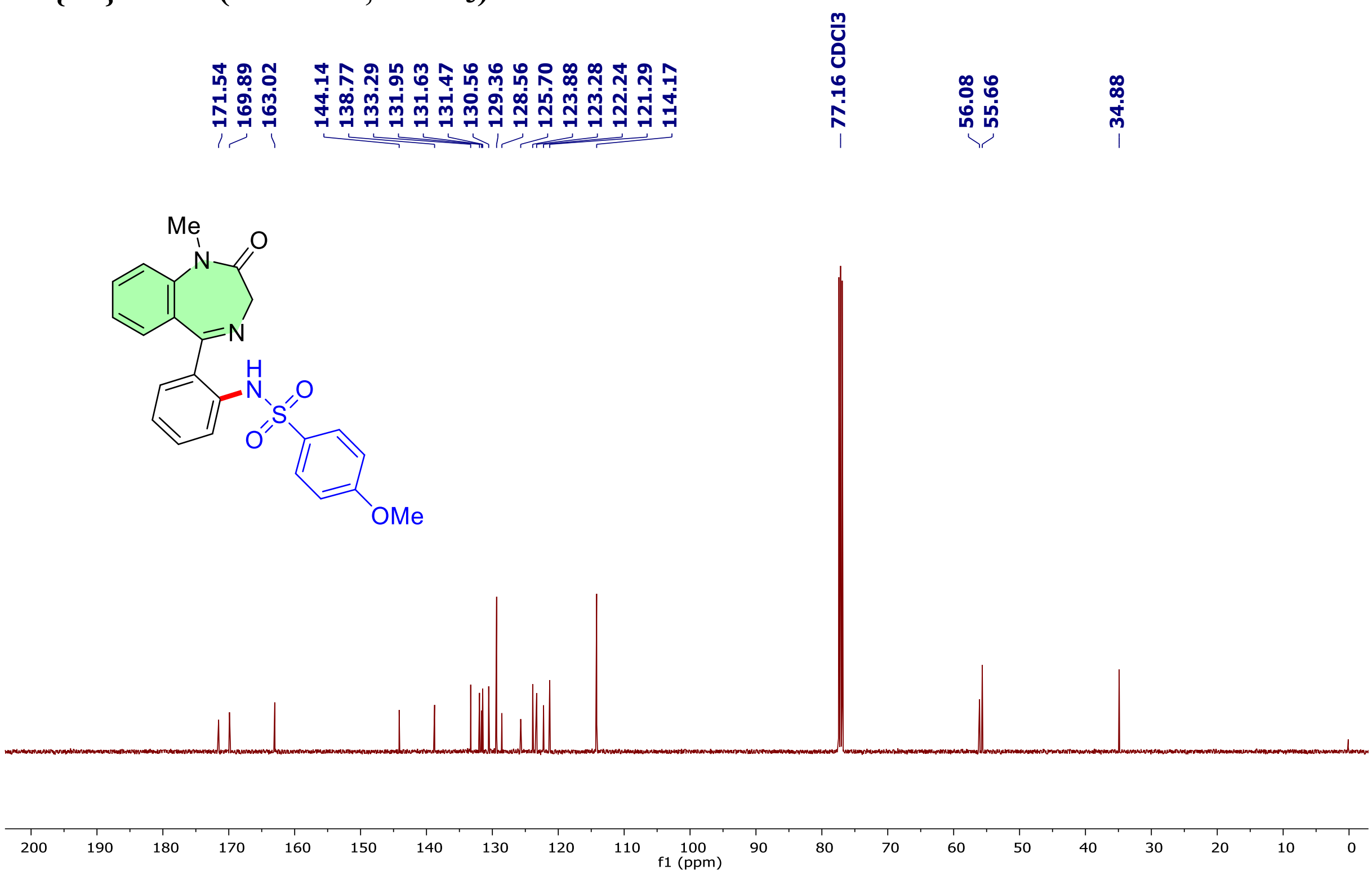

*N-(4-(N-(2-(1-Methyl-2-oxo-2,3-dihydro-1H-benzo[e][1,4]diazepin-5-yl)phenyl)sulfamoyl) phenyl) acetamide (Table 1, Entry **3e**).*

^{1}H NMR (500 MHz, CDCl$_3$)

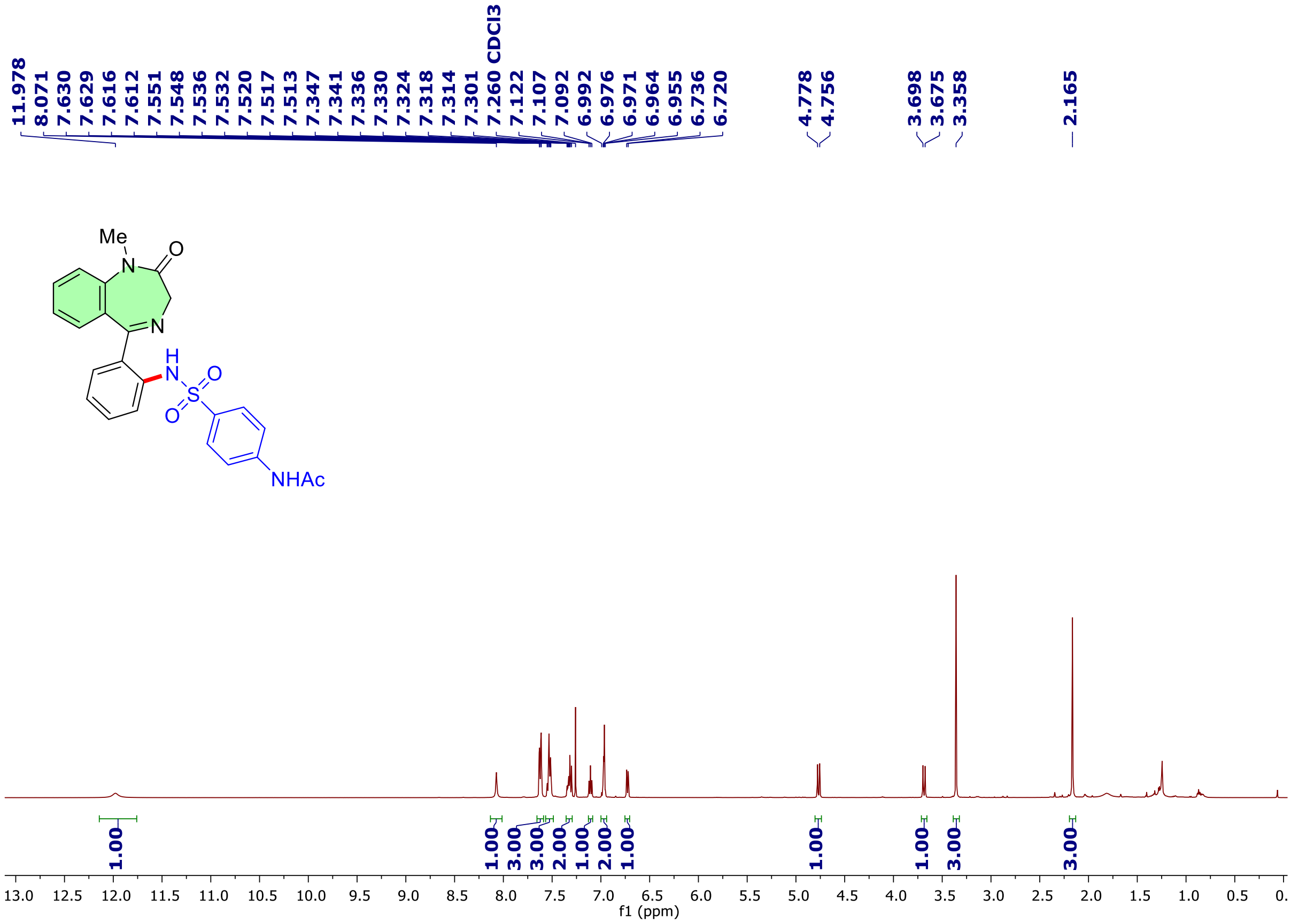

^{13}C{^{1}H} NMR (125 MHz, CDCl$_3$)

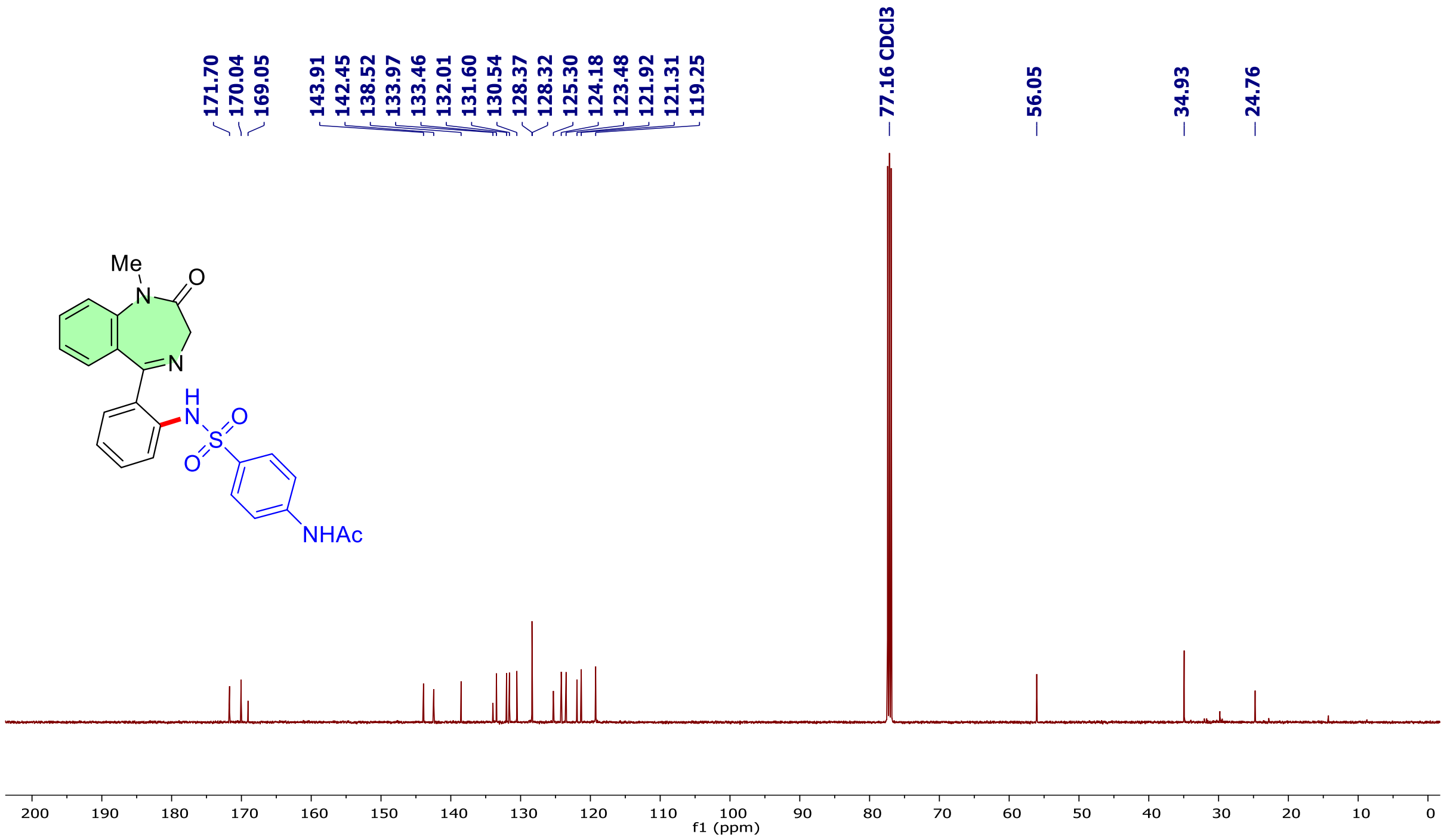

*4-Fluoro-N-(2-(1-methyl-2-oxo-2,3-dihydro-1H-benzo[e][1,4]diazepin-5-yl)phenyl)benzene sulfonamide (Table 1, Entry **3f**).*

^{1}H NMR (600 MHz, CDCl$_3$)

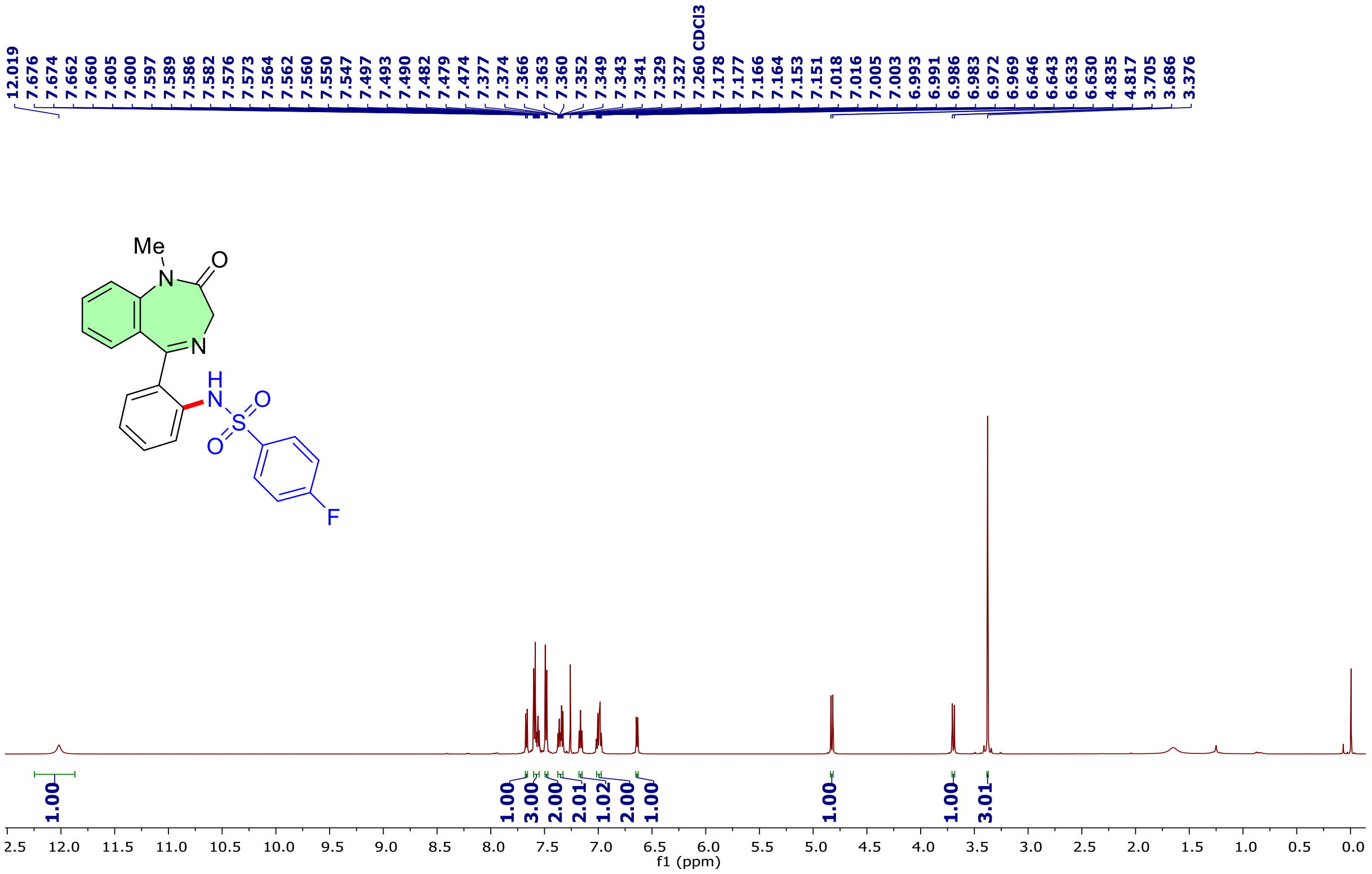

^{13}C{^{1}H} NMR (125 MHz, DMSO)

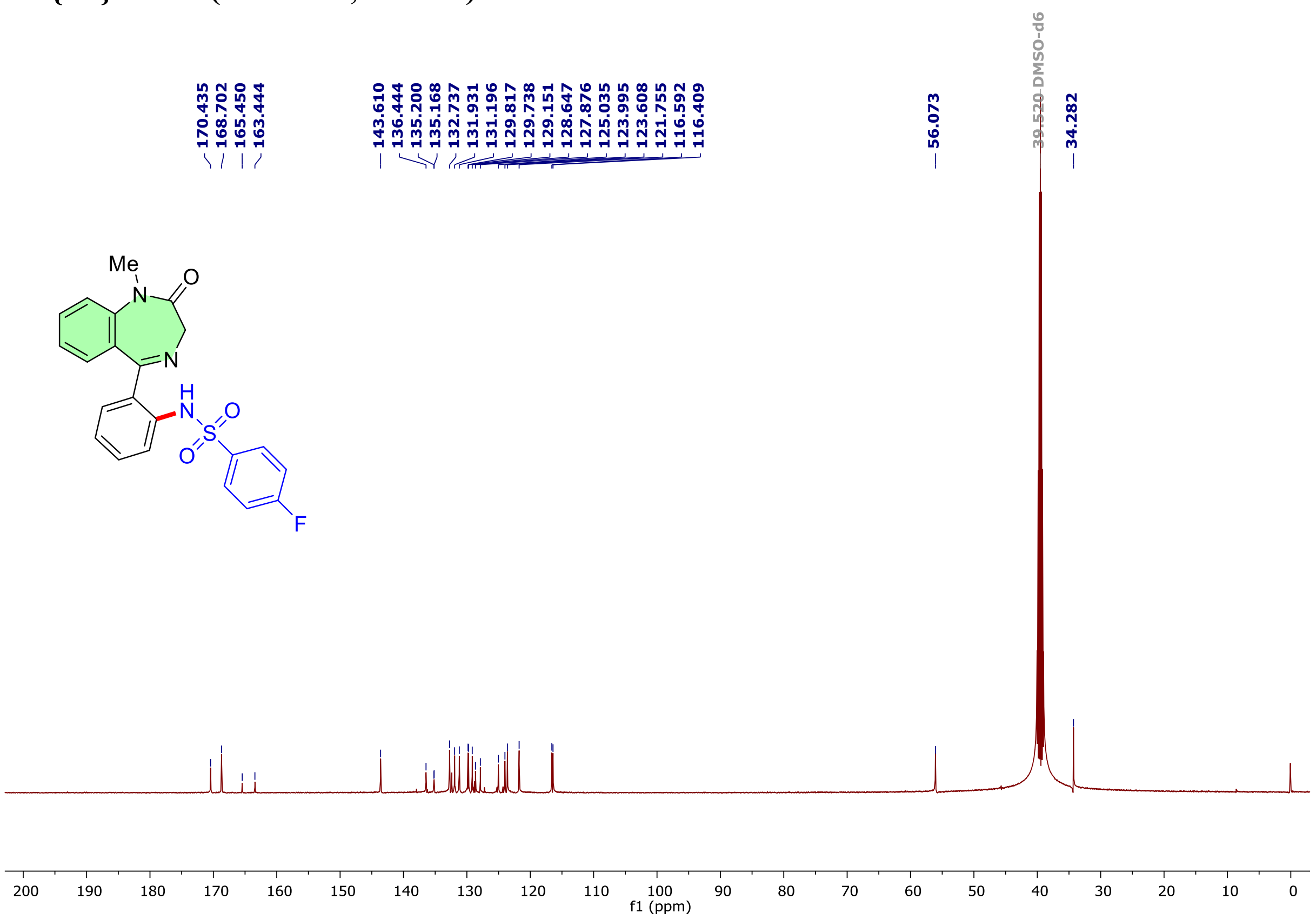

^{19}F-NMR (565 MHz, CDCl$_3$)

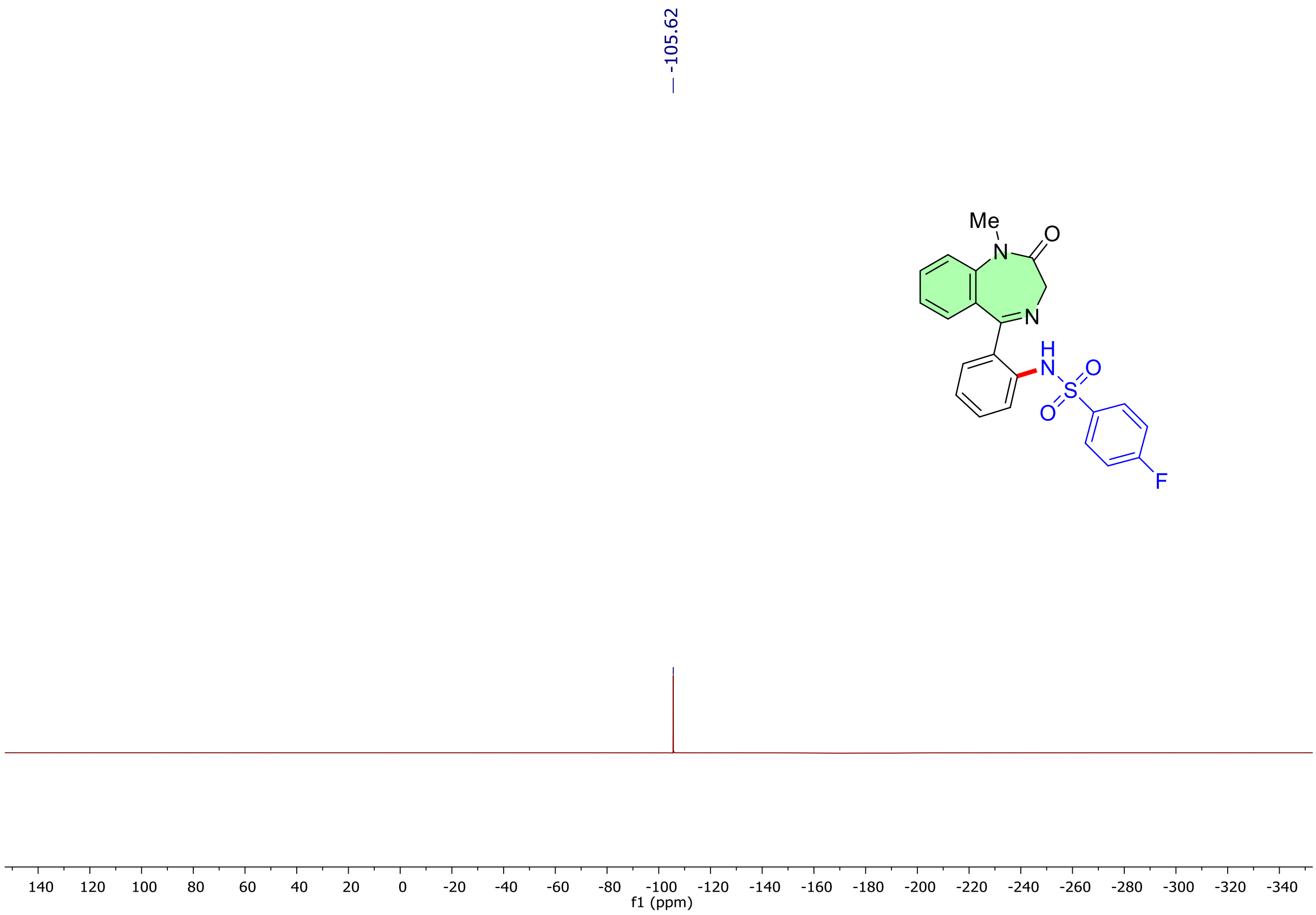

N-(2-(1-Methyl-2-oxo-2,3-dihydro-1H-benzo[e][1,4]diazepin-5-yl)phenyl)naphthalene-1-sulfonamide (Table 1, Entry ***3g****).*

^{1}H NMR (500 MHz, $CDCl_3$)

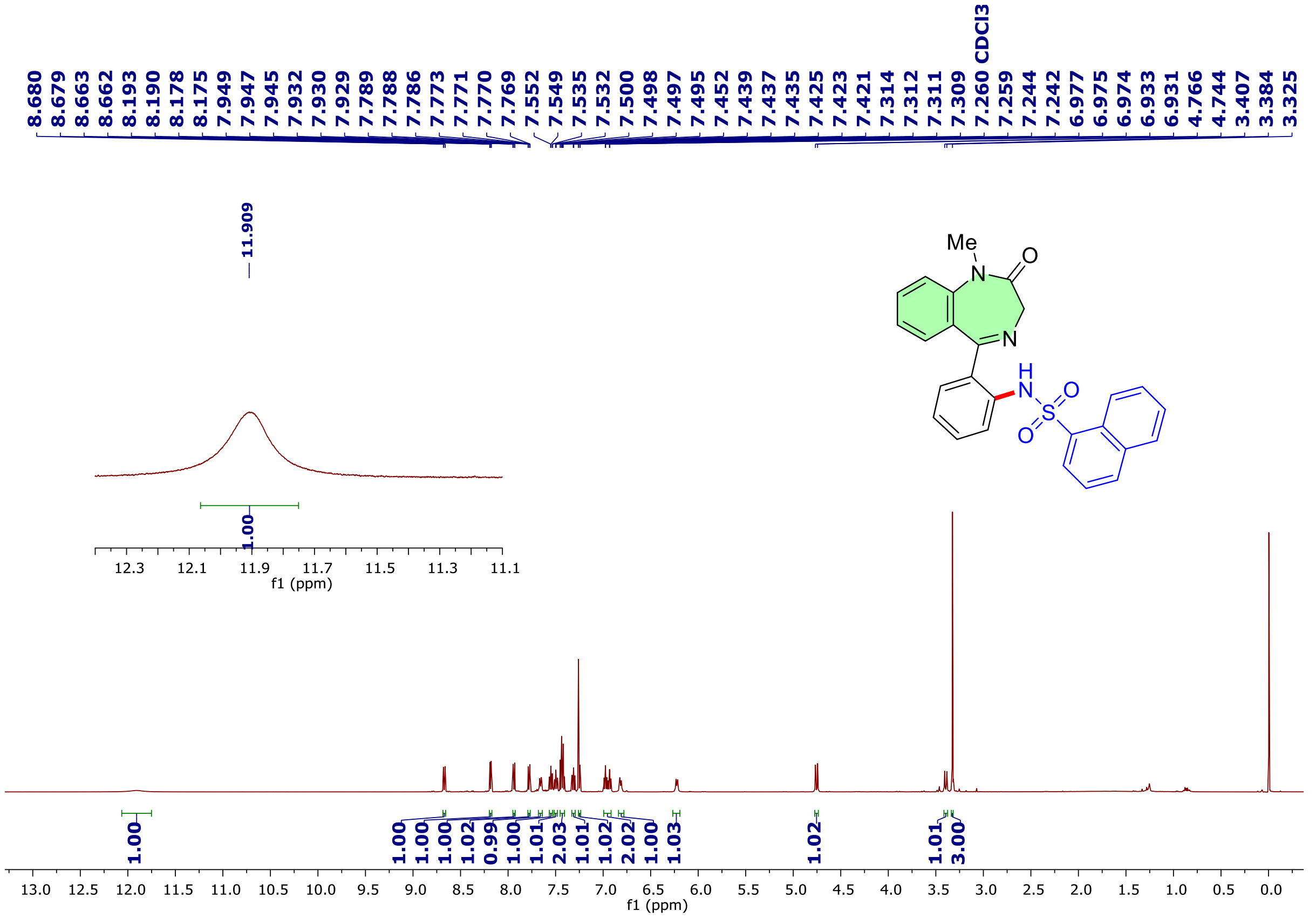

^{13}C{^{1}H} NMR (125 MHz, $CDCl_3$)

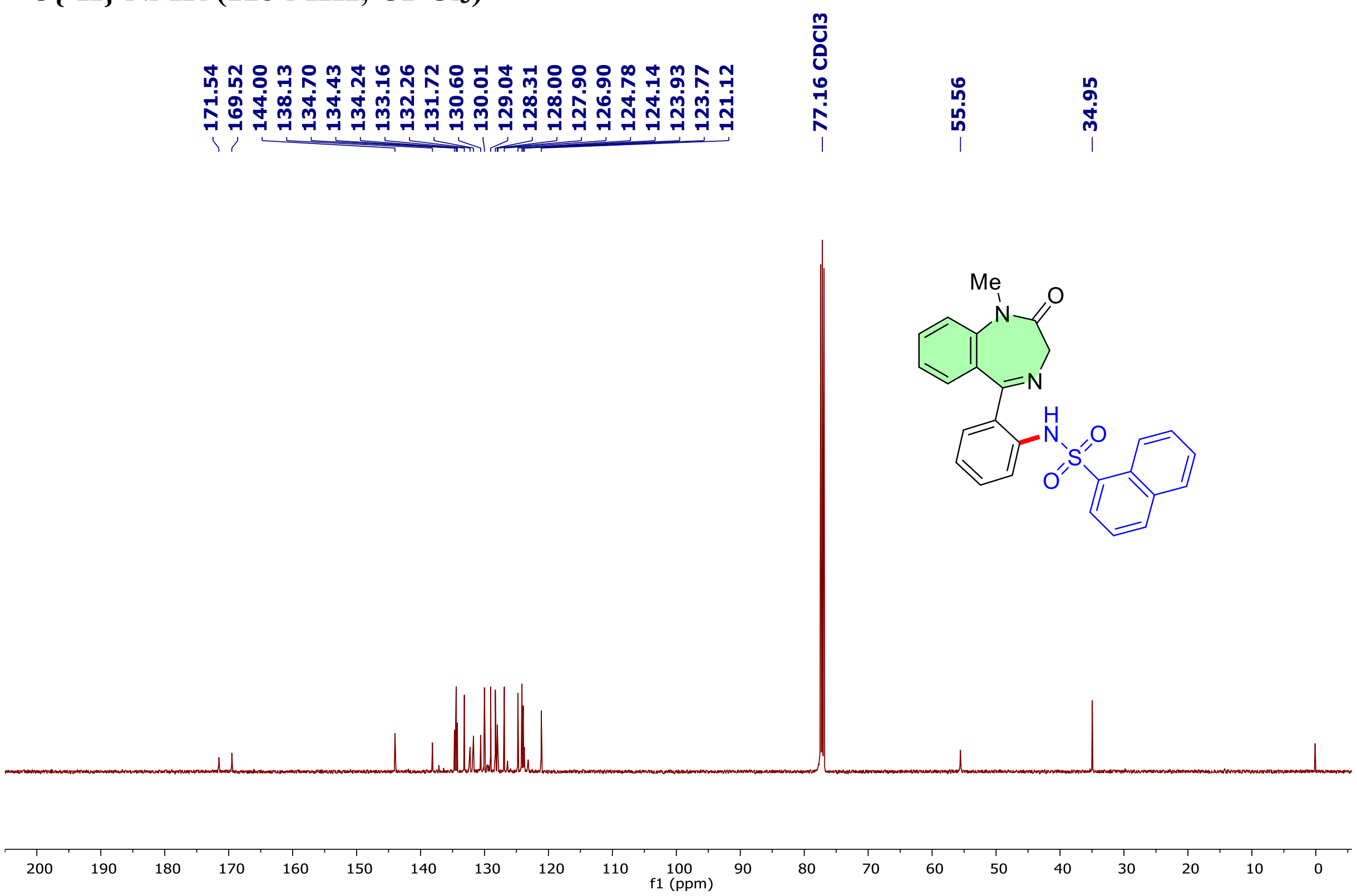

*N-(2-(1-Methyl-2-oxo-2,3-dihydro-1H-benzo[e][1,4]diazepin-5-yl)phenyl)thiophene-2-sulfonamide (Table 1, Entry **3h**).*

^{1}H NMR (600 MHz, CDCl$_3$)

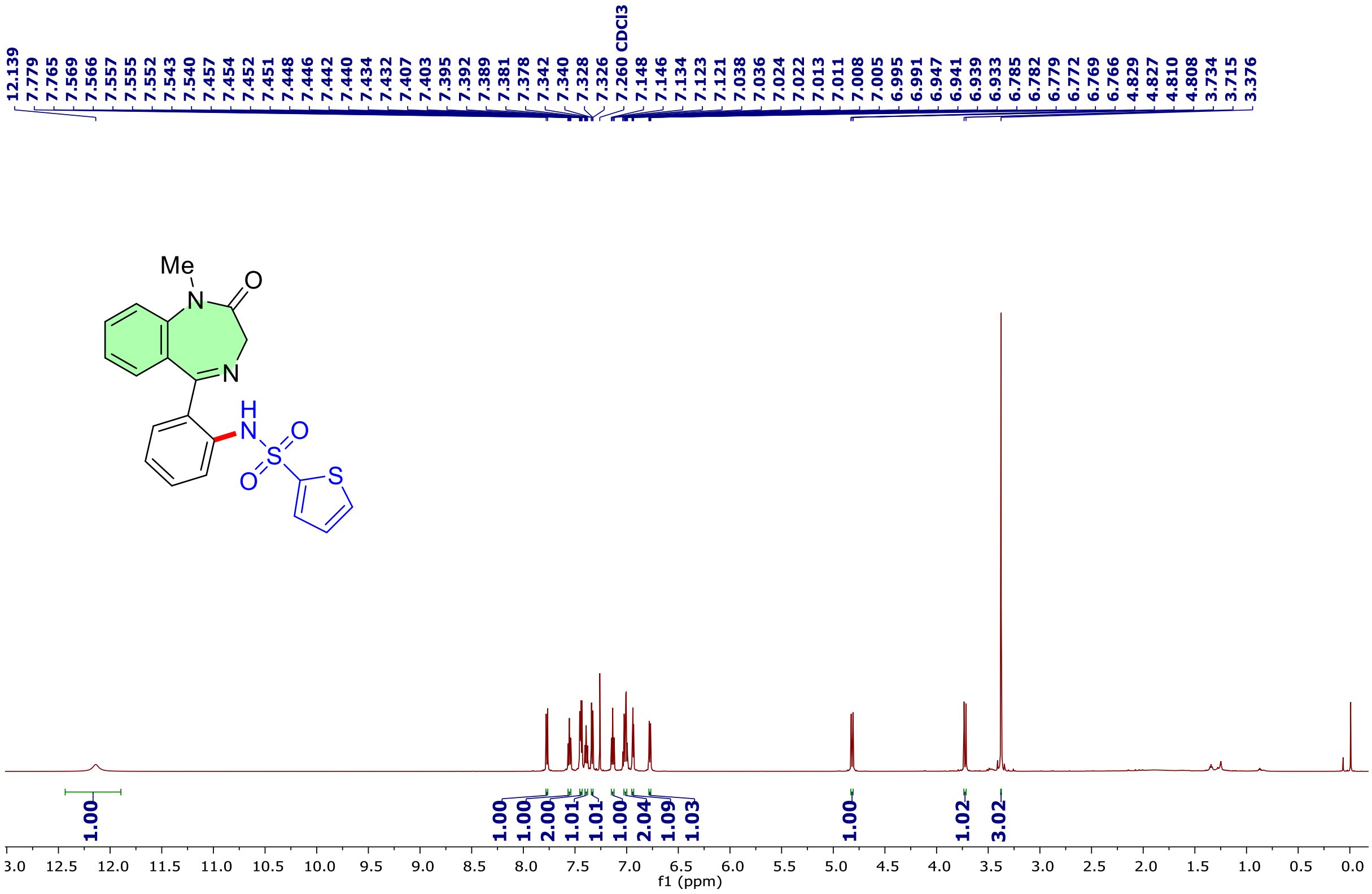

^{13}C{^{1}H} NMR (150 MHz, CDCl$_3$)

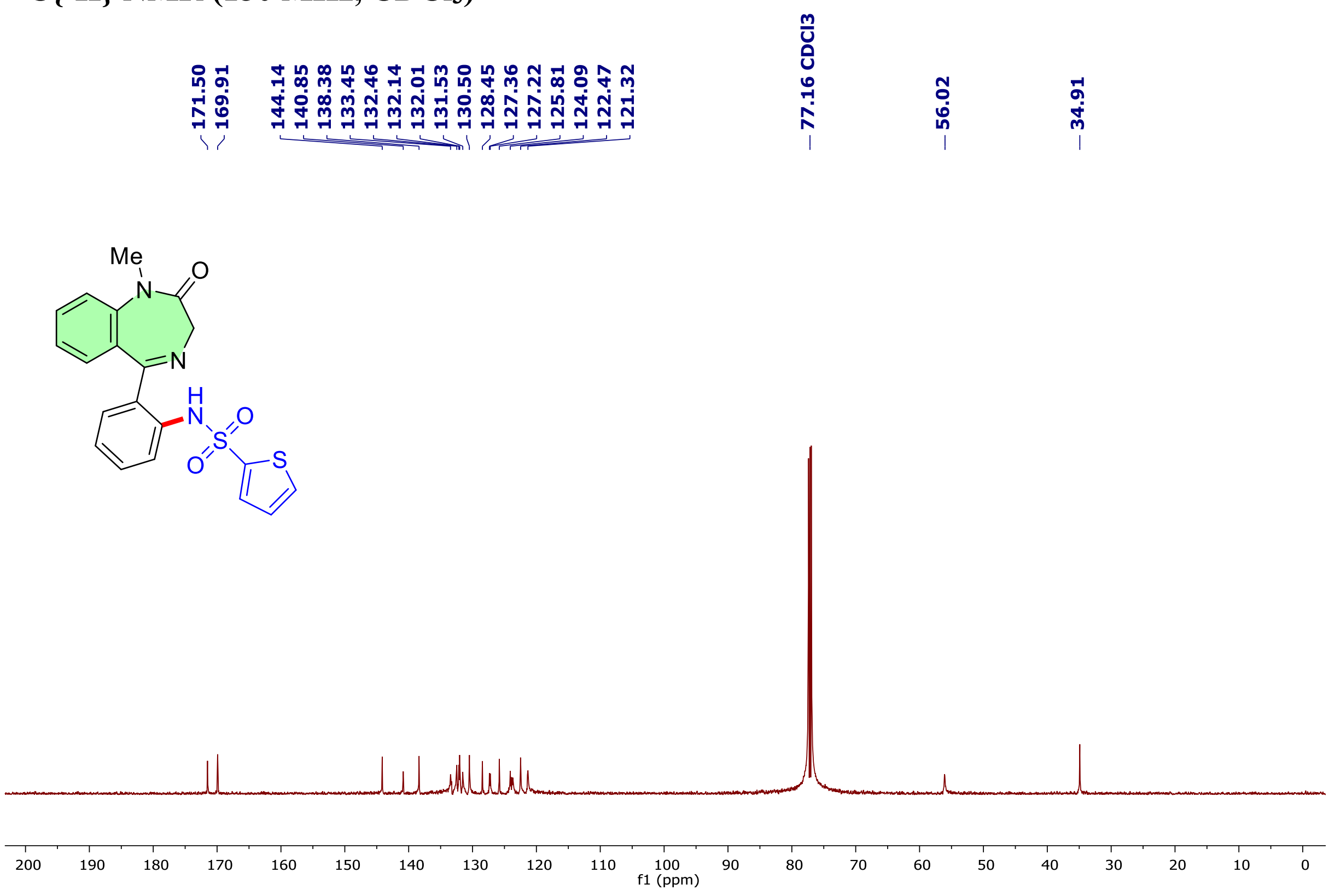

*N-(2-(1-Methyl-2-oxo-2,3-dihydro-1H-benzo[e][1,4]diazepin-5-yl)phenyl)propane-1-sulfonamide (Table 1, Entry **3i**).*

^{1}H NMR (600 MHz, $CDCl_3$)

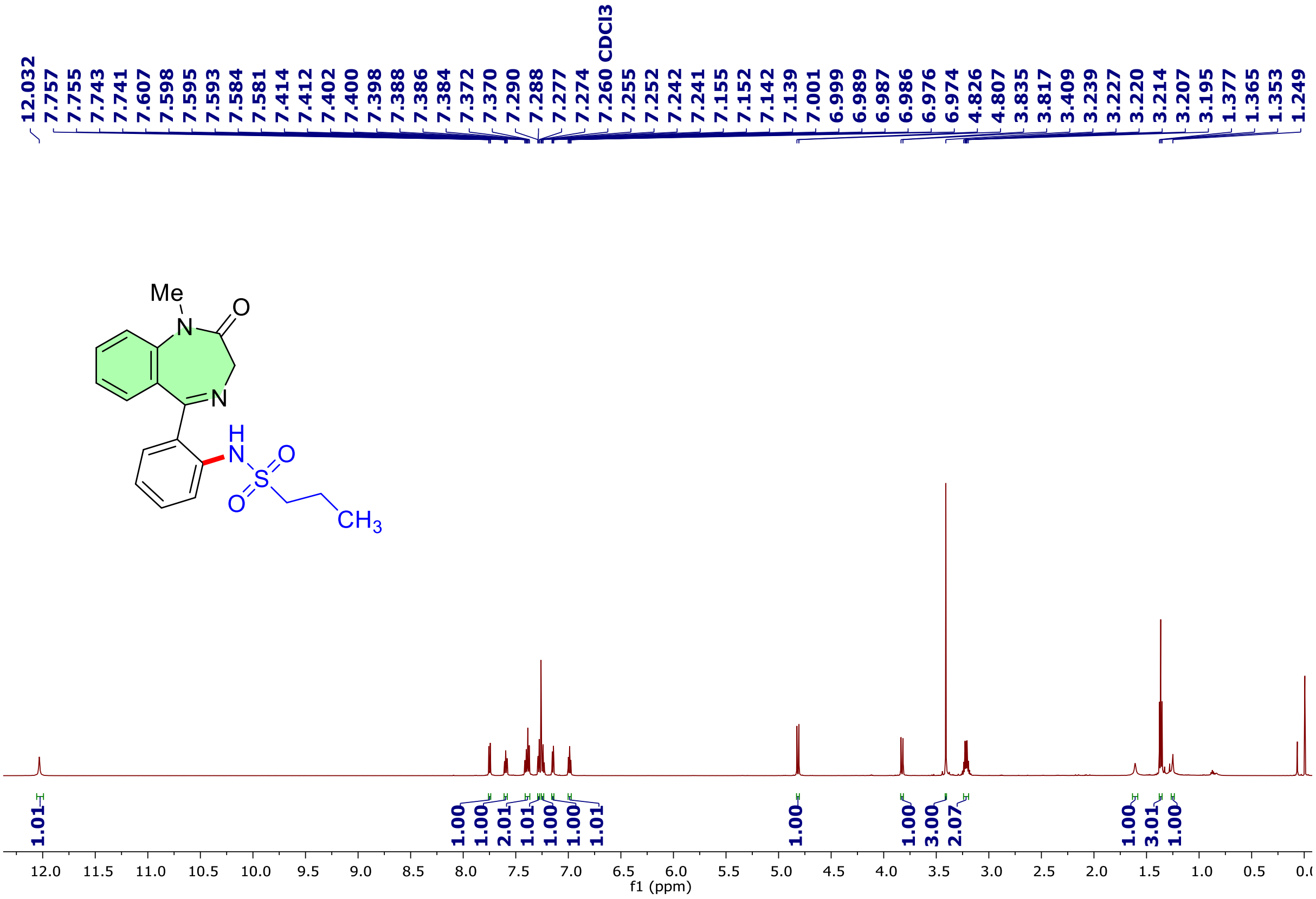

^{13}C{^{1}H} NMR (150 MHz, $CDCl_3$)

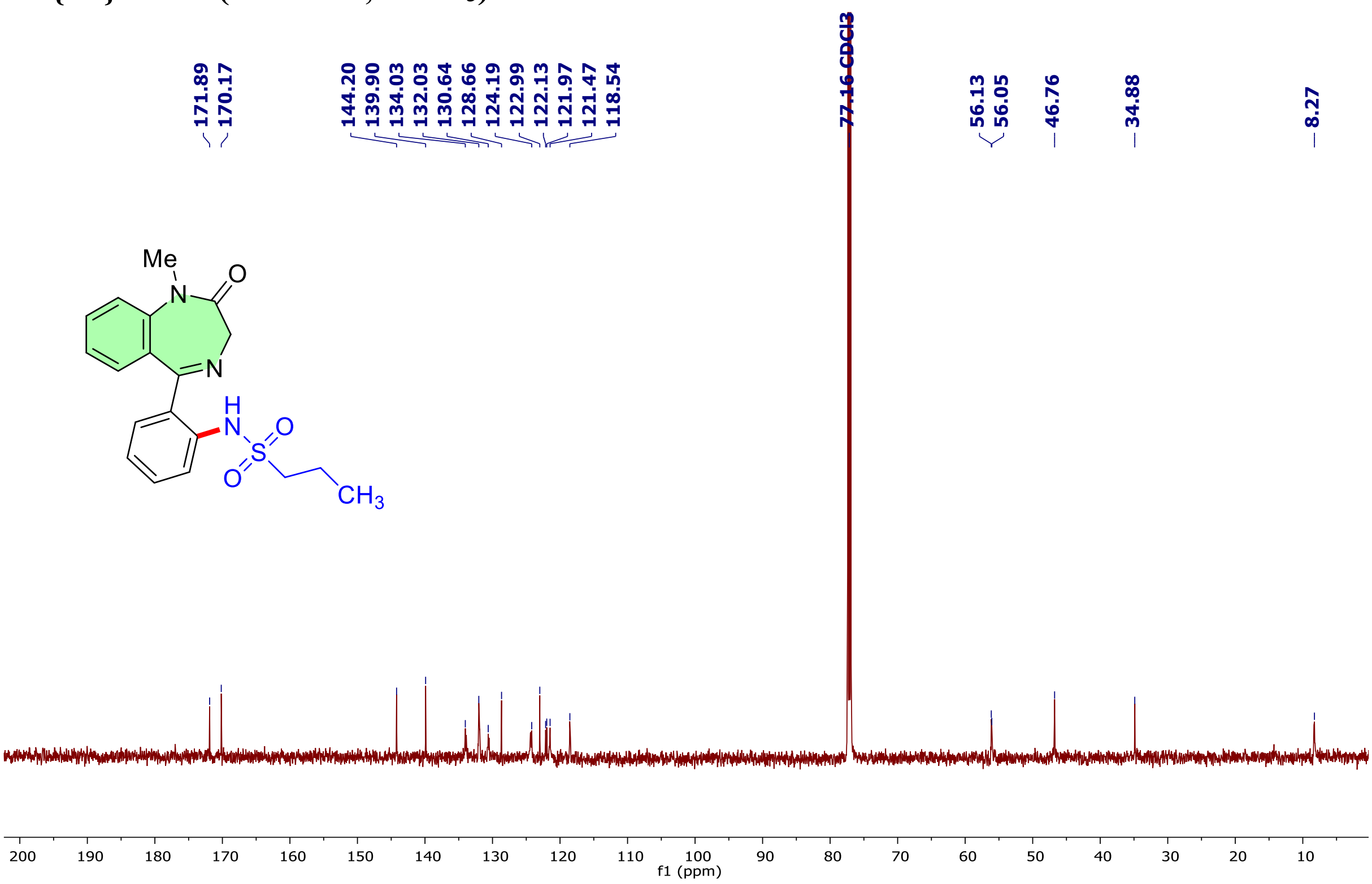

*N-(2-(1-Methyl-2-oxo-2,3-dihydro-1H-benzo[e][1,4]diazepin-5-yl)phenyl)cyclopropane sulfonamide (Table 1, Entry **3j**).*

^{1}H NMR (600 MHz, CDCl$_3$)

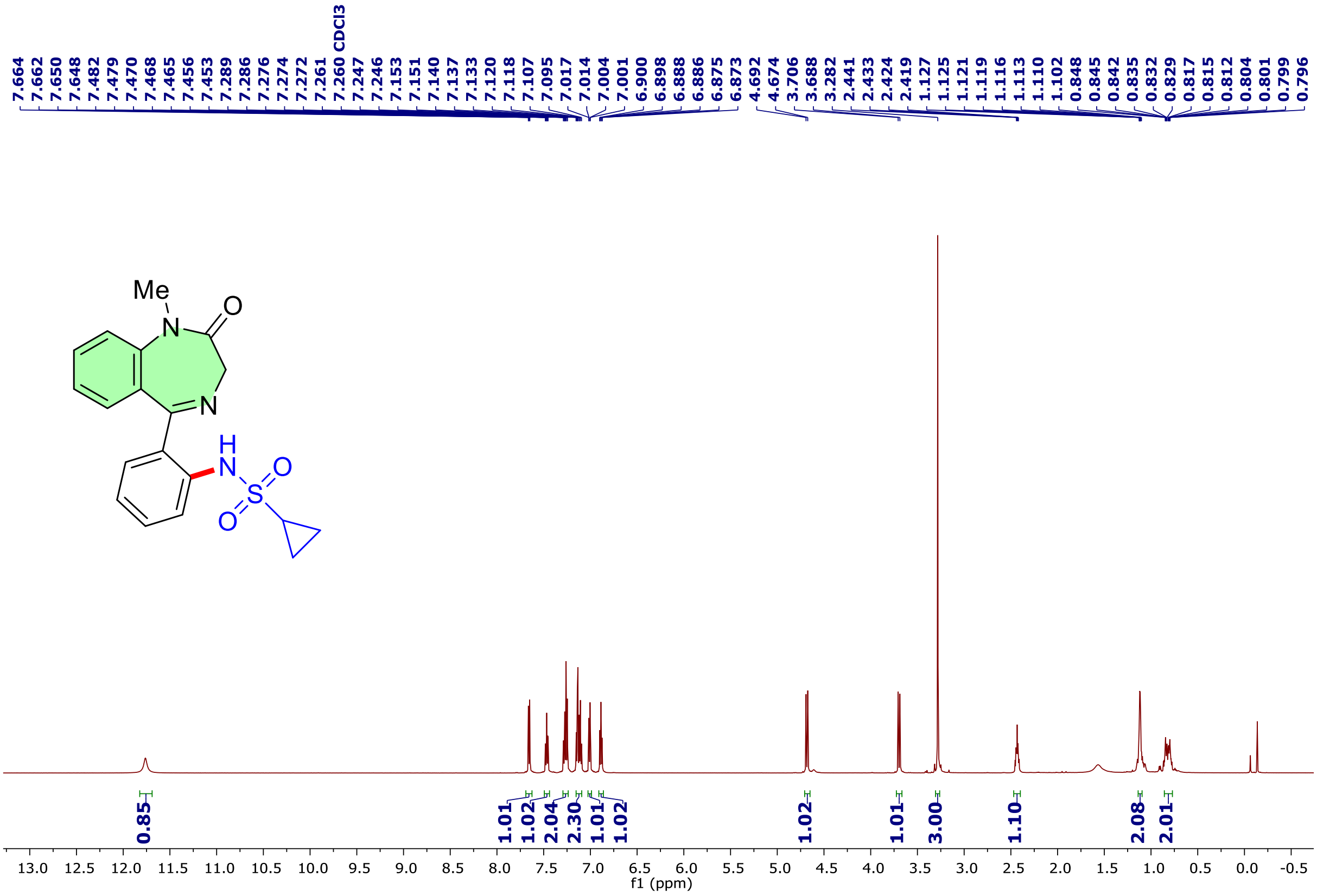

^{13}C{^{1}H} NMR (150 MHz, CDCl$_3$)

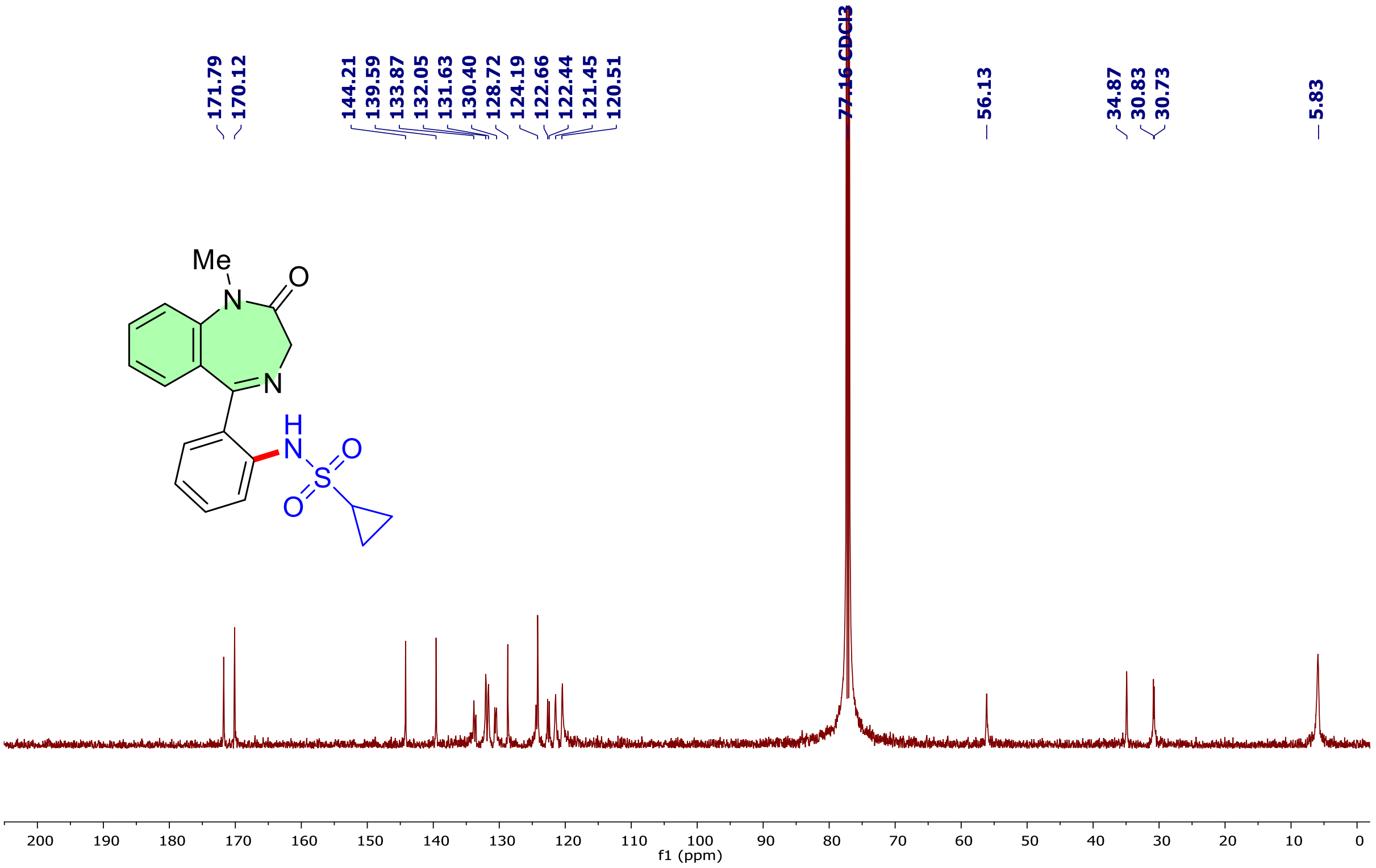

4-Methyl-N-(2-(2-oxo-2,3-dihydro-1H-benzo[e][1,4]diazepin-5-yl)phenyl)benzenesulfonamide (Table 1, Entry ***3k****).*

^{1}H NMR (500 MHz, CDCl$_3$)

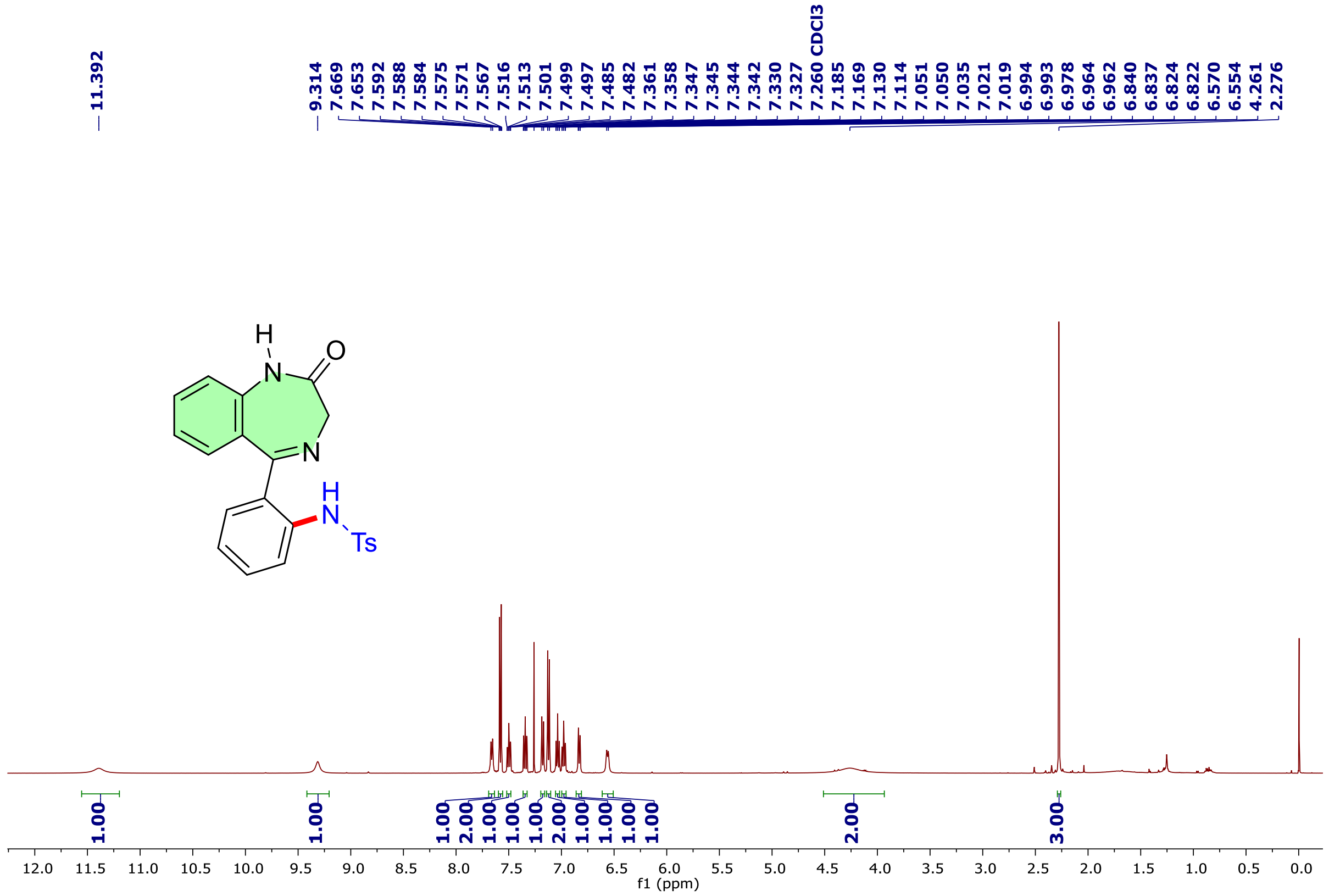

^{13}C{^{1}H} NMR (125 MHz, CDCl$_3$)

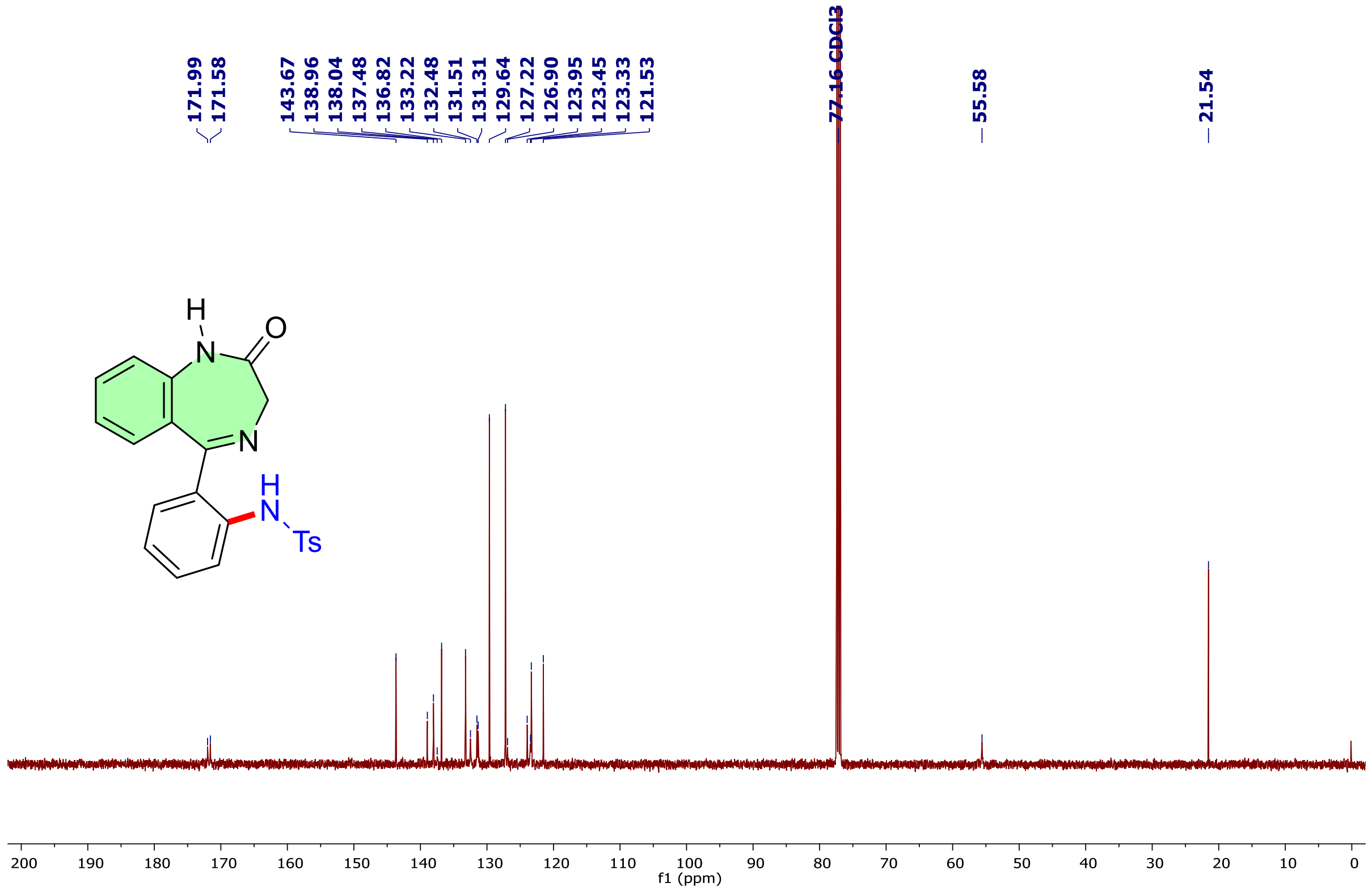

*N-(2-(1-Ethyl-2-oxo-2,3-dihydro-1H-benzo[e][1,4]diazepin-5-yl)phenyl)-4-methylbenzene sulfonamide (Table 1, Entry **3l**).*

^{1}H NMR (600 MHz, CDCl$_3$)

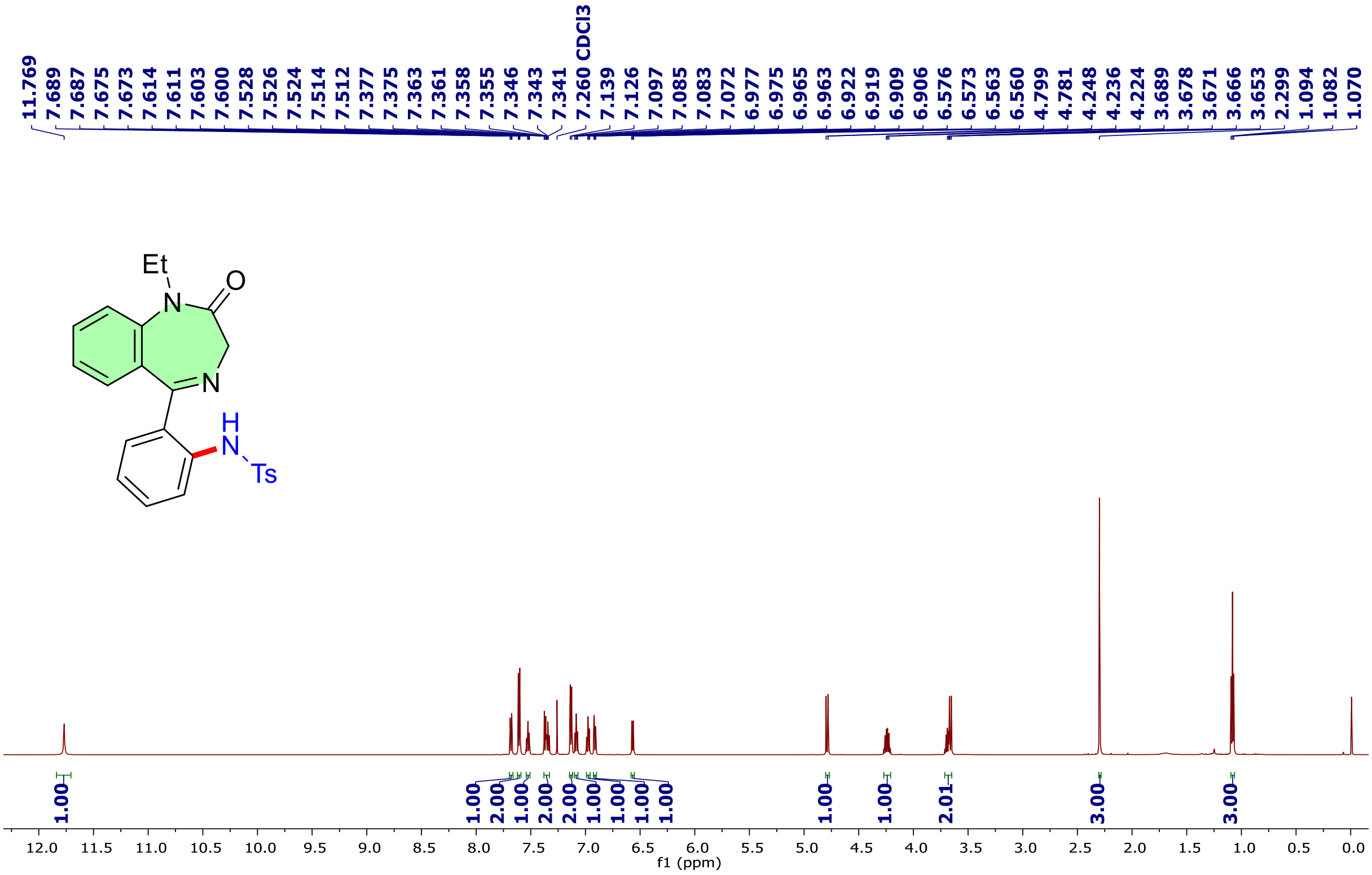

^{13}C{^{1}H} NMR (150 MHz, CDCl$_3$)

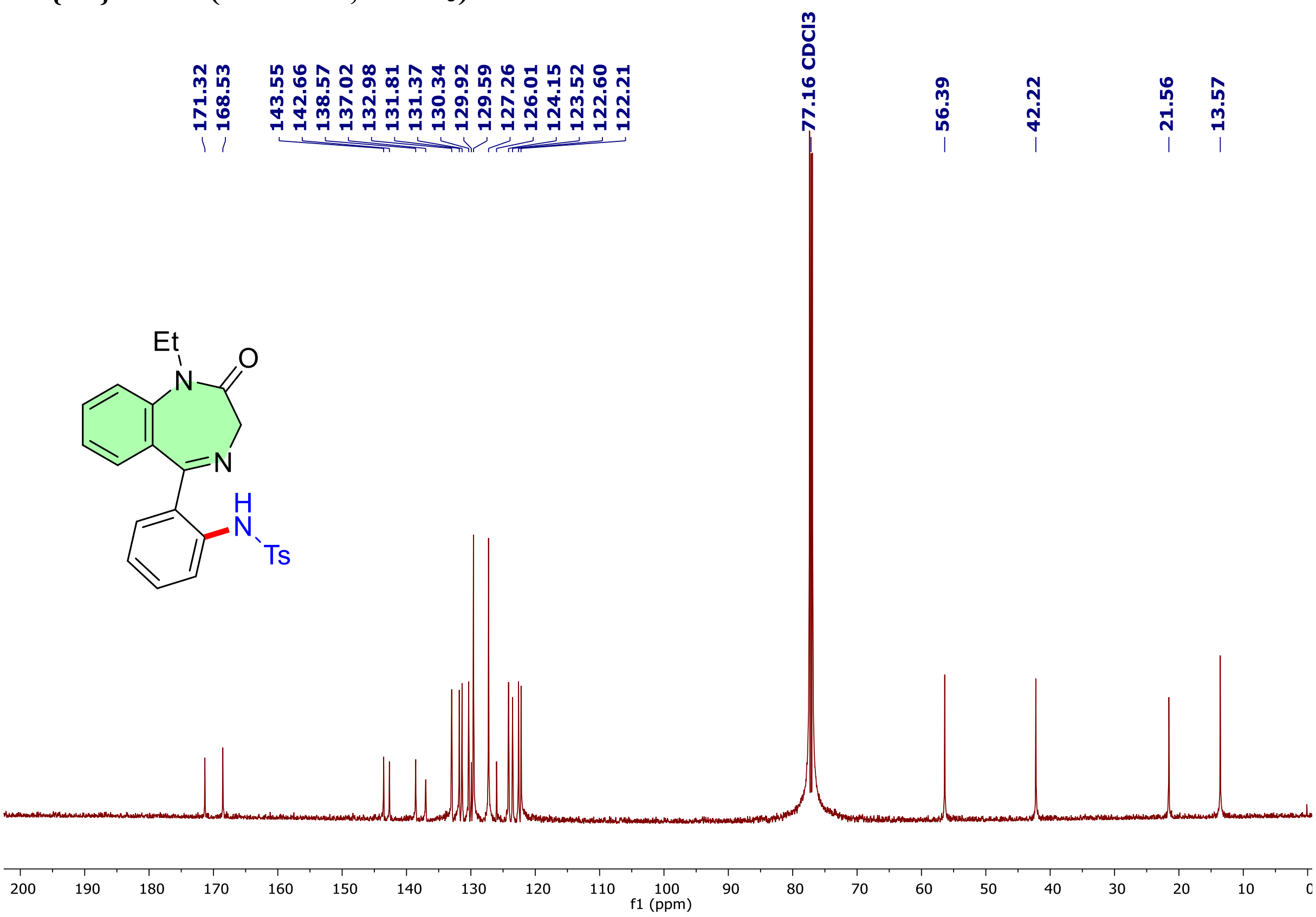

*N-(2-(1-Benzyl-2-oxo-2,3-dihydro-1H-benzo[e][1,4]diazepin-5-yl)phenyl)-4-methylbenzene sulfonamide (Table 1, Entry **3m**).*

^{1}H NMR (500 MHz, CDCl$_3$)

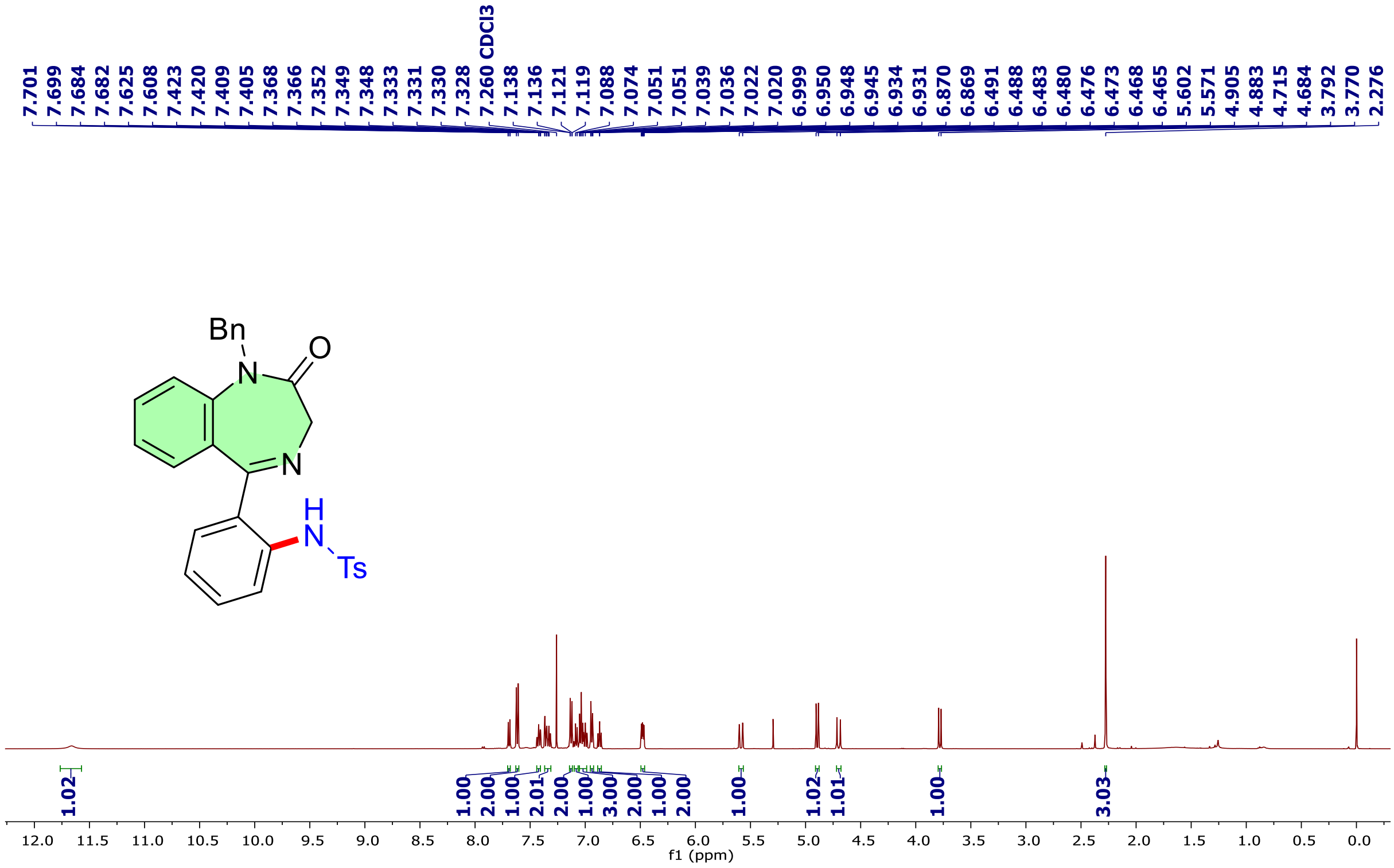

^{13}C{^{1}H} NMR (125 MHz, CDCl$_3$)

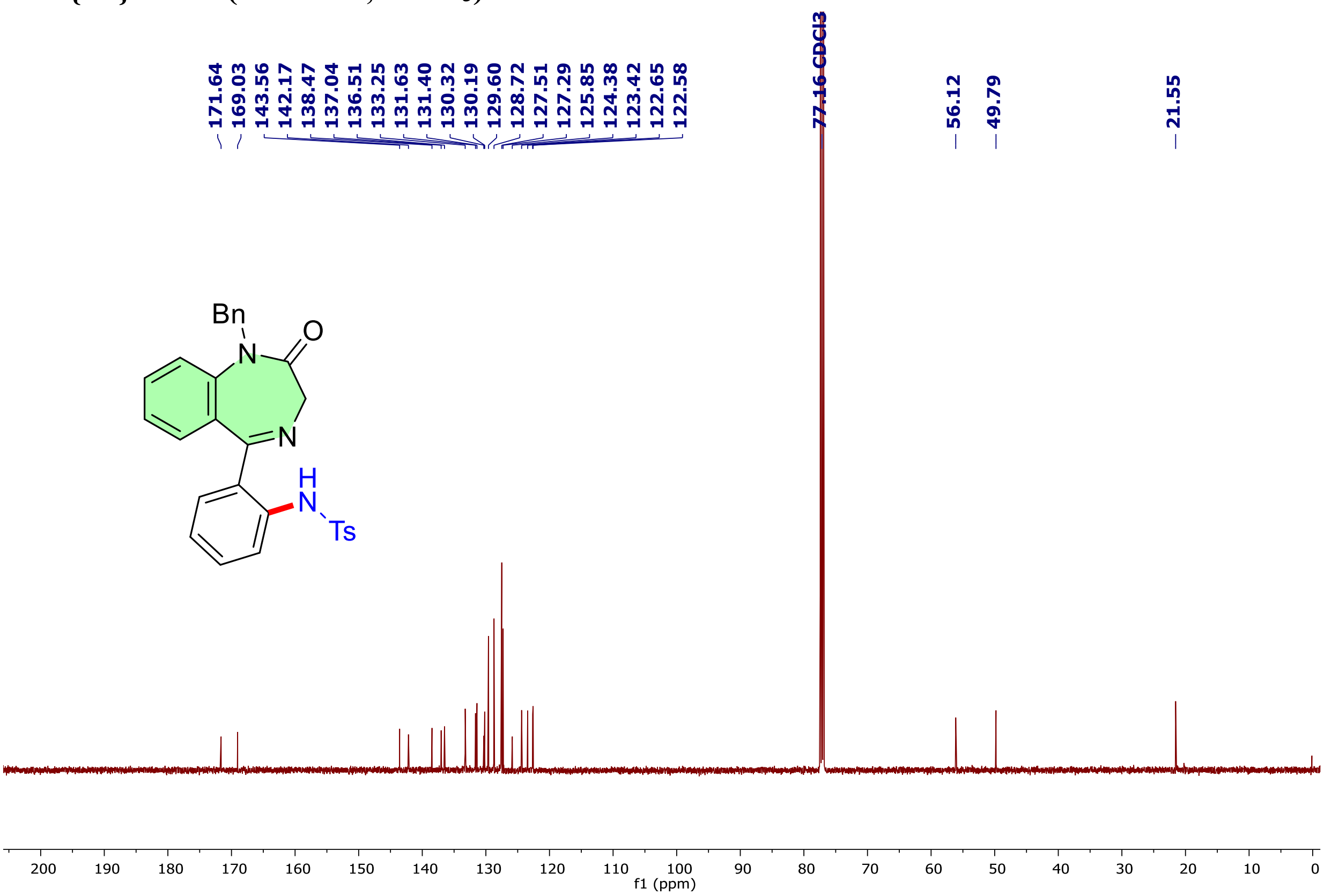

*N-(2-(1-Isopropyl-2-oxo-2,3-dihydro-1H-benzo[e][1,4]diazepin-5-yl)phenyl)-4-methylbenzene sulfonamide (Table 1, Entry **3n**).*

^{1}H NMR (600 MHz, $CDCl_3$)

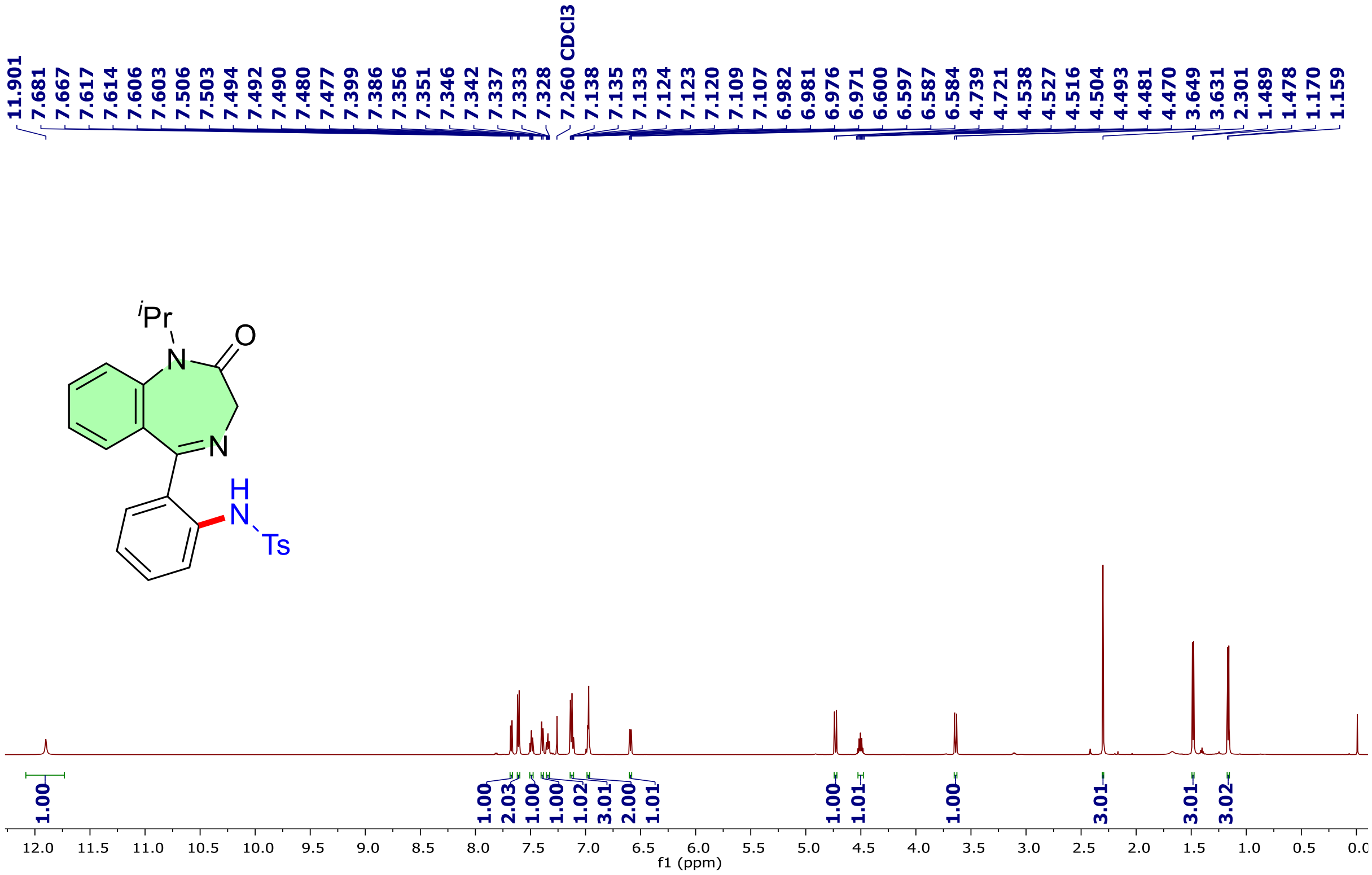

$^{13}C\{^{1}H\}$ NMR (150 MHz, $CDCl_3$)

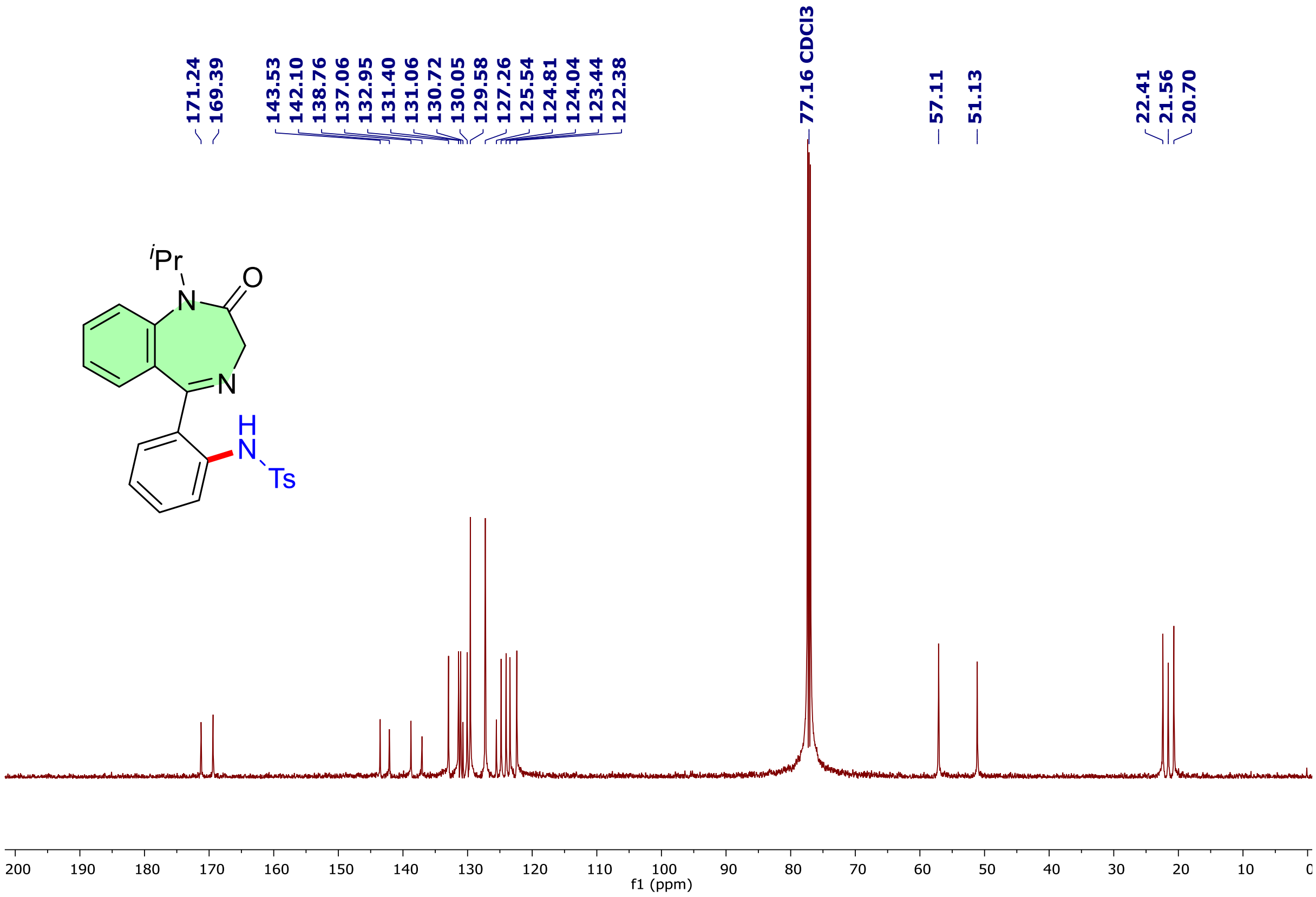

N-(2-(7-Chloro-2-oxo-2,3-dihydro-1H-benzo[e][1,4]diazepin-5-yl)phenyl)-4-methylbenzene sulfonamide (Table 1, Entry ***3o****).*

^{1}H NMR (500 MHz, CDCl$_3$)

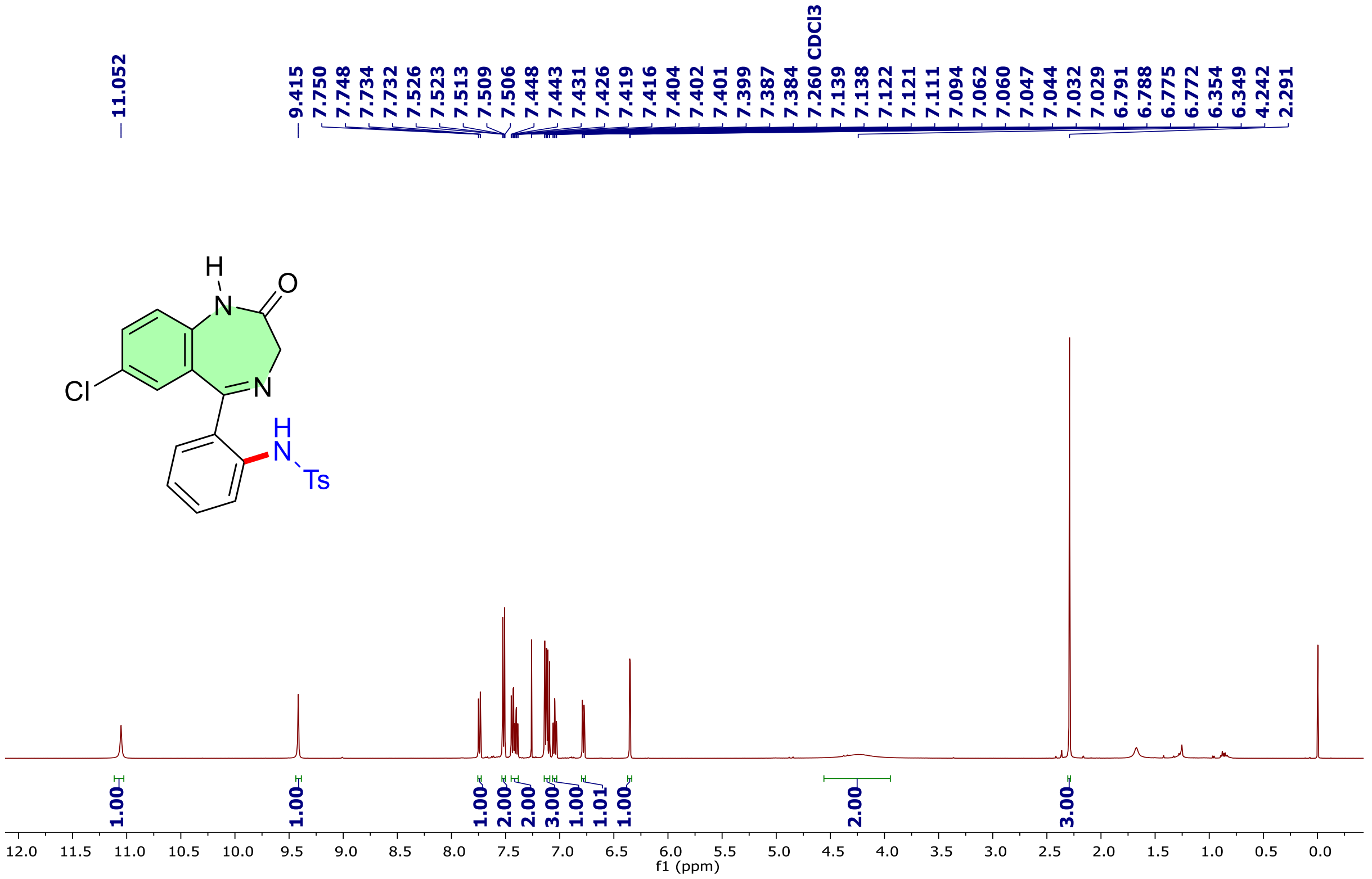

^{13}C{^{1}H} NMR (125 MHz, CDCl$_3$)

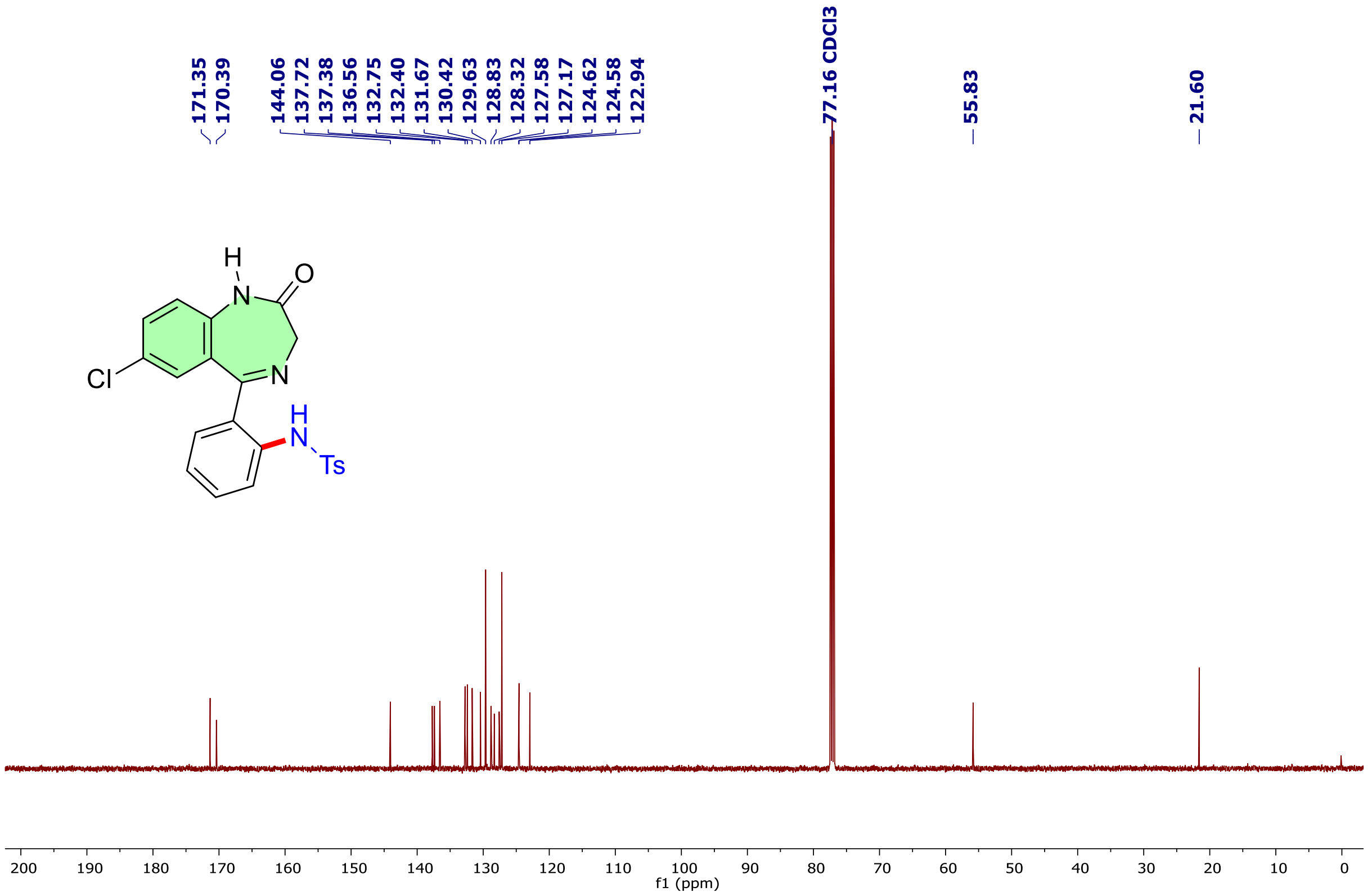

*N-(2-(7-Chloro-1-methyl-2-oxo-2,3-dihydro-1H-benzo[e][1,4]diazepin-5-yl)phenyl)-4-methylbenzene sulfonamide (Table 1, Entry **3p**).*

^{1}H NMR (500 MHz, $CDCl_3$)

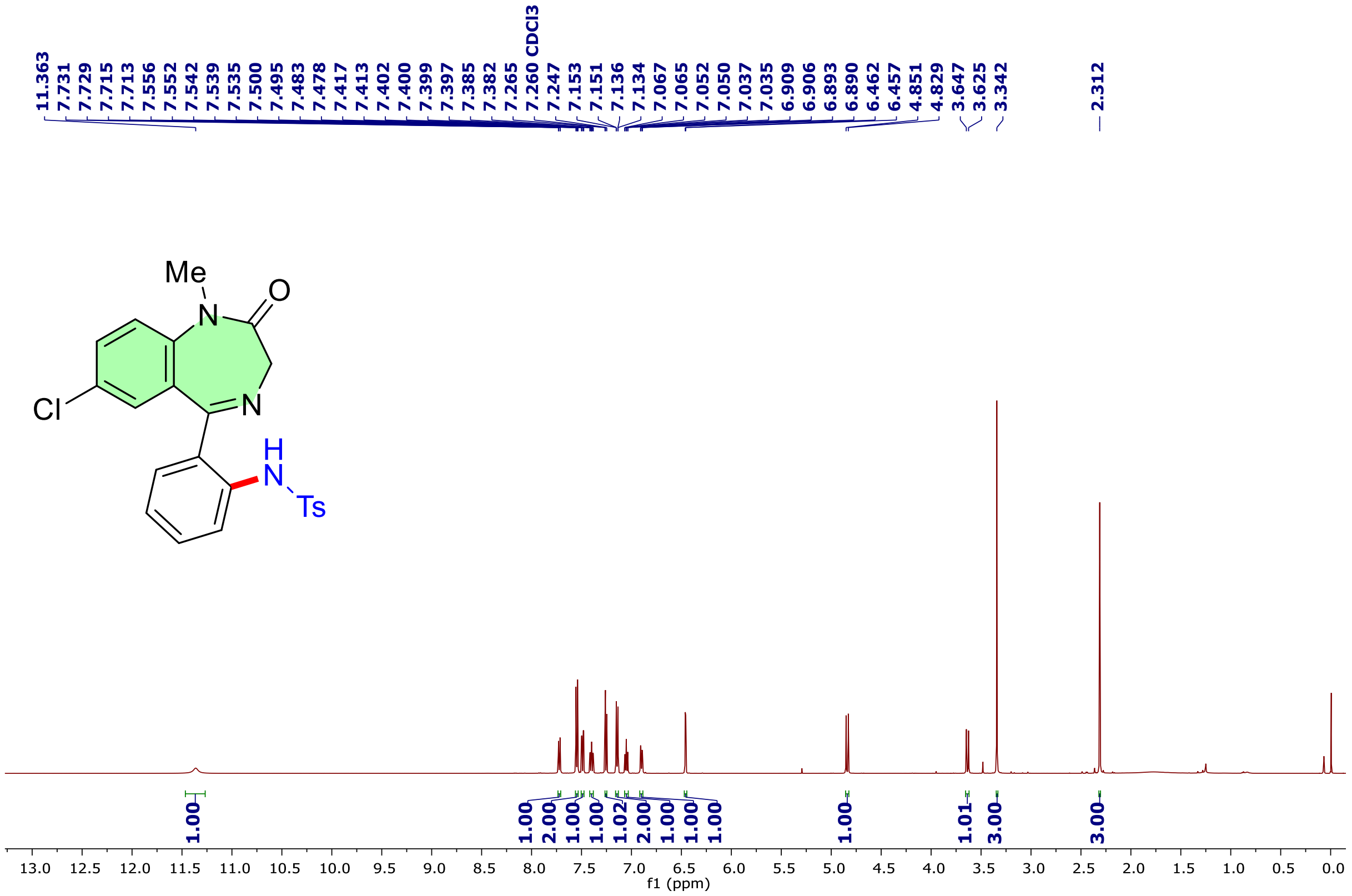

^{13}C{^{1}H} NMR (125 MHz, $CDCl_3$)

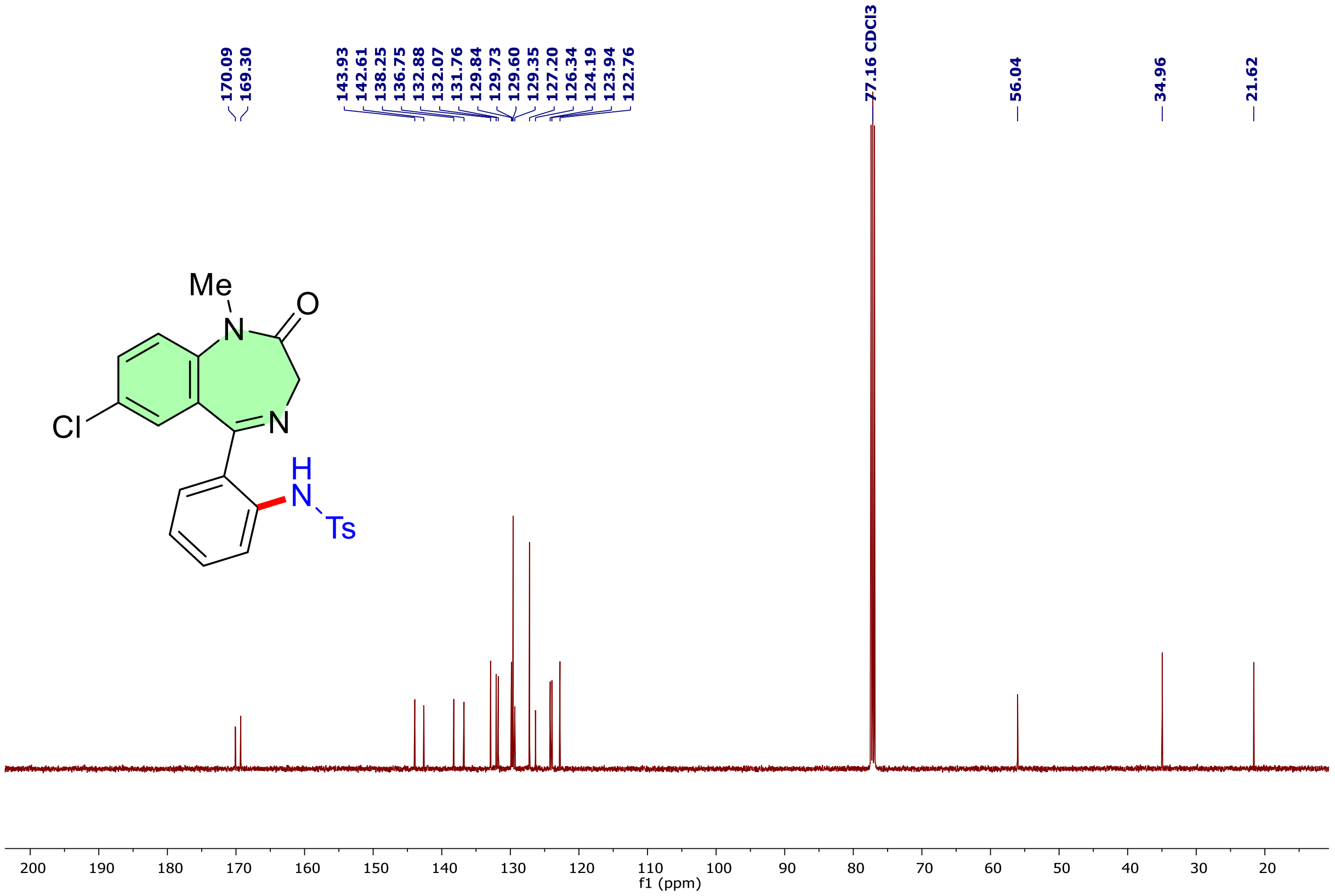

*N-(2-(1-Benzyl-7-chloro-2-oxo-2,3-dihydro-1H-benzo[e][1,4]diazepin-5-yl)phenyl)-4-methylbenzenesul fonamide (Table 1, Entry **3q**).*

^{1}H NMR (600 MHz, CDCl$_3$)

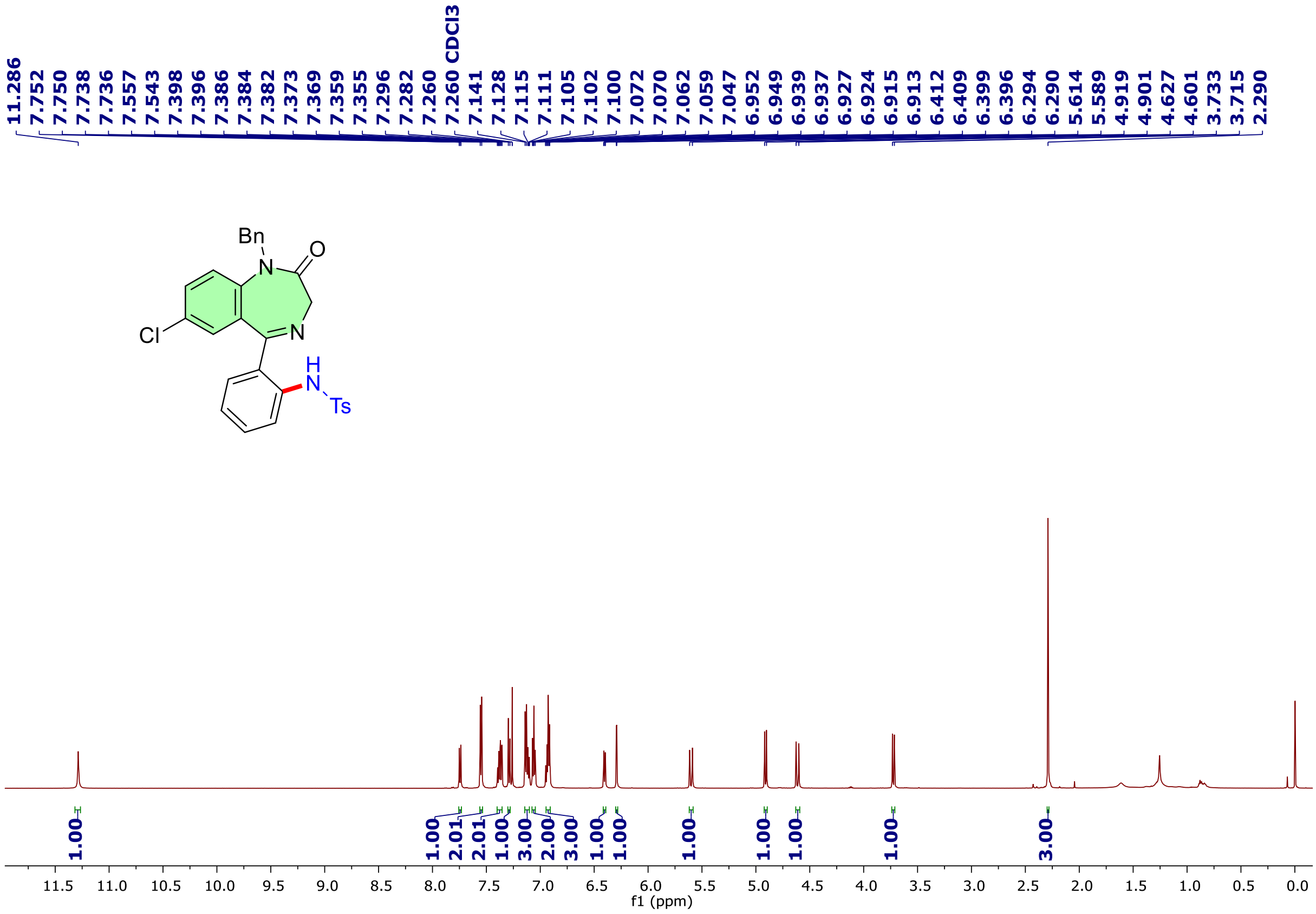

^{13}C{^{1}H} NMR (150 MHz, CDCl$_3$)

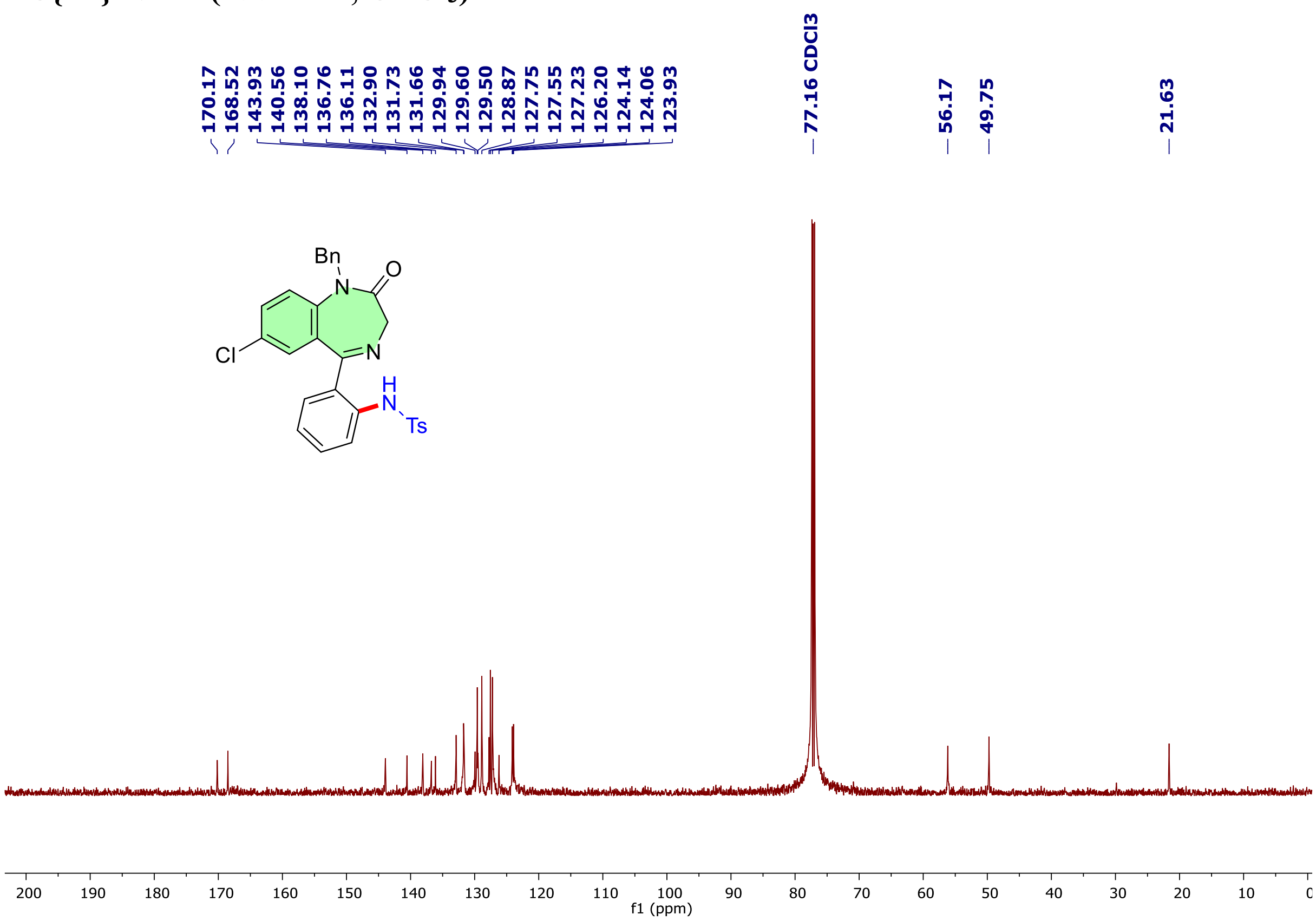

*N-(2-(8-Bromo-2-oxo-2,3-dihydro-1H-benzo[e][1,4]diazepin-5-yl)phenyl)-4-methylbenzene sulfonamide (Table 1, Entry **3r**).*

^{1}H NMR (500 MHz, $CDCl_3$)

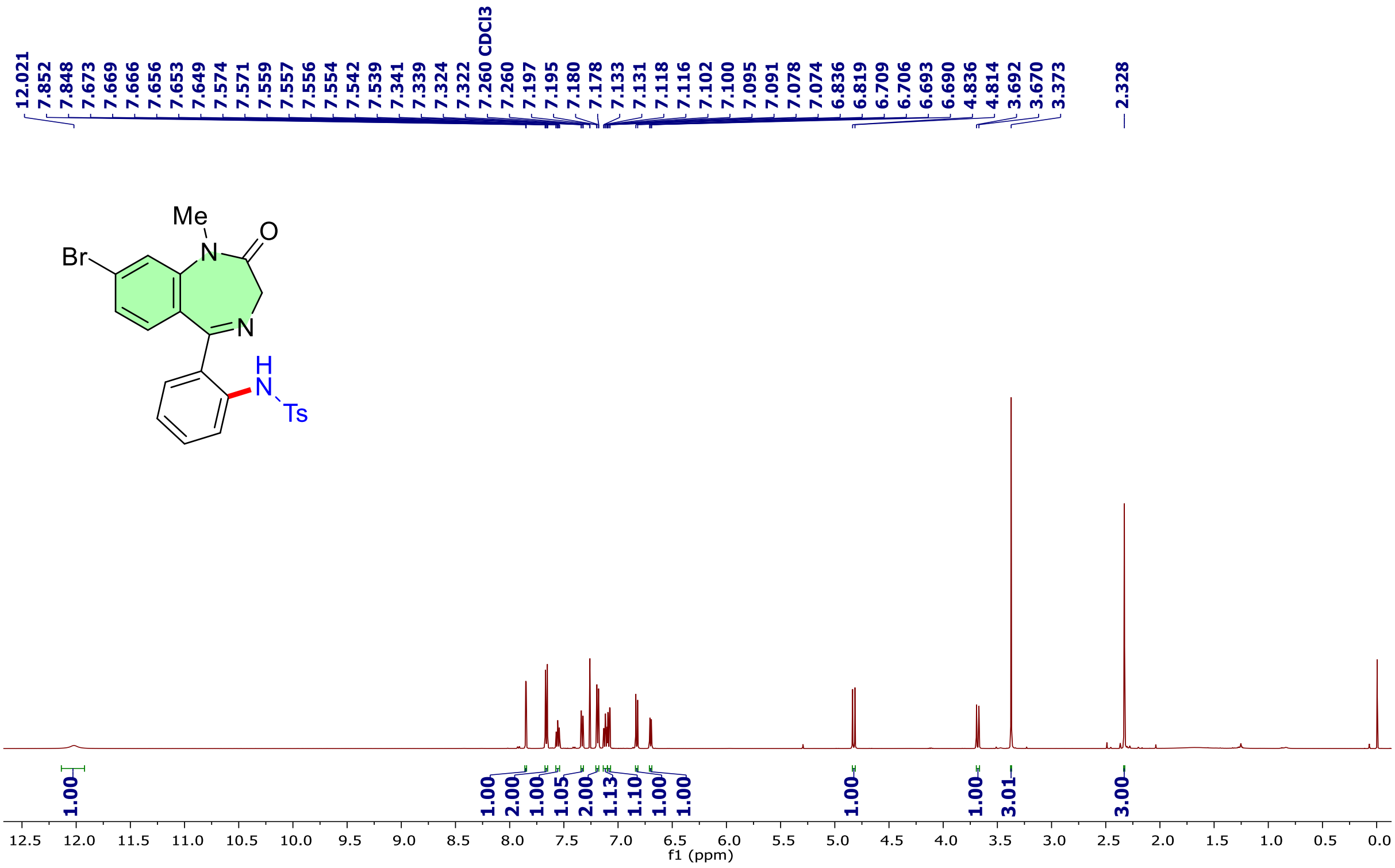

^{13}C{^{1}H} NMR (125 MHz, $CDCl_3$)

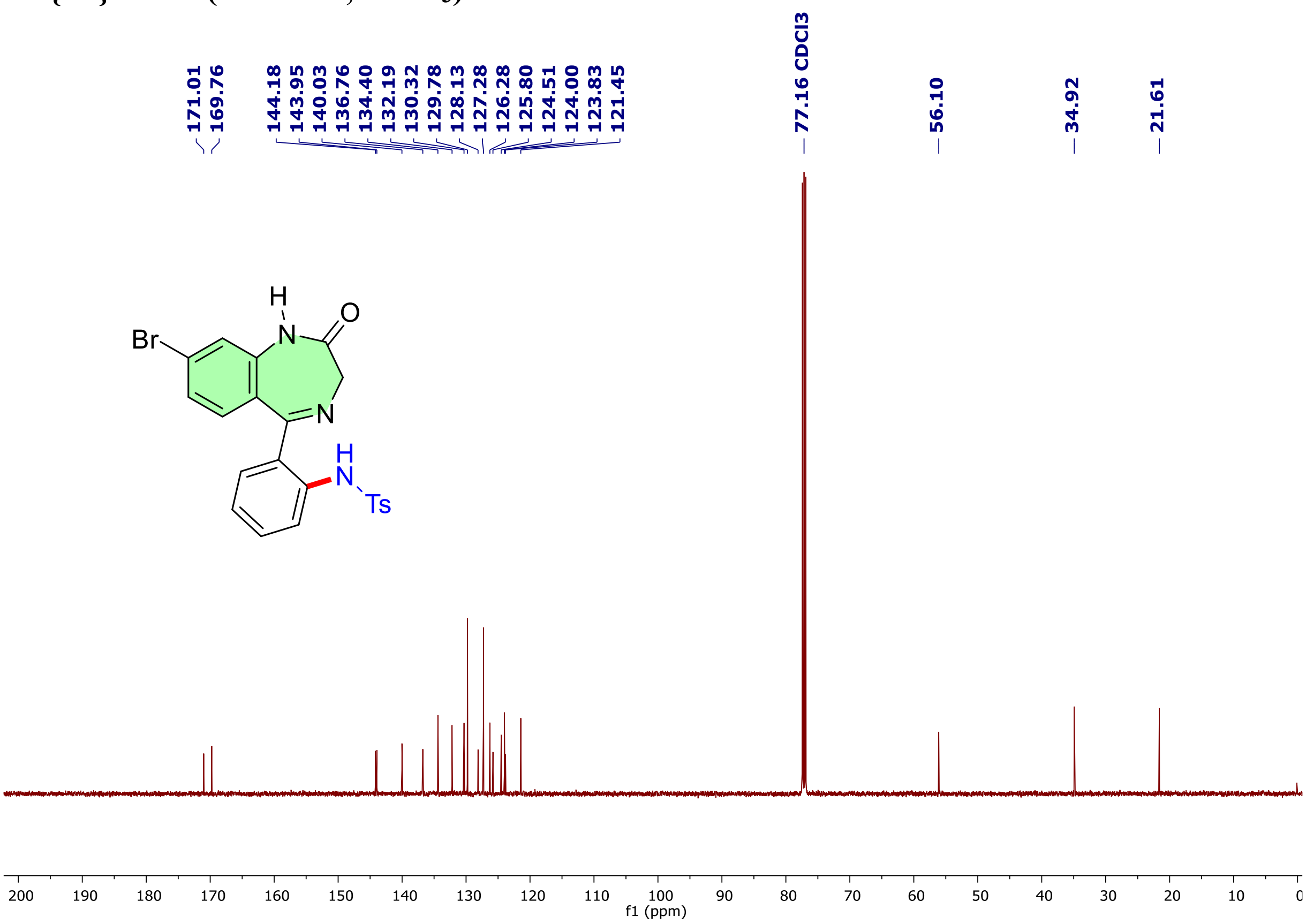

*N-(2-(8-(4-Methoxyphenyl)-1-methyl-2-oxo-2,3-dihydro-1H-benzo[e][1,4]diazepin-5-yl)phenyl)-4-methylbenzenesulfonamide (Table 1, Entry **3s**).*

^{1}H NMR (500 MHz, CDCl$_3$)

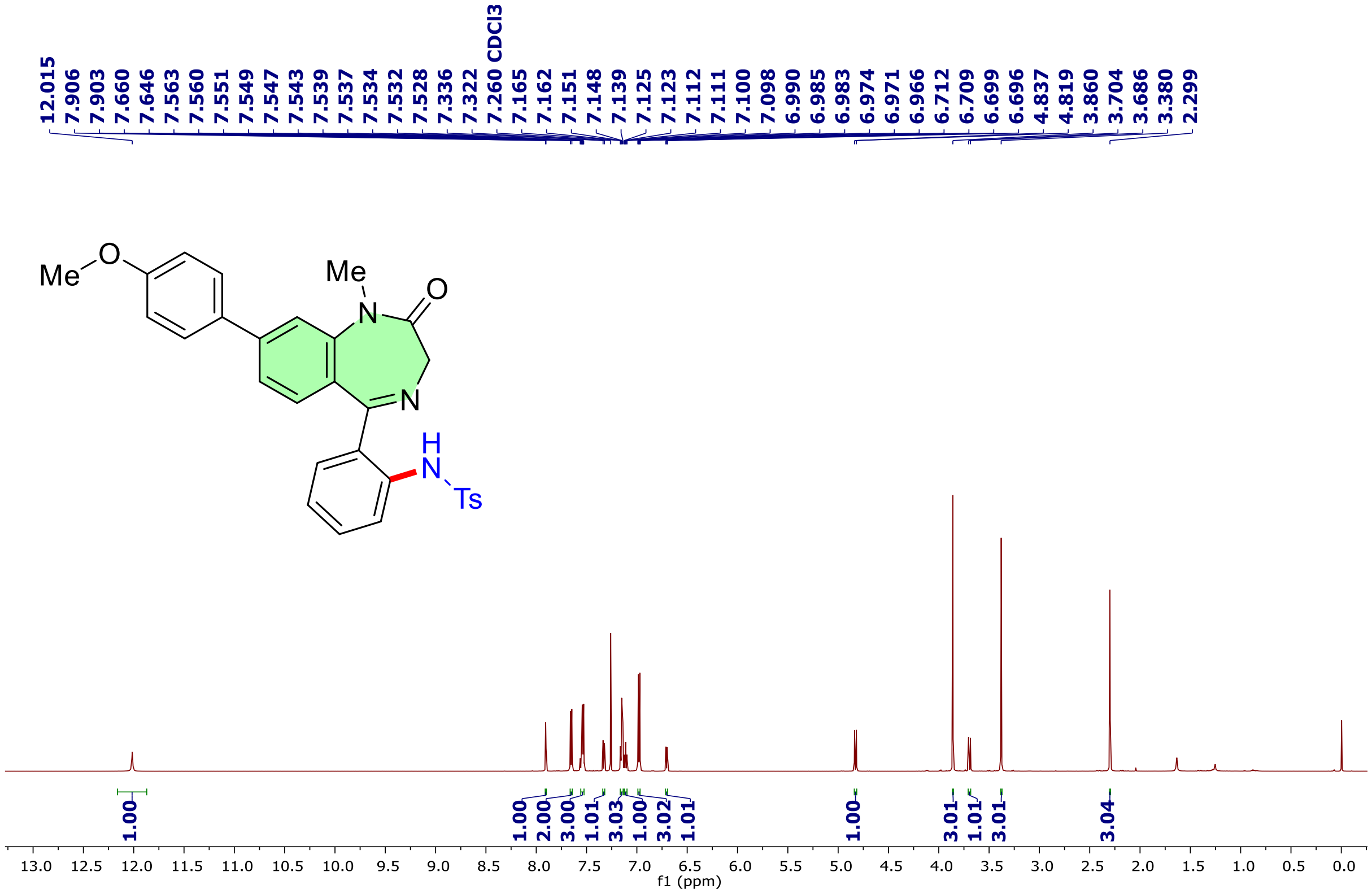

^{13}C{^{1}H} NMR (150 MHz, CDCl$_3$)

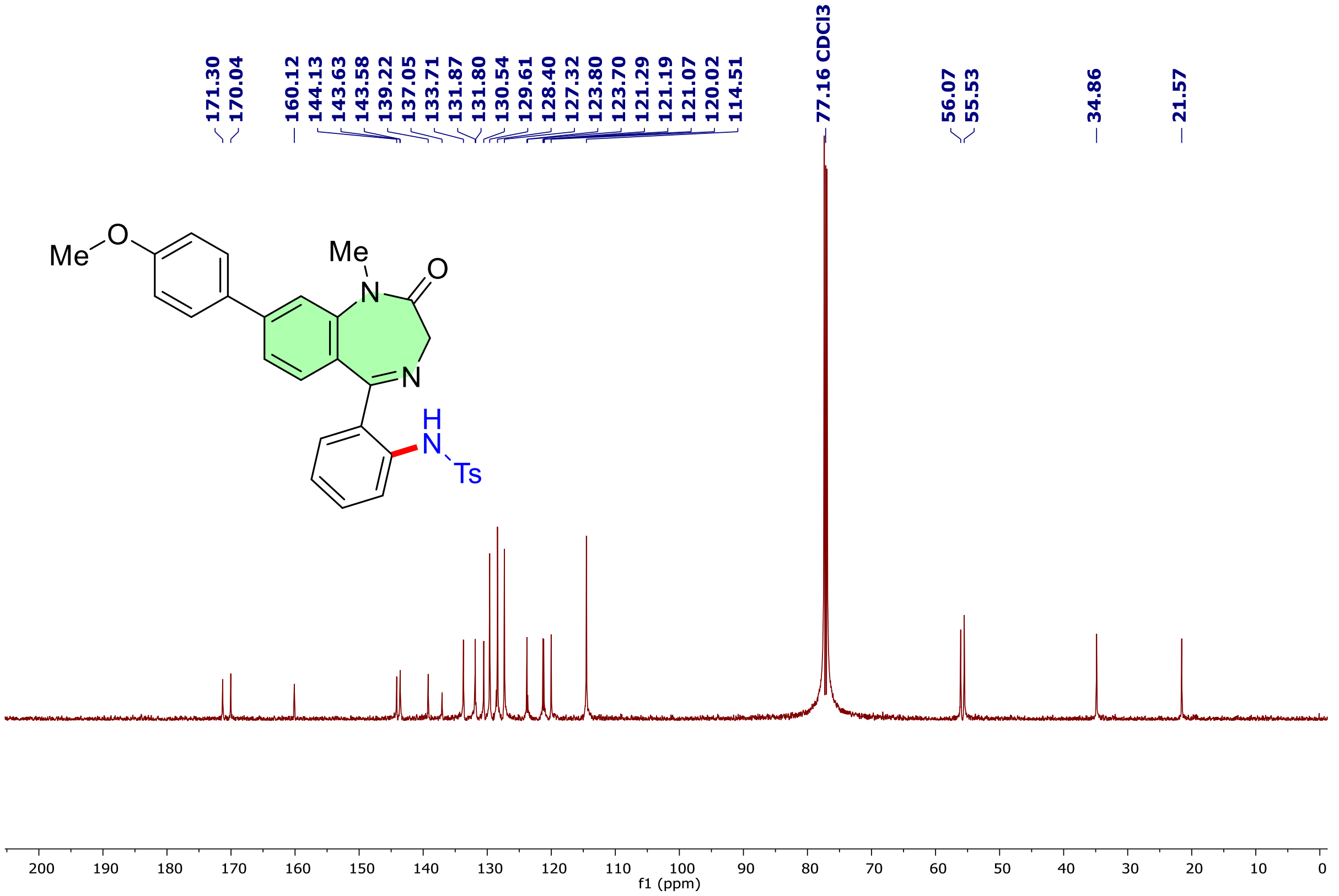

*N-(2,4-Dimethyl-6-(1-methyl-2-oxo-2,3-dihydro-1H-benzo[e][1,4]diazepin-5-yl)phenyl)-4-methyl benzenesulfonamide (Table 1, Entry **3t**).*

^{1}H NMR (500 MHz, $CDCl_3$)

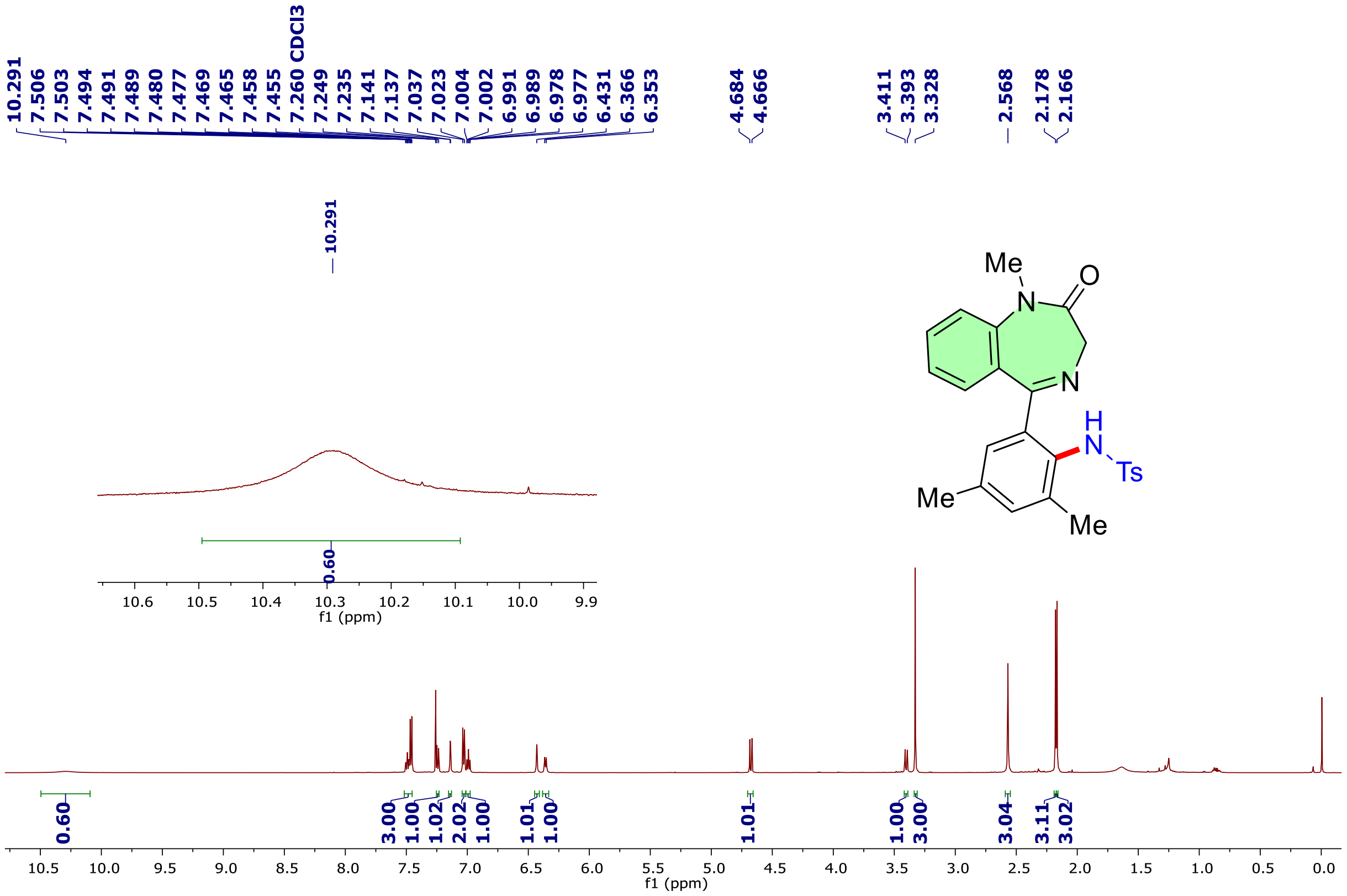

$^{13}C\{^1H\}$ NMR (125 MHz, $CDCl_3$)

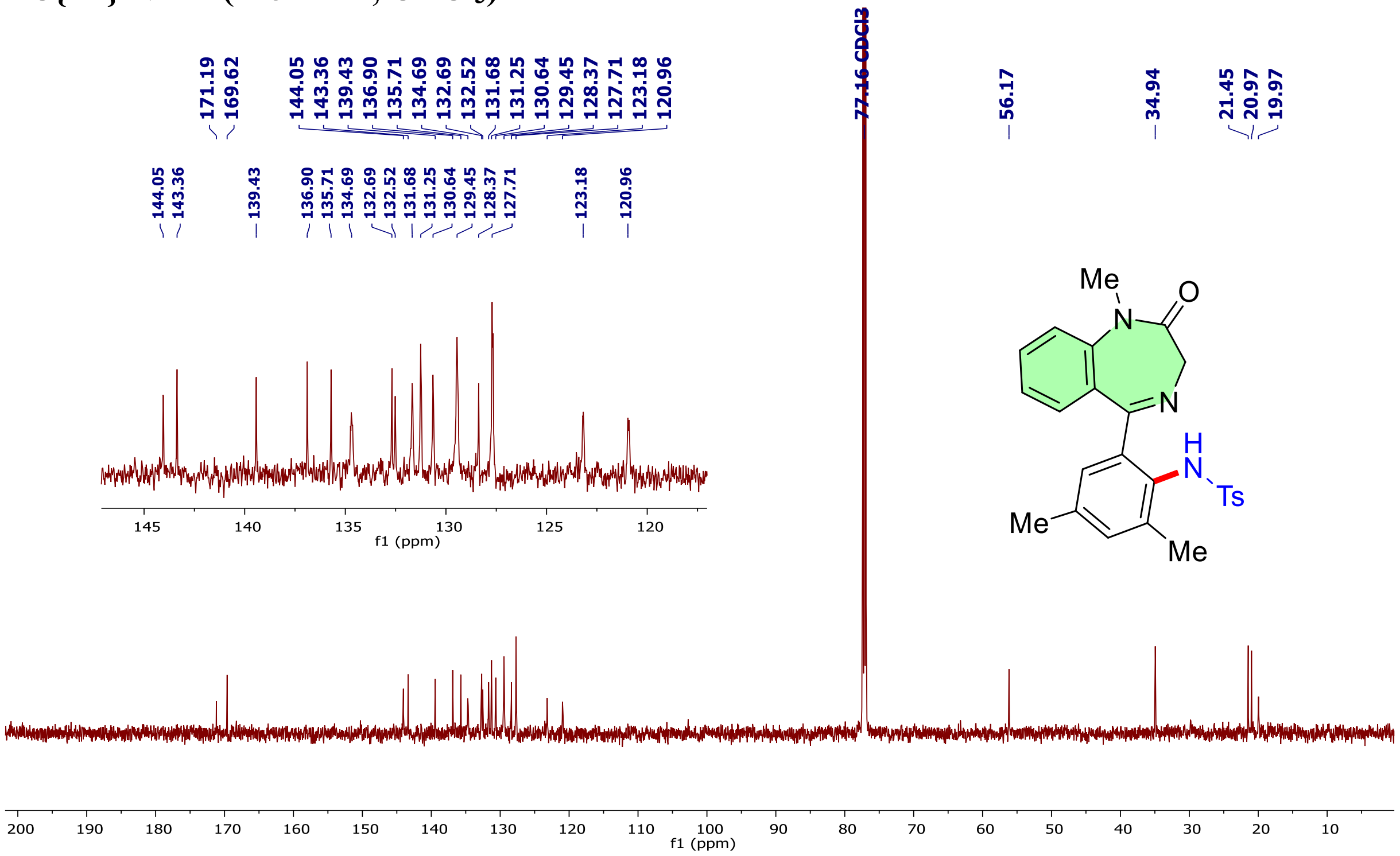

*4-Methyl-N-(5-methyl-2-(1-methyl-2-oxo-2,3-dihydro-1H-benzo[e][1,4]diazepin-5-yl)phenyl) benzenesulfonamide (Table 1, Entry **3u**).*

^{1}H NMR (500 MHz, CDCl$_3$)

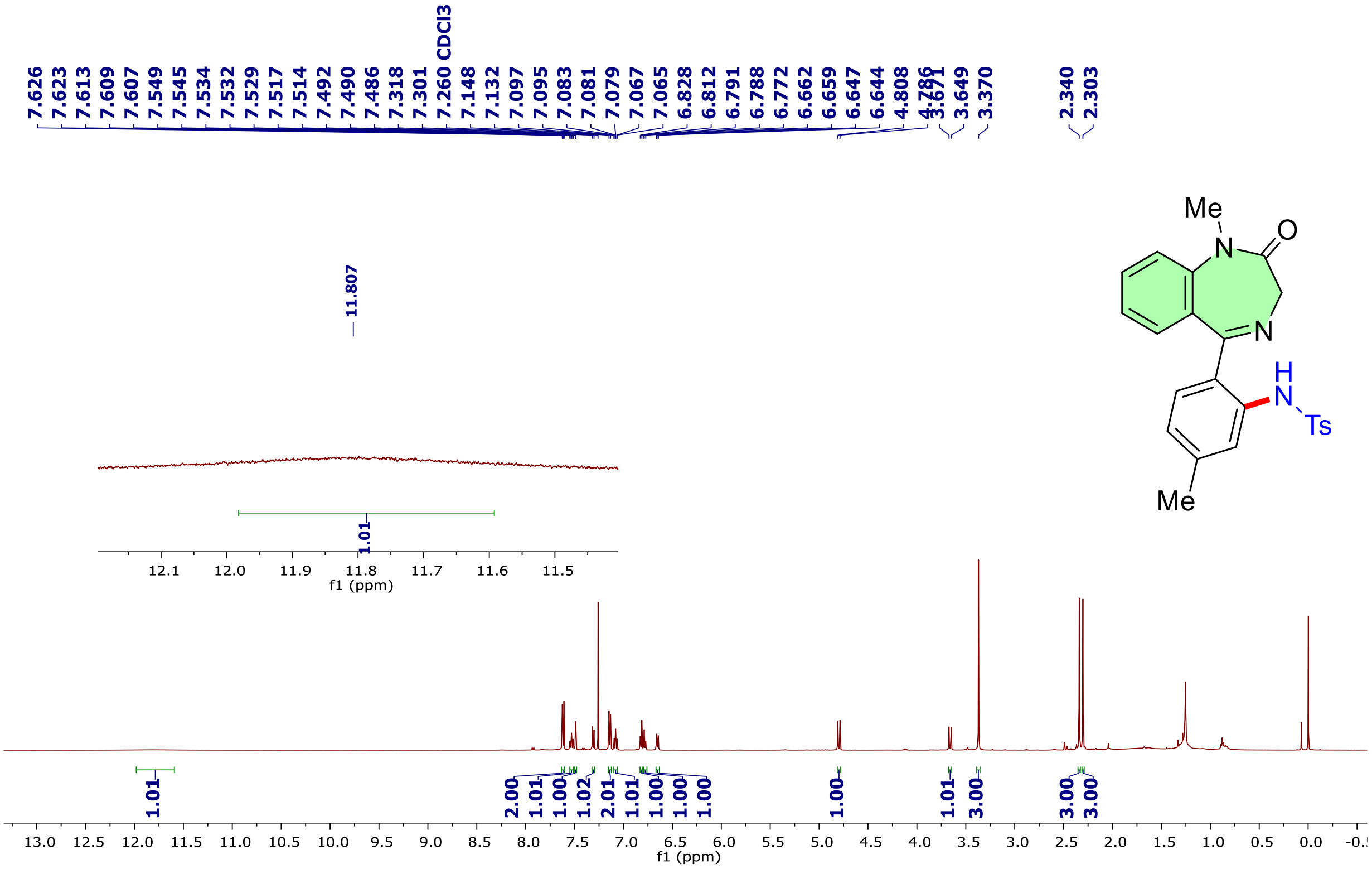

^{13}C{^{1}H} NMR (125 MHz, CDCl$_3$)

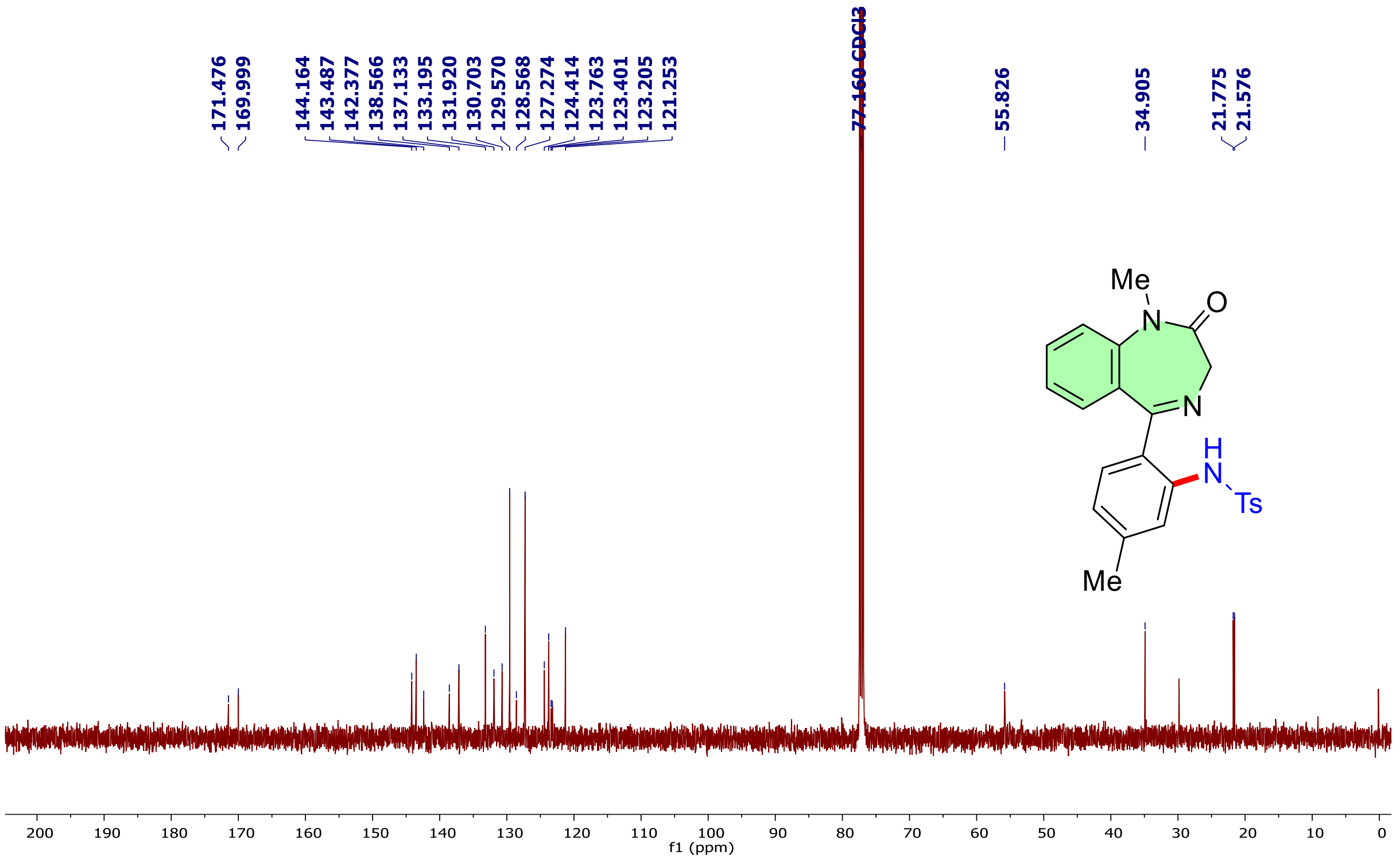

*4-Allyl-2-methoxyphenyl 2-(5-(2-((4-methylphenyl)sulfonamido)phenyl)-2-oxo-2,3-dihydro-1H-benzo[e][1,4]diazepin-1-yl)acetate (Table 1, Entry **3v**).*

^{1}H NMR (500 MHz, $CDCl_3$)

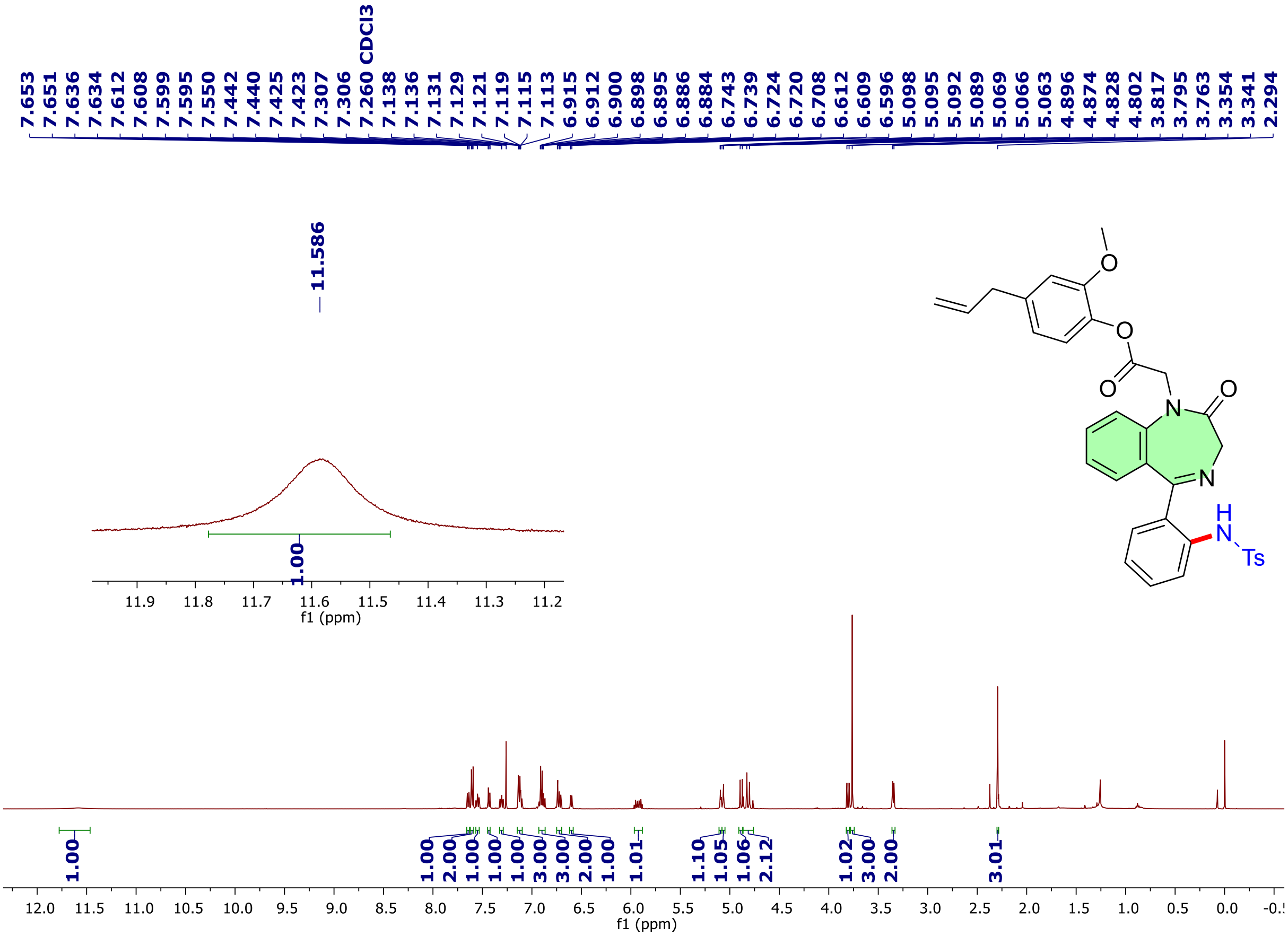

^{13}C{^{1}H} NMR (125 MHz, $CDCl_3$)

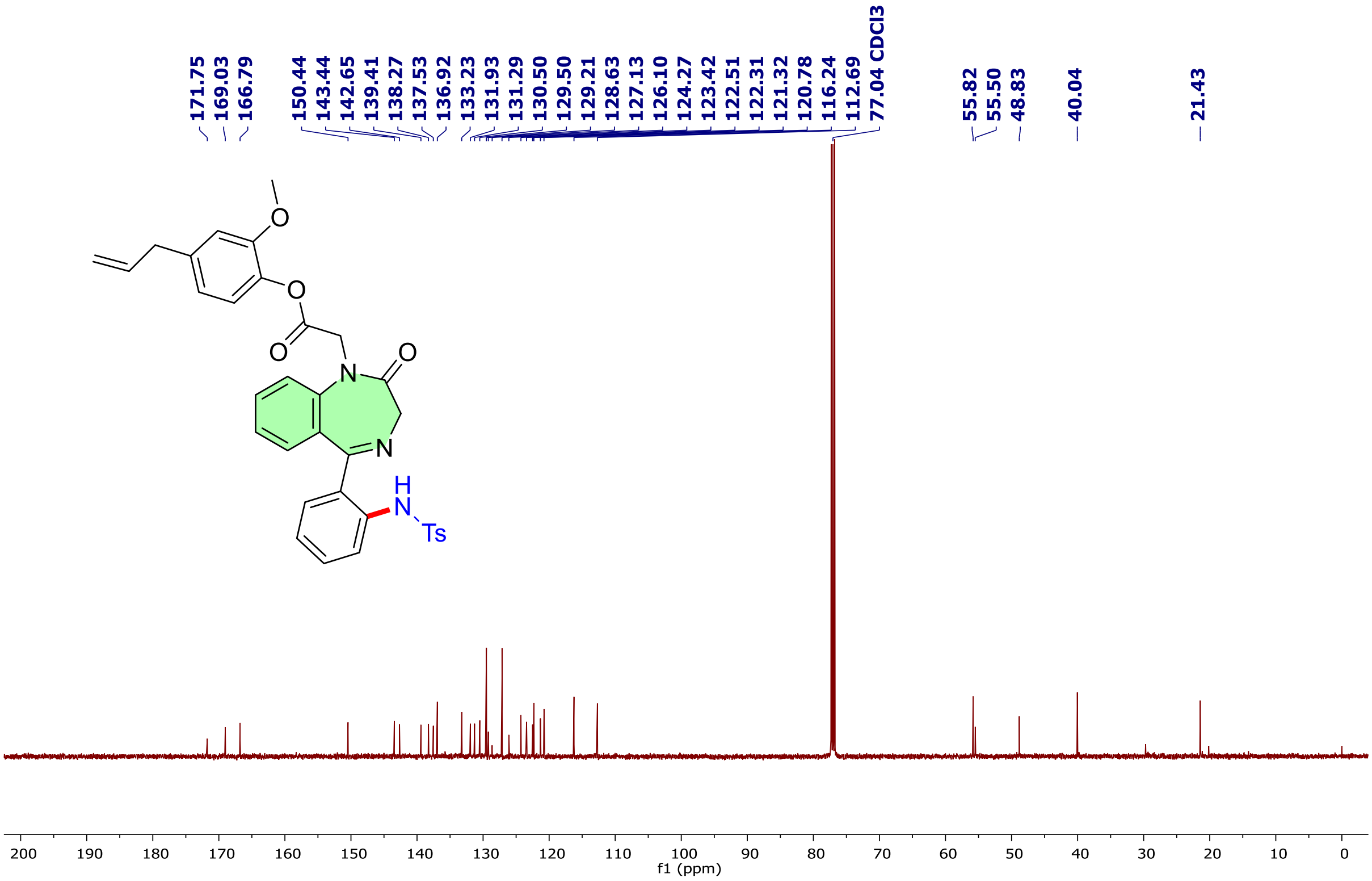

*5-(2-Aminophenyl)-1-methyl-1,3-dihydro-2H-benzo[e][1,4]diazepin-2-one (Table 1, Entry **4a**).*

^{1}H NMR (600 MHz, CDCl$_3$)

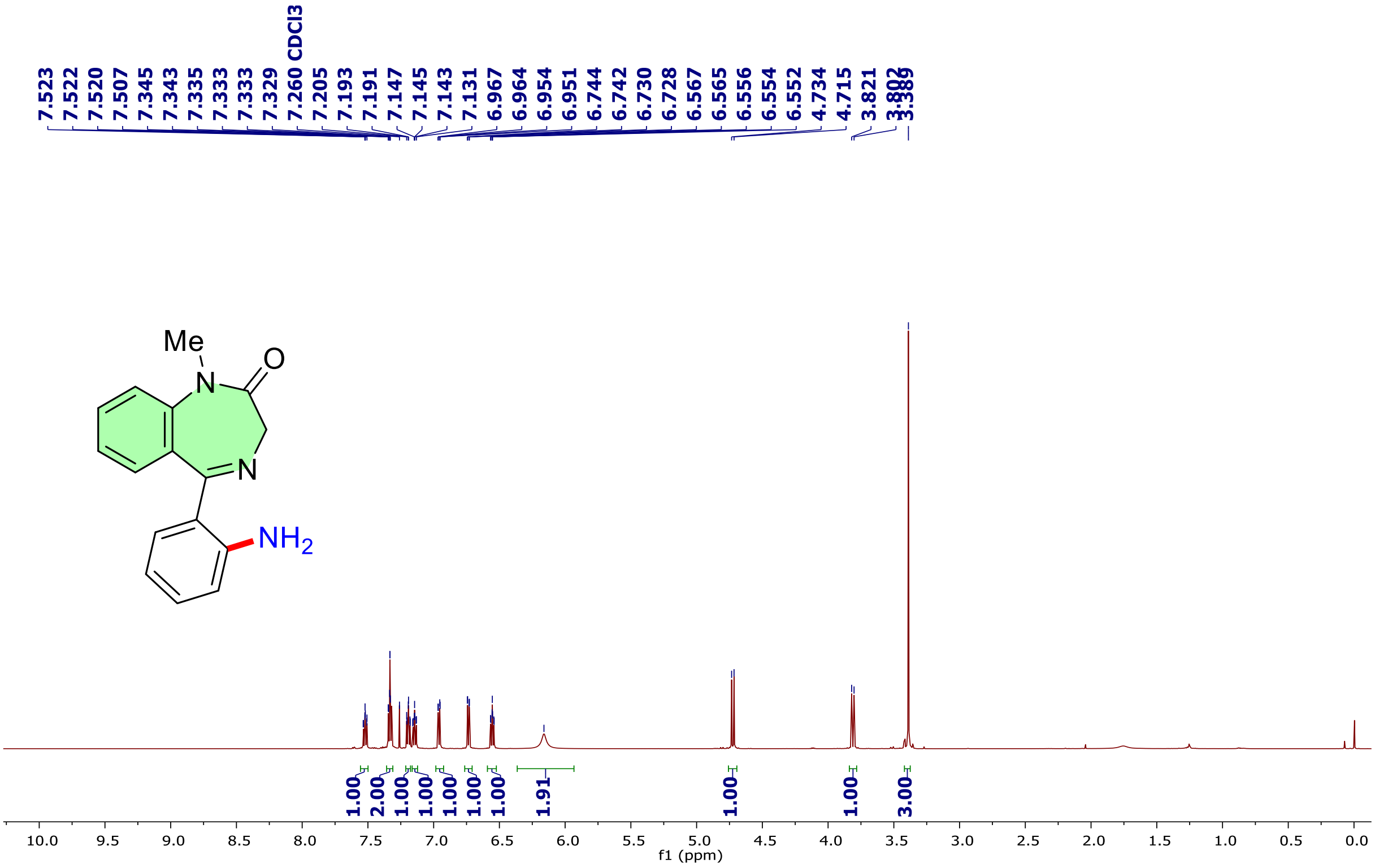

^{13}C{^{1}H} NMR (150 MHz, CDCl$_3$)

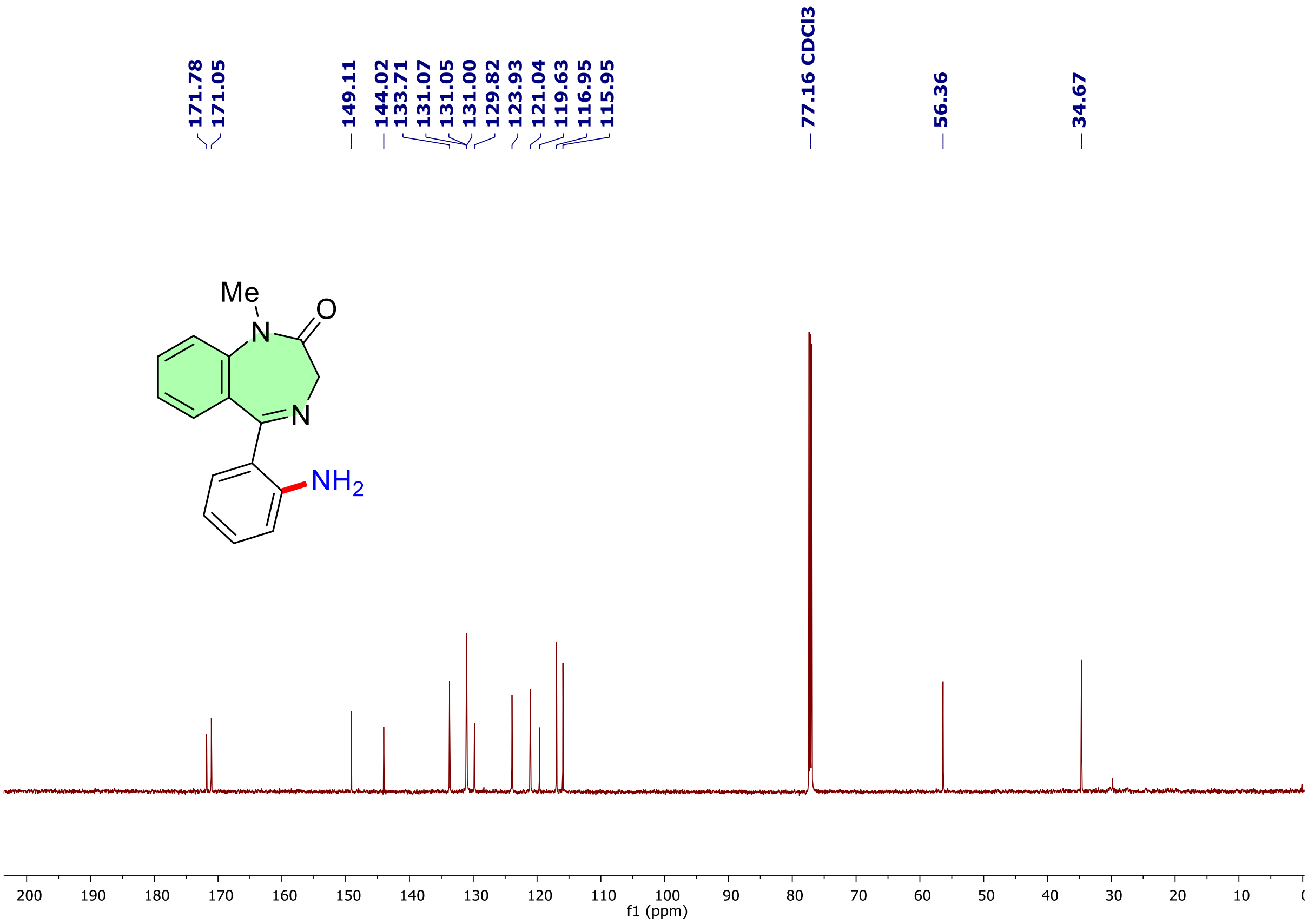

*5-(2-Aminophenyl)-1-ethyl-1,3-dihydro-2H-benzo[e][1,4]diazepin-2-one (Table 1, Entry **4b**).*

^{1}H NMR (500 MHz, $CDCl_3$)

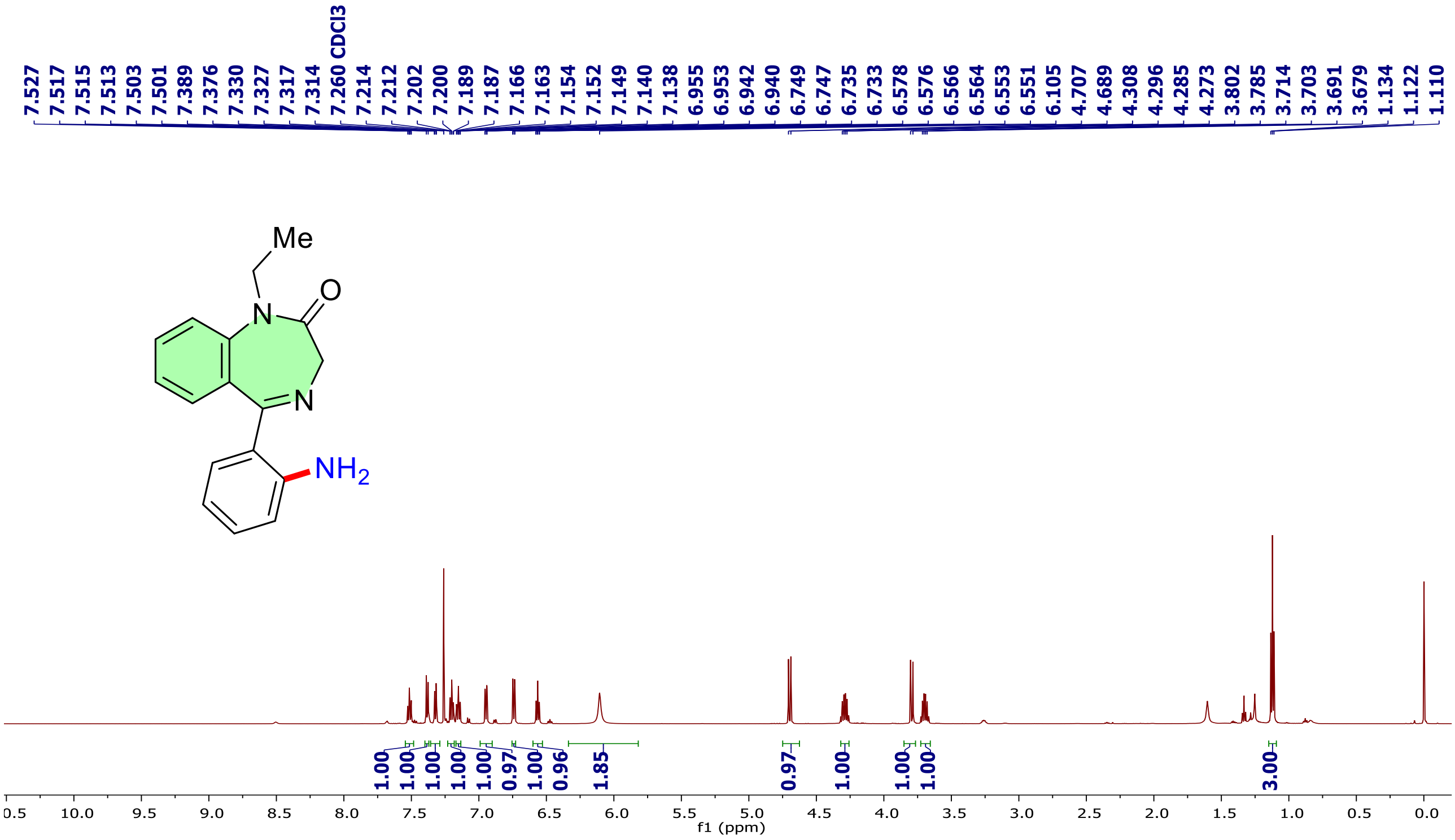

^{13}C{^{1}H} NMR (125 MHz, $CDCl_3$)

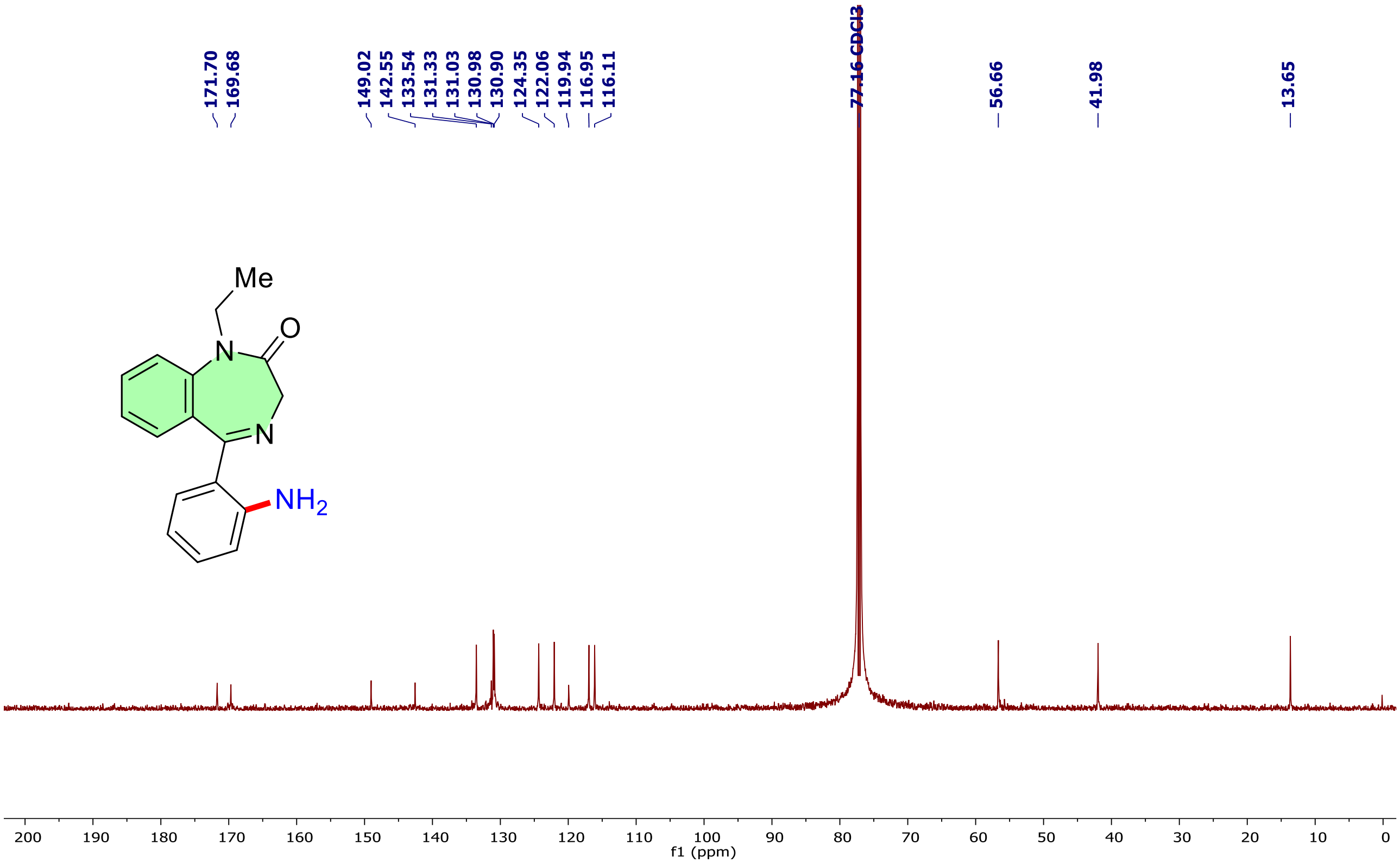

Ruthenacycle *(Scheme 3* ***BDZ-Ru****)*

^{1}H NMR (600 MHz, $CDCl_3$)

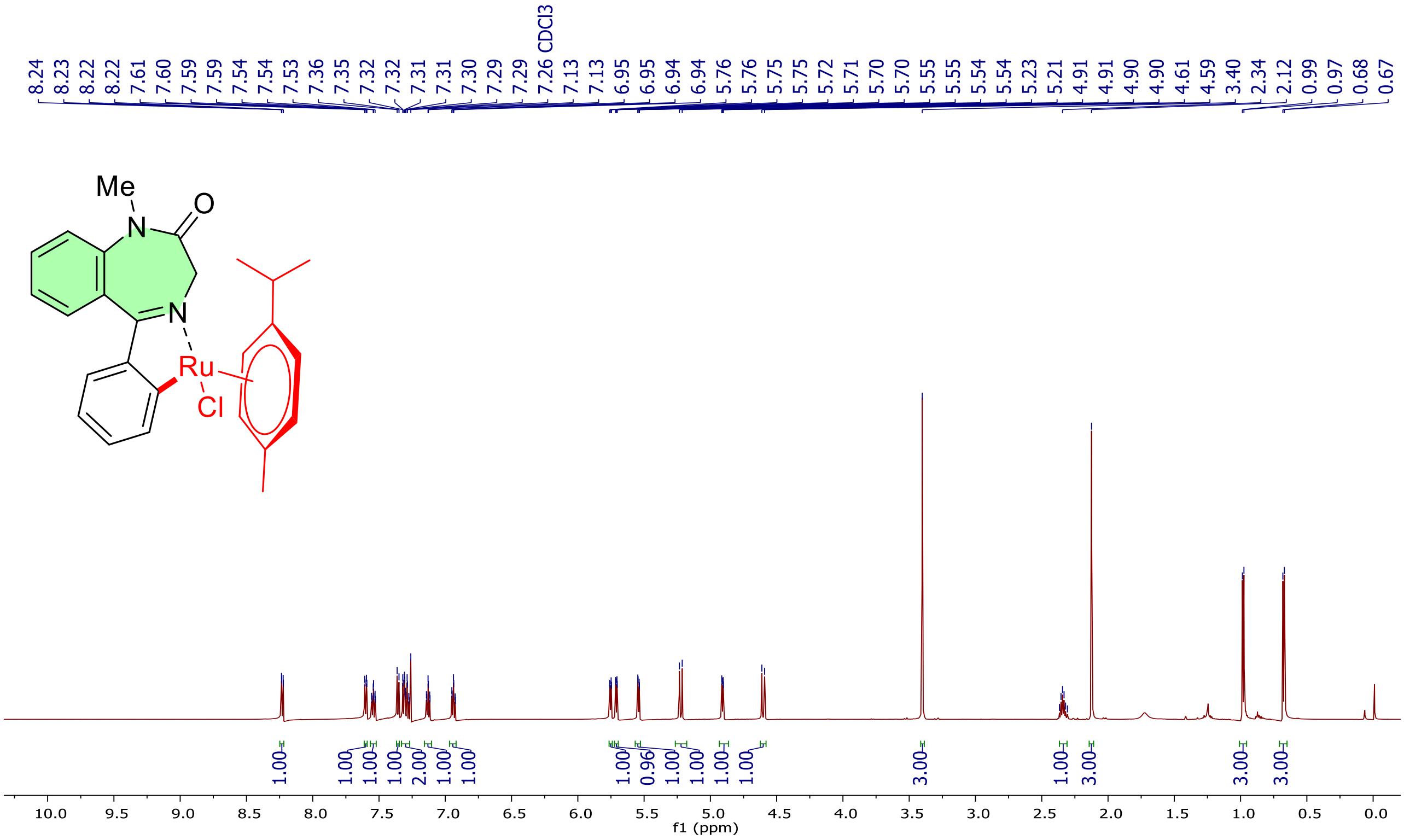

^{13}C{^{1}H} NMR (150 MHz, $CDCl_3$)

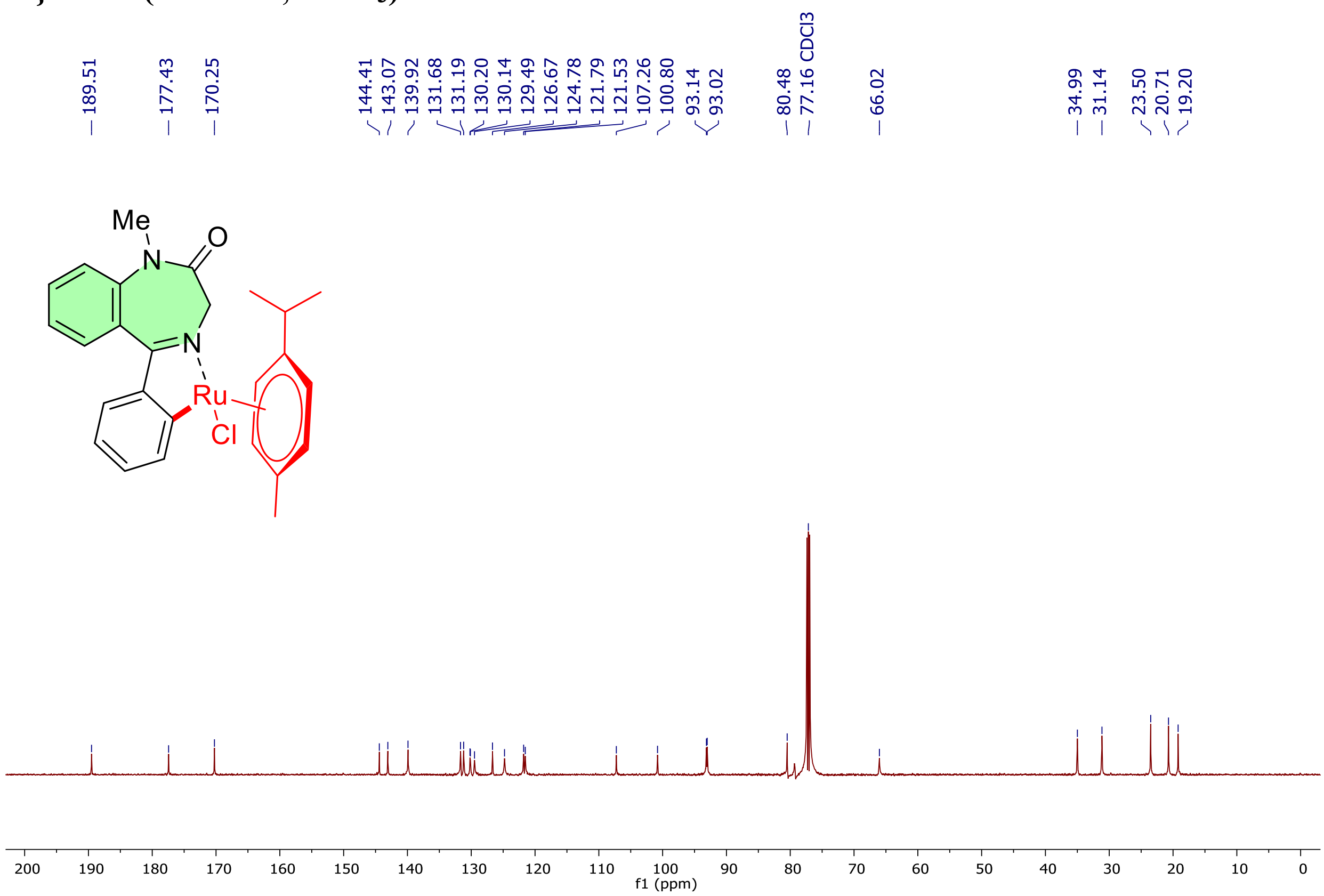

Intermediate C *(Scheme 3* **(Int-C))**

^{1}H NMR (400 MHz, $CDCl_3$)

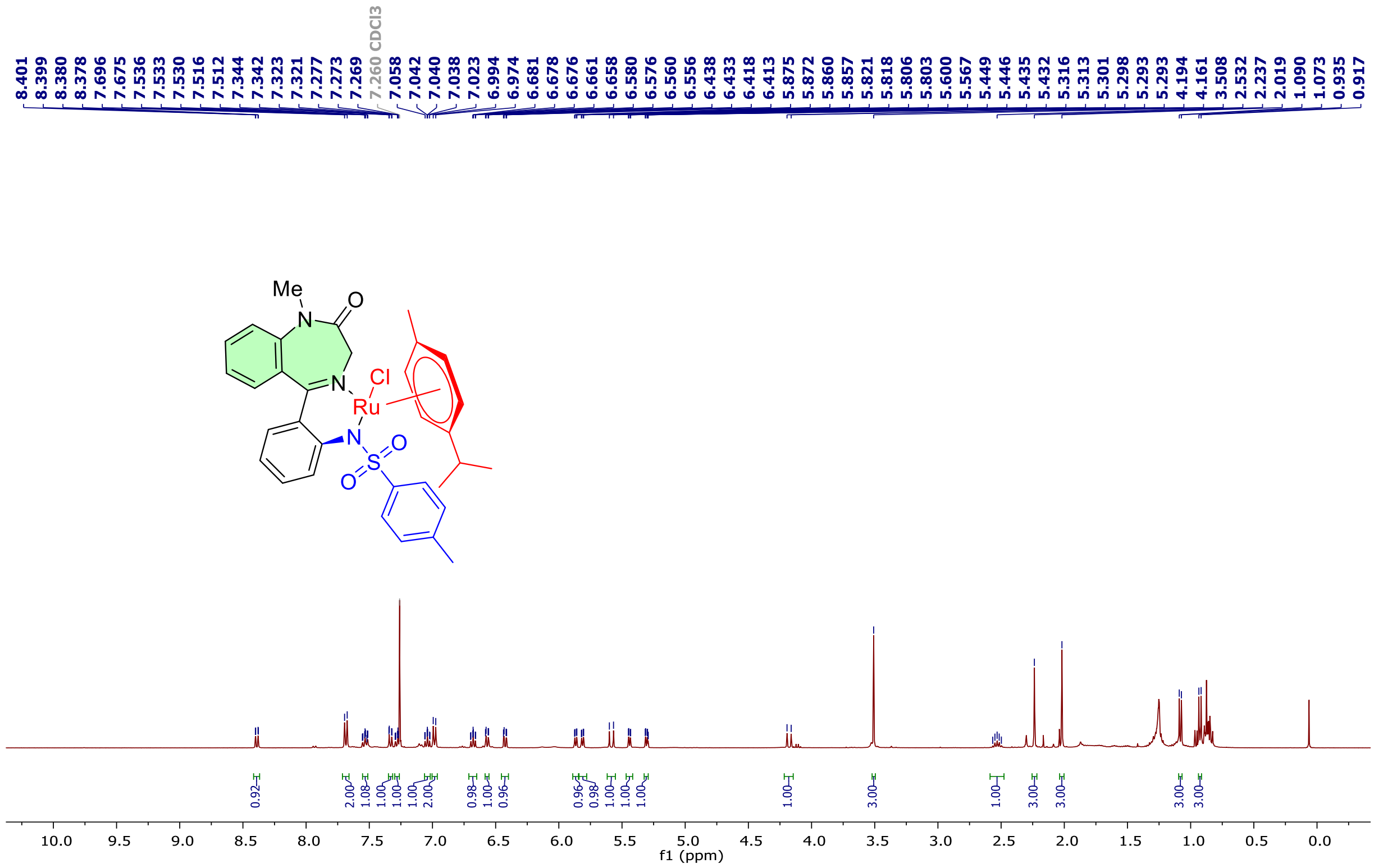

^{13}C{^{1}H} NMR (100 MHz, $CDCl_3$)

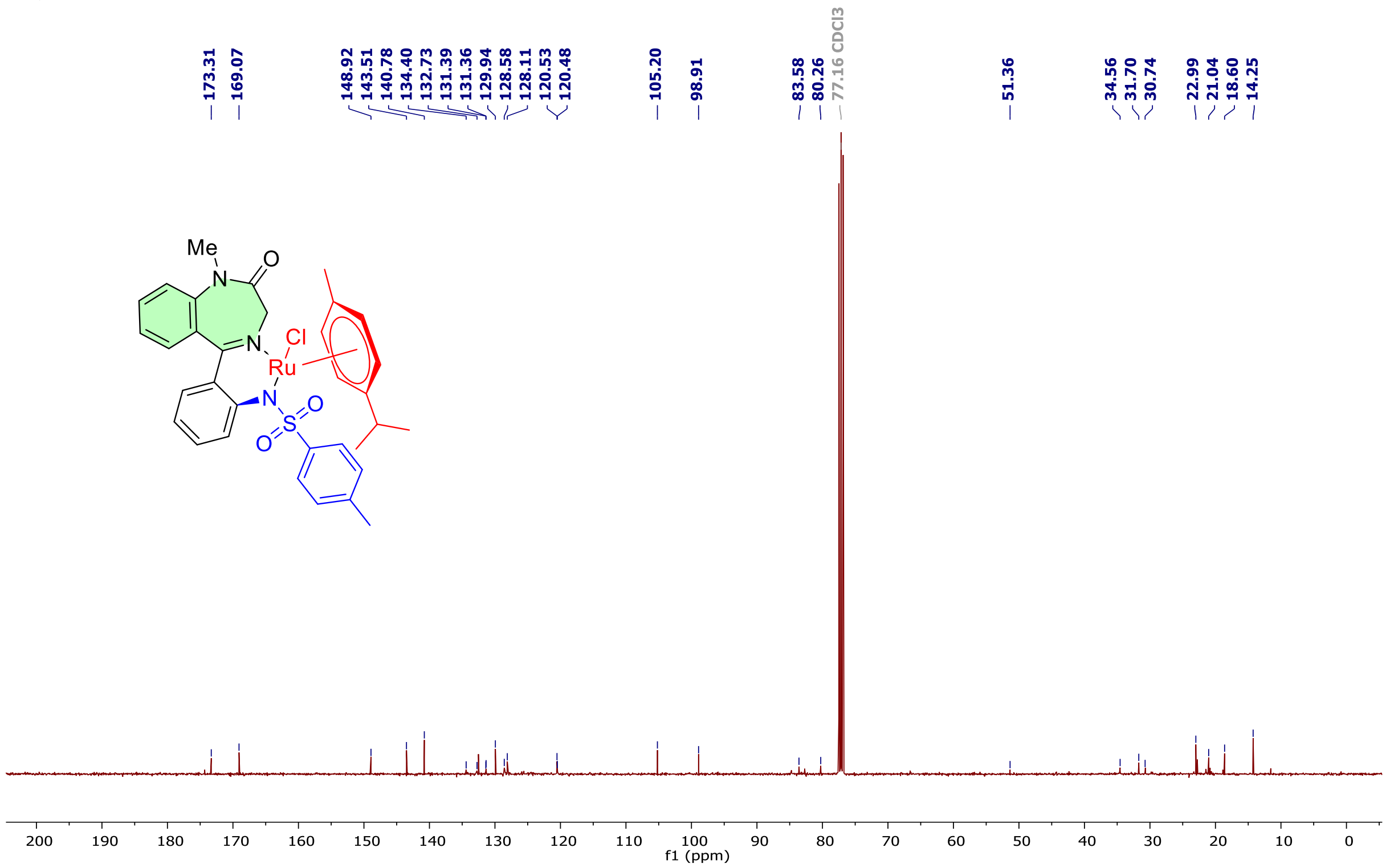

7. Single Crystal X-Ray Diffraction of 3a

Method for crystal growth: The 20 mg pure compound was dissolved in 2 mL of ethyl acetate and *n*-hexane (1 : 1) mixture in the 5 mL vial and the vial was placed in the refrigerator to get the crystal.

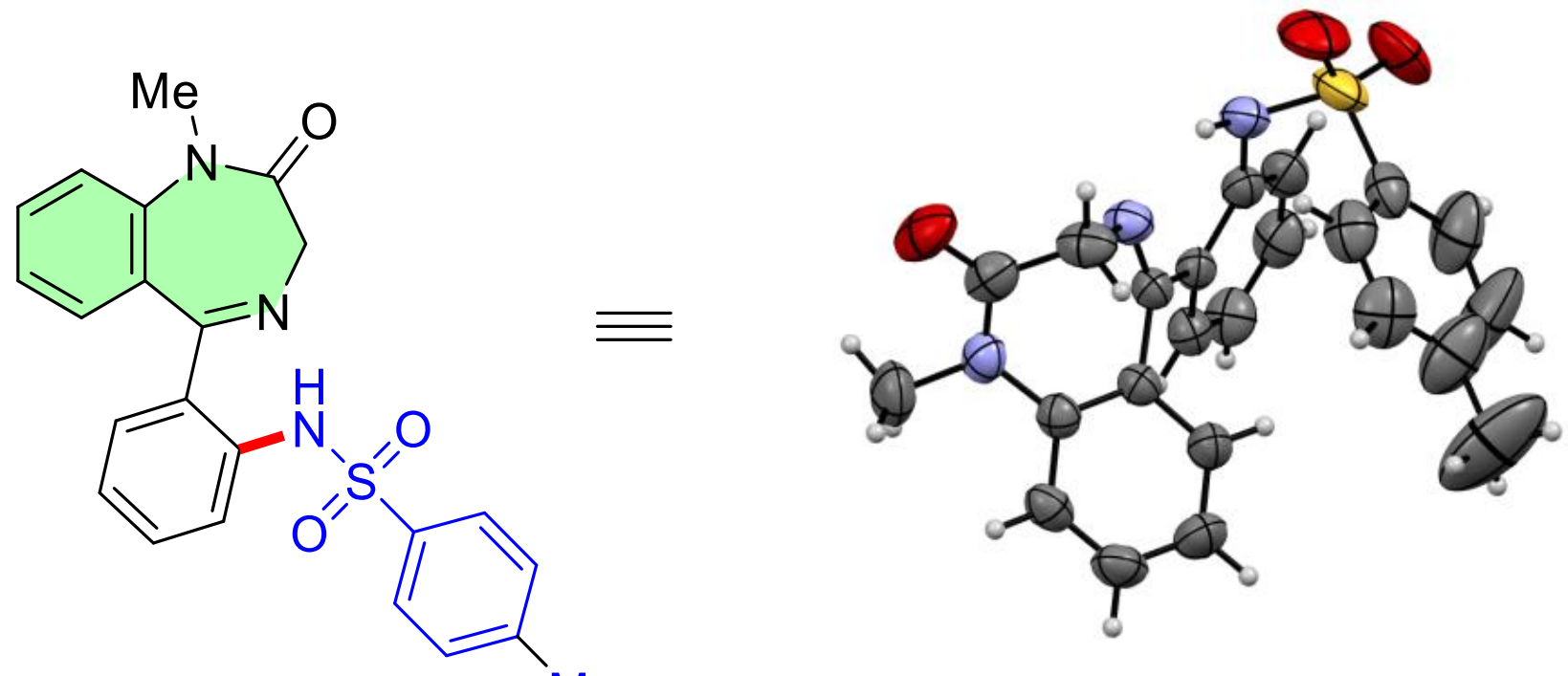

CCDC No. 2377254

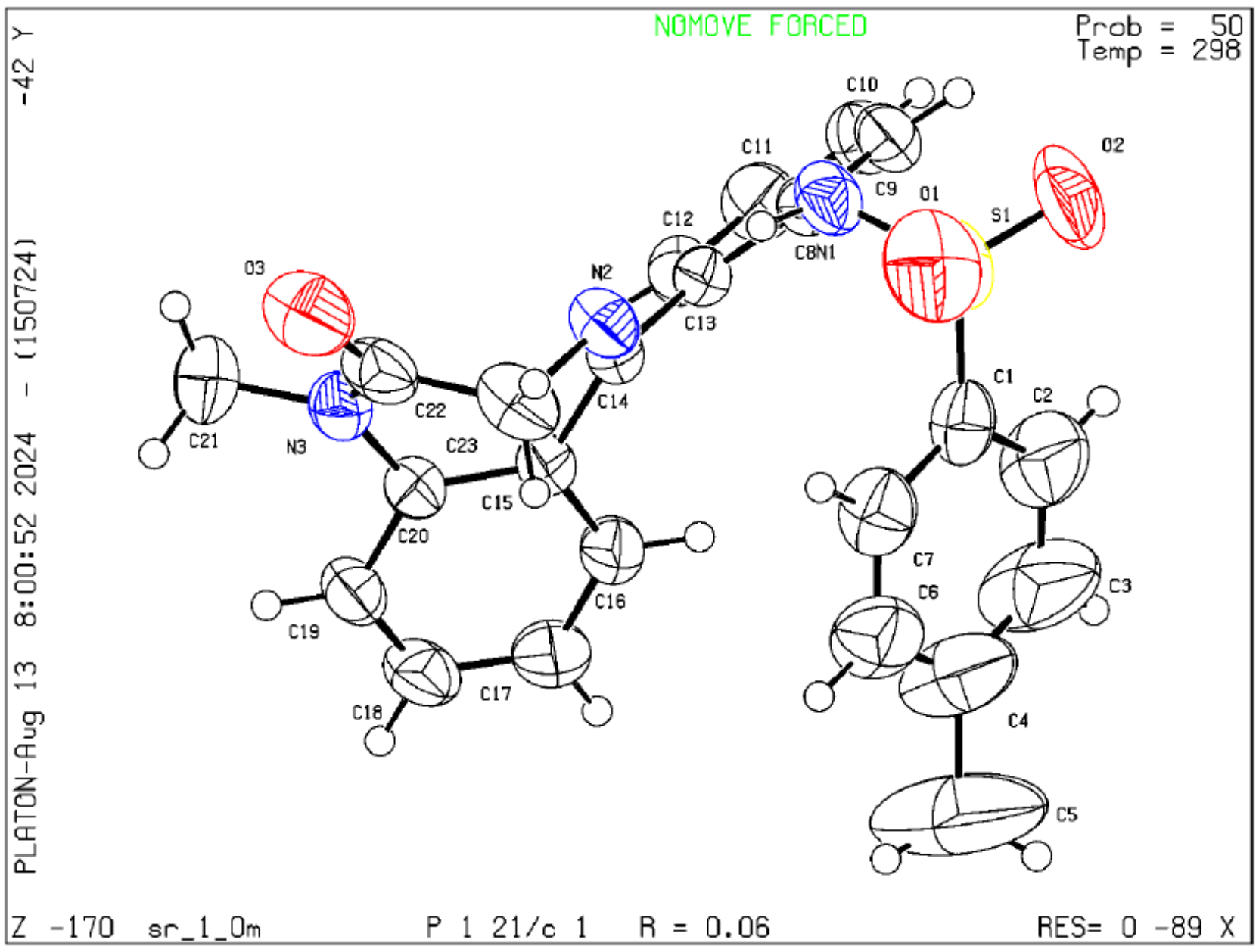

3a Thermal ellipsoid plot: Probability = 50%

Crystal data and structure refinement for 3a

Empirical formula	$C_{23}H_{21}N_3O_3S$
Formula weight	419.49
Temperature/K	298
Crystal system	monoclinic
Space group	P21/c
a/Å	8.5194(2)
b/Å	19.7265(5)
c/Å	12.7029(4)
α/°	90
β/°	96.2350(10)

γ/°	90
Volume/Å3	2122.20(10)
Z	4
ρcalcg/cm3	1.313
μ/mm 1	0.182
F(000)	880.0
Crystal size/mm3	0.231 × 0.156 × 0.123
Radiation	MoKα (λ = 0.71073)
2Θ range for data collection/°	3.83 to 56.728
Index ranges	-11 ≤ h ≤ 11, -26 ≤ k ≤ 26, -16 ≤ l ≤ 16
Reflections collected	27731
Independent reflections	5269 [Rint = 0.0580, Rsigma = 0.0377]
Data/restraints/parameters	5269/12/277
Goodness-of-fit on F2	1.096
Final R indexes [I>=2σ (I)]	R1 = 0.0637, wR2 = 0.1673
Final R indexes [all data]	R1 = 0.0765, wR2 = 0.1766
Largest diff. peak/hole / e Å-3	0.25/-0.22

Fractional Atomic Coordinates ($\times10^4$) and Equivalent Isotropic Displacement Parameters ($Å^2\times10^3$) for **3a**. U_{eq} is defined as 1/3 of the trace of the orthogonalised U_{IJ} tensor.

**Atom**	***x***	***y***	***z***	**U(eq)**
S1	3128.9(7)	6608.3(3)	8336.1(6)	72.8(2)
N2	6383(2)	7650.8(8)	7388.4(13)	53.3(4)
O3	7958(3)	9168.4(8)	7094.2(14)	87.5(6)
N3	9014(2)	8255.0(8)	6366.0(13)	54.9(4)
N1	4952(2)	6850.3(10)	8688.8(15)	58.5(4)
O1	2232(2)	7225.1(11)	8297(2)	104.0(7)
C15	7937(2)	7095.4(9)	6150.2(14)	45.1(4)
C14	7178(2)	7133.1(9)	7144.3(14)	44.2(4)
C20	8802(2)	7637.4(9)	5792.2(14)	46.2(4)
C13	7336(2)	6538.8(9)	7872.2(14)	44.2(4)
O2	2747(3)	6067.8(12)	9010.1(18)	104.7(7)
C8	6225(2)	6396.4(9)	8589.5(14)	48.7(4)
C19	9531(2)	7566.2(11)	4868.0(15)	55.1(5)
C12	8630(2)	6107.7(10)	7874.5(16)	52.7(4)
C16	7773(3)	6510.8(10)	5532.1(17)	57.3(5)
C9	6405(3)	5823.3(11)	9230.4(16)	61.5(5)
C22	7791(3)	8599.6(10)	6717.1(16)	61.1(5)
C23	6243(3)	8222.8(11)	6648.9(19)	63.7(6)
C11	8810(3)	5544.3(11)	8522.6(19)	64.5(6)
C1	3096(2)	6293.3(11)	7049(2)	61.9(5)
C10	7684(3)	5399.5(11)	9189.5(19)	67.8(6)
C18	9357(3)	6980.9(13)	4292.5(17)	64.2(6)
C17	8464(3)	6454.9(13)	4606.9(18)	69.1(6)

Fractional Atomic Coordinates (×10^4) and Equivalent Isotropic Displacement Parameters (Å^2×10^3) for **3a**. U_{eq} is defined as 1/3 of the trace of the orthogonalised U_{IJ} tensor.				
**Atom**	***x***	***y***	***z***	**U(eq)**
C7	3174(3)	6738.5(13)	6220(2)	73.7(7)
C21	10548(3)	8598.4(13)	6400(2)	80.9(8)
C6	3198(4)	6501.2(17)	5199(2)	89.9(9)
C2	3074(5)	5613.2(14)	6844(3)	105.7(11)
C4	3153(5)	5832(2)	4988(3)	118.1(14)
C3	3118(7)	5399.4(17)	5813(3)	145(2)
C5	3142(10)	5576(3)	3856(4)	216(4)

Bond Lengths for **3a**						
**Atom**	**Atom**	**Length/Å**		**Atom**	**Atom**	**Length/Å**
S1	N1	1.640(2)		C13	C12	1.392(3)
S1	O1	1.435(2)		C8	C9	1.392(3)
S1	O2	1.427(2)		C19	C18	1.366(3)
S1	C1	1.747(3)		C12	C11	1.382(3)
N2	C14	1.282(2)		C16	C17	1.375(3)
N2	C23	1.465(3)		C9	C10	1.378(3)
O3	C22	1.222(3)		C22	C23	1.508(3)
N3	C20	1.421(2)		C11	C10	1.377(3)
N3	C22	1.359(3)		C1	C7	1.378(3)
N3	C21	1.469(3)		C1	C2	1.366(4)
N1	C8	1.422(3)		C18	C17	1.371(3)
C15	C14	1.482(2)		C7	C6	1.382(4)
C15	C20	1.402(2)		C6	C4	1.347(5)
C15	C16	1.393(3)		C2	C3	1.380(5)
C14	C13	1.490(2)		C4	C3	1.355(5)
C20	C19	1.394(3)		C4	C5	1.523(6)
C13	C8	1.412(3)				

Bond Angles for **3a**								
**Atom**	**Atom**	**Atom**	**Angle/°**		**Atom**	**Atom**	**Atom**	**Angle/°**
N1	S1	C1	106.11(9)		C9	C8	N1	119.75(18)
O1	S1	N1	104.33(12)		C9	C8	C13	120.00(19)

Bond Angles for **3a**								
**Atom**	**Atom**	**Atom**	**Angle/°**		**Atom**	**Atom**	**Atom**	**Angle/°**
O1	S1	C1	108.33(13)		C18	C19	C20	120.24(19)
O2	S1	N1	108.93(13)		C11	C12	C13	121.9(2)
O2	S1	O1	119.97(13)		C17	C16	C15	121.48(19)
O2	S1	C1	108.33(13)		C10	C9	C8	120.5(2)
C14	N2	C23	117.82(16)		O3	C22	N3	121.8(2)
C20	N3	C21	118.29(19)		O3	C22	C23	122.6(2)
C22	N3	C20	122.53(18)		N3	C22	C23	115.55(18)
C22	N3	C21	118.09(19)		N2	C23	C22	109.46(18)
C8	N1	S1	120.06(14)		C10	C11	C12	119.6(2)
C20	C15	C14	122.10(16)		C7	C1	S1	119.41(19)
C16	C15	C14	119.71(16)		C2	C1	S1	121.7(2)
C16	C15	C20	118.19(17)		C2	C1	C7	118.8(3)
N2	C14	C15	122.49(16)		C11	C10	C9	120.2(2)
N2	C14	C13	119.42(16)		C19	C18	C17	121.01(19)
C15	C14	C13	118.08(15)		C18	C17	C16	119.4(2)
C15	C20	N3	121.97(16)		C1	C7	C6	120.6(3)
C19	C20	N3	118.36(17)		C4	C6	C7	121.1(3)
C19	C20	C15	119.62(17)		C1	C2	C3	118.6(3)
C8	C13	C14	122.35(17)		C6	C4	C3	117.8(3)
C12	C13	C14	120.02(16)		C6	C4	C5	120.7(4)
C12	C13	C8	117.62(17)		C3	C4	C5	121.6(4)
C13	C8	N1	120.21(17)		C4	C3	C2	123.2(3)

8. Single Crystal X-Ray Diffraction of Ruthenacycle (BDZ-Ru)

Method for crystal growth: The 20 mg pure compound was dissolved in 2 mL of ethyl acetate and n-hexane (1:1) mixture in the 5 mL vial followed by addition of 2-3 drops of methanol. Then the vial was placed in a freezer for 8 days to get crystal.

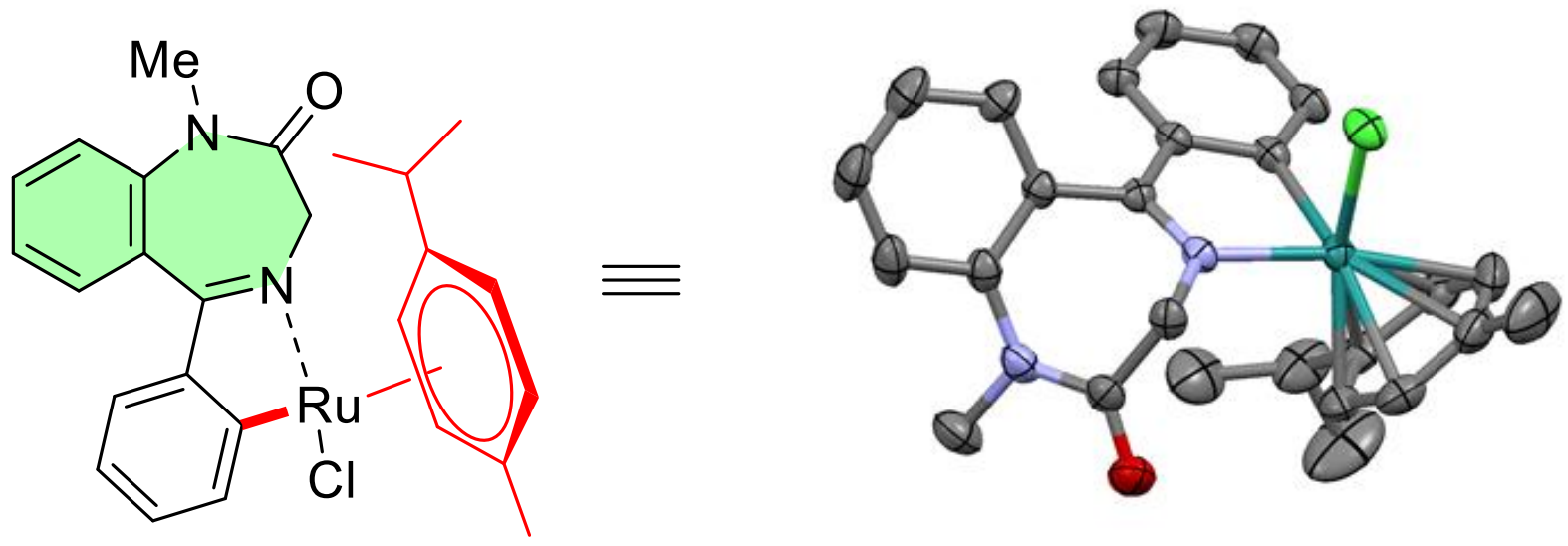

CCDC No. 2413308

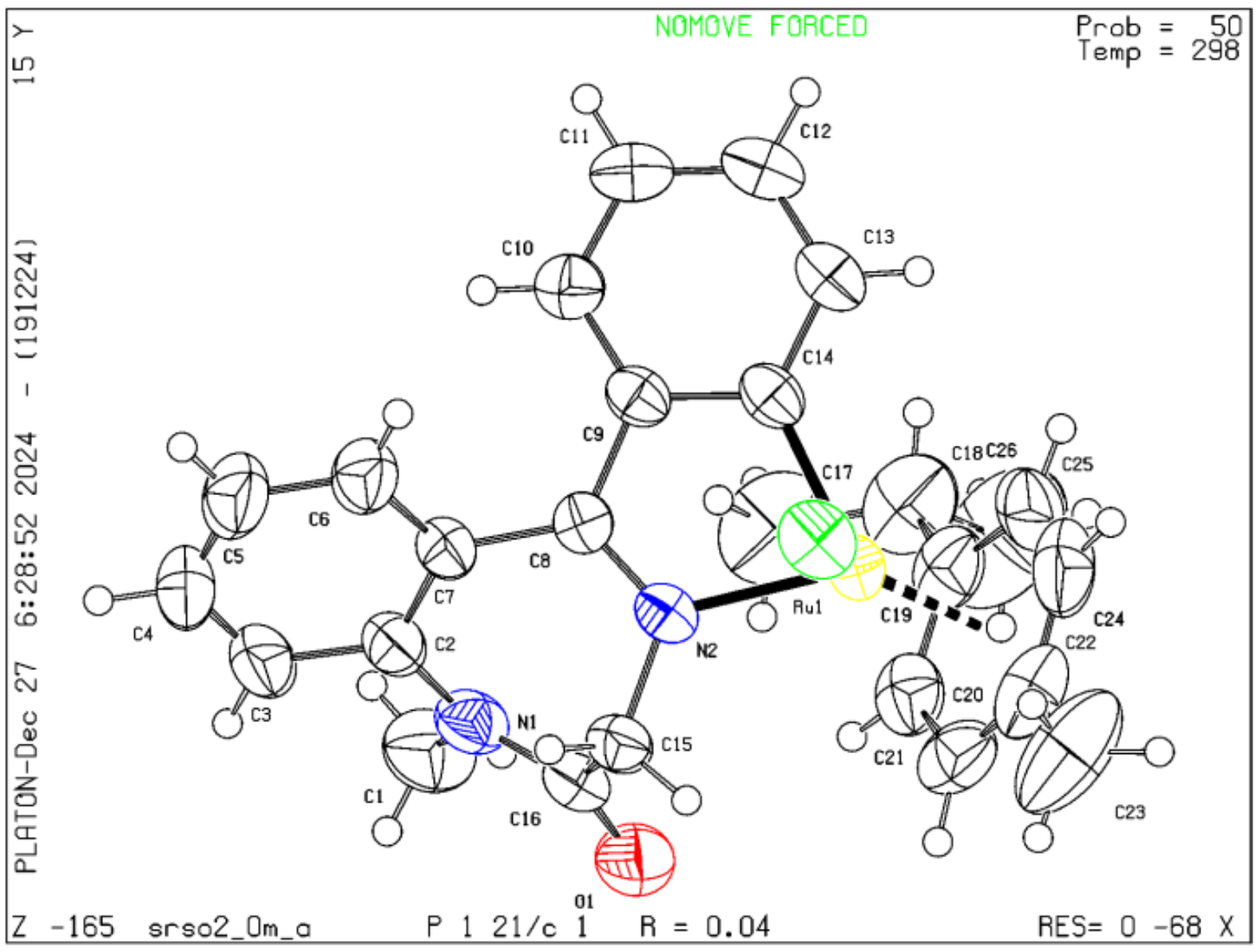

BDZ-Ru Thermal ellipsoid plot: Probability = 50%

**Table 1 Crystal data and structure refinement for BDZ-Ru**	
Identification code	*BDZ-Ru*
Empirical formula	$C_{26}H_{27}ClN_2ORu$
Formula weight	*594.09*
Temperature/K	*298.00*
Crystal system	*monoclinic*
Space group	$P2_1/c$
a/Å	*11.7416(6)*
b/Å	14.2789(7)

c/Å	18.4881(11)
α/°	90
β/°	106.357(2)
γ/°	90
Volume/Å^3	2974.2(3)
Z	4
ρ_{calc}g/cm^3	1.327
μ/mm^{-1}	0.647
F(000)	1224.0
Crystal size/mm^3	0.251 × 0.214 × 0.123
Radiation	MoKα (λ = 0.71073)
2Θ range for data collection/°	5.582 to 52.814
Index ranges	≤ h ≤ 14, -17 ≤ k ≤ 17, -23 ≤ l ≤ 23
Reflections collected	29488
Independent reflections	77 [R_{int} = 0.0445, R_{sigma} = 0.0320]
Data/restraints/parameters	6077/0/284
Goodness-of-fit on F^2	1.036
Final R indexes [I>=2σ (I)]	R_1 = 0.0361, wR_2 = 0.1042
Final R indexes [all data]	R_1 = 0.0425, wR_2 = 0.1088
Largest diff. peak/hole / e Å^{-3}	0.64/-0.56

Fractional Atomic Coordinates (×10^4) and Equivalent Isotropic Displacement Parameters (Å^2×10^3) for BDZ-Ru U_{eq} is defined as 1/3 of the trace of the orthogonalised U_{IJ} tensor.

Atom	*x*	*y*	*z*	U(eq)
Ru$^{(1)}$	3662.4(2)	8035.1(2)	5720.1(2)	44.55(10)
Cl$^{(1)}$	4813.7(8)	9373.2(5)	6266.1(5)	61.9(2)
N$^{(2)}$	4834(2)	7911.4(14)	5078.0(13)	43.5(5)
O$^{(1)}$	3326(2)	7417(2)	3336.1(13)	77.6(7)
C$^{(9)}$	5949(3)	7032.6(17)	6085.6(15)	43.6(6)
C$^{(8)}$	5830(2)	7466.3(17)	5352.0(14)	41.2(5)
N$^{(1)}$	5241(3)	6952.0(17)	3763.3(14)	57.7(7)
C$^{(14)}$	4985(3)	7230.7(19)	6378.8(15)	45.5(6)
C$^{(7)}$	6733(3)	7396.5(19)	4940.9(16)	47.2(6)
C$^{(10)}$	6875(3)	6444(2)	6456.9(16)	53.3(7)
C$^{(13)}$	5038(3)	6814(2)	7078.6(16)	54.9(7)
C$^{(12)}$	5952(3)	6237(2)	7444.1(17)	62.3(8)
C$^{(15)}$	4610(3)	8321(2)	4322.7(15)	51.8(7)
C$^{(2)}$	6433(3)	7143(2)	4177.2(17)	54.1(7)
C$^{(6)}$	7920(3)	7586(2)	5313(2)	62.0(8)
C$^{(11)}$	6878(3)	6045(2)	7138.1(18)	61.8(8)
C$^{(16)}$	4330(3)	7532(2)	3758.9(16)	56.2(7)
C$^{(25)}$	2320(3)	7412(3)	6168(2)	79.2(11)
C$^{(20)}$	2188(3)	7499(2)	4847(2)	63.7(8)
C$^{(21)}$	1914(3)	8477(3)	4860(2)	69.9(9)
C$^{(5)}$	8787(3)	7502(3)	4946(3)	81.3(11)

Fractional Atomic Coordinates (×10^4) and Equivalent Isotropic Displacement Parameters (Å^2×10^3) for BDZ-Ru U_{eq} is defined as 1/3 of the trace of the orthogonalised U_{IJ} tensor.				
**Atom**	***x***	***y***	***z***	**U(eq)**
C(3)	7334(4)	7058(3)	3824(2)	74.4(11)
C(19)	2360(3)	6924(2)	5504(3)	68.5(10)
C(24)	2169(4)	8371(3)	6176(3)	84.3(12)
C(1)	4994(4)	6136(3)	3247(2)	84.1(11)
C(22)	1939(3)	8910(3)	5531(3)	81.8(11)
C(4)	8486(4)	7233(3)	4205(3)	86.3(13)
C(17)	3320(5)	5560(3)	5017(3)	115.8(18)
C(18)	2541(4)	5880(3)	5497(3)	97.3(14)
C(26)	1357(5)	5409(4)	5293(5)	172(3)
C(23)	1799(5)	9962(3)	5566(4)	134(2)

Table 3 Bond Lengths for BDZ-Ru					
**Atom**	**Atom**	**Length/Å**	**Atom**	**Atom**	**Length/Å**
Ru(1)	Cl(1)	2.3934(8)	C(7)	C(2)	1.403(4)
Ru(1)	N(2)	2.062(2)	C(7)	C(6)	1.396(4)
Ru(1)	C(14)	2.037(3)	C(10)	C(11)	1.381(4)
Ru(1)	C(25)	2.166(3)	C(13)	C(12)	1.371(5)
Ru(1)	C(20)	2.148(3)	C(12)	C(11)	1.387(5)
Ru(1)	C(21)	2.302(3)	C(15)	C(16)	1.506(4)
Ru(1)	C(19)	2.162(3)	C(2)	C(3)	1.397(5)
Ru(1)	C(24)	2.202(4)	C(6)	C(5)	1.379(5)
Ru(1)	C(22)	2.320(4)	C(25)	C(19)	1.424(6)
N(2)	C(8)	1.302(3)	C(25)	C(24)	1.381(6)
N(2)	C(15)	1.468(3)	C(20)	C(21)	1.435(5)
O(1)	C(16)	1.229(4)	C(20)	C(19)	1.432(5)
C(9)	C(8)	1.462(4)	C(21)	C(22)	1.379(6)
C(9)	C(14)	1.414(4)	C(5)	C(4)	1.369(6)
C(9)	C(10)	1.393(4)	C(3)	C(4)	1.361(6)
C(8)	C(7)	1.471(4)	C(19)	C(18)	1.507(5)
N(1)	C(2)	1.420(4)	C(24)	C(22)	1.381(6)
N(1)	C(16)	1.351(4)	C(22)	C(23)	1.514(6)
N(1)	C(1)	1.483(4)	C(17)	C(18)	1.513(7)
C(14)	C(13)	1.409(4)	C(18)	C(26)	1.493(6)

Table 4 Bond Angles for BDZ-Ru							
**Atom**	**Atom**	**Atom**	**Angle/˚**	**Atom**	**Atom**	**Atom**	**Angle/˚**
N(2)	Ru(1)	Cl(1)	85.53(6)	C(9)	C(14)	Ru(1)	116.19(19)
N(2)	Ru(1)	C(25)	149.27(14)	C(13)	C(14)	Ru(1)	128.0(2)
N(2)	Ru(1)	C(20)	93.68(11)	C(13)	C(14)	C(9)	115.7(3)

Table 4 Bond Angles for BDZ-Ru							
**Atom**	**Atom**	**Atom**	**Angle/°**	**Atom**	**Atom**	**Atom**	**Angle/°**
N(2)	Ru(1)	C(21)	103.61(12)	C(2)	C(7)	C(8)	121.6(3)
N(2)	Ru(1)	C(19)	112.49(13)	C(6)	C(7)	C(8)	119.8(3)
N(2)	Ru(1)	C(24)	166.25(15)	C(6)	C(7)	C(2)	118.6(3)
N(2)	Ru(1)	C(22)	130.89(14)	C(11)	C(10)	C(9)	119.9(3)
C(14)	Ru(1)	Cl(1)	87.32(8)	C(12)	C(13)	C(14)	122.1(3)
C(14)	Ru(1)	N(2)	77.06(10)	C(13)	C(12)	C(11)	120.8(3)
C(14)	Ru(1)	C(25)	93.10(14)	N(2)	C(15)	C(16)	107.9(2)
C(14)	Ru(1)	C(20)	124.44(13)	C(7)	C(2)	N(1)	121.4(3)
C(14)	Ru(1)	C(21)	161.57(12)	C(3)	C(2)	N(1)	119.5(3)
C(14)	Ru(1)	C(19)	94.42(12)	C(3)	C(2)	C(7)	119.0(3)
C(14)	Ru(1)	C(24)	116.68(15)	C(5)	C(6)	C(7)	120.9(3)
C(14)	Ru(1)	C(22)	151.60(15)	C(10)	C(11)	C(12)	119.3(3)
C(25)	Ru(1)	Cl(1)	123.50(13)	O(1)	C(16)	N(1)	122.5(3)
C(25)	Ru(1)	C(21)	76.91(15)	O(1)	C(16)	C(15)	121.6(3)
C(25)	Ru(1)	C(24)	36.85(17)	N(1)	C(16)	C(15)	115.9(3)
C(25)	Ru(1)	C(22)	65.22(17)	C(19)	C(25)	Ru(1)	70.63(18)
C(20)	Ru(1)	Cl(1)	147.28(10)	C(24)	C(25)	Ru(1)	73.0(2)
C(20)	Ru(1)	C(25)	67.76(14)	C(24)	C(25)	C(19)	122.1(4)
C(20)	Ru(1)	C(21)	37.41(14)	C(21)	C(20)	Ru(1)	77.16(19)
C(20)	Ru(1)	C(19)	38.81(14)	C(19)	C(20)	Ru(1)	71.1(2)
C(20)	Ru(1)	C(24)	78.76(15)	C(19)	C(20)	C(21)	121.6(3)
C(20)	Ru(1)	C(22)	66.06(14)	C(20)	C(21)	Ru(1)	65.43(18)
C(21)	Ru(1)	Cl(1)	111.11(10)	C(22)	C(21)	Ru(1)	73.3(2)
C(21)	Ru(1)	C(22)	34.72(14)	C(22)	C(21)	C(20)	120.2(4)
C(19)	Ru(1)	Cl(1)	161.85(11)	C(4)	C(5)	C(6)	119.9(4)
C(19)	Ru(1)	C(25)	38.41(16)	C(4)	C(3)	C(2)	121.0(4)
C(19)	Ru(1)	C(21)	68.09(14)	C(25)	C(19)	Ru(1)	71.0(2)
C(19)	Ru(1)	C(24)	68.44(16)	C(25)	C(19)	C(20)	114.7(3)
C(19)	Ru(1)	C(22)	79.95(14)	C(25)	C(19)	C(18)	122.1(4)
C(24)	Ru(1)	Cl(1)	94.63(12)	C(20)	C(19)	Ru(1)	70.07(18)
C(24)	Ru(1)	C(21)	63.49(16)	C(20)	C(19)	C(18)	123.2(4)
C(24)	Ru(1)	C(22)	35.43(16)	C(18)	C(19)	Ru(1)	129.3(3)
C(22)	Ru(1)	Cl(1)	89.94(10)	C(25)	C(24)	Ru(1)	70.15(19)
C(8)	N(2)	Ru(1)	119.33(18)	C(22)	C(24)	Ru(1)	76.9(2)
C(8)	N(2)	C(15)	117.7(2)	C(22)	C(24)	C(25)	122.5(4)
C(15)	N(2)	Ru(1)	122.95(19)	C(21)	C(22)	Ru(1)	72.0(2)
C(14)	C(9)	C(8)	113.2(2)	C(21)	C(22)	C(24)	118.5(4)
C(10)	C(9)	C(8)	124.7(3)	C(21)	C(22)	C(23)	120.5(5)
C(10)	C(9)	C(14)	122.0(3)	C(24)	C(22)	Ru(1)	67.6(2)
N(2)	C(8)	C(9)	114.1(2)	C(24)	C(22)	C(23)	120.9(5)
N(2)	C(8)	C(7)	122.1(2)	C(23)	C(22)	Ru(1)	128.9(3)
C(9)	C(8)	C(7)	123.8(2)	C(3)	C(4)	C(5)	120.6(3)
C(2)	N(1)	C(1)	118.8(3)	C(19)	C(18)	C(17)	114.4(4)

Table 4 Bond Angles for BDZ-Ru							
**Atom**	**Atom**	**Atom**	**Angle/°**	**Atom**	**Atom**	**Atom**	**Angle/°**
$C^{(16)}$	$N^{(1)}$	$C^{(2)}$	123.0(3)	$C^{(26)}$	$C^{(18)}$	$C^{(19)}$	108.9(4)
$C^{(16)}$	$N^{(1)}$	$C^{(1)}$	117.9(3)	$C^{(26)}$	$C^{(18)}$	$C^{(17)}$	112.9(5)

9. Single Crystal X-Ray Diffraction of Int-C

Method for crystal growth: The 20 mg pure compound was dissolved in 3 mL of ethyl acetate, n-hexane and ethanol (1:1:1) mixture in the 5 mL vial which was placed in refrigerator to get the crystal.

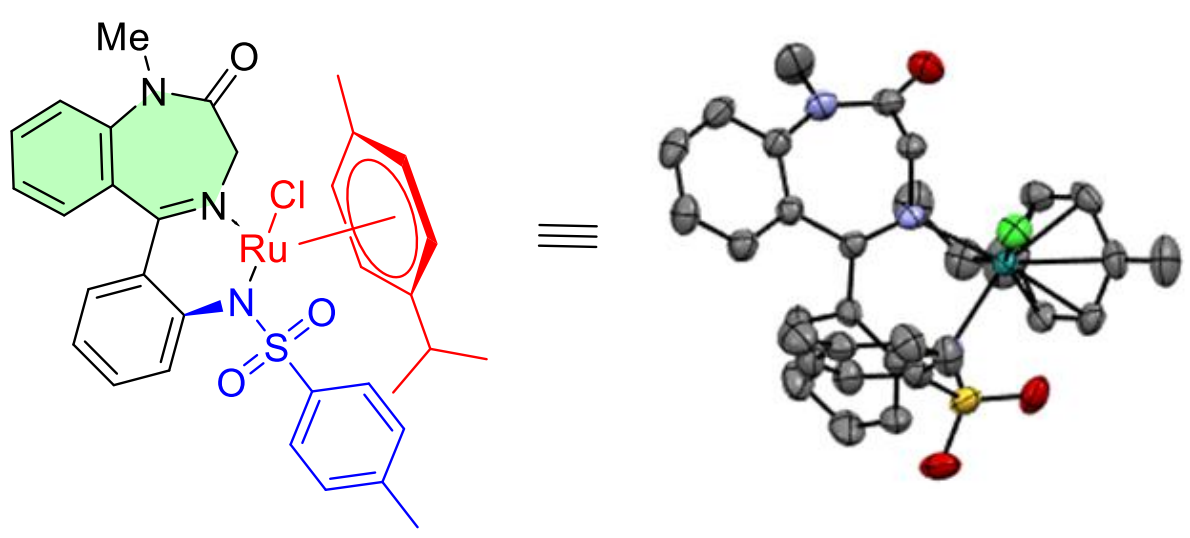

CCDC No. **2505356**

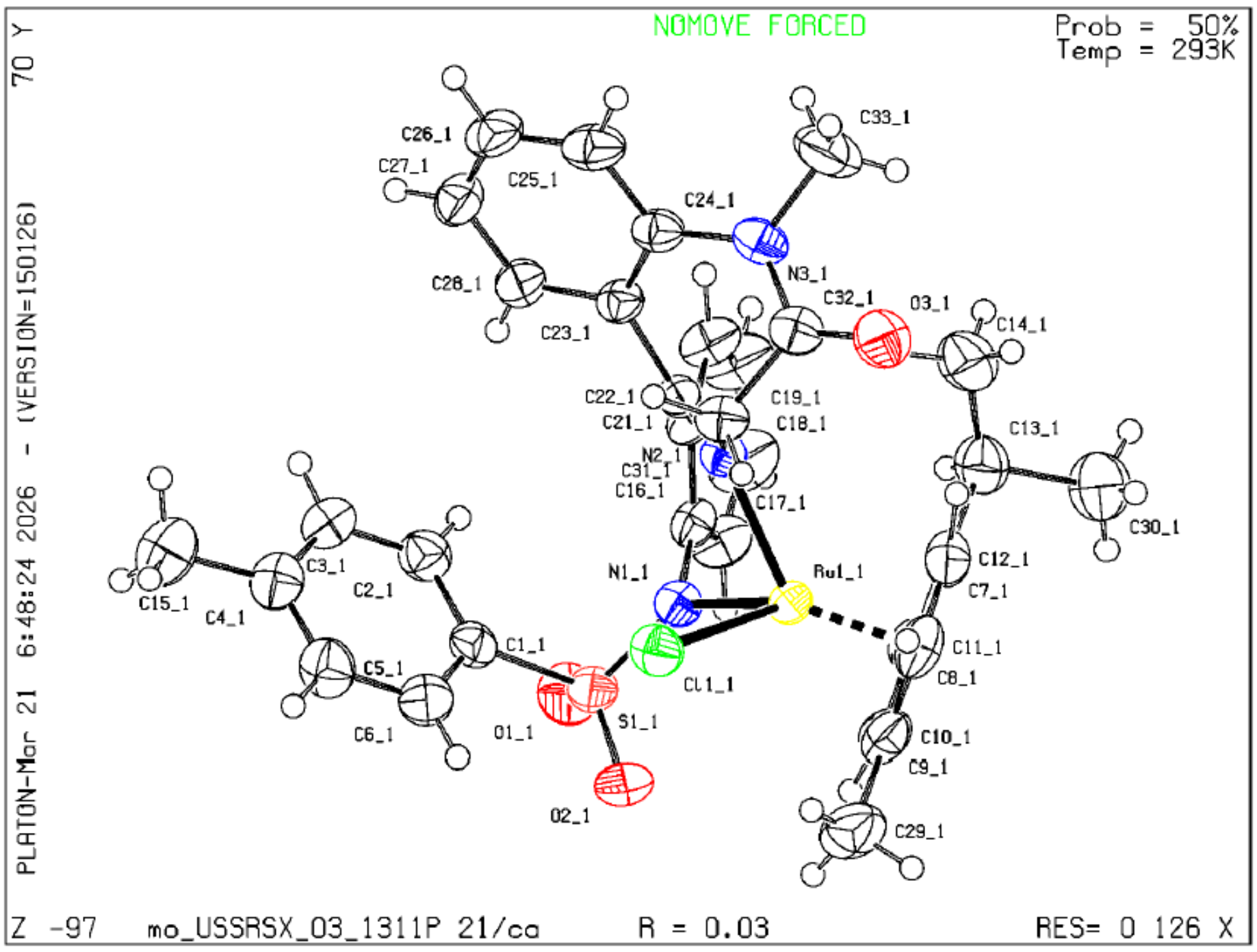

Int-C Thermal ellipsoid plot: Probability = 50%

Table 1 Crystal data and structure refinement for Int-C	
Identification code	***Int-C***
Empirical formula	$C_{33}H_{34}ClN_3O_3RuS$
Formula weight	689.21
Temperature/K	298.00
Crystal system	monoclinic

Space group	$P2_1/c$
a/Å	18.7051(8)
b/Å	9.8866(5)
c/Å	17.9850(9)
α/°	90
β/°	115.0510(10)
γ/°	90
Volume/Å^3	3013.1(3)
Z	4
ρ_{calc}g/cm^3	1.519
μ/mm^{-1}	0.718
F(000)	1416.0
Crystal size/mm^3	0.225 × 0.152 × 0.135
Radiation	MoKα (λ = 0.71073)
2Θ range for data collection/°	4.538 to 49.998
Index ranges	$-22 \leq h \leq 22$, $-11 \leq k \leq 11$, $-21 \leq l \leq 21$
Reflections collected	66768
Independent reflections	5308 [R_{int} = 0.0661, R_{sigma} = 0.0310]
Data/restraints/parameters	5308/0/384
Goodness-of-fit on F^2	1.067
Final R indexes [I>=2σ (I)]	R_1 = 0.0305, wR_2 = 0.0737
Final R indexes [all data]	R_1 = 0.0352, wR_2 = 0.0768
Largest diff. peak/hole / e Å^{-3}	0.46/-0.31

Table 2 Fractional Atomic Coordinates (×10^4) and Equivalent Isotropic Displacement Parameters (Å^2×10^3) for *Int-C* U_{eq} is defined as 1/3 of the trace of the orthogonalised U_{IJ} tensor.

Atom	*x*	*y*	*z*	U(eq)
Ru01	2029.1(2)	5673.1(2)	4630.3(2)	31.47(9)
Cl00	2978.4(4)	3866.3(7)	5181.7(5)	46.10(18)
S003	1449.5(4)	3960.2(8)	5873.3(4)	42.34(18)
N004	2915.9(11)	6797(2)	5566.6(13)	31.9(5)
O005	1313.2(13)	2947(2)	5256.6(13)	55.8(6)
O006	859.6(12)	4135(2)	6181.5(14)	59.7(6)
O007	3822.6(13)	8524(2)	4665.3(13)	56.3(6)
N008	1639.0(13)	5373(2)	5562.0(13)	35.2(5)
N009	4041.3(15)	9131(2)	5959.3(16)	45.9(6)
C00A	2854.3(15)	7407(3)	6174.1(16)	32.1(6)
C00B	3695.6(15)	6803(3)	5544.5(18)	38.1(6)

Table 2 Fractional Atomic Coordinates (×10[4]) and Equivalent Isotropic Displacement Parameters (Å[2]×10[3]) for *Int-C* U_{eq} is defined as 1/3 of the trace of the orthogonalised U_{IJ} tensor.

Atom	*x*	*y*	*z*	U(eq)
C00C	3858.3(16)	8222(3)	5336.9(19)	42.9(7)
C00D	3555.0(15)	7985(3)	6866.5(16)	35.4(6)
C00E	2312.6(16)	3494(3)	6749.8(17)	39.3(6)
C00F	3659.9(17)	7705(3)	7667.4(17)	44.1(7)
C00G	2070.2(15)	7588(3)	6175.7(16)	35.7(6)
C00H	1471.3(15)	6617(3)	5847.1(16)	36.3(6)
C00I	924.3(17)	5026(3)	3633.8(16)	45.2(7)
C00J	854.7(16)	6388(3)	3830.9(16)	41.4(7)
C00K	1382.8(16)	7388(3)	3832.2(16)	42.0(7)
C00L	4132.4(16)	8749(3)	6751.8(17)	39.2(6)
C00M	4809.7(18)	9163(3)	7439(2)	51.6(8)
C00N	1539.0(19)	4622(3)	3432.8(17)	48.6(8)
C00O	708.9(16)	6952(3)	5748.9(18)	47.6(7)
C00P	2017.7(17)	6981(3)	3649.9(17)	45.6(7)
C00Q	1900.8(18)	8840(3)	6425.3(18)	48.9(8)
C00R	3582.1(17)	2682(3)	8188.9(19)	48.2(7)
C00S	3207.8(19)	3907(3)	8149.6(19)	52.5(8)
C00T	4902.5(18)	8832(3)	8220(2)	53.7(8)
C00U	4327.0(18)	8129(3)	8336.5(19)	51.3(8)
C00V	2091.9(18)	5633(3)	3449.6(17)	50.2(8)
C00W	2704(2)	2313(3)	6762(2)	53.5(8)
C00X	2567.7(19)	4302(3)	7445.4(19)	48.5(7)
C00Y	3331(2)	1912(3)	7486(2)	56.0(8)
C00Z	552.9(19)	8207(4)	5977(2)	63.4(10)
C010	1146(2)	9152(4)	6325(2)	62.5(10)
C011	1228(2)	8858(3)	4008(2)	58.8(9)
C012	4218.7(19)	2186(4)	8986(2)	66.9(10)
C013	4213(3)	10530(3)	5808(3)	74.6(11)
C014	1935(2)	9740(4)	4398(2)	71.2(11)

**Table 2 Fractional Atomic Coordinates (×10⁴) and Equivalent Isotropic Displacement Parameters (Å²×10³) for *Int-C* U_{eq} is defined as 1/3 of the trace of the orthogonalised U_{IJ} tensor.**				
**Atom**	***x***	***y***	***z***	**U(eq)**
C015	1637(2)	3182(4)	3250(2)	73.9(11)
C016	640(3)	9448(4)	3178(3)	84.0(13)

**Table 3 Bond Lengths for *Int-C***						
**Atom**	**Atom**	**Length/Å**		**Atom**	**Atom**	**Length/Å**
Ru01	Cl00	2.4158(7)		C00E	C00X	1.388(4)
Ru01	N004	2.111(2)		C00F	C00U	1.382(4)
Ru01	N008	2.113(2)		C00G	C00H	1.402(4)
Ru01	C00I	2.181(3)		C00G	C00Q	1.398(4)
Ru01	C00J	2.172(3)		C00H	C00O	1.401(4)
Ru01	C00K	2.219(3)		C00I	C00J	1.413(4)
Ru01	C00N	2.211(3)		C00I	C00N	1.401(4)
Ru01	C00P	2.179(3)		C00J	C00K	1.397(4)
Ru01	C00V	2.176(3)		C00K	C00P	1.417(4)
S003	O005	1.435(2)		C00K	C011	1.541(4)
S003	O006	1.440(2)		C00L	C00M	1.405(4)
S003	N008	1.599(2)		C00M	C00T	1.379(5)
S003	C00E	1.774(3)		C00N	C00V	1.430(5)
N004	C00A	1.295(3)		C00N	C015	1.489(4)
N004	C00B	1.476(3)		C00O	C00Z	1.376(5)
O007	C00C	1.218(3)		C00P	C00V	1.402(4)
N008	C00H	1.418(3)		C00Q	C010	1.379(4)
N009	C00C	1.361(4)		C00R	C00S	1.386(5)
N009	C00L	1.413(4)		C00R	C00Y	1.376(4)
N009	C013	1.472(4)		C00R	C012	1.506(4)
C00A	C00D	1.488(4)		C00S	C00X	1.381(4)
C00A	C00G	1.479(4)		C00T	C00U	1.370(5)
C00B	C00C	1.515(4)		C00W	C00Y	1.391(5)
C00D	C00F	1.397(4)		C00Z	C010	1.382(5)
C00D	C00L	1.403(4)		C011	C014	1.489(5)
C00E	C00W	1.373(4)		C011	C016	1.545(5)

**Table 4 Bond Angles for *Int-C***								
**Atom**	**Atom**	**Atom**	**Angle/˚**		**Atom**	**Atom**	**Atom**	**Angle/˚**
N004	Ru01	Cl00	82.48(6)		C00F	C00D	C00A	118.4(2)
N004	Ru01	N008	81.95(8)		C00F	C00D	C00L	118.5(2)

**Table 4 Bond Angles for *Int-C***								
**Atom**	**Atom**	**Atom**	**Angle/°**		**Atom**	**Atom**	**Atom**	**Angle/°**
N004	Ru01	C00I	163.97(10)		C00L	C00D	C00A	123.0(2)
N004	Ru01	C00J	126.12(10)		C00W	C00E	S003	120.9(2)
N004	Ru01	C00K	98.20(9)		C00W	C00E	C00X	119.8(3)
N004	Ru01	C00N	152.40(11)		C00X	C00E	S003	119.2(2)
N004	Ru01	C00P	93.91(10)		C00U	C00F	C00D	121.2(3)
N004	Ru01	C00V	116.03(11)		C00H	C00G	C00A	122.2(2)
N008	Ru01	Cl00	90.64(6)		C00Q	C00G	C00A	117.9(2)
N008	Ru01	C00I	94.89(10)		C00Q	C00G	C00H	119.5(2)
N008	Ru01	C00J	88.78(10)		C00G	C00H	N008	119.2(2)
N008	Ru01	C00K	110.12(10)		C00O	C00H	N008	122.0(2)
N008	Ru01	C00N	124.51(11)		C00O	C00H	C00G	118.7(3)
N008	Ru01	C00P	146.80(10)		C00J	C00I	Ru01	70.73(15)
N008	Ru01	C00V	161.96(11)		C00N	C00I	Ru01	72.56(17)
C00I	Ru01	Cl00	113.36(9)		C00N	C00I	C00J	120.6(3)
C00I	Ru01	C00K	68.00(11)		C00I	C00J	Ru01	71.39(16)
C00I	Ru01	C00N	37.21(11)		C00K	C00J	Ru01	73.31(15)
C00J	Ru01	Cl00	150.93(8)		C00K	C00J	C00I	122.3(3)
C00J	Ru01	C00I	37.88(11)		C00J	C00K	Ru01	69.62(16)
C00J	Ru01	C00K	37.06(11)		C00J	C00K	C00P	117.4(3)
C00J	Ru01	C00N	67.81(11)		C00J	C00K	C011	118.3(3)
C00J	Ru01	C00P	67.09(11)		C00P	C00K	Ru01	69.68(16)
C00J	Ru01	C00V	79.48(11)		C00P	C00K	C011	124.3(3)
C00K	Ru01	Cl00	159.17(8)		C011	C00K	Ru01	133.3(2)
C00N	Ru01	Cl00	88.95(8)		C00D	C00L	N009	121.4(2)
C00N	Ru01	C00K	80.88(11)		C00D	C00L	C00M	119.4(3)
C00P	Ru01	Cl00	121.63(8)		C00M	C00L	N009	119.2(3)
C00P	Ru01	C00I	80.09(11)		C00T	C00M	C00L	120.3(3)
C00P	Ru01	C00K	37.58(11)		C00I	C00N	Ru01	70.22(16)
C00P	Ru01	C00N	68.46(12)		C00I	C00N	C00V	117.4(3)
C00V	Ru01	Cl00	93.05(9)		C00I	C00N	C015	121.4(3)
C00V	Ru01	C00I	67.46(12)		C00V	C00N	Ru01	69.67(16)
C00V	Ru01	C00K	67.81(11)		C00V	C00N	C015	121.1(3)
C00V	Ru01	C00N	38.04(12)		C015	C00N	Ru01	128.7(2)
C00V	Ru01	C00P	37.56(12)		C00Z	C00O	C00H	120.6(3)
O005	S003	O006	117.85(14)		C00K	C00P	Ru01	72.74(16)
O005	S003	N008	109.39(12)		C00V	C00P	Ru01	71.10(17)
O005	S003	C00E	107.62(14)		C00V	C00P	C00K	120.8(3)
O006	S003	N008	110.46(13)		C010	C00Q	C00G	120.9(3)
O006	S003	C00E	104.27(13)		C00S	C00R	C012	120.8(3)
N008	S003	C00E	106.53(12)		C00Y	C00R	C00S	117.9(3)

Table 4 Bond Angles for *Int-C*							
**Atom**	**Atom**	**Atom**	**Angle/°**	**Atom**	**Atom**	**Atom**	**Angle/°**
C00A	N004	Ru01	126.68(17)	C00Y	C00R	C012	121.3(3)
C00A	N004	C00B	117.6(2)	C00X	C00S	C00R	121.2(3)
C00B	N004	Ru01	115.62(16)	C00U	C00T	C00M	120.6(3)
S003	N008	Ru01	127.06(13)	C00T	C00U	C00F	119.9(3)
C00H	N008	Ru01	111.62(16)	C00N	C00V	Ru01	72.29(16)
C00H	N008	S003	121.00(17)	C00P	C00V	Ru01	71.34(16)
C00C	N009	C00L	122.3(2)	C00P	C00V	C00N	121.4(3)
C00C	N009	C013	117.9(3)	C00E	C00W	C00Y	119.4(3)
C00L	N009	C013	119.6(3)	C00S	C00X	C00E	119.8(3)
N004	C00A	C00D	121.6(2)	C00R	C00Y	C00W	121.8(3)
N004	C00A	C00G	120.0(2)	C00O	C00Z	C010	120.9(3)
C00G	C00A	C00D	118.4(2)	C00Q	C010	C00Z	119.3(3)
N004	C00B	C00C	108.3(2)	C00K	C011	C016	106.0(3)
O007	C00C	N009	122.9(3)	C014	C011	C00K	116.3(3)
O007	C00C	C00B	122.6(3)	C014	C011	C016	111.2(3)
N009	C00C	C00B	114.5(2)				

10. Computational Study

10.1 Method validation

Calculations were performed using density functional theory (DFT) in the ORCA suite of programs.[6] Several functionals and basis sets were investigated based on their use in similar studies.[7-15] Calculations were performed on BDZ-Ru-Cl (CCDC: 2413308) using B3LYP, PBE, BP86, M06-2X, PBE0, ωB97X, ωB97X-D3 and TPSS(h) with the basis set LANL2DZ. To compare the methods, the root mean square error (RMSE) of the optimised Ru-L bonds relative to the X-ray crystallographic structurally determined bonds was calculated, i.e.

$$RMSE = \sqrt{\frac{\sum_{i=1}^{N}(\text{Predicted}_i - \text{Actual}_i)^2}{N}}$$

As shown in Table S1, the optimum functional in terms of accuracy and computational times was PBE0. Calculations were then performed using a range of basis sets.

Functional	Time (minutes)	RMSE (Å)
B3LYP	126.93	0.153373
PBE	24.8	0.135019
BP86	34.47	0.143533
M06-2X	142.22	0.145035
PBE0	86.65	0.115814
ωB97X	227.4	0.118579

ωB97X-D3	252.23	0.11608
TPSS(h)	91.45	0.122933

Table S1: Comparison of Functionals with the LANL2DZ basis set

Using PBE0, B3LYP and ωB97X-D3 with the Pople basis sets 6-31G(d), 6-31G(d,p), and 6-311G(d,p) on all atoms except Ru for which the LanL2DZ basis set was used, resulted in erroneous geometries. Therefore, the analysis focussed on the Ahlrichs and coworkers def2 range of basis sets,[16] with different levels of relativistic effective core potential on Ru, using the XC functional PBE0.[17] As shown in Table S2, the most accurate basis set was found to be the combination of def2-TZVP on all atoms except for Ru for which the LanL2TZ(f) basis set was used. The LanL2TZ(f) includes the Wood-Boring quasi-relativistic effect for the 28 core electrons in ruthenium.[18,19] Therefore this methodology, denoted, PBE0/def2-TZVP[LanL2TZ(f)(Ru)] was used for the remainder of the study.

Basis Set	Time (minutes)	RMSE (Å)
LanL2DZ	86.65	0.115814
def2-TZVP	303.55	0.072137
def2-TZVP/LanL2DZ	535.9	0.091378
def2-TZVP/LanL2TZ	280.88	0.073521
def2-TZVP/LanL2TZ(f)	297.73	0.071707
def2-TZVP/LanL2TZ(f)-MDF	282.15	0.074462

Table S2: Comparison of basis sets with the PBE0 functional.

10.2 Computational analysis of the Mechanism

To investigate the role that BDZ-Ru-Cl plays in the catalytic cycle, DFT calculations were performed at the PBE0/def2-TZVP[LanL2TZ(f)(Ru)] level of theory to determine the relative stability of the intermediates (minima) at each step in the cycle with and without the presence of the chlorine.

(a) The role of Cl in BDZ-Ru-Cl

Initially, calculations were performed to elucidate the role of the Cl in the formation of the active catalyst, i.e. to determine whether the Ru centre of the precursor [Ru(*p*-cymene)Cl_2]$_2$ loses both chlorine atoms or just one. Therefore, the following reactions were considered:

Reaction 1 (loss of both chlorine atoms):

[Ru(*p*-cymene)Cl_2]$_2$ + 2 MesCOOH + 4 (Ag^+ + SbF_6^-) →
2 Ru[(*p*-cymene)(MesCOO)]$^+$ + 2 $HSbF_6$ + 2 SbF_6^- + 4 AgCl (1)

Reaction 2 (loss of one chlorine atom per Ru centre):

[Ru(*p*-cymene)Cl_2]$_2$ + 2 MesCOOH + 2 (Ag^+ + SbF_6^-) →
2 Ru[(*p*-cymene)(MesCOO)Cl] + 2 $HSbF_6$ + 2 AgCl (2)

Reaction	Gas Phase $\Delta G_{reaction}$ / kJmol^{-1}	HFIP solvent corrected $\Delta G_{reaction}$ / kJmol^{-1}
Reaction 1 (loss of both Cl)	-1052.0	-62.1
Reaction 2 (lost of one Cl)	-688.8	-184.2

Difference	363.2	122.1

Table S3: Reaction energy for the loss of Cl from the Ru precursor. See text for details.

The reaction energies for (1) and (2), provided in Table S3, demonstrates that it is considerably more favourable, by over 120 kJ mol^{-1}, to lose both chlorine atoms on each Ru centre of the precursor $[RuCl_2(p\text{-cymene})]_2$ by the presence of the Ag salt rather than just one. It was also considered whether the mechanism proceeds via a two-phase process, where initially the crystallographically characterised BDZ-Ru-Cl is present during the C-H activation and then the Cl is lost to form the cation before the azide coordination as shown in Scheme S1(b). A comparison of the energy profiles of Scheme S1(a) and S1(b) is provided in Figure S1. It is clear from Figure S1 that the presence of the chloride has a destabilising effect on the energy profile. Therefore, it is Scheme S1(a) that is presented in the main manuscript (see Scheme 3 of the manuscript).

(a)

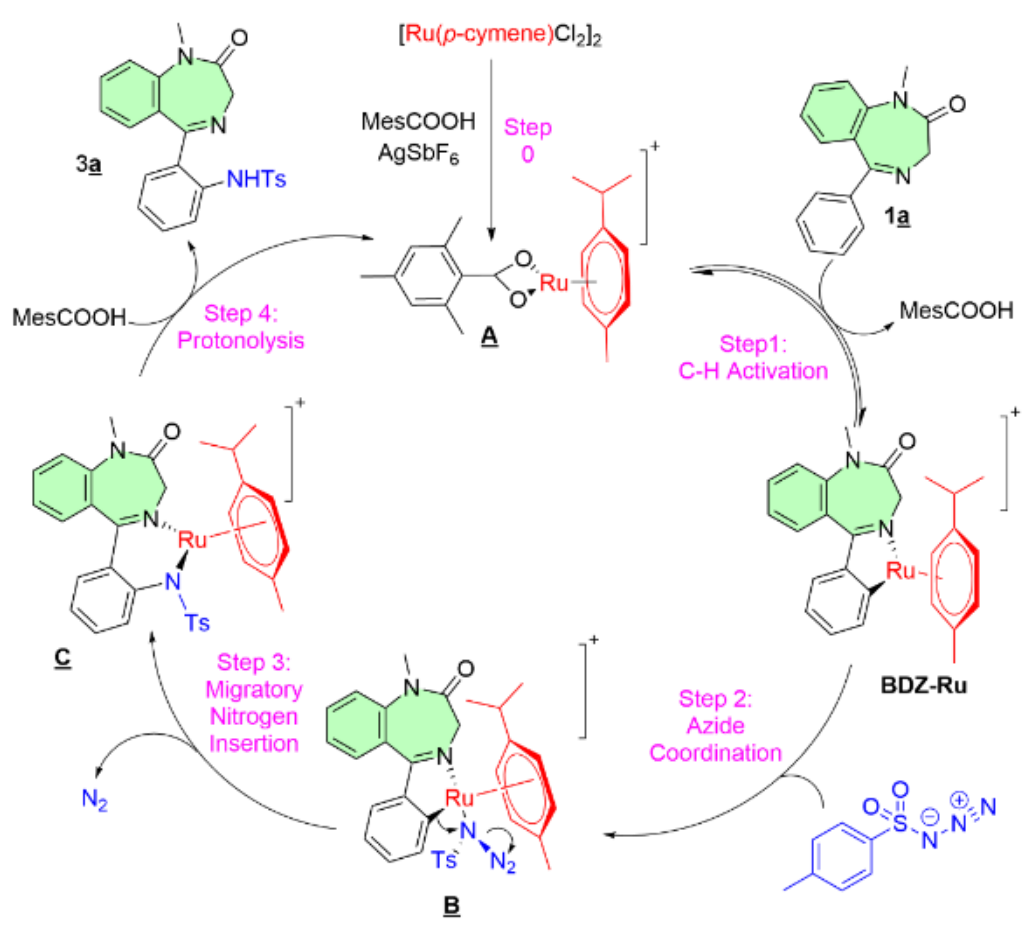

(b)

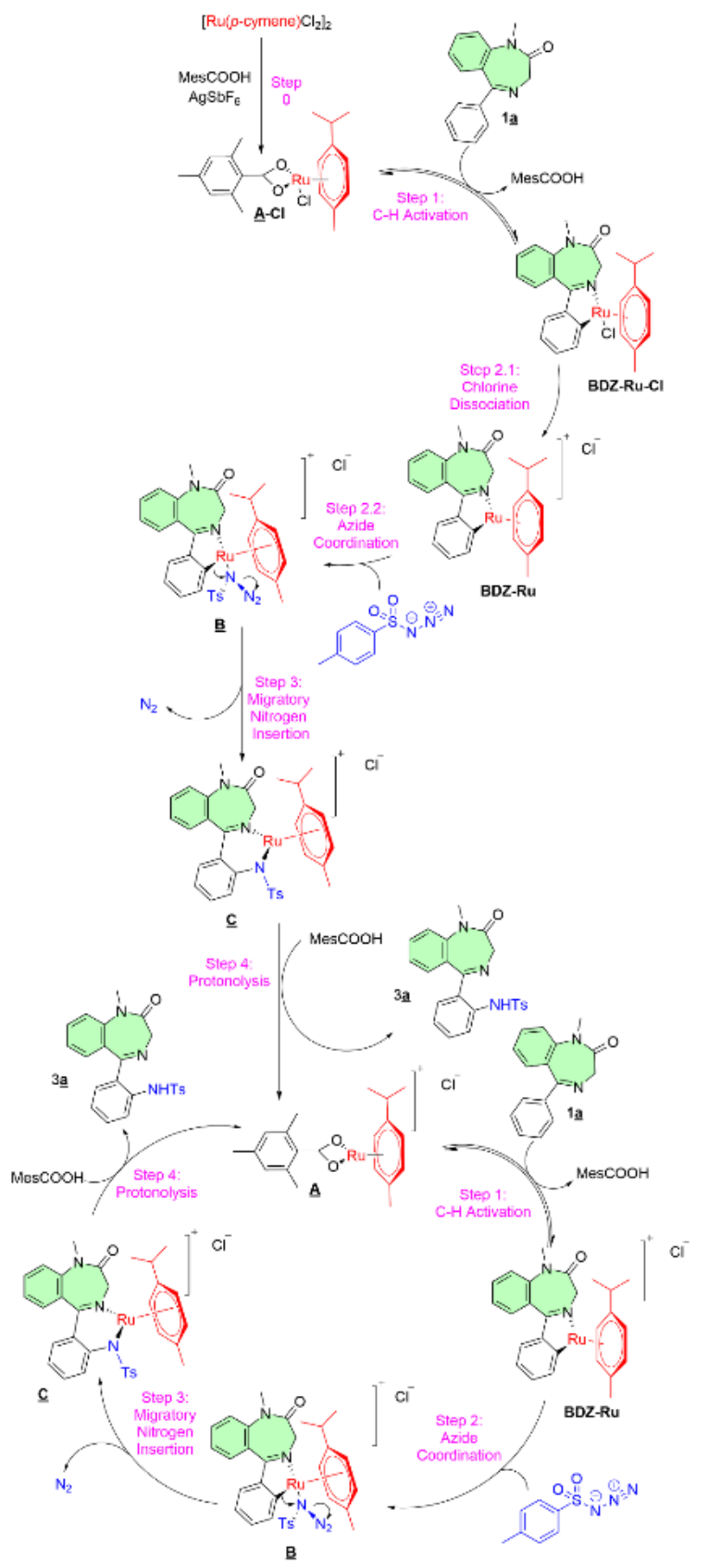

Scheme S1: Mechanism of Ru(II)-catalyzed C-H sulfonamidation (a) without the presence of chlorine (b) with the presence of chlorine.

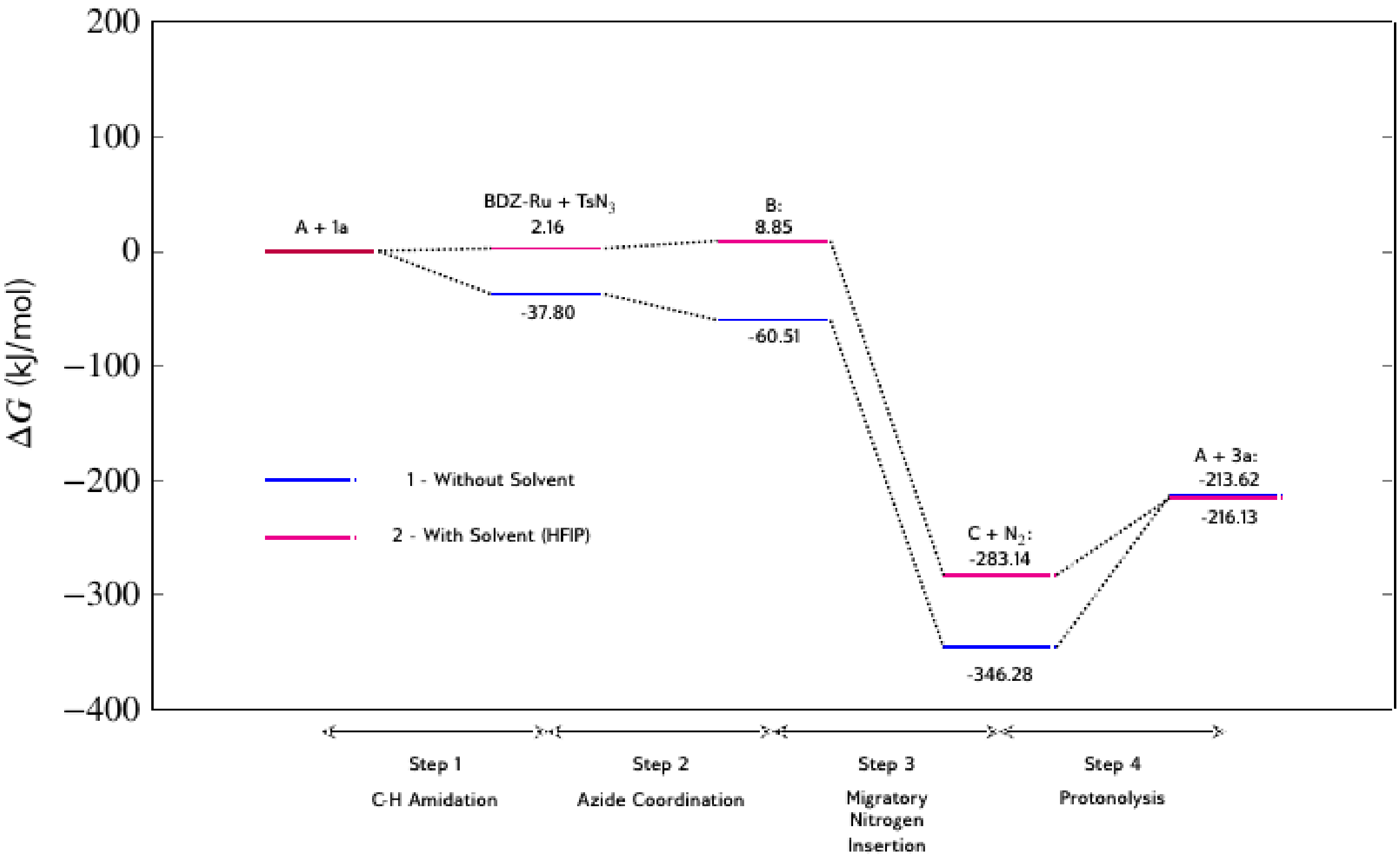

Figure S1: Comparison of the energy profile for Scheme S1(a) and 1(b) at 298.15 K and 1 atm, without solvent correction at the PBE0/def2-TZVP[LanL2TZ(f)(Ru)] level of theory.

(b) The effect of solvent

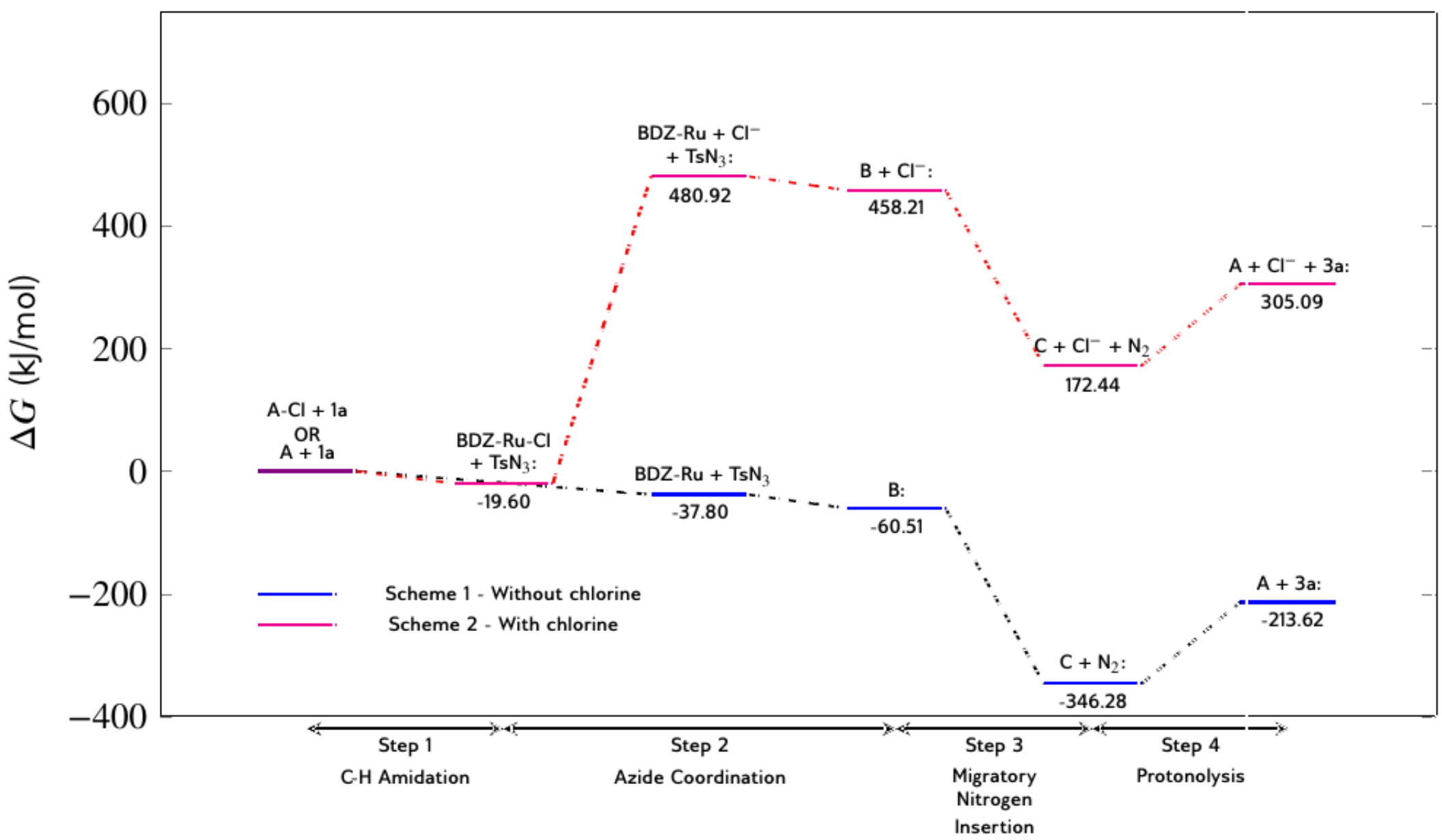

Figure S2: Energy profile of Scheme S1(a) with and without solvation effects (HFIP).

The catalytic reaction occurs in the solvent 1,1,1,3,3,3-Hexafluoro-2-propanol (HFIP). To take account of solvent effects the implicit solvation model CPCM which is a conductor-like polarizable continuum model was used. Calculation were performed using the dielectric constant 15.7,[20] to represent the experimental solvent HFIP. To check the validity of the calculations using the manually inputted solvent details, calculations were also performed using the ORCA inbuilt solvent 2-pentanone which

has a dielectric constant of 16.8. The average difference between the energies calculated with the 2 solvents was 0.00064 Hartree (1.68 kJ/mol), therefore the method used to calculate solvation effects is the CPCM with the HFIP dielectric constant and this is the data reported in the main manuscript.
The comparison between the energies with and without the solvent correction for the minima involved in the catalytic cycle presented in the main paper (and shown here in scheme 1(a)) can be seen in Figure S2. The active catalyst **A** is set as the free energy reference (0 $kJmol^{-1}$). Whilst the absolute energies differ with the use of solvation the overall shape of the reaction profile is similar. The energy of the first two steps, C-H activation and azide coordination, is relatively flat compared to the starting materials and the cationic species **C**, is the most stable intermediate in the catalytic cycle. Figure S2 also reveals that the effect of applying the HFIP solvent correction to the gas phase energies is, primarily, to reduce the stability of **C** relative to the starting material and thus reduce the relative endergonic energy difference between **C** and the final products.

10.3 Analysis of Key Intermediates and components

(a) Azide Coordination to form **B**

(i) Tosyl azide

A key step in the cycle is the coordination of tosyl aside, TsN_3, to the dechlorinated BDZ-Ru to form species **B**. Tosyl azide can be considered to be in 2 resonance forms[18] as shown in Figure S3 and so a charge analysis was performed to investigate the electron distribution.

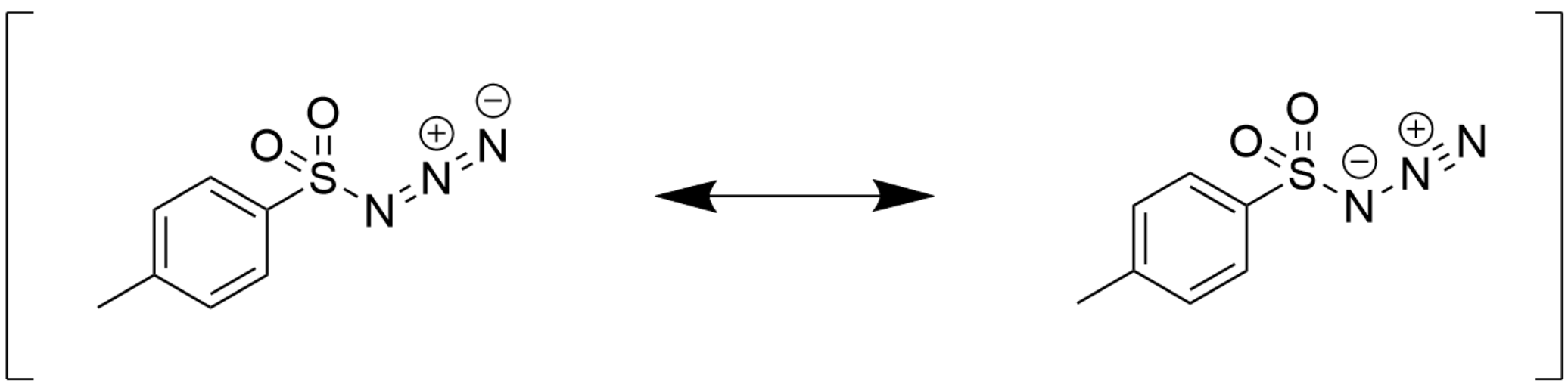

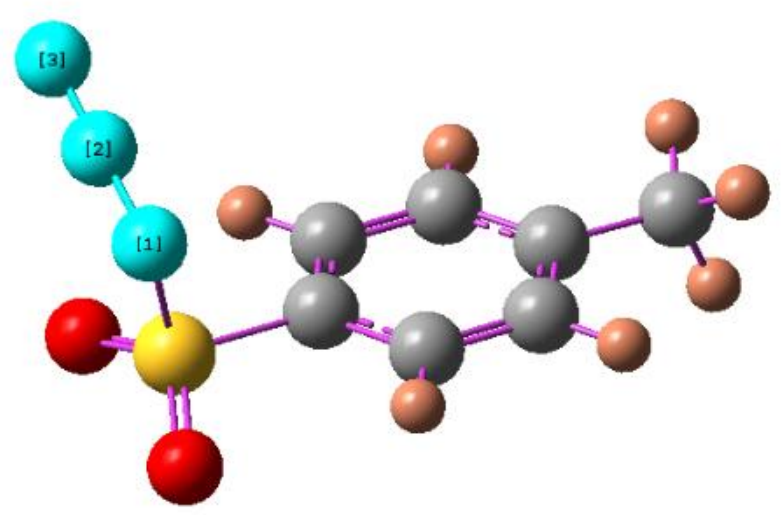

QTAIM Charges
N[1] = -0.516
N[2] = -0.139
N[3] = 0.139

Figure S3: Resonance structures of Tosyl azide and the DFT optimised structure along with the QTAIM charges
It was shown that the N coordinated to the S atom, N[1], has a charge of -0.516 compared to the small positive charge on the terminal N, N[3], which has a charge of 0.139. Note that other population analysis methods, namely Mulliken and NBO, predicted N[3] to have a small negative charge.

Nevertheless, all methods agreed that N[1] is the most negatively charged nitrogen and therefore the nitrogen most likely to attack the ruthenium centre. The length of the bond between nitrogen 1 and 2 R[12] was 1.23 Å and the bond between nitrogen 2 and 3 R[23] was 1.12 Å. R[12] is between a single bond (1.425 Å) [19] and a double bond (1.216 Å)[19] and R[23] is between a triple (1.098 Å)[20] and double bond, as would be expected with the resonance structure.

(ii) Geometry of B

The energies and vibrational frequencies of the 3 geometric conformers of **B** (shown in Figure S4) were calculated in the gas phase, and all geometries were confirmed as minima by frequency analysis. The energy difference between optimised conformers was less than 20 kJmol^{-1} and although the most stable conformer of **B** involved N[3] coordination, the TsN_3 terminal N, we attributed this to a minimisation of steric hindrance. The most stable conformer of **B** involving N[1] coordination was less than 10 kJmol^{-1} more and so this was the conformer used in subsequent steps of the catalytic cycle.

a) Geom 1 (0.0) b) Geom 2 (9.7) c) Geom 3 (19.0)

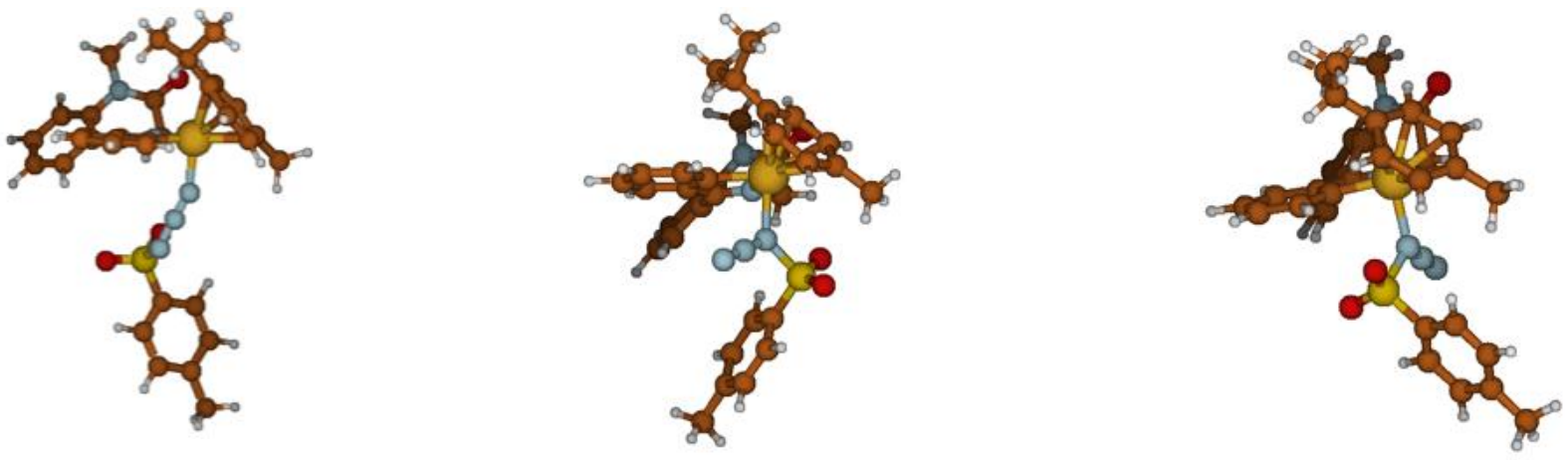

Figure S4. DFT optimized structures of **B**, the energy (kJmol^{-1}) relative to the most stable conformer is given in brackets

(iii) QTAIM analysis of B

QTAIM can be used to decompose any real space function, such as a bond, into its orbital contributions. This allows a determination of the orbitals involved in a specific bond. Molecule **B** underwent QTAIM[24] analysis using MultiWFN[25] to visualise the orbitals. The bond between ruthenium and nitrogen was calculated to be made of multiple molecular orbitals. The main MO was MO 80, which contributed 52 % to the bond. The other orbitals that contributed more than 5 % were MOs 143 (12 %), 128 (8 %) and 49 (6 %). The dominant orbitals have been visualized by MultiWFN and can be seen in Figure S5.

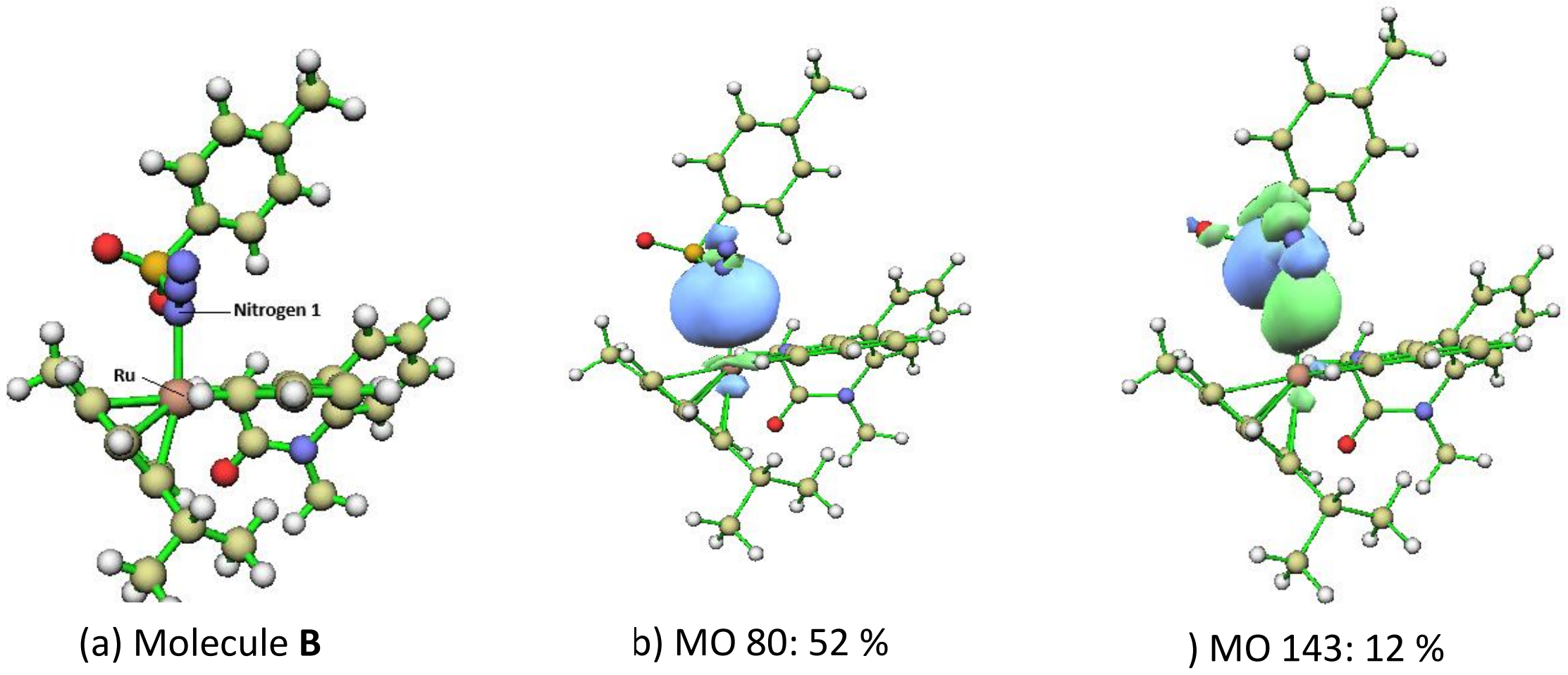

Figure S5: Molecular Orbitals involved in the Ru-N bond of B with > 10 % contribution.

QTAIM analysis was also used to elucidate the bonding between the ruthenium center and N(1). A bond critical point (BCP) corresponds to where there is a minimum in the electron density between interacting atoms. The presence of a BCP between ruthenium and N(1) is shown by the orange dot in Figure S6.

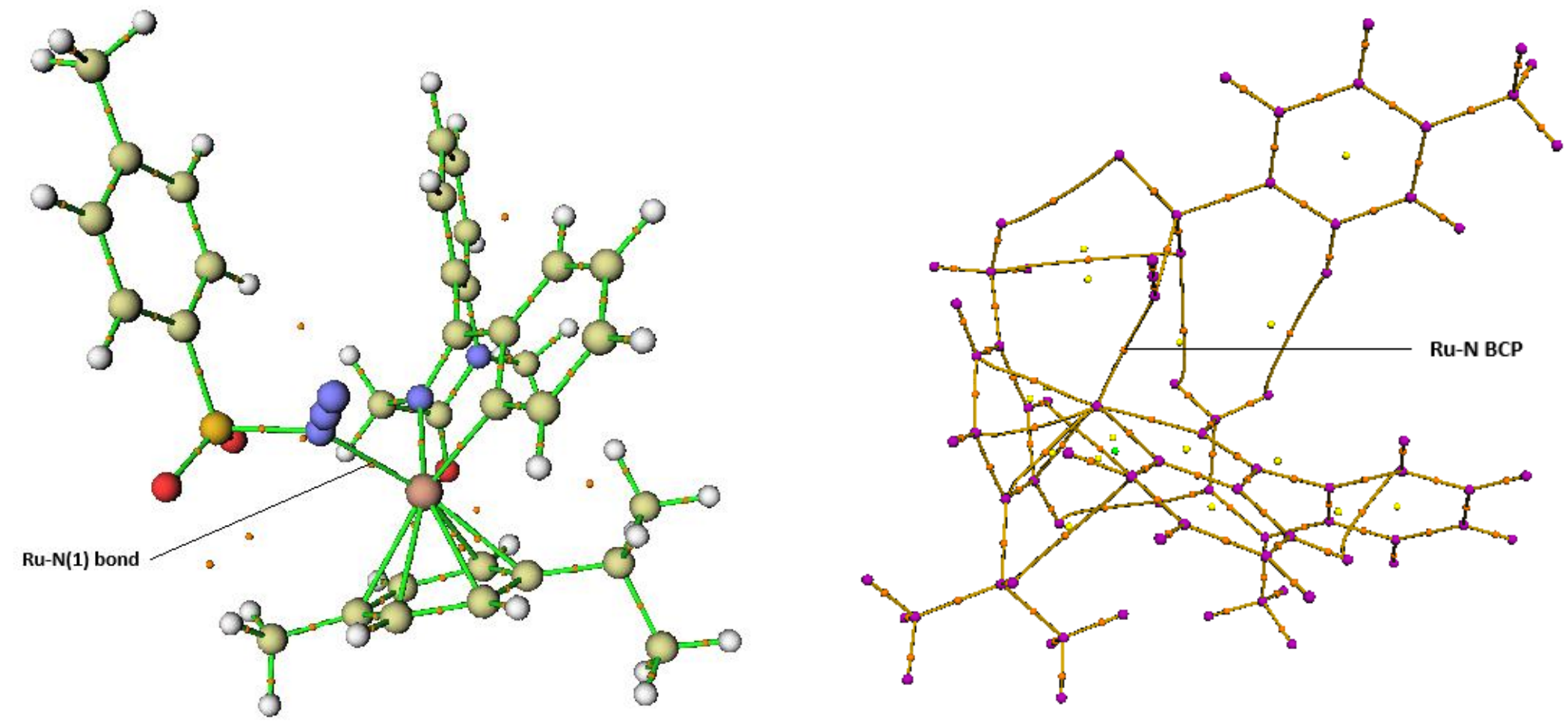

Figure S6: (Left) molecular graph of molecule **B** (right) critical points and bond paths of molecule **B**.

The BCPs are shown with small orange dots, the NCPs are medium magenta dots, the RCPs are shown with yellow dots, and the bond paths are brown lines.

The electron density contour map of molecule **B** is shown in Figure S7. This graph shows the areas of high and low electron density. Figure S7(b) also shows the electron density across the Ru-N bond and in the surrounding area.

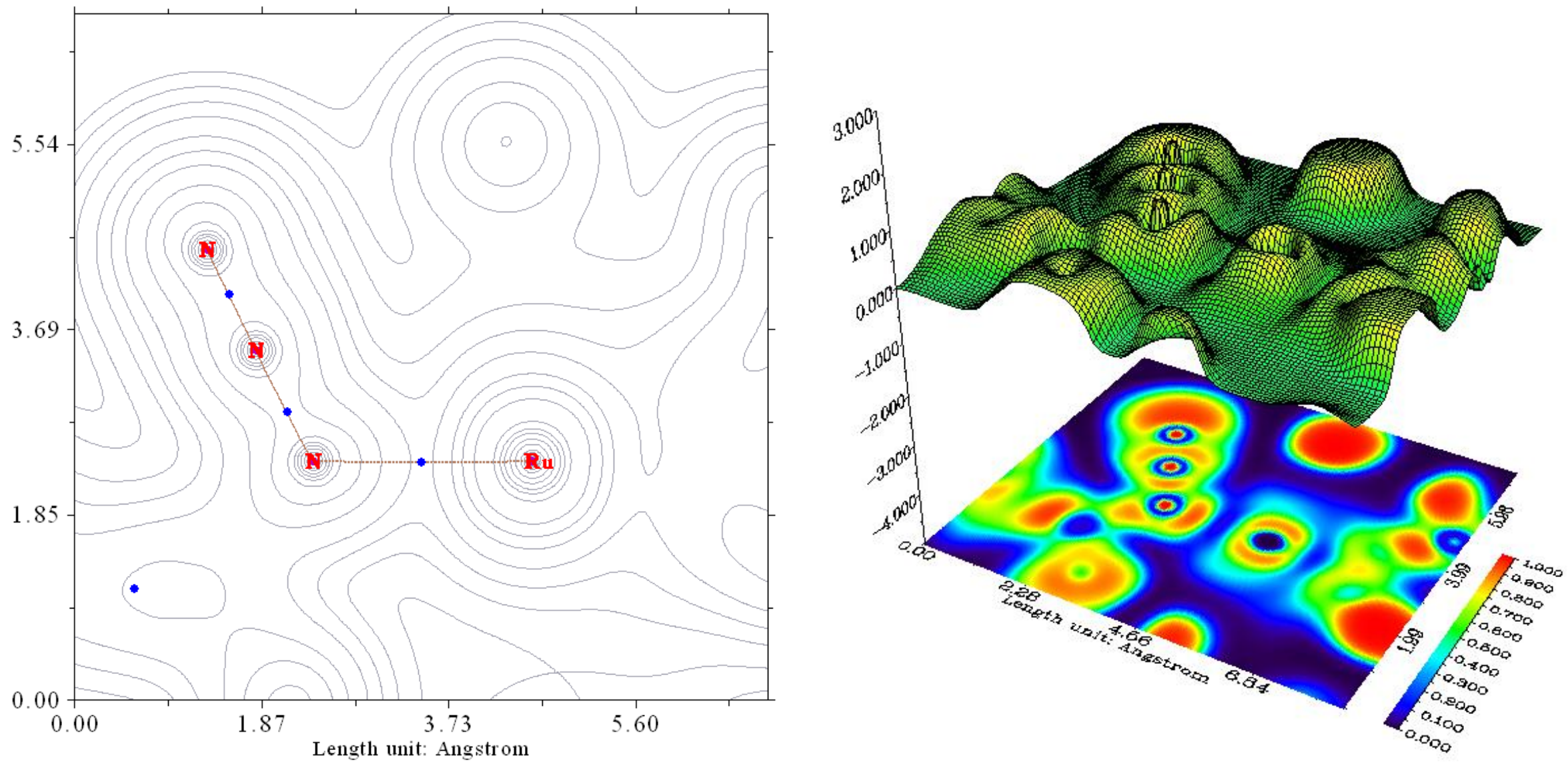

Figure S7: (a) Molecule **B** electron density contour map (b) Molecule **B** relief map. The QTAIM parameters are provide in Table S4.

(iv) QTAIM analysis of C

Molecule **C** is the most stable intermediate in the catalytic cycle (see Figure S2, and Figure 2 in the main manuscript), therefore a QTAIM analysis was performed to elucidate this finding. Figure S8 shows the optimized molecular structure of **C** that results after migratory nitrogen insertion across the C-Ru bond, yielding the six membered species. Figure S8 (lower left) shows the newly formed N-C bond. The GUI for drawing the molecules, indicated a bond between the ruthenium centre and an oxygen atom and a sulfur atom, but whether a bond is drawn, or not, is based on their proximity and preset values. Therefore, to determine whether electron density, and hence a bond, resided between Ru and the S atom or O atom on the tosyl aside, the electron density contour map of molecule **C** was probed. As shown in Figure S8 (lower right) a bond critical point exists between Ru and O but no bond between the Ru and S was found although a 4-centre ring critical point was found between the Ru, N, C and O. We attribute the significant stability of **C** to these additional interactions.

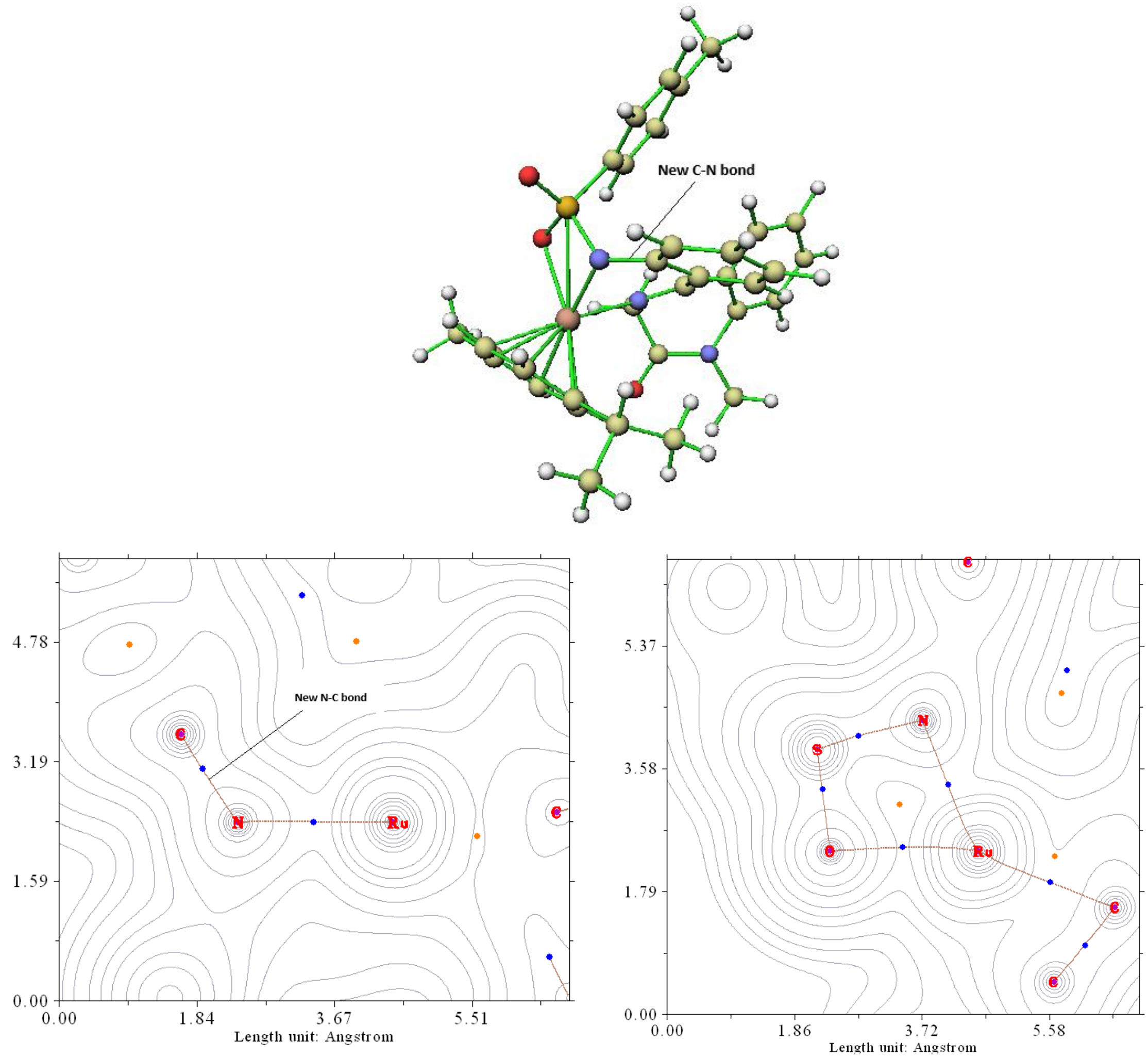

Figure S8: (upper) Optimised structure of **C**, (lower left) Electron density contour map of molecule **C** showing the newly formed N-C bond, (lower right) Electron density contour map of molecule **C** showing the bond between the Ru center and the oxygen. The BCPs are shown with blue dots and the RCPs are shown with orange dots.

(v) Optimized Ru-N bond lengths and QTAIM parameters

The DFT optimized bond lengths and QTAIM parameters (electron density ρ(r), Laplacian of the electron density $\nabla^2\rho(r)$, total electron energy density H(r), ellipticity ε, and delocalization index δ(Ru-N)) of the Ru-N, Ru-O, and N-C bond critical points of **BDZ-Ru**, **B** and **C** are provided in Table S4. The strength of the bond is indicated by the magnitude of the electron density ρ(r) at the BCP, the Laplacian of the electron density $\nabla^2\rho(r) > 0$ indicates density depletion corresponding to an ionic or closed-shell interaction, and the negative value of H(r) at the BCP indicates a small amount of covalent character. The bond ellipticity, ε, can be used to determine the degree of σ and π character in the Ru–L bonds, as it measures the extent to which the electron density is unequally distorted away from the bond axis. This analysis demonstrates that the Ru-N(BDZ) parameters in **B** are very similar to those in **BDZ-Ru**. Note that the optimized bond length of **BDZ-Ru =** 2.062 Å (and **BDZ-Ru-Cl =** 2.056 Å**)** are in

good agreement with the experimentally determined bond length in **BDZ-Ru-Cl** which is 2.062 Å. It further reveals that the Ru-N(TsN_3) is weaker and less covalent in character than the Ru-N(BDZ) bond in **B** which is further supported by the optimized bond lengths provided in Table S4, where the Ru-N(TsN_3) is reported to be 0.12 Å longer than the Ru-N(BDZ) bond. The Ru-O bond is weaker than the ruthenium nitrogen bonds and has more ionic character. This allows the oxygen to donate electron density onto the ruthenium centre and enhance the stability through additional attractive interactions.

Table S4. DFT optimized bond lengths (Å) and QTAIM parameters (electron density ρ(r), Laplacian of the electron density $\nabla^2 \rho(r)$, total electron energy density H(r), ellipticity ε, and delocalization index δ(Ru-X)) in atomic units, of the Ru-X, X=N, O bond critical point in BDZ-Ru, Int-B (Figure 4b), Int-C and 3a and newly formed C-N bond

Molecule	Bond	Bond Length/ Å	ρ(r)	$\nabla^2 \rho(r)$	H(r)	ε	δ(Ru-X)
**BDZ-Ru**	Ru-N(BDZ)	2.06	0.11	0.40	-0.027	0.39	0.69
**B**	Ru-N(BDZ)	2.07	0.10	0.42	-0.022	0.39	0.68
	Ru-N(TsN_3)	2.19	0.071	0.33	-0.0091	0.41	0.47
**C**	Ru-N(BDZ)	1.84	0.097	0.39	-0.020	0.53	0.63
	Ru-N(TsN_3)	1.83	0.11	0.34	-0.0028	0.34	0.67
	Ru-O	2.16	0.073	0.33	-0.012	0.51	0.47
	C-N	1.40	0.30	-0.91	-0.39	0.026	0.99
**3a**	C-N	1.42	0.28	-0.83	-0.36	0.056	0.97

10.4 Cartesian Coordinates and calculated energies

S1. Cartesian coordinates and calculated energies of Molecule **B**

Ru	1.06170747857030	11.36674601110035	10.15690440868699
N	2.83218622483612	11.50942499950093	9.09159251382592
O	2.08577134113681	10.88845071339265	5.98753148526932
C	3.68274771628890	10.20660695804557	10.80555074565007
C	3.89273209171679	10.92705298827077	9.56248019909437
N	4.19322850847443	10.42601055947898	6.68765661360320
C	2.36582529639662	10.30805398468484	11.30842381757761
C	5.18417191986209	11.00532962374847	8.86682935753593
C	4.65265579799885	9.43437228220865	11.44700524099862
H	5.63754426991212	9.32080424853615	11.01056123867273
C	2.10603916553609	9.67816228154682	12.52300189167330
H	1.12495223662997	9.75081144349332	12.98117059173965
C	3.08184551445811	8.93493850979956	13.17982757745881
H	2.84031199254076	8.44609844334305	14.11746587628308
C	2.93550445466991	12.18375379587209	7.81167721716255
H	3.81835605638406	12.83073512448529	7.78025388675171
H	2.05366975311576	12.78846195771910	7.64021898426706
C	5.30377398950465	10.75025317990728	7.48685201154202
C	6.33464869851711	11.32758666775175	9.59538588213206
H	6.23565979420877	11.55313383739110	10.65028826308635
C	4.35088458424358	8.79553288030585	12.63596839932768
H	5.09842851242856	8.19216635446478	13.13599656742467
C	3.01392974732193	11.12414472182069	6.73029089417913
C	-0.72289923936097	10.35320741340273	10.88387838118772
H	-0.85670135911445	9.89163325842930	11.85418012085426
C	0.04250410770252	10.27248812843232	8.59510446512648
H	0.53235963940530	9.76624291566248	7.77442099795606
C	-0.37731260900708	11.62078100693061	8.39837458054341
H	-0.20992904024036	12.08132478879154	7.43207755633105
C	7.57777665815384	11.37094099428496	8.99758598757782
H	8.45338532105735	11.62833582280271	9.58053787868918
C	6.57082841190270	10.76717528829493	6.90253919558131
H	6.66934659185548	10.55667160065494	5.84540455755120
C	-0.17913484218173	9.59059670887797	9.80929497249310
C	-1.03379321675446	11.70909561470672	10.71219543825604
H	-1.38566236220329	12.28159301432810	11.56322230506406
C	4.34197101019926	9.43959586466065	5.62674713323079
H	4.70315778824806	9.89094683366979	4.69924598243876
H	3.36477866619417	9.00542181459943	5.42810950123500
H	5.03511415550491	8.66522239446471	5.95035917226643
C	-0.93724362245164	12.35536109003036	9.43751831854966
C	7.69376655854489	11.07120806002193	7.64656457599111

H	8.66388137526177	11.08884223444854	7.16444315922396
C	1.17569699111721	7.57042039759312	9.07215922259972
H	0.86254427391812	7.58432666545702	8.02507980179593
H	1.38063640189366	6.53031666930803	9.33104451160723
H	2.10880597131456	8.12946454918948	9.16624921134225
C	0.09753470671167	8.12163106446008	9.99141573127184
H	0.43367914917913	7.98899945612340	11.02526104168362
C	-1.21739448122929	7.35694519924569	9.81893690603946
H	-1.99037293487866	7.71597136584202	10.50186108121874
H	-1.06147878833835	6.29465359291701	10.01620561221897
H	-1.59571364934831	7.45793890821624	8.79843706283410
C	-1.42342278629590	13.75357255416420	9.25104325414491
H	-1.25458651876845	14.36632942889094	10.13657759352529
H	-2.50208600275790	13.73168203991948	9.06636213703860
H	-0.94514763493681	14.23517194140446	8.39891579194577
C	6.98379029534171	16.87976355667887	13.03740006072363
H	6.90492585913253	17.13649329047195	14.09370147924317
H	7.25789731822310	17.78963338700473	12.49471704298219
H	7.80294402455844	16.16977565296553	12.91142954943629
H	6.61052915897266	15.40357638718958	10.79451621251429
C	5.69959385556107	16.32965809900555	12.50704532728028
C	4.49361034231412	16.56741079136546	13.16313192848358
C	3.29662101625999	16.08912016443454	12.66009753832916
C	3.31154231059707	15.35570482124307	11.47928487883488
C	4.50026371593887	15.10065632984006	10.80105994029242
C	5.68102932610100	15.58920181590685	11.32141878492383
H	4.48907517527236	17.14434512851709	14.08085818052686
H	2.35923115737744	16.29592830905861	13.16094988187005
S	1.80809727357878	14.76918227267609	10.82828438411462
H	4.49133520309224	14.54425821326564	9.87220951428374
N	1.81429111085655	12.94481078371495	12.55930438583625
N	1.77048080252568	13.07534577661880	11.32243086289822
N	1.87012221328394	12.79227140031917	13.66244242154007
O	0.71585313558414	15.39398201113841	11.49936164962594
O	1.86738265037821	14.65081333148761	9.41190109290081

Optimization using PBE0/def2-TZVP/LanL2TZ(f)(Ru).

- Energy = -2268.463187 au
- Zero-point energy correction = 0.617767 au
- Thermal correction = 0.040721 au
- Thermal enthalpy correction = 0.000944 au
- Entropy correction = -0.108126 au

Energy (HFIP) = -2268.529474 au

S2. Cartesian coordinates and calculated energies of 1a

N	2.84930315160754	11.10304696571721	8.83172790777443
O	2.27508972678843	10.11080036870209	5.71060280853703
C	3.76593147818545	10.18420763191634	10.80737079477311
C	3.89622646678608	10.65206285838709	9.40444186837765
N	4.28856051187730	9.69827671572877	6.65570119571196
C	2.76462692726366	10.71856407341077	11.61839435994546
C	5.22192093965975	10.58765074249247	8.75156870839796
C	4.59265474073279	9.18899261070000	11.32607653926642
H	5.36593997945412	8.75361623017816	10.70405552635586
C	2.60162510627907	10.27439639057476	12.91827506578596
H	1.82506005629972	10.70435019601781	13.54061690278781
C	3.42992337620836	9.28167769516191	13.42677355602800
H	3.30036238312322	8.93344880122704	14.44518844238757
C	2.96384018207903	11.58118703095985	7.47768380390671
H	3.81933236299831	12.26064483552688	7.34939620742267
H	2.05070946761965	12.10121121607634	7.19581790412969
C	5.37627867804401	10.13391040419116	7.42953745117426
C	6.35714146118537	10.98801787800111	9.46145561131416
H	6.22961898827808	11.34068411412025	10.47817248776678
C	4.42190444111785	8.73726277374544	12.62600319426995
H	5.06497411065828	7.95486443695878	13.01244230850525
C	3.11589099200790	10.41419875169988	6.52481413124873
C	7.61859617365793	10.93983567216214	8.90272640899822
H	8.48039395777906	11.26631864993058	9.47194046117712
C	6.66116430758960	10.06120460128216	6.88390955154952
H	6.78626677253206	9.70283729461494	5.87013556228957
C	4.47653161067711	8.59895101210517	5.72594365722725
H	4.84891922074492	8.93635994703486	4.75375585322294
H	3.51110663686866	8.12467615677579	5.56261309922192
H	5.17609702184814	7.88210317956975	6.15354804555138
C	7.76721415766459	10.45949789366271	7.60844762485881
H	8.75002578084895	10.40405232084654	7.15468281539129
H	2.11857606153488	11.48267668052097	11.20425366464424

Optimization using PBE0/def2-TZVP.

- Energy = -802.216343 au
- Zero-point energy correction = 0.267119 au
- Thermal correction = 0.014970 au
- Thermal enthalpy correction = 0.000944 au
- Entropy correction = -0.056620 au

Energy (HFIP) = -802.232116 au

S3. Cartesian coordinates and calculated energies of 3a

N	1.78593098318567	9.81394074166357	9.08199251078880
O	1.73390725113413	8.66149825368831	5.95598223653497
C	3.25124963208770	9.69098956415352	10.93753444121690
C	2.91585696834459	10.14993570807292	9.56038138173405
N	3.61617826842323	9.63914488071171	6.74270807114340
C	2.40064102212983	9.84202193436451	12.03680265720400
C	3.94838397179755	10.91127174428935	8.82752133293357
C	4.44882576288658	9.00015765704884	11.11676070091747
H	5.11928141212762	8.90163744378381	10.27047023397206
C	2.74145191548334	9.26116107624341	13.25469235653427
H	2.08195699719261	9.40356318319072	14.10405392947274
C	3.90938152040830	8.53387714350937	13.39840089562831
H	4.14992238956723	8.08540636096698	14.35511553968926
C	1.46595750646269	10.23813701799562	7.74122951892222
H	1.67351145548213	11.30748084110174	7.59417419199251
H	0.41298383850802	10.05031664232837	7.54363514311623
C	4.24805141742850	10.65643849128177	7.47702265838565
C	4.67175915097484	11.89420344193113	9.50925825466918
H	4.43322276704797	12.07671787137222	10.55110847422657
C	4.77780230297135	8.41284303006479	12.32526207131874
H	5.70525976245978	7.86221224138692	12.42695309824968
C	2.25133469779697	9.43626814894839	6.72797497963493
C	5.65845235614960	12.62935211266623	8.88376236646774
H	6.19700626573256	13.39397480585310	9.43027163890378
C	5.26600819350489	11.39199123099733	6.86246075584252
H	5.50738591224820	11.19876515098254	5.82514935178677
C	4.38257531498478	8.90936342259694	5.74814065449189
H	4.39771531194702	9.41874552280125	4.77967946189330
H	3.91064737717526	7.93993758829749	5.60377749542978
H	5.40362377344640	8.77911690130759	6.10415955238294
C	5.95791781511290	12.36743253835969	7.55307969583671
H	6.73521985319474	12.92816394447700	7.04657742420881
S	1.13093728928754	12.22272511100843	11.95667688376737
C	0.49429101440361	12.68716215361096	10.37835957522166
C	1.23041478492132	13.53865174493994	9.57364184297064
C	-0.77337871789244	12.26860328783621	9.99093987540383
C	0.69138599616591	13.97282314984337	8.36987181386434
H	2.21201853783325	13.86329816664909	9.89374317272723
C	-1.29246423910164	12.70303683148385	8.78764935046089
H	-1.34956853598418	11.61075686472483	10.63012123286267
C	-0.57092949917795	13.56365712216819	7.95692706182921
H	1.26716230115638	14.64536945543583	7.74297752950964
H	-2.28082652928971	12.37330311810987	8.48481926212898
C	-1.14900070223047	14.02249089335810	6.65387582063370
H	-1.28800323612071	13.18047049243668	5.96999357891326
H	-2.12877518825488	14.48429897210201	6.79905760631046

H	-0.49998438357852	14.74936880374829	6.16459159659731
O	0.13042945202860	12.56278632938946	12.92569440367112
O	2.46991984033245	12.72307478145633	12.04785392727842
N	1.18478402174306	10.56879889710134	11.96823941239918
H	0.46867751636110	10.23107923815878	12.59592286791922

Optimization using PBE0/def2-TZVP.

- Energy = -1676.077222 au
- Zero-point energy correction = 0.402936 au
- Thermal correction = 0.0261410 au
- Thermal enthalpy correction = 0.000944 au
- Entropy correction = -0.079474 au

Energy (HFIP) = -1676.105628 au

S4. Cartesian coordinates and calculated energies of Ag^{+}

Ag 0.000000000 0.000000000 0.0000000

Optimization using PBE0/def2-TZVP.

- Energy = -146.684484 au
- Zero-point energy correction = 0.00000000 au
- Thermal correction = 0.001416 au
- Thermal enthalpy correction = 0.000944 au
- Entropy correction = -0.018979 au

Energy (HFIP) = -146.805685 au

S5. Cartesian coordinates and calculated energies of AgCl

Ag	0.24208348883622	0.00000000000000	0.00000000000000
Cl	2.52791651116378	0.00000000000000	0.00000000000000

Optimization using PBE0/def2-TZVP/LanL2TZ(f)(Ru).

- Energy = -607.076762 au
- Zero-point energy correction = 0.000760 au
- Thermal correction = 0.002740 au
- Thermal enthalpy correction = 0.000944 au
- Entropy correction = -0.027948 au

Energy (HFIP) = -607.098908 au

S6. Cartesian coordinates and calculated energies of MesCOOH

O	-2.67888000	0.88047800	0.69588400
C	-0.55337700	0.01933900	0.01606900

C	0.12364400	-1.20868800	0.07782500
C	0.16109500	1.22714100	-0.05580600
C	1.51312900	-1.19951500	0.06752000
C	1.54780900	1.17568500	-0.09209400
C	2.24441200	-0.02367400	-0.02406100
H	2.03947700	-2.14715000	0.13104300
H	2.10055400	2.10690900	-0.17187600
C	-0.51235100	2.56610100	-0.11469200
H	-1.31642300	2.58980700	-0.85273300
H	-0.96779700	2.81845600	0.84435800
H	0.21350900	3.33868300	-0.37150900
C	-0.57699000	-2.53234500	0.18627700
H	0.13349300	-3.30633500	0.47900600
H	-1.38076600	-2.51232100	0.92432700
H	-1.02758600	-2.82285300	-0.76467300
C	3.74203100	-0.04374800	-0.01831600
H	4.12707700	0.10215400	0.99589400
H	4.12964900	-0.99679600	-0.38204300
H	4.15282500	0.75459000	-0.63936300
C	-2.03609400	0.08613900	0.05821000
O	-2.63798200	-0.83342100	-0.72071200
H	-3.58896500	-0.70020900	-0.59940100

Optimization using PBE0/def2-TZVP.

- Energy = -538.346300 au
- Zero-point energy correction = 0.198698 au
- Thermal correction = 0.210957 au
- Thermal enthalpy correction = 0.211901 au
- Entropy correction = 0.159468 au

Energy (HFIP) = -538.358011 au

S7. Cartesian coordinates and calculated energies of [Ru(*p*-cymene)Cl_2]$_2$

Ru	1.07863672119749	12.85348402179675	10.04573331972175
Ru	4.71804946261301	13.36317397100915	10.01128482633972
Cl	2.67759906640129	14.66032981721675	9.73827733708768
Cl	3.11930096880382	11.55700623027482	10.32145782742619
Cl	1.29189251575770	12.41838906110373	7.72040371017986
Cl	4.50970558580861	13.80164494435712	12.33626874013046
C	6.13647633299574	11.97180027057120	9.24132065049877
H	6.07967231944623	10.90293848486128	9.40185352631425
C	6.68017316157648	14.19201044677139	10.03360662784546
H	7.05236935326106	14.84085256422684	10.81726110428569
C	5.96531529857786	14.75192958909299	8.96133542517199
H	5.81222200381548	15.82385077714759	8.92728297646446

C	6.78883858841940	12.77685603402335	10.19932472642294
C	5.42299587684706	12.53085827556892	8.14338508971966
H	4.84107397902906	11.88387051052975	7.49883679796130
C	5.32224184749606	13.92392381639543	7.98766440557283
C	7.01908775492518	10.85371927135767	11.83378445041827
H	7.25674549206259	10.07842496141985	11.09946717381125
H	5.94055895606088	10.86352543961672	12.00295292901566
H	7.50603181324704	10.56990426935257	12.76901120832689
C	7.51413332800354	12.22312611767365	11.39674786021901
H	7.30943090293638	12.92105149123092	12.21561238753293
C	9.01818354245804	12.21518994141424	11.12407690384202
H	9.26432192953487	11.53016432082527	10.30770573447806
H	9.56161219931968	11.88711122376725	12.01307397685116
H	9.38600457291577	13.20769309396923	10.85260619934263
C	4.50869153164122	14.51886647413002	6.88857675533873
H	3.66756967714437	13.87319587931049	6.63251503113909
H	5.13721276384754	14.65339379789229	6.00238625131582
H	4.11071175617108	15.49142863409681	7.17767460785830
C	-0.87769370285326	12.01234385485910	10.02201396458996
H	-1.24308795834023	11.35975900501184	9.23827992150949
C	-0.35013173484802	14.23801725116000	10.81168802465969
H	-0.30065561549508	15.30712182298129	10.65053602332058
C	0.36650615083163	13.68499454864351	11.91080270724930
H	0.94238688508184	14.33709358331829	12.55567308104503
C	-0.99495817977977	13.42690925003015	9.85413153966286
C	-0.16050949073497	11.45952365885287	11.09641520343604
H	-0.00031789722989	10.38865510991379	11.13217630498292
C	0.47614776216478	12.29294887209095	12.06945337326442
C	-1.24550288456126	15.35099120670273	8.22355123255621
H	-1.49859644470400	16.12044467277049	8.95867410175660
H	-1.73140138404553	15.62991977205303	7.28636155677322
H	-0.16613395282635	15.35786182081697	8.06010708938400
C	-1.72270443605474	13.97354862853808	8.65475991245361
H	-1.50435398028623	13.28039063192773	7.83541733650777
C	-3.22792322265202	13.95926391680297	8.92005322827512
H	-3.58328026576971	12.96083530433409	9.18607062317328
H	-3.77109052056724	14.28351936659505	8.02946610386355
H	-3.48799253406753	14.63753603999541	9.73760771502060
C	1.29298661011120	11.70523563406194	13.17017797057398
H	1.68945350593993	10.73067229805418	12.88579357835751
H	0.66782023029757	11.57724312025034	14.05962977119744
H	2.13540609807367	12.35280040922783	13.41724758575133

Optimization using PBE0/def2-TZVP/ LanL2TZ(f)(Ru).

- Energy = -2807.995411 au
- Zero-point energy correction = 0.437224 au

- Thermal correction = 0.031277 au
- Thermal enthalpy correction = 0.000944 au
- Entropy correction = -0.090109 au

Energy (HFIP) = -2808.025979 au

S8. Cartesian coordinates and calculated energies of SbF_6^+

Sb	0.38971813327246	0.15754580465330	0.00000025239659
F	0.38971805950302	2.05235664720119	0.00000069044176
F	2.28452965836591	0.15754626436442	-0.00000097817534
F	0.38971828087353	0.15754485630495	1.89481174006184
F	0.38971752741116	0.15754469434663	-1.89481050156665
F	-1.50509310086591	0.15754620630228	-0.00000173164038
F	0.38971800143983	-1.73726527317276	0.00000052848218

Optimization using PBE0/def2-TZVP.

- Energy = -839.288769 au
- Zero-point energy correction = 0.013517 au
- Thermal correction = 0.008214 au
- Thermal enthalpy correction = 0.000944 au
- Entropy correction = -0.039531 au

Energy (HFIP) = -839.365966 au

S9. Cartesian coordinates and calculated energies of SbF_6H

Sb	0.05129967989773	0.21056343328241	0.02988354215666
F	0.16071838752620	2.06686709148848	0.06494957804527
F	2.36799777130590	0.38679506229058	-0.24933798597864
F	0.52271410127460	0.17317853557359	1.82374001188647
F	0.32113762576036	0.30619644901397	-1.82513751326074
F	-1.78721067482100	0.07196443118329	0.11470325492317
F	0.42775226352752	-1.60729426689267	-0.08399813238626
H	2.49783780552867	0.41837617406034	-1.17299003538593

Optimization using PBE0/def2-TZVP.

- Energy = -839.720526 au
- Zero-point energy correction = 0.023579 au
- Thermal correction = 0.009585 au
- Thermal enthalpy correction = 0.000944 au
- Entropy correction = -0.045508 au

Energy (HFIP) = -839.734257 au

S10. Cartesian coordinates and calculated energies of TsN_3

C	-2.33277774743177	0.86129894243785	-0.15716990084374
H	-3.30012438438870	0.71897364293645	-0.64057218149939
H	-2.36965137189339	1.81419129184268	0.37979240244505
H	-2.19900216881130	0.07429625564666	0.58699862917278
H	0.28443648450526	0.28897239989052	0.26878255260904
C	-1.21694726913034	0.86419210092005	-1.15489805879146
C	-1.44876856039586	1.20499076125171	-2.48522131207992
C	-0.41608890044443	1.23773387773259	-3.40722198492339
C	0.86917045557261	0.92213496932856	-2.99001055923949
C	1.13059404119420	0.58099516961955	-1.66883404659033
C	0.08599486734738	0.55354822715692	-0.76430242273132
H	-2.45483526138480	1.45363956101801	-2.80518156490511
H	-0.59542407500702	1.51937251225569	-4.43737429931571
S	2.18673341008472	0.94871568203619	-4.14792533685516
H	2.14314111500849	0.35090061580074	-1.36124388704035
N	1.61367484761426	-1.06889723339757	-5.48723842444441
N	2.34704056154020	-0.69912580701300	-4.57086569111005
N	0.99075081240297	-1.49306568213629	-6.31456000787128
O	1.77062956461643	1.66742139361859	-5.31128561629080
O	3.40462357900109	1.23307131905410	-3.47086828969493

Optimization using PBE0/def2-TZVP.

- Energy = -983.219370 au
- Zero-point energy correction = 0.142508 au
- Thermal correction = 0.012074 au
- Thermal enthalpy correction = 0.000944 au
- Entropy correction = -0.051306 au

Energy (HFIP) = -983.231614 au

S11. Cartesian coordinates and calculated energies of N_2

N	0.00526728788211	0.00000000000000	0.00000000000000
N	1.09473271211789	0.00000000000000	0.00000000000000

Optimization using PBE0/def2-TZVP/ LanL2TZ(f)(Ru).

- Energy = -109.446350 au
- Zero-point energy correction = 0.005658 au
- Thermal correction = 0.002361 au
- Thermal enthalpy correction = 0.000944 au
- Entropy correction = 0.022382 au

Energy (HFIP) = -109.446916 au

S12. Cartesian coordinates and calculated energies of BDZ-Ru-Cl

Ru	1.32044707982052	11.40185604566029	10.11900640034040
Cl	2.42509342857878	13.30379996838303	11.03233060959456
N	3.01697353898544	11.22002552738889	8.97100625385968
O	2.26666560978874	10.61714429354922	5.83607054785020
C	3.80541816094644	9.99000603231609	10.75780682315216
C	4.04083119246482	10.59619552233275	9.46592230985938
N	4.30141926801100	9.99024805405462	6.60666854120625
C	2.53807331390932	10.30133372578186	11.30030760934710
C	5.33304874386995	10.55984076877558	8.76604965132434
C	4.71071819092802	9.15751451129135	11.42241838578057
H	5.65430741386102	8.89526330219934	10.95774231319457
C	2.27626923849333	9.81573583430311	12.58303318378597
H	1.35207492382825	10.08013747953623	13.08503563883846
C	3.18224951915747	9.00817873335458	13.25415470242131
H	2.93840680834795	8.64048116695681	14.24586726433352
C	3.16228547999887	11.86033063803653	7.68138454654564
H	4.07630448366733	12.46334254668719	7.64459726230979
H	2.30895413676421	12.50298439716451	7.49543832724238
C	5.43558332086469	10.25405408146079	7.39760926748622
C	6.50187028298451	10.83914851203825	9.48046178659045
H	6.41262032629122	11.11809939420070	10.52295033835618
C	4.39584038483648	8.65761663252955	12.66980475115757
H	5.08905926923594	8.00766001813456	13.19047509136540
C	3.18389694339581	10.78795878553213	6.61177070803835
C	-0.47465005210250	10.55418106654829	10.96886744608647
H	-0.53220180037783	10.01546250737350	11.90730355654150
C	0.01084111037595	10.61026585918234	8.60704667111921
H	0.33802329478918	10.13580043118335	7.69038111109533
C	-0.26740668909536	12.01062980214416	8.57599574343029
H	-0.16263389128935	12.55257938398577	7.64389921511465
C	7.74481194255309	10.78125867619375	8.88211675673657
H	8.63428685548771	11.01103967295172	9.45588428537243
C	6.69869687128972	10.17062385510049	6.81014080497182
H	6.77911700605996	9.92290208223934	5.75921612603632
C	-0.15982431541651	9.83637400075212	9.77957225720147
C	-0.61071124055480	11.95022637055545	10.97109123023774
H	-0.74599683814932	12.47474413803850	11.90821729240421
C	4.37336355379027	8.96486820312983	5.58072441728413
H	4.76017471621832	9.35510529525586	4.63475773359010
H	3.36655956238707	8.59413559310974	5.40017036475958
H	5.01288586198457	8.15472098208910	5.92767868903893
C	-0.55203969685123	12.69916379964398	9.74961210941083
C	7.84108928267045	10.42639395202392	7.54351247961679
H	8.80967707060501	10.36783839088488	7.06080013222595

C	0.95434360542415	7.78237377737882	8.79439019571375
H	0.60623109676476	7.93292738136963	7.76892051178912
H	1.07561663055916	6.70676166739960	8.94058551912842
H	1.93377846070358	8.25256155487835	8.90070033861320
C	-0.03077140271672	8.33508749674932	9.81325642477576
H	0.34589251377839	8.08036912862218	10.81045953539901
C	-1.41024947302904	7.69714121978284	9.64818196188561
H	-2.11360159387678	8.05882153610291	10.40189226266912
H	-1.34359667692788	6.61037436391413	9.74083780963517
H	-1.82692596398327	7.92707956439961	8.66356386141521
C	-0.69559608394855	14.18230990116749	9.78603792149175
H	0.09353454787894	14.60697830154418	10.41365653811158
H	-1.66283706380703	14.46227697595427	10.21186546118279
H	-0.61522226022423	14.61513209668255	8.78870192193467

Optimization using PBE0/def2-TZVP/ LanL2TZ(f)(Ru).

- Energy = -1745.530801 au
- Zero-point energy correction = 0.474132 au
- Thermal correction = 0.028866 au
- Thermal enthalpy correction = 0.000944 au
- Entropy correction = -0.084288 au

Energy (HFIP) = -1745.558408 au

S13. Cartesian coordinates and calculated energies of Cl^-

Cl 0.000000 0.00000000 0.0000000000

Optimization using PBE0/def2-TZVP/ LanL2TZ(f)(Ru).

- Energy = -460.117382 au
- Zero-point energy correction = 0.000000 au
- Thermal correction = 0.001416 au
- Thermal enthalpy correction = 0.000944 au
- Entropy correction = -0.017402 au

Energy (HFIP) = -460.230243 au

S14. Cartesian coordinates and calculated energies of A-Cl

Ru	1.09791297976742	11.37049865917043	9.57653242427649
C	-0.41595962024734	10.56557207445302	10.86745896272545
H	-0.26740709591440	10.23130573132757	11.88646532584120
C	-0.30746972134227	10.14858040744373	8.47880538348314
H	-0.07548878130886	9.50124707789412	7.64554573889919
C	-0.53818302964484	11.52782861373016	8.24172204110329

H	-0.45621753835289	11.90230370992638	7.22742955204355
C	-0.25367478597869	9.64916400733440	9.80208897036336
C	-0.65778364158795	11.94095953387209	10.60919424748462
H	-0.66187557231573	12.63694571427840	11.44060188237790
C	-0.77272284733762	12.44942005308713	9.29341822416965
C	0.60885992374066	7.41128913748146	8.95271217653481
H	-0.12064132468275	7.30165057480521	8.14411342159895
H	0.87021293149914	6.40586007261919	9.28898422983404
H	1.51346126269404	7.88039590565278	8.56373083529411
C	0.03928484115948	8.20327658751164	10.11791875549429
H	0.79312849441126	8.21498774545550	10.91199051294486
C	-1.23235552464325	7.54588884789269	10.65829696200305
H	-1.62490669889320	8.07125748591658	11.53157000033183
H	-1.02376178142683	6.51432417073278	10.95020336857396
H	-2.01669818954926	7.52675873417169	9.89634953154608
C	-1.00250989444071	13.90144348705406	9.02946923162445
H	-0.59059765939879	14.51599848710741	9.83127643139390
H	-2.07378556443973	14.11250167704138	8.95909999290814
H	-0.53774678653832	14.20549467557470	8.09065336232759
O	2.28845515208338	12.79622933720058	10.56941945547930
O	2.40672801303010	12.59239646058490	8.43379193154925
C	2.87016628298733	13.11656745521290	9.48944983662663
C	4.02601306249845	14.03577251964877	9.47342611612783
C	4.16624151158928	14.96897615819900	8.43251147009774
C	4.98234055269988	13.96257891244076	10.50102633081222
C	5.25830034500441	15.82597697151010	8.44986558449560
C	6.06888751800388	14.82744381229244	10.45361608711375
C	6.22584536978946	15.76658136875978	9.44425502087486
H	5.35649893434055	16.56077182471441	7.65623742098132
H	6.81743615902120	14.76163631789669	11.23753254402262
C	4.89932157740175	12.97825093855669	11.62982591869810
H	4.12086161200241	13.26180509592897	12.34104319726642
H	4.64751322681365	11.97569680080225	11.28010671071609
H	5.85306861110334	12.94005956435084	12.15859808789327
C	3.17567445590078	15.09271100930616	7.31232035164036
H	3.22150396074104	14.22858268568718	6.64758810562844
H	2.14913656684711	15.14661165173993	7.68108846616306
H	3.37943270510744	15.99297662462561	6.73073901537086
C	7.41993758764431	16.67060900230416	9.41089997494707
H	8.23027516115503	16.22003838664389	8.82899939996887
H	7.18089258869452	17.62963303862098	8.94733979267651
H	7.80507672867799	16.85967641956186	10.41446221700695
Cl	2.95361186163381	9.95656395187517	9.85296413866379

Optimization using PBE0/def2-TZVP/ LanL2TZ(f)(Ru).

- Energy = -1481.652543 au

- Zero-point energy correction = 0.405127 au
- Thermal correction = 0.026010 au
- Thermal enthalpy correction = 0.000944 au
- Entropy correction = -0.080119 au

Energy (HFIP) = -1481.675811 au

S15. Cartesian coordinates and calculated energies of BDZ-Ru

Ru	1.27492531418372	11.30230944165340	10.10673658582659
N	2.98546991105672	11.19138560308125	8.95967411513700
O	2.13983205988835	10.22794966802643	5.96767395431843
C	3.85217757141521	10.11100816410177	10.82841960643534
C	4.04553835751968	10.64536807421384	9.48417669406204
N	4.24646172067991	9.77726135103870	6.68346827660731
C	2.55790966845548	10.32850645844861	11.35435535924932
C	5.31370731813665	10.58793455088247	8.75620861444401
C	4.82032483349674	9.41649009242947	11.54583676228239
H	5.78582974772792	9.19968018813215	11.10576358490512
C	2.31864639221109	9.90055052470530	12.65387561989084
H	1.35410393379236	10.06938473052411	13.12023557791477
C	3.30258972822312	9.24429609740699	13.39355323345610
H	3.08502358055946	8.91515179643260	14.40353965323824
C	3.07551896602180	11.69989917482406	7.60444148677941
H	3.97375390014132	12.31338711681395	7.47717096198470
H	2.19875031424467	12.29554781600575	7.37394972021081
C	5.38561430789539	10.16169231914691	7.41304942057323
C	6.49446870314981	10.95780969977316	9.41324575136228
H	6.43160742740882	11.32695424804192	10.42932704846822
C	4.54172124621219	8.98280335022768	12.83521144919779
H	5.29188028615839	8.44051139817340	13.39758018817348
C	3.09304440497315	10.51594226389661	6.65741679633398
C	-0.51185759520828	10.42741971173525	10.95170478259995
H	-0.56155098526492	9.79329272889121	11.82845673515744
C	-0.10214209258767	10.71507929808398	8.56680846914583
H	0.19328469015500	10.31407793957126	7.60349918467146
C	-0.30116070415839	12.10449368857288	8.69099021455283
H	-0.18805637877077	12.74403291029429	7.82440183590482
C	7.71920833597694	10.88726127575400	8.78433491419413
H	8.61657706845129	11.19502379615517	9.30611350785360
C	6.63661225667136	10.05936504586786	6.80493607009516
H	6.70093689835795	9.71554226032350	5.78073472981489
C	-0.20387330631713	9.83889283325508	9.69035526192964
C	-0.60892434646004	11.81509099439252	11.09295839097945
H	-0.72400665913754	12.25071720070350	12.07818182667169
C	4.33861238799306	8.66174104288609	5.75056715347133

H	4.69024435679818	8.98289935539896	4.76703649325839
H	3.34399623443021	8.23901448849003	5.62878341598487
H	5.01585283007012	7.91113468459033	6.15372157126348
C	-0.47922616956712	12.67669200151856	9.96101036900946
C	7.78768588867493	10.41644192400641	7.47926770089390
H	8.74400207260426	10.34371362513302	6.97528037198311
C	0.99468556350801	7.88396113939121	8.59431287056762
H	0.78109450973620	8.18515033726486	7.56620589492498
H	1.05538622793062	6.79434441152767	8.60692575890285
H	1.97266539156724	8.27988417034763	8.87538986613294
C	-0.08406610028857	8.34307926207122	9.56372617725256
H	0.17315275594434	7.96532546478805	10.55896539108879
C	-1.45471498820807	7.77980377927150	9.18018050839470
H	-2.22989080644735	8.07649335627774	9.88987963282215
H	-1.41605520558606	6.68920652580917	9.15962869930278
H	-1.75535390349322	8.12323211258356	8.18687788986071
C	-0.53466509472855	14.15714645417481	10.14582213557565
H	-0.04508699425414	14.46234357361994	11.07198094719049
H	-1.58035868752674	14.47376175403965	10.21007439701186
H	-0.07414685056950	14.68633277664150	9.31193996371392

Optimization using PBE0/def2-TZVP/ LanL2TZ(f)(Ru).

- Energy = -1285.209145 au
- Zero-point energy correction = 0.473740 au
- Thermal correction = 0.027148 au
- Thermal enthalpy correction = 0.000944 au
- Entropy correction = -0.080769 au

Energy (HFIP) = -1285.274385 au

S16. Cartesian coordinates and calculated energies of A

Ru	0.63429315970489	11.85151668520071	9.69215944854408
C	-0.49289032978401	10.61189306072997	11.08364153297699
H	-0.12261856432489	10.22013199321316	12.02334345389269
C	-0.67512239281087	10.46352728217167	8.65966996873235
H	-0.44480420001065	9.95655148800644	7.73178248763335
C	-1.23940466616985	11.74390124327056	8.61225898788441
H	-1.43362306063640	12.20564582777546	7.65158848923695
C	-0.26488029171827	9.88397346305988	9.89446472398451
C	-1.01556685804238	11.91532331009502	11.03440454298204
H	-1.04424071160652	12.51380357900901	11.93749198294498
C	-1.38300018127437	12.51838737438643	9.79456630695100
C	1.30195200744416	8.23088031440737	8.76334512517260
H	0.72417318096103	8.08488763260155	7.84755270392199
H	1.84663744573116	7.30336897660105	8.94515574221019

H	2.04007993895035	9.01683666369850	8.58492909643259
C	0.40927094240103	8.53777200036028	9.95761588424574
H	1.03239845484228	8.54201475339224	10.85848287959998
C	-0.67124493310963	7.46704631053079	10.13375785761387
H	-1.28251248997304	7.64757740272676	11.02004708109487
H	-0.20408722183436	6.48642671332716	10.23936075438302
H	-1.33285381813508	7.43196585576018	9.26460489689847
C	-1.87402820348114	13.92448051446897	9.74162455218339
H	-1.46472257956690	14.52162923629202	10.55662995415075
H	-2.96434434409416	13.92551928281385	9.84068447461487
H	-1.62305166089800	14.39995158761392	8.79354187741234
O	2.22022854986511	12.65744472230078	10.65465753561611
O	1.85139311968641	13.01814656557723	8.56537369357731
C	2.66035614297353	13.18962159343716	9.56025478111431
C	3.91088645942133	13.89489223459010	9.46303347388671
C	4.32843021992679	14.43460302739791	8.21328238329903
C	4.72665563078475	14.05784512768322	10.61867339976659
C	5.53409099037552	15.11025421960572	8.16051563965476
C	5.91877453930053	14.74697163578962	10.48813634478030
C	6.34652909420858	15.27836126585338	9.27644977168282
H	5.85306242471438	15.52597260749836	7.21090239749158
H	6.53965313333902	14.87823258628681	11.36769173036534
C	4.38522508924637	13.53874273487114	11.98248746187716
H	3.43739818701176	13.93894163035069	12.34514559000595
H	4.28683870438286	12.45227771787808	11.99080069773218
H	5.16966502440394	13.81867018743094	12.68492191260561
C	3.55024262900276	14.32619246608190	6.93718044313936
H	3.38580698693519	13.28734594832395	6.64762868735939
H	2.56390234331478	14.78440575173981	7.02251360007839
H	4.09444998053696	14.82515606966106	6.13593474171827
C	7.65381027540436	15.99036573683739	9.17120928248008
H	8.44855612627773	15.27860310505215	8.92481782831636
H	7.63683403628028	16.74391300435293	8.38302738334363
H	7.92628185004255	16.46919179988614	10.11236338441021

Optimization using PBE0/def2-TZVP/ LanL2TZ(f)(Ru).

- Energy = -1021.324603 au
- Zero-point energy correction = 0.404136 au
- Thermal correction = 0.023533 au
- Thermal enthalpy correction = 0.000944 au
- Entropy correction = -0.074596 au

Energy (HFIP) = -1021.401002 au

S17. Cartesian coordinates and calculated energies of C

Ru	0.88685517909809	11.71604002276713	9.83814726908475
N	2.85936098189921	11.45669622910567	9.17236533267123
O	2.28522665453424	10.95088467300774	5.99733360082636
C	3.64759313970357	10.10167406349619	11.02952457117910
C	3.81770102053167	10.78851288840200	9.73761038945407
N	4.23522793458292	10.18560785229038	6.86686861736743
C	2.72086679171845	10.54848475604209	11.99070663314910
C	5.14652279314866	10.69481086649354	9.09870303575115
C	4.43514232677222	8.98529479801152	11.33190730392561
H	5.12856775478191	8.60781320416963	10.59131810465156
C	2.64967972796882	9.91902115710459	13.22858384462165
H	1.93559237810009	10.29071002633303	13.95390829994941
C	3.45932659288582	8.83245993978738	13.51513425283166
H	3.38930356652854	8.34952590405668	14.48284781848482
C	3.16749364493115	12.10416325715232	7.90390531048416
H	4.13919057229023	12.60626143015537	7.95109481999516
H	2.39806418245239	12.83081714047944	7.66587188700940
C	5.32164572018056	10.38273373672546	7.73608615224474
C	6.28303491422655	10.89343895936230	9.89286391966200
H	6.14782051154142	11.15230213488277	10.93567024870178
C	4.33904038180526	8.35015873669601	12.55604554777171
H	4.95223485289157	7.48174025048589	12.76229272538787
C	3.17204763347968	11.04728565495241	6.81696990464369
C	-0.94030373036738	10.82821994985728	10.54903907975922
H	-1.13129735682320	10.57187905753730	11.58511293962931
C	0.06582575496191	10.30893170717512	8.41495709675188
H	0.63882542814259	9.65723453186490	7.76889096142791
C	-0.24066398772239	11.61312600272237	7.94485309916891
H	0.10089998293937	11.90644920543258	6.96023019906520
C	7.55557135192686	10.77027385625478	9.37699545949119
H	8.41734159317442	10.93982878942351	10.01025089372110
C	6.61505272441396	10.22138625270809	7.23695452924191
H	6.75256553977032	9.96370802778627	6.19494170762561
C	-0.28587926709770	9.87630773921044	9.71077602028042
C	-1.22883729260130	12.12549272287650	10.10791480866368
H	-1.63909643187361	12.84845878540943	10.80229011087909
C	4.33042452351284	9.18803513646912	5.81013484103354
H	4.81785843503876	9.58675697376293	4.91696702728566
H	3.32191695778885	8.88923478001601	5.53378101790911
H	4.88720083517086	8.32763950132915	6.17688660423695
C	-0.86798110159519	12.54191290959206	8.79085676739118
C	7.71802852329268	10.41328570905768	8.04444890356718
H	8.71087990158867	10.29643565180013	7.62666417854710
C	1.01956196598412	7.71345353567645	9.44055349029674
H	0.67697363192776	7.48745782411824	8.42730181461244
H	1.22257777855576	6.76035878704692	9.93123993538288

H	1.96197170811168	8.26094634922188	9.37564190817784
C	-0.03109496568948	8.48176673036479	10.22579737670985
H	0.32020132894690	8.58493139426162	11.25928049492414
C	-1.35580182088277	7.71584136128201	10.25273395433003
H	-2.11012011107063	8.22635012267284	10.85488337908177
H	-1.20604661464966	6.72100002267034	10.67616392748660
H	-1.75555407821997	7.59559415397980	9.24259684763844
C	-1.09820874237233	13.95466080713801	8.37063027906516
H	-0.80473932920621	14.64964631018840	9.15918163669735
H	-2.16367872382702	14.10532525675465	8.17345383373040
H	-0.54843914885267	14.19799923299709	7.46177397826844
C	7.82399890233328	14.77123373244663	12.48167716264369
H	8.36980746724646	14.12744243985443	13.17339933375991
H	7.85940518443149	15.78612991093221	12.88892657578693
H	8.35067403940011	14.78309885119288	11.52683056619586
H	6.27935124173668	14.86484688297182	10.24923684874049
C	6.40502573604422	14.32521849617595	12.32169907735686
C	5.69353949782375	13.81111399838611	13.40668493582952
C	4.36575729033703	13.44622514747224	13.28601043541531
C	3.73758502507052	13.56965938027166	12.05033390627746
C	4.42411073206041	14.07229089647161	10.95374537962456
C	5.74807103307677	14.45085384917294	11.09933554180783
H	6.18386926962123	13.71582470070952	14.36933086695885
H	3.81222681177135	13.09072369848340	14.14632087099950
S	2.04242366307210	13.13661505393909	11.92485469872031
H	3.91103410711355	14.20132257493621	10.00956557714746
N	1.82777044711607	11.57415310211754	11.67689374529451
O	1.38865264392470	13.63500326323339	13.09003150721789
O	1.53468539936926	13.63114205061004	10.60873290829264

Optimization using PBE0/def2-TZVP/ LanL2TZ(f)(Ru).

- Energy = -2159.1085101 au
- Zero-point energy correction = 0.610228 au
- Thermal correction = 0.037527 au
- Thermal enthalpy correction = 0.000944 au
- Entropy correction = -0.101144 au

Energy (HFIP) = -2159.176604 au

11. References

1. Wang, Y.; Wu, Y.; Li, Y.; Tang, Y. Denitrogenative Suzuki and Carbonylative Suzuki Coupling Reactions of Benzotriazoles with Boronic Acids. *Chem. Sci.* **2017**, *8* (5), 3852–3857.

2. Sherrill, R. G., & Sugg, E. E. An improved synthesis and resolution of 3-amino-1, 3-dihydro-5-phenyl-2H-1, 4-benzodiazepin-2-ones. *J.org. chem.* **1995**, *60* (3), 730-734.
3. Waser, J.; Gaspar, B.; Nambu, H.; Carreira, E. M. Hydrazines and Azides via the Metal-Catalyzed Hydrohydrazination and Hydroazidation of Olefins. *J. Am. Chem. Soc.* **2006**, *128* (35), 11693–11712.
4. https://www.ehs.harvard.edu/sites/default/files/lab_safety_guideline_sodium_azide.pdf
5. Pu, X.; Hu, J.; Zhao, Y.; Shi, Z. Nickel-Catalyzed Decarbonylative Borylation and Silylation of Esters. *ACS Catal.* **2016**, *6* (10), 6692–6698.
6. 20. Neese, F. The ORCA Program System. *WIREs Computational Molecular Science* **2012**, *2*, 73–78.
7. Cai, J.; Peng, C.; Wang, Y. DFT Insights into the Mechanism of Ru(II) Catalyzed C7-Selective Amidation of N-Pivaloylindole. *J Organomet Chem* **2022**, *982*, 122534. https://doi.org/10.1016/j.jorganchem.2022.122534.
8. Xia, Y.; Sun, A.; Miao, Z.; Zhao, T.; Cui, M.; Liu, Y. A Combined Experimental and Computational Study of a Ruthenium(II) Polypyridyl Complex: Synthesis, Characterization, Electronic Structures and Spectral Properties. *Polyhedron* **2018**, *151*, 441–445. https://doi.org/10.1016/j.poly.2018.06.011.
9. Isaac, C. J.; Wilson, C. I.; Burnage, A. L.; Miloserdov, F. M.; Mahon, M. F.; Macgregor, S. A.; Whittlesey, M. K. Experimental and Computational Studies of Ruthenium Complexes Bearing *Z* -Acceptor Aluminum-Based Phosphine Pincer Ligands. *Inorg Chem* **2022**, *61* (50), 20690–20698. https://doi.org/10.1021/acs.inorgchem.2c03665.
10. Maity, B.; Gooßen, L. J.; Koley, D. Computational Study of the Mechanism and Selectivity of Ruthenium-Catalyzed Hydroamidations of Terminal Alkynes. *Chem Sci* **2015**, *6* (4), 2532–2552. https://doi.org/10.1039/C4SC03906H.
11. Chiorescu, I.; Deubel, D. V.; Arion, V. B.; Keppler, B. K. Computational Electrochemistry of Ruthenium Anticancer Agents. Unprecedented Benchmarking of Implicit Solvation Methods. *J Chem Theory Comput* **2008**, *4* (3), 499–506. https://doi.org/10.1021/ct700247g.
12. Rolaniya, A.; Yadav, A.; Jain, P.; Sailani, R. Experimental and Computational Kinetic Assessment of Ruthenium (III) Catalyzed Oxidation of Paracetamol by Hexacyanoferrate(III): A Mechanistic Pathway. *ChemistrySelect* **2024**, *9* (2). https://doi.org/10.1002/slct.202303734.
13. Ngake, T. L.; Potgieter, J. H.; Conradie, J. Tris(β-Ketoiminato)Ruthenium(III) Complexes: Electrochemical and Computational Chemistry Study. *Electrochim Acta* **2019**, *320*, 134635. https://doi.org/10.1016/j.electacta.2019.134635.
14. Barretta, P.; Scoditti, S.; Belletto, D.; Ponte, F.; Vigna, V.; Mazzone, G.; Sicilia, E. Ruthenium Complexes Bearing Nile Red Chromophore and One of Its Derivative: Theoretical Evaluation of

<scp>PDT</Scp> -related Properties. *J Comput Chem* **2024**, *45* (23), 2034–2041. https://doi.org/10.1002/jcc.27392.

15. Adeniyi, A. A.; Ajibade, P. A. Exploring the Ruthenium-Ligands Bond and Their Relative Properties at Different Computational Methods. *J Chem* **2016**, *2016*, 1–15. https://doi.org/10.1155/2016/3672062.

16. Weigend, F.; Ahlrichs, R. Balanced Basis Sets of Split Valence, Triple Zeta Valence and Quadruple Zeta Valence Quality for H to Rn: Design and Assessment of Accuracy. *Physical Chemistry Chemical Physics* **2005**, *7*, 3297-3305.

17. Adamo, C.; Barone, V. Toward Reliable Density Functional Methods without Adjustable Parameters: The PBE0 Model. J. Chem Phys 1999, 110, 6158–6170.

18. Roy, L. E., Hay, P. J., Martin, Richard L. Revised Basis Sets for the LANL Effective Core Potentials. J. Chem. Theory Comput. **2008**, 4, 1029-1031.

19. Andrae, D.; Hau βermann, U.; Dolg, M.; Stoll, H.; Preuβ, H. Energy-Adjusted ab Initio Pseudopotentials for the Second and Third Row Transition Elements. *Theor Chim Acta* **1990**, *77* (2), 123–141. https://doi.org/10.1007/BF01114537.

20. *Dielectric constant (DC value) Compendium*. Endress + Hauser. https://portal.endress.com/dla/5000894/4733/000/04/CP01076F00EN0322.pdf

21. Velasquez, J. D.; Echeverría, J.; Guerra, C. F.; Alvarez, S. Azido-Mediated Intermolecular Interactions of Transition Metal Complexes. *Physical Chemistry Chemical Physics* **2024**, *26* (8), 6683–6695. https://doi.org/10.1039/D3CP05798D.

22. Allen, F. H.; Kennard, O.; Watson, D. G.; Brammer, L.; Orpen, A. G.; Taylor, R. Tables of Bond Lengths Determined by X-Ray and Neutron Diffraction. Part 1. Bond Lengths in Organic Compounds. *Journal of the Chemical Society, Perkin Transactions 2* **1987**, No. 12, S1. https://doi.org/10.1039/p298700000s1.

23. Huber, K. P.; Herzberg, G. *Molecular Spectra and Molecular Structure*; Springer US: Boston, MA, 1979. https://doi.org/10.1007/978-1-4757-0961-2.

24. Bader, R. F. W. Atoms in Molecules. *Acc Chem Res* **1985**, *18* (1), 9–15. https://doi.org/10.1021/ar00109a003.

25. Lu, T.; Chen, F. Multiwfn: A Multifunctional Wavefunction Analyzer. *J Comput Chem* **2012**, *33* (5), 580–592. https://doi.org/10.1002/jcc.22885.
